# The HIV Treatment Gap: Estimates of the Financial Resources Needed versus Available for Scale-Up of Antiretroviral Therapy in 97 Countries from 2015 to 2020

**DOI:** 10.1371/journal.pmed.1001907

**Published:** 2015-11-24

**Authors:** Arin Dutta, Catherine Barker, Ashley Kallarakal

**Affiliations:** Palladium, Washington, District of Columbia, United States of America; Centers for Disease Control and Prevention, UNITED STATES

## Abstract

**Background:**

The World Health Organization (WHO) released revised guidelines in 2015 recommending that all people living with HIV, regardless of CD4 count, initiate antiretroviral therapy (ART) upon diagnosis. However, few studies have projected the global resources needed for rapid scale-up of ART. Under the Health Policy Project, we conducted modeling analyses for 97 countries to estimate eligibility for and numbers on ART from 2015 to 2020, along with the facility-level financial resources required. We compared the estimated financial requirements to estimated funding available.

**Methods and Findings:**

Current coverage levels and future need for treatment were based on country-specific epidemiological and demographic data. Simulated annual numbers of individuals on treatment were derived from three scenarios: (1) continuation of countries’ current policies of eligibility for ART, (2) universal adoption of aspects of the WHO 2013 eligibility guidelines, and (3) expanded eligibility as per the WHO 2015 guidelines and meeting the Joint United Nations Programme on HIV/AIDS “90-90-90” ART targets. We modeled uncertainty in the annual resource requirements for antiretroviral drugs, laboratory tests, and facility-level personnel and overhead.

We estimate that 25.7 (95% CI 25.5, 26.0) million adults and 1.57 (95% CI 1.55, 1.60) million children could receive ART by 2020 if countries maintain current eligibility plans and increase coverage based on historical rates, which may be ambitious. If countries uniformly adopt aspects of the WHO 2013 guidelines, 26.5 (95% CI 26.0 27.0) million adults and 1.53 (95% CI 1.52, 1.55) million children could be on ART by 2020. Under the 90-90-90 scenario, 30.4 (95% CI 30.1, 30.7) million adults and 1.68 (95% CI 1.63, 1.73) million children could receive treatment by 2020. The facility-level financial resources needed for scaling up ART in these countries from 2015 to 2020 are estimated to be US$45.8 (95% CI 45.4, 46.2) billion under the current scenario, US$48.7 (95% CI 47.8, 49.6) billion under the WHO 2013 scenario, and US$52.5 (95% CI 51.4, 53.6) billion under the 90-90-90 scenario. After projecting recent external and domestic funding trends, the estimated 6-y financing gap ranges from US$19.8 billion to US$25.0 billion, depending on the costing scenario and the U.S. President’s Emergency Plan for AIDS Relief contribution level, with the gap for ART commodities alone ranging from US$14.0 to US$16.8 billion.

The study is limited by excluding above-facility and other costs essential to ART service delivery and by the availability and quality of country- and region-specific data.

**Conclusions:**

The projected number of people receiving ART across three scenarios suggests that countries are unlikely to meet the 90-90-90 treatment target (81% of people living with HIV on ART by 2020) unless they adopt a test-and-offer approach and increase ART coverage. Our results suggest that future resource needs for ART scale-up are smaller than stated elsewhere but still significantly threaten the sustainability of the global HIV response without additional resource mobilization from domestic or innovative financing sources or efficiency gains. As the world moves towards adopting the WHO 2015 guidelines, advances in technology, including the introduction of lower-cost, highly effective antiretroviral regimens, whose value are assessed here, may prove to be “game changers” that allow more people to be on ART with the resources available.

## Abstract 2

Arin Dutta and colleagues project global ART-related funding, global ART-related need under established targets, and the gap between the two.

## Editors' Summary

**Background:**

AIDS has killed 39 million people since 1981, and over 36 million people (mostly living in resource-limited countries) are currently living with HIV, the virus that causes AIDS. HIV destroys immune system cells (including CD4 lymphocytes), leaving HIV-infected individuals susceptible to other infections. Early in the AIDS epidemic, most HIV-infected individuals died within ten years of infection. Then, in 1996, effective antiretroviral therapy (ART) became available. For people living in affluent countries, HIV/AIDS became a chronic condition, but it remained fatal for people living in resource-limited countries. In 2003, the international community began to work towards universal access to ART. Now, more than a third of people living with HIV are receiving ART, and the global death rate from HIV/AIDS is falling. The “90-90-90” targets recently set by UNAIDS—90% of all people living with HIV knowing their status, 90% of all people diagnosed with HIV infection receiving ART, and 90% of all people receiving ART having robust viral suppression by 2020—aim to end the AIDS epidemic by 2030.

**Why Was This Study Done?:**

Because early ART initiation both improves the health of HIV-positive individuals and reduces HIV transmission, the latest WHO guidelines (September 2015) recommend that people living with HIV should initiate ART upon diagnosis, regardless of their CD4 count. Previous WHO guidelines recommended initiating ART in certain populations based on CD4 count; for instance, the 2013 guidelines recommended adults living with HIV start ART only when their CD4 count fell below 500 cells/mm^3^ blood. Following the latest guidelines and meeting the UNAIDS targets will involve a rapid scale-up of ART, but are the financial resources available for this scale-up? Here, the researchers use mathematical modeling to estimate the numbers of people eligible for and receiving ART in 97 countries from 2015 to 2020 and the facility-level financial resources needed under three ART scale-up scenarios. Facility-level financial resources cover the costs of antiretroviral drugs, laboratory testing, and facility-level personnel and overhead (e.g., utilities, support staff, transportation, and other supplies), but not costs above the facility level or the costs of other services that are often integrated into ART provision. The facility-level ART costs are compared to the estimated financial resources available from domestic and external sources to assess the ART funding gap in the 97 countries.

**What Did the Researchers Do and Find?:**

The researchers estimated current ART coverage levels and future treatment needs using country-specific epidemiological and demographic data for 97 countries. They estimated annual numbers of people eligible for and receiving treatment assuming that countries continued to follow their current criteria for treatment (scenario 1), universally adopted aspects of the WHO 2013 eligibility criteria (scenario 2), or expanded eligibility to meet the WHO 2015 guidelines and the 90-90-90 targets (scenario 3) while increasing the percentage of persons reached based on historical rates. Under scenario 1, 25.7 million adults and 1.57 million children living with HIV could receive ART by 2020. Under scenarios 2 and 3, 26.5 million and 30.4 million adults, respectively, and 1.53 million and 1.68 million children, respectively, could receive ART by 2020. The estimated facility-level financial resources needed for ART scale-up from 2015 to 2020 were US$45.8 billion, US$48.7 billion, and US$52.5 billion under scenarios 1, 2, and 3, respectively. Finally, the estimated gap over six years between the resources needed for ART scale-up and the domestic and external financial resources available ranged from US$19.8 billion to US$25.0 billion depending on the eligibility scenario and the level of ART support provided by PEPFAR (the U.S. President’s Emergency Plan for AIDS Relief) and the Global Fund to Fight AIDS, Tuberculosis and Malaria, two major external ART funding sources.

**What Do These Findings Mean?:**

The accuracy of this study’s findings may be limited by the quality of country- and region-specific data, assumptions made about the funding available for ART, and the fact that only the facility-level costs of ART provision were considered. However, the estimates of the number of people receiving ART by 2020 suggest that countries are unlikely to meet the 90-90-90 treatment target (81% of people living with HIV on ART by 2020; that is, 90% of people living with HIV diagnosed and 90% of diagnosed patients receiving ART) unless countries expand ART access to all people living with HIV and rapidly increase coverage. Moreover, the estimated future resource needs for ART scale-up are large enough to threaten the sustainability of the global response to AIDS unless additional resources are made available through domestic or external financing sources or through efficiency gains. Thus, the researchers conclude, advances in technology and the introduction of new lower-cost antiretroviral regimens could be essential to allow more people to receive ART with the resources available.

**Additional Information:**

This list of resources contains links that can be accessed when viewing the PDF on a device or via the online version of the article at http://dx.doi.org/10.1371/journal.pmed.1001907.

- Information is available from the US National Institute of Allergy and Infectious Diseases on HIV infection and AIDS
- NAM/aidsmap also provides basic information about HIV/AIDS, summaries of recent research findings on HIV care and treatment, and personal stories about living with HIV/AIDS
- Information is available from Avert, an international AIDS charity, on many aspects of HIV/AIDS, including information on universal access to ART, the 90-90-90 targets, and starting, monitoring, and switching ART; Avert also provides personal stories about living with HIV/AIDS
- WHO provides information on all aspects of HIV/AIDS, including its 2013 ART guidelines and its early-released 2015 ART guidelines
- The UNAIDS Fast-Track Strategy to End the AIDS Epidemic by 2030 provides up-to-date information about the AIDS epidemic, including progress towards universal access to ART; UNAIDS also provides detailed information about its 90-90-90 treatment targets
- The Health Policy Project examines ways to make existing funds for ART go further and to maximize the effectiveness and efficiency of ART programs; information on PEPFAR and on the Global Fund to Fight AIDS, Tuberculosis and Malaria is available

## Introduction

Recent global initiatives have renewed focus on rapidly scaling up antiretroviral therapy (ART) to prevent AIDS-related deaths and HIV transmission. In September 2015, the World Health Organization (WHO) early-released revised guidelines recommending that all people living with HIV be offered and voluntarily initiate ART upon diagnosis, regardless of CD4 T cell count. This recommendation urges countries to expand ART eligibility beyond recommendations made in the WHO 2013 guidelines, based on new evidence of the improved health and epidemiological benefits of initiating treatment earlier in the course of the disease [1,2]. The number of patients receiving ART and the related resource requirements could increase in countries that adopt the 2015 guidelines and raise ART coverage for the eligible patient group. These new guidelines are aligned with the recent Joint United Nations Programme on HIV/AIDS (UNAIDS) treatment targets to end the global HIV epidemic by 2030. These targets, collectively named 90-90-90, call for 90% of all people living with HIV to know their HIV status, 90% of all people diagnosed with HIV to receive sustained ART, and 90% of all people receiving ART to have durable viral suppression by 2020 [3]. Concurrent with the 90-90-90 announcement, the U.S. President’s Emergency Plan for AIDS Relief (PEPFAR) and the Children’s Investment Fund Foundation launched Accelerating Children’s HIV/AIDS Treatment (ACT) in July 2014. This US$200 million initiative aims to double the number of children receiving ART across nine priority African countries in 2 y [4]. Prior to the early release of the WHO 2015 guidelines, global leaders at the 2014 International AIDS Society Conference signed onto the Vancouver Consensus, which states that all people living with HIV should have access to treatment, further supporting the 90-90-90 initiative’s call to rapidly scale up ART coverage [5]. 90-90-90 and ACT have set ambitious targets. However, just 48% and 37% of all people living with HIV in 2013 were aware of their status and receiving ART, respectively [6]. Pediatric treatment is lagging in particular, with only 32% of children living with HIV receiving ART as of 2013 [7].

The early-released WHO 2015 guidelines and the 90-90-90 and ACT initiatives suggest initiating treatment in all people living with HIV at any CD4 T cell count. Currently, constrained resources have required most countries with a high HIV burden to prioritize treatment for only those individuals most in need, based on specific disease or demographic characteristics of groups or individuals. ART is predominantly supported by external funding in low- and lower-middle-income countries, and funding levels have plateaued in recent years [6]. The WHO guidelines on the use of antiretroviral (ARV) drugs for treating and preventing HIV infection serve as an important framework for national HIV treatment policies that define eligibility for initiating treatment, indicate preferred drug regimens, and define the type and frequency of laboratory tests conducted to monitor treatment success. In the 2013 guidelines, WHO recommended expanding eligibility—e.g., increasing the CD4 T cell count threshold, below which HIV-positive individuals are eligible to start treatment, from 350 to 500 cells/mm^3^ for all adults and children over age 5 y [2]. Many countries have yet to fully adopt these guidelines or increase the percentage of people reached against a denominator of need based on the new, expanded eligibility criteria [6]. The early-released WHO 2015 guidelines recommend further expansion in eligibility to all people living with HIV, and acknowledge that a phased approach to expanding eligibility may be needed in many countries with limited capacity and constrained resources [1].

Scaling up ART to ambitious levels will have significant cost implications. Only a few studies have projected the global resources needed for ART, an exercise that requires modeling the number of people expected to receive HIV treatment annually under different treatment eligibility and baseline coverage assumptions, in addition to estimating the unit cost of ART per patient per year. Several of these recent studies have limitations. They make limited use of country- or region-specific data, despite significant variance in unit costs and HIV treatment policies across countries, or do not account for uncertainty in the future coverage or unit costs of treatment [8–12]. Additionally, based on our review of the literature, there are no published studies that model future global resource requirements for pediatric and adult HIV treatment separately.

Of the existing studies that projected ART resource requirements, one estimated that the annual cost of maintaining treatment for those currently receiving ART through the Global Fund to Fight AIDS, Tuberculosis and Malaria (Global Fund) will be US$1.7 billion in 2020, assuming 2.3 million people living with HIV will be on ART and supported by the Global Fund that year [9]. Another study estimated that at least US$22 billion will be needed for HIV prevention, treatment, care, and support in 2015, assuming that 13.1 million people globally are on ART [10]. While this study used country- or region-specific epidemiological and cost data, the data were not recent. Cost data were from 2009, when ARV drug prices were significantly higher, and treatment eligibility was based on the 2010 WHO recommendations [10,13]. Further, by assuming that 80% of all those eligible for treatment would receive it in 2015, this analysis did not account for possible variations in future coverage due to different starting points [10]. A more recent study estimated that global adoption of the 2013 WHO guidelines would cost an additional US$1.8 billion in 2015 and US$3.3 billion in 2020, compared to maintaining current eligibility criteria based on the 2010 WHO guidelines [8]. This analysis appears limited by its use of the same unit cost for ART across all countries and its assumption of simultaneous and universal achievement of a target coverage rate. UNAIDS and the Global Fund have also estimated HIV resource needs. For 2020, UNAIDS estimates that the global HIV response will require US$9.7 billion, US$8.7 billion, and US$17.2 billion in low-, lower-middle-, and upper-middle-income countries, respectively [11]. The detailed assumptions behind these cost estimations are unavailable. In an analysis leading up to its fourth replenishment, spanning 2014 to 2016, the Global Fund forecasted that US$58 billion was needed for HIV response in countries eligible for Global Fund funding [12]. Similar to other modeling analyses, the Global Fund’s projections do not provide ART costs separately from those for the total HIV response. Additionally, it used historical cost data and defined universal ART coverage as 80% of those eligible for treatment receiving treatment [12].

All of these studies indicate that domestic and international funding for the global HIV response will need to increase and be sustained at higher levels as eligibility for treatment and coverage expand. Funding levels for the HIV response have remained relatively constant in recent years, and the future financial landscape is uncertain [6,14]. In low- and middle-income countries, US$19.1 billion was invested in the HIV and AIDS response in 2013, of which US$9.7 billion was estimated to be from domestic resources [6]. Donors like PEPFAR and the Global Fund are placing greater emphasis on shared responsibility and a country-led approach in the HIV response, which may require greater resource commitments from countries and regions that rely heavily on external funding for ART [14,15].

Given funding constraints for the global HIV response, it is critical to improve resource requirement and funding estimates for ART. This study seeks to fill data gaps by estimating the number of adults and children eligible for and receiving first- and second-line ART in 97 countries from 2015 to 2020, using recent epidemiological data and accounting for uncertainty in the baseline and projected numbers of people eligible for treatment, future coverage scale-up, and annual migration to second-line treatment, in addition to other factors. We also conduct an uncertainty analysis of the unit costs for ART services—medication, laboratory tests, and facility-level overhead and personnel—using recent data specific to country income levels. We compare the resource requirements to projections of available external and domestic resources for facility-level ART to assess the financial sustainability of the global ART response.

## Methods

We estimated the number of adults and children eligible for and receiving HIV treatment, as well as the cost of providing ART, in 97 countries across six regions (Western and Central Africa, Eastern and Southern Africa, Latin America and the Caribbean, Eastern Europe and Central Asia, the Middle East and North Africa, and Asia and the Pacific) and four per capita gross national income levels: low-income countries (LICs), lower-middle-income countries (LMICs), upper-middle-income countries (UMICs), and high-income countries (HICs) (see Table 1).

**Table 1:** Countries included in the study by region and country income level.

Country Income Level	Western and Central Africa	Eastern and Southern Africa	Latin America and the Caribbean	Eastern Europe and Central Asia	Middle East and North Africa	Asia and the Pacific
**Low income**	Benin, Burkina Faso, Central African Republic, Chad, Democratic Republic of the Congo, Gambia, Guinea, Guinea Bissau, Liberia, Mali, Niger, Sierra Leone, Togo	Burundi, Comoros, Eritrea, Ethiopia, Madagascar, Malawi, Mozambique, Rwanda, South Sudan, Tanzania, Uganda, Zimbabwe	Haiti		Somalia	Cambodia, Nepal
**Lower-middle income**	Cameroon, Congo, Côte d’Ivoire	Kenya, Lesotho, Swaziland, Zambia	Bolivia, Guatemala, Guyana, Honduras, Nicaragua	Armenia, Georgia, Kyrgyzstan, Moldova, Tajikistan, Ukraine, Uzbekistan	Djibouti, Egypt, Morocco, Sudan, Yemen	Bangladesh, Bhutan, India, Indonesia, Lao People’s Democratic Republic, Myanmar, Pakistan, Papua New Guinea, Philippines, Sri Lanka, Viet Nam
**Upper-middle income**	Gabon, Ghana, Mauritania, Nigeria, Senegal	Angola, Botswana, Mauritius, Namibia, South Africa	Belize, Cuba, Dominican Republic, Jamaica, Suriname	Azerbaijan, Belarus, Bulgaria, Kazakhstan, Romania, Serbia	Algeria, Iran, Tunisia	China, Malaysia, Mongolia, Thailand
**High income**	Equatorial Guinea		Bahamas, Barbados, Trinidad and Tobago	Russia		

Of the 188 countries that have in recent years reported data to UNAIDS, we excluded (1) 42 countries that had fewer than 1,000 people living with HIV or did not have an estimate for this number as of December 2013, (2) 31 countries that were members of the Organisation for Economic Co-operation and Development in 2014 (membership indicates a country is high-income and able to pay for its entire HIV response), (3) 15 countries that had other inadequate or incomplete data for analysis, and (4) three countries with low or moderate HIV epidemics that are ineligible for financing from the Global Fund. The final set of 97 countries included those eligible for Global Fund grant funding, as well as a few ineligible countries with significant HIV epidemics.

We developed three scenarios for analysis based on current eligibility and various emphases on expanding eligibility for treatment. The first scenario (current eligibility) assumes that countries will maintain current eligibility guidelines from 2015 to 2020. Based on official files, some countries plan to expand eligibility in the coming years, sometimes beyond the thresholds recommended in the 2013 WHO guidelines. Others will maintain less expanded eligibility thresholds primarily based on the 2010 WHO guidelines [16]. The second scenario (WHO 2013) assumes uniform adoption of certain aspects of the 2013 WHO guidelines across all countries in our study beginning in 2014, as discussed below. This scenario examines the impact of a harmonized standard for treatment eligibility. The third scenario (90-90-90) is based on expanding eligibility as per the early-released WHO 2015 guidelines and achieving the UNAIDS 90-90-90 targets. This scenario assumes that all people diagnosed with HIV are eligible for treatment. Scale up in coverage across all three scenarios is based on countries' historical rates of increase, adjusted for changes in eligibility. These increases in coverage may be ambitious for some countries that experienced rapid ART scale-up in recent years.

Across all scenarios, we modeled underlying uncertainty in the numbers of people eligible for treatment, coverage of treatment, and the unit cost of various treatment inputs per person. A parametric uncertainty analysis was not in the original analysis plan but emerged during the peer review process. Uncertainty in future estimates of numbers of individuals on ART and the costs of these services derives from uncertain estimates of the number of people living with HIV and needing treatment, future coverage achieved by countries, switching rates from first- to second-line ART, future unit costs, as well as other parameters. The range of uncertainty in projections of future populations eligible for ART and the number of people living with HIV depends fundamentally on the methods and quality of data used to estimate national prevalence curves from historic point HIV prevalence estimates in sentinel surveillance and population-based surveys [17]. The Spectrum AIDS Impact Model (AIM) is an internationally recognized modeling platform designed to forecast HIV epidemiological trends based on demographic and epidemiological parameters specific to each country’s epidemic, and is used by UNAIDS for its published estimates and by individual countries [6,8,10,18–20]. It has been described formally elsewhere [21]. Official AIM estimates derive HIV prevalence and incidence using the Estimation and Projection Package (EPP). The EPP analysis is based on HIV sentinel surveillance and population-based surveys, integrated bio-behavioral surveillance surveys, and assumptions about epidemic pattern by country [21]. See S1 Text for more details on the EPP and AIM Country Data Package in Spectrum. AIM builds on EPP and tracks adults living with HIV by age, sex, and CD4 T cell count. There are seven CD4 T cell count categories, ranging from less than 50 to more than 500 cells/mm^3^. From year to year, a person can stay in the same CD4 T cell count category, move to the next lower category, die from HIV-related causes, die from non-HIV causes, or initiate ART. The parameters for these transitions are based on cohort data from the Analysing Longitudinal Population-Based HIV/AIDS Data on Africa (ALPHA) network [21]. Mortality for those on ART is based on an analysis by the International Epidemiologic Databases to Evaluate AIDS consortium and depends on CD4 T cell count at the time of ART initiation, duration on treatment, sex, and age [22]. Children living with HIV are also tracked by age and CD4 T cell count. Their mortality rate depends on when they acquire HIV and whether or not they receive ART and/or co-trimoxazole.

We conducted probabilistic sensitivity analysis to examine the impact of uncertain epidemiological, coverage, and unit cost inputs in the model. Expected mean values were derived from sampling events in 5,000 second-order Monte Carlo simulation trials performed using RiskAMP software (Structured Data LLC) integrated with Microsoft Excel [23]. Each simulation was run simultaneously over all uncertain parameters. The simulation utilized distribution types with bounds and modes derived from country-specific Spectrum and/or other secondary data as described in Table 2 (also see S1 and S2 Texts). Confidence intervals were estimated for most results to describe the overall uncertainty in the estimates. Uncertainty bounds for future financial resources available for HIV were not similarly quantifiable, and hence were not modeled. Deterministic analysis for certain types of funding sources, such as PEPFAR, was performed where feasible and is discussed further below. Most data are from third-party sources including government agencies, watchdog organizations, and external funding organizations. Additional details on data sources and data accessibility can be found in S5 Text.

**Table 2:** Parameters used in uncertainty analysis.

Category	Variable	Description of Range Used in Simulation Analysis	Disaggregation Level	Distribution	Sources
**Epidemiological and coverage parameters and inputs**					
	**Annual number of adults 15+ and children 0–15 living with HIV who need ART, by scenario**				
	Current eligibility	Median and 95% CI from Spectrum uncertainty analysis, given country-specific current eligibility guidelines^1^	Country	Beta PERT	[16]
	WHO 2013	Median and 95% CI from Spectrum uncertainty analysis, given selected elements of WHO 2013 eligibility guidelines^2^	Country	Beta PERT	[2,16]
	90-90-90	Median and 95% CI from Spectrum uncertainty analysis of adults and children living with HIV	Country	Beta PERT	[3,16]
	Baseline coverage: 2013 (percent)	Calculated: actual number on ART ÷ simulation mean of number eligible, December 2013	Country	Beta PERT	[16]
	**Annual rate of change in percentage coverage for adult and pediatric ART (percent), by scenario**				
	Current eligibility	Lower bound based on CAGR^3^ from 2011 to 2013 of country-specific coverage with past eligibility as denominator, mode as the median CAGR of the region 2011–2013, and higher bound as the weighted average CAGR of the region over 2011–2013	Country	Beta PERT	[16,24]
	WHO 2013	Range based on current eligibility scenario, adjusted by a factor of 0.66 for adults	Country	Beta PERT	[16,24]
	90-90-90	Lower bound based on CAGR^3^ from 2011 to 2013 of country-specific coverage with the number of people living with HIV as denominator, mode as the median CAGR of the region 2011–2013, and higher bound as the weighted average CAGR of the region over 2011–2013	Country	Beta PERT	[16,24]
	Baseline first-/second-line ART split	Actual reported country-specific or regional splits based on 2013 data	Country	N/A	[16]
	**Year-to-year changes in numbers on second-line treatment**				
	Mortality on second-line ART	Lower bound: annual mortality rate for patients with CD4 cell count > 350 cells/m^3^, upper bound: annual mortality rate for patients with CD4 cell count ≤ 350 cells/m^3^	Epidemiological region	Uniform	[16,18,22]
	Migration from first- to second-line ART	Range based on literature review on resistance, retention, and other predictive factors (data by country)	Epidemiological region	Beta PERT	[25–32]
**Cost parameters and inputs**					
	**Proportional distribution of patients on ARV regimens**	Based on baseline in 2014 of the proportion of patients receiving each regimen by income group, adult/pediatric, and line of ART; change in proportions over 2015–2020 based on market analysis	Country income group and zone (separates Africa)	N/A (deterministic)	[26,33]
	**Baseline ARV regimen cost per patient-year (2014 US$)**	Range based on lowest, median, and highest price of regimen paid recently among countries in income group (Africa versus non-Africa), separated for adults/pediatric and first-/second-line ARV	Country income group and zone (separates Africa)	Beta PERT	[34–40]
	**Annual percentage change in ARV drug prices (cost per patient-year)**				
	Adult first-line ARVs	Lower bound of zero (assumed to be at price floor), mode based on market analyses, and upper bound as the historical average decline per year over 2010–2013	Country income group and zone (separates Africa)	Beta PERT	[33–38, 40]
	Adult second-line, pediatric first-line, and pediatric second-line ARVs	Lower bound and mode based on market analyses of price declines across originator and generic producers (considering income group and generics accessibility and entry of new producers), and upper bound as the historical average decline per year over 2010–2013; see S1 Text for detailed data	Country income group and zone (separates Africa)	Beta PERT	[26,34–38,40]
	**Baseline cost per laboratory test (2014 US$)**	Costs separated by tests for CD4 count, viral load, hematology, and biochemistry (panel); lower bound and mode based on literature review and country-specific data, and upper bound determined by maximum cost as seen by income group; see S3 Text for detailed data	Country income groups and zone (separates Africa)	Beta PERT	[28,41–53]
	**Annual decrease in cost per test**	Lower bound of zero, mode based on literature review (varies from 2% to 6.6% per year), and upper bound based on literature review and assumption of reaching lowest production cost	Country income group and zone (separates Africa)	Beta PERT	[45,46,48–50,52]
	**Facility-level labor and overhead unit costs**	Based on literature review: lower bound as minimum of values seen by income group, mode as median, and upper bound as the maximum	Country income group	Beta PERT	[53–64]
	**One-time scale efficiency gains factor**	Lower bound: 0.5–0.8 for HICs and UMICs, 0.85–0.95 for LICs and LMICs; higher bound: 1 (no gains)	Country income group	Uniform	[65,66]

^1^Current eligibility for adults and children is defined in countries’ official UNAIDS AIM files. Some countries have already expanded treatment eligibility according to or beyond the 2013 WHO guidelines, while others do not currently plan to expand treatment eligibility.

^2^WHO 2013 eligibility for adults and children is based on a standardized and universal adoption of the 2013 WHO guidelines in 2014. Under this scenario, the following individuals are eligible for ART: adults and children older than 5 y with a CD4 T cell count of 500 cells/mm^3^ or below, children under 5 y (regardless of CD4 count), pregnant women living with HIV, and those co-infected with HIV and tuberculosis.

^3^CAGR is the compound annual growth rate. This measures the average annual growth rate in coverage increases between an initial and final year.

N/A, not applicable.

### Estimating the Number of People Receiving HIV Treatment

The study estimates the number of people receiving HIV treatment annually in each country based on the numbers of adults and children eligible for treatment and country-specific coverage rates, i.e., the estimated percentage of people eligible for treatment who will receive treatment each year. We define children as people ages 0–14 y and adults as people ages 15 y and older based on previous WHO recommendations for these age groups [2]. However, the early-released WHO 2015 guidelines define children as the age group 0–10 y. The number of people eligible for treatment varies across the three scenarios: current eligibility, WHO 2013, and 90-90-90. The number of adults and children on first- and second-line treatment is based on (1) baseline proportions of people on each line of treatment by country or region from a literature review and (2) annual migration from first- to second-line treatment. Migration rates were derived based on data on resistance to first-line treatment and other trends by country or region. Mortality on second-line treatment was incorporated when carrying over patients from year to year. Both mortality and migration rates were subjected to second-order Monte Carlo simulation, based on the assumptions shown in Table 2. The number of patients on third-line treatment is increasing in low- and lower-middle-income countries, particularly in sub-Saharan Africa. However, these patient populations are still small. As a result, we excluded this group of patients from our analysis.

### Eligibility for Treatment

The study used the uncertainty analysis function in the AIM within the Spectrum software to estimate the median number and 95% confidence interval of people eligible for treatment from 2013 to 2020 across scenarios in each of the 97 countries. The uncertainty analysis randomly selected parameter values for the number of people eligible for treatment for each of the 250 iterations run. For 81 countries, we used the official national Spectrum estimates projection file, obtained from UNAIDS [16]. For 15 countries, we used the Country Data Package in Spectrum and adjusted methods to generate country-specific projections and conduct the uncertainty analysis because of the unavailability of national country AIM files (for a description of these methods, see S1 Text). These ranges from the Spectrum uncertainty analysis were thereafter used in the second-order Monte Carlo simulations, as per Table 2.

As discussed above, we developed three treatment eligibility scenarios for each country. For each scenario, we conducted separate AIM analyses including uncertainty within Spectrum to account for shifts in need for treatment. This resulted in a total of 291 projections across the three scenarios and 97 countries. The first scenario, current eligibility, assumes each country’s eligibility criteria from 2014 to 2020 will be the criteria as set in each country’s official AIM file updated up to 2013 (see S1 Text). In this scenario, CD4 T cell count thresholds for initiating treatment vary by country based on which WHO guidelines countries follow. The second scenario, WHO 2013, assumes that all countries adopt certain aspects of the 2013 WHO guidelines universally beginning in 2014. Universal implementation of these elements of the guidelines would expand the number of treatment-eligible people living with HIV globally, especially since some high-burden countries still apply eligibility thresholds based on the 2010 WHO guidelines and have not made official plans for expanding eligibility in the near term [6,67–69]. The 2013 WHO guidelines recommended expanding eligibility to initiate ART to additional age groups, those co-infected with hepatitis B or tuberculosis, HIV-positive pregnant women, and HIV-positive individuals in a serodiscordant partnership (see Table A in S1 Text) [70]. However, we excluded serodiscordant couples and those co-infected with hepatitis B from our analysis because of a lack of country-specific data and because of the fact that many of the HIV-positive people who are in a serodiscordant relationship are included under the expanded CD4 T cell threshold or as HIV-positive pregnant women. This approach also enabled a focus on analysis of the stronger recommendations for adults, children, pregnant women, and those co-infected with tuberculosis in the 2013 WHO guidelines [2].

The third scenario, 90-90-90, is based on accelerating coverage increases towards the 90-90-90 target and assumes eligibility will expand to all people living with HIV who know their HIV status, as per the recommendations in the early-released 2015 WHO guidelines [3]. To meet the 90-90-90 treatment targets, at least 81% of all people living with HIV will need to be on ART by 2020. We derive this by multiplying 90% by 90%, as 90% of all people living with HIV will be diagnosed with HIV by 2020, and 90% of these people will be on ART. It may also be possible to achieve the 90-90-90 treatment target under the WHO 2013 scenario if countries rapidly scale up treatment coverage in addition to expanding eligibility based on some aspects of the 2013 WHO guidelines. We will compare the number of treatment-eligible people under each scenario to comment on the relationship between global scale-up of ART based on the eligibility thresholds and the 90-90-90 target.

### Annual Coverage Rate for ART

**Fig 1:**
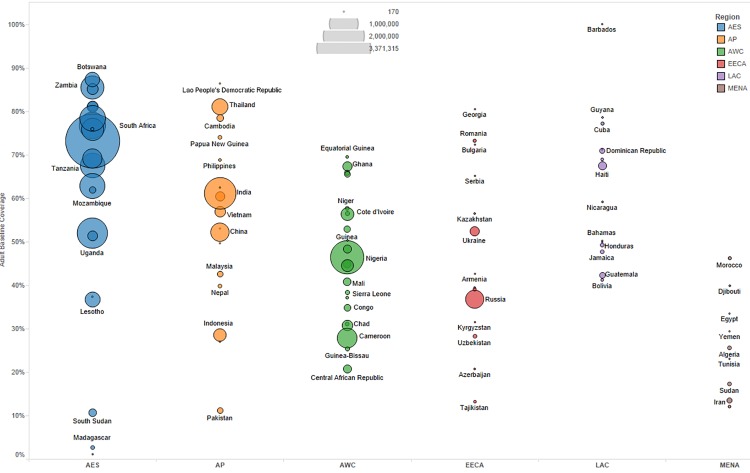
Baseline adult coverage by country under current eligibility scenario. Bubble size represents the number of adults in need of ART in 2013, according to current country eligibility guidelines. The vertical axis shows the percentage of adults eligible for ART who received ART in 2013, which is the baseline coverage rate. The horizontal axis sorts the countries into six regions: Eastern and Southern Africa (AES), Asia and the Pacific (AP), Western and Central Africa (AWC), Eastern Europe and Central Asia (EECA), Latin America and the Caribbean (LAC), and the Middle East and North Africa (MENA). Not all countries included in the analysis are labeled in the figure.

**Fig 2:**
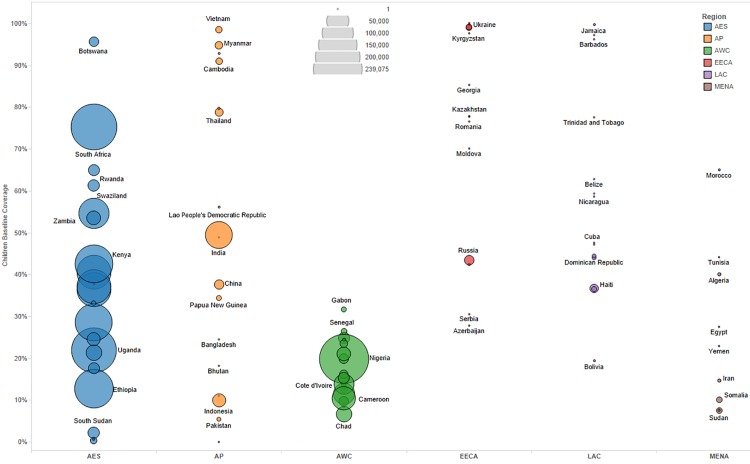
Baseline pediatric coverage by country under current eligibility scenario. Bubble size represents the number of children in need of ART in 2013, according to current country eligibility guidelines. The vertical axis shows the percentage of children eligible for ART who received ART in 2013, which is the baseline coverage rate. The horizontal axis sorts the countries into six regions: Eastern and Southern Africa (AES), Asia and the Pacific (AP), Western and Central Africa (AWC), Eastern Europe and Central Asia (EECA), Latin America and the Caribbean (LAC), and the Middle East and North Africa (MENA). Not all countries included in the analysis are labeled in the figure.

We primarily used data from country-specific AIM analyses and officially reported figures for adults and children living with HIV on ART to establish the baseline coverage of adults and children for ART in 2013 in each of the 97 countries [6,24]. If country-level data for numbers on ART were missing, we used data from the UNAIDS AIDSinfo database or recent Global AIDS Response Progress Reporting [24]. These are data usually submitted by national governments. As of May 2015, data updated to December 2013 or June 2014 were available for most of the 97 countries included in our analysis. The baseline coverage was calculated as a percentage for adults and children by dividing the number of people on treatment at the end of December 2013 by the simulated mean number of people eligible for treatment in December 2013, as determined from the Monte Carlo simulation (Table 2) corresponding to the eligibility scenario. Figs 1 and 2 show the baseline coverage by country and region for adults and children, respectively. (Please see S1 Text for more details on determining coverage.)

Actual country plans to scale up ART vary and are dependent on local interpretation of WHO recommendations, financial resources available, and overarching national HIV strategies. Given the uncertainty in future scale-up paths, we modeled as uncertain the likely value for the annual percentage increase in coverage each year from 2015 to 2020 by country under each of the three scenarios. Based on Monte Carlo simulations, we derived a mean coverage increase based on ranges determined by countries’ and regions’ historical achievement for ART from 2011 to 2013 (see Table 2). If coverage reached 100% of the modeled eligibility in a year, we capped the coverage rate at 100% for the remaining years of analysis.

Our approach for estimating coverage differs from other studies that assume universal coverage rates in a particular future year without considering differing starting points for baseline coverage levels or the factors governing uncertainty in a country’s ability to scale up coverage. For instance, the 2014 study by Stover et al. assumes that 80% of all treatment-eligible people will receive treatment globally in 2015 [8]. By accounting for country-specific starting points and country- or region-specific uncertainty in future treatment scale-up across scenarios, we believe our study presents more rigorous ranges for targets for the total number of adults and children on treatment from 2015 to 2020.

### First- versus Second-Line Treatment

Second-line ART is more resource-intensive than first-line regimens, and the need for such treatment is growing globally, particularly in Africa, as more patients need to switch regimens due to treatment failure [30,34,71]. However, actual migration rates from first- to second-line regimens at the national level are poorly understood, though more data on underlying factors are becoming available [25,30]. Despite the difference in cost and the rising need for second-line regimens, our literature review did not reveal any recent costing studies of ART that account for uncertainty in changes in the number of people receiving second- versus first-line treatment in a global cost analysis.

We used region-specific—and, where available, country-specific—data to model the proportion of adults and children switching to second-line treatment each year. For the baseline split across lines of ART, WHO estimates for adults and children on first- and second-line ART in 2013, country- or region-specific first- versus second-line splits from UNAIDS Global AIDS Response Progress Reporting, and other sources were used to establish the proportions of individuals on first- versus second-line treatment by country [26,27,29,31,32,72]. Annual switching rates were modeled as uncertain rates differentiated at the regional level, with ranges as per Table 2. Ranges by region were set based on literature review of trends in treatment failure, adherence, and clinical management on ART (see S1 Text for a discussion of the trends).

The means from Monte Carlo simulations of annual migration rates from first- to second-line ART were used to estimate the number of patients on first-line ART who would switch to second-line treatment each year and the annual mortality rate among patients on second-line ART, by country. We also used region-specific mortality rates, modeled as an uncertain value (see Table 2), for those on second-line ART to estimate annual mortality rates among the patients on second-line ART carried over year to year.

### Estimating the Costs of HIV Treatment

While there are few modeling analyses estimating the total global costs of ART, several facility-based costing studies, mostly conducted in sub-Saharan Africa, estimate the per-person unit cost of providing ART. In the largest study of its kind, Tagar et al. collected data from a stratified random sample of 161 facilities in five African countries and found that facility-level ART costs ranged from US$136 per patient-year in Malawi to US$682 in South Africa. These costs included drugs, laboratory testing, direct and indirect personnel costs, patient support, equipment, and administrative services [53]. Another study collected HIV treatment costs from 54 clinical sites in six countries and estimated that adult HIV treatment costs per patient per year ranged from US$177 to US$353, depending on the stage of treatment. Patient volume and facility maturity were significant predictors of these facility-level HIV treatment costs [73]. Facility-based studies that estimated unit costs for pediatric HIV treatment found that they are often comparable to adult HIV treatment costs [73]. The average annual cost of providing ART to a child in Zambia was US$209 in 2011, ranging from US$116 to US$516 depending on site [59]. The cost is higher in South Africa: the first-year average cost per child in care and responding to treatment was US$830 at one site and US$678 at another [41]. Duong et al. found that the median costs of non-first-year ART and second-line ART per patient-year in Viet Nam were US$303 and US$1,557 in adults and US$320 and US$1,069 in children, respectively [63]. Other methodologies have also been used to estimate the average unit cost of HIV treatment. Doherty et al. estimated the unit costs of various pediatric HIV treatment regimens based on WHO guidelines by weight band and a ceiling price list provided by the Clinton Health Access Initiative [15]. A systematic review of ART unit costing studies from 2001 to 2009 found that median ART cost per patient-year varied by country income level: median ART costs were US$792, US$932, and US$1,454 for low-income, lower-middle-income, and upper-middle-income countries, respectively [54]. Due to substantial shifts in the pricing of ARVs since this period, these estimates are unsuitable for future cost projections. While ARVs were the largest cost drivers for ART in all settings, analysis of ARV procurement data suggests that the price of ART has declined significantly in more recent years [13,54,74].

This study uses an ingredients-based approach to estimate the unit cost of HIV treatment. We estimated the unit cost of ARVs; laboratory monitoring through CD4 T cell count, viral load, hematology, and clinical chemistry panel tests; and facility-level personnel salaries and overhead expenses, differentiated by country income level. We did not use standard unit costs across all countries because prior studies have shown that costs vary by region and a country’s income level [54,75]. There is also uncertainty in these costs, particularly surrounding future ARV price reductions and middle-income countries’ ability to negotiate and expand access to generic ARVs. We accounted for this uncertainty in determining unit costs for ARVs, laboratory, personnel, and overhead using Monte Carlo simulations based on ranges for current baseline costs and future reductions in costs (ARVs and laboratory only) by country income level (see Table 2). Accounting for projected declines in commodity costs over time was taken into consideration during the peer review process. Cost data were adjusted to constant 2014 US dollars [76].

### Costs of Antiretroviral Drugs

We estimated the current range in adult and pediatric first- and second-line ARV costs by country income level, separating sub-Saharan Africa from other zones, using the Global Price Reporting Mechanism (GPRM) database. This publically available database shows prices that countries have actually paid for ARVs, rather than the price quoted by manufacturers or the cost to the end user [34]. For more information on the GPRM and our dataset, please see S3 Text.

**Fig 3:**
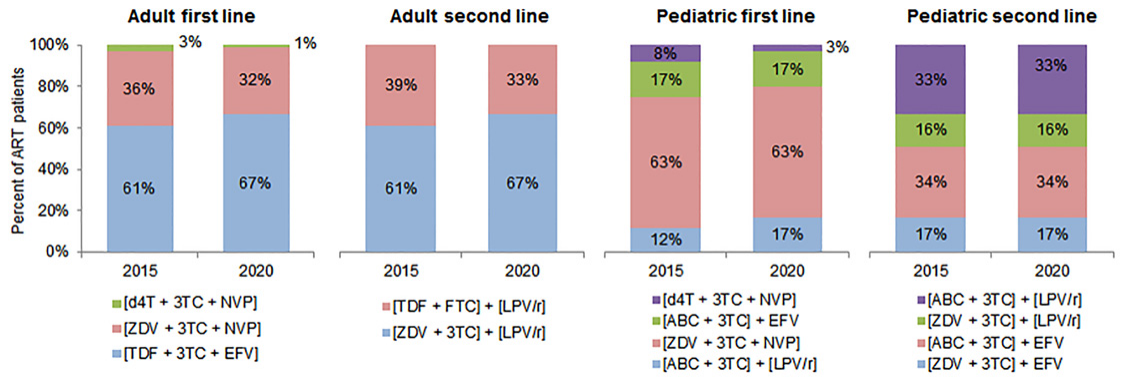
Antiretroviral regimens used in cost calculations. This figure shows the regimens used to calculate the average cost of treatment in each country, along with the percentage of patients on each regimen in 2015 and 2020 among adult and pediatric patients on first- and second-line ART. Regimen distributions for 2016 to 2019 were estimated but are omitted from this figure. Please note: fixed-dose regimens are in square brackets. 3TC, lamivudine; ABC, abacavir; d4T, stavudine; EFV, efavirenz; FTC, emtricitabine; LPV/r, lopinavir/ritonavir; NVP, nevirapine; TDF, tenofovir; ZDV, zidovudine.

The GPRM data query yields the lowest, median, and highest cost per patient-year for select regimens by dosage strength derived from recent country-specific transactions. We sorted these cost results by the World Bank’s income classification scheme (LICs, LMICs, UMICs, and HICs) and zone (sub-Saharan Africa versus all other zones). These ranges were used as parameters for an uncertainty analysis of the current treatment unit costs. We retrieved prices for commonly used and WHO-recommended regimens for adults and children on first- and second-line treatment (see Fig 3 for regimens included in the analysis). The regimens and dosage strengths chosen for analysis are based on WHO recommendations and ARV market analyses [2,26,33]. Transactions before 2011 were excluded. When two or more ARV formulations were used in a particular treatment regimen, their median price based on dosage was summed to estimate the median price of the entire regimen. We used the Clinton Health Access Initiative August 2014 price ceiling list to set the upper limit on ARV costs in LICs. For all HICs except Russia, we assumed ARV costs were equal to those in UMICs [39]. In Russia, we used country-specific pricing data to set the cost parameters because of the very high prices in that country (see Tables B and C in S3 Text) [40].

We assumed that the percentage of adults and children on each regimen would change over time. While stavudine (d4T)–and zidovudine (ZDV)–based regimens are expected to decrease as a proportion of adult formulations, tenofovir (TDF)–based regimens’ market share is expected to increase. For pediatric treatment, d4T-based regimens will further decrease in use, and ZDV-based regimens will continue to represent over half of the market share. Approximately one-third of adult formulations are projected to be nevirapine (NVP)–based, and two-thirds efavirenz (EFV)–based. The majority of pediatric formulations will be NVP-based, with regimens based on lopinavir/ritonavir and EFV comprising the remaining market share [26].

Drug prices have declined significantly from 2000 to 2014, largely because of price reductions in, and expanded access to, generic ARVs [35,36]. Fixed-dose combinations have lowered the cost of ART in recent years and have improved outcomes through improvements in treatment adherence (patients following recommended treatment dosing and frequency) and persistence (length of treatment) [37]. Better procurement policies, improved coordination, partnerships, and increased competition (e.g., competitive pricing of TDF-based fixed-dose combinations that replace d4T-based regimens) have also allowed low- and middle-income countries to provide WHO-recommended regimens at a lower cost and lower patient-level toxicity [37]. For instance, the lowest generic price of the fixed-dose first-line regimen TDF/lamivudine/EFV has declined from US$426 per person per year in 2007 to US$136 per person per year in 2014. The lowest generic price for the protease inhibitor lopinavir/ritonavir, which is commonly used in adult second-line treatment, also declined drastically from 2007 to 2014, from US$1,034 to US$250 per person per year [36]. However, it is unlikely that ARV prices will continue to decrease at this rate, especially for widely used ARVs, including ZDV, lamivudine, and NVP, that are procured in high volumes [33]. There are mixed signals for how ARV pricing in UMICs will change in the future, and ARV pricing is highly sensitive to these countries’ ability to negotiate with generic and original manufacturers and to their compulsory licensing and trade policies. South Africa has been able to negotiate significant price reductions in recent years. However, market analyses suggest that current prices in LICs, particularly for adult first-line treatment, are near the price floor [35,36,38]. UMICs and HICs face the biggest challenge in accessing lower prices [36]. In order to account for uncertainty in future price declines, we used GPRM price trends from 2011 to 2013 and recent ARV market analyses to set ranges for potential annual price declines by country income level [35,36,38]. These ranges were included in the Monte Carlo simulation analysis (see Table 2). In general, we assumed that recent trends in price declines represent the upper bound for future price declines for adult first-line treatment across income groups. For pediatric first- and second-line treatment in middle- and high-income countries, we assumed recent rates of price declines could continue [35].

Using predicted prices, global ARV market projections for the proportion of people on particular regimens, and WHO recommendations for second-line treatment, we simulated a weighted average unit cost of adult and pediatric first- and second-line treatment each year [2,26,33]. Table 3 shows the mean ARV costs per patient per year, by country income level (with sub-Saharan Africa separated). These mean unit costs were reality-checked through comparisons with data from recently published unit costing studies, as well as from the US Agency for International Development–and PEPFAR-funded Health Policy Project’s in-country technical assistance in Ghana, Mozambique, Tanzania, Madagascar, Ukraine, and Kenya [42,43,53].

**Table 3:** Simulated mean antiretroviral unit costs per person-year by country income group and zone.

Country Income Group	Adult				Pediatric			
	First-Line ART		Second-Line ART		First-Line ART		Second-Line ART	
	2015	2020	2015	2020	2015	2020	2015	2020
LICs	$106 (95, 114)	$97 (83, 108)	$277 (236, 314)	$176 (138, 217)	$109 (99, 120)	$86 (67, 105)	$174 (153, 198)	$114 (90, 143)
LMICs (Africa)	$114 (103, 123)	$104 (90, 117)	$317 (287, 350)	$293 (254, 332)	$111 (103, 119)	$84 (65, 102)	$173 (158, 185)	$105 (86, 128)
LMICs (other)	$117 (110, 129)	$107 (95, 121)	$405 (297, 555)	$374 (269, 522)	$145 (129, 160)	$110 (84, 136)	$234 (209, 255)	$141 (114, 173)
UMICs (Africa)	$119 (116, 122)	$113 (101, 121)	$398 (324, 466)	$250 (198, 301)	$100 (94, 107)	$89 (77, 100)	$144 (137, 153)	$122 (103, 138)
UMICs (other)	$189 (120, 337)	$185 (113, 336)	$608 (502, 680)	$375 (303, 436)	$123 (115, 135)	$109 (95, 126)	$214 (194, 241)	$182 (153, 214)
HICs^1^	$1,510 (683, 2,540)	$1,326 (568, 2,302)	$1,891 (1,109, 2,506)	$1,224 (690, 1,677)	$1,882 (997, 2,818)	$1,511 (720, 2,378)	$1,901 (1,332, 2,555)	$1,073 (625, 1,650)

Data are mean (95% CI) unit cost in 2014 US dollars. Unit costs were estimated for all intervening years and are not shown here.

^1^We estimated ARV costs in all HICs except Russia using the UMIC mean ARV costs. The HIC costs given here were used only for Russia, where ARV pricing is known to be much higher than in other HICs in our sample [40].

### Laboratory Costs

We modeled the unit cost of laboratory tests as uncertain based on recent literature and in-country analyses on the reagent and commodity costs of monitoring viral load and CD4 cell count and conducting hematology and clinical chemistry panel tests for ART patients. Cost data collected prior to 2011 were excluded from the analysis. The data used are from 12 countries (primarily in sub-Saharan Africa) and were collected by the Clinton Health Access Initiative, the Health Policy Project, and Médecins Sans Frontières; we compared these data to results in older studies [28,42–46,53]. Costs per test were categorized by country income level and zone, and laboratory costs per patient per year were based on frequency assumptions. We assumed that every ART patient in LICs and LMICs would receive one viral load, CD4 T cell count, hematology, and clinical chemistry panel test per year. For UMICs and HICs, we assumed patients would receive two hematology and clinical chemistry panel tests per year because of improved patient monitoring conditions in those settings. The 2013 WHO guidelines recommend two CD4 T cell count tests per ART patient per year, but the average number of CD4 tests per patient per year in sub-Saharan Africa is currently less than two, and the need for frequent CD4 T cell count testing for patient monitoring could be scaled back as countries move to routine viral load testing [47,53]. Although the 2013 WHO guidelines recommend routine use of virological testing to detect early signs of treatment failure, many countries do not conduct routine viral load testing because of the still high costs per test and the need for specialized laboratory equipment [48,53]. Instead, immunological tests are performed to detect the level of CD4 T cells. As a result, we ran an additional scenario that assumes targeted viral load testing, i.e., viral load tests being conducted only among those with suspected virological failure. Under this targeted viral load testing scenario, 5% of ART patients in LICs and LMICs and 10% of patients in UMICs and HICs would receive a viral load test in a year, and all patients would receive two CD4 count tests per year.

The lower bound for current costs of viral load testing in LICs and LMICs is Roche Diagnostics’s recently announced global access price of US$9.40 [49]. We used the average cost within country income groups from the data sources discussed above as the most likely cost, which was significantly higher than US$9.40. The upper limit for uncertainty analysis was the highest reported price within a country income group. The highest effective cost per viral load test, particularly in LICs (shown in Table 4), is likely a result of the low batch volume of the viral load assays run, rather than resulting from higher prices of reagents and supplies [45]. Because there were limited data for UMICs and HICs, we used a large range (US$10–US$60) for uncertainty analysis of the cost of viral load testing in these country income groups. For all other tests, we generally used the lowest, average, and highest prices within each country income group classification to determine the uncertainty in baseline costs of laboratory tests.

**Table 4:** Simulated mean laboratory and overhead and personnel unit costs per person-year.

Country Income Group	Baseline Mean Cost per Test (2014)			Annual Mean Percent Decline in Cost		Mean Laboratory Cost per Person per Year (95% CI)		Mean Overhead and Personnel Cost per Person per Year^1^ (95% CI)
	Viral Load	CD4	Hematology and Clinical Chemistry Panel	Viral Load	CD4	2015	2020	
LICs	$38.6	$5.4	$3.9	3.6%	1.3%	$35.9 (23.3, 49.1)	$26.9 (16.7, 40.5)	$72 (56.1, 89.8)
LMICs (Africa)	$21.9	$6.2	$3.9	8.0%	1.8%	$32.6 (23.1, 41.7)	$24.5 (15.8, 35.2)	$72 (57.7, 88.7)
LMICs (other)	$24.7	$12.1	$4.0	5.9%	7.6%	$36.2 (25.4, 45.9)	$27.1 (17.6, 38.3)	$72 (57.7, 88.7)
UMICs (Africa)	$34.1	$8.6	$4.8	10.7%	3.9%	$44.9 (30, 63.3)	$35.3 (23.3, 51.9)	$328 (167.1, 477.5)
UMICs (other)	$44.5	$7.5	$3.5	7.0%	2.8%	$52.3 (34.4, 69.8)	$40.5 (26, 59)	$328 (167.1, 477.5)
HICs	$52.9	$8.9	$4.5	14.5%	5.3%	$52.4 (34.9, 69.5)	$40.5 (26.3, 58.8)	$328 (167.1, 477.5)

^1^Overhead and personnel costs here reflect the application of the efficiency factor to the raw estimates from the literature.

Similar to ARVs, viral load and CD4 testing costs have declined over the last decade, by as much as 80%, though it is uncertain whether and to what extent prices may decline further [50]. The cost of HIV diagnosis and monitoring could be lowered through increased use of new technologies, including point-of-care testing, greater competition and better coordination in procurement, and increased testing volumes [51,52]. Once available, a single-use disposable CD4 test could be priced as low as US$2–US$3 per test [48]. Given the uncertainty in future CD4 test costs, we assumed the range in cost declines from 2015 to 2020 to be 0% to 16%. Because of wide ranges in current viral load test costs and countries’ ability to increase viral load testing volumes and efficiency, we assumed a wide range for annual cost declines (0% to 20%) for viral load testing [45]. The costs of hematology and clinical chemistry panel tests are likely to stay relatively constant in real terms, so we did not develop uncertainty parameters for changes in these costs.

Table 4 shows the mean weighted unit cost for all laboratory tests by country income level and zone. These costs are in line with UNITAID estimates. According to UNITAID, the current cost of CD4 reagents and consumables ranges from US$3 to US$16 per test, depending on testing volumes and reagents used. In relation, costs for viral load testing are currently significantly higher, at about US$28 to US$29 per test, though higher unit costs have been reported for LICs, and lower costs elsewhere [43–45]. One component of the cost of hematology tests is the basic full blood count; the average cost of consumables for this test is about US$3.15 per test. The cost of a limited blood chemistry panel test, with consumables, ranges from US$1.60 to US$1.95 per test [48].

### Facility-Level Overhead and Personnel Costs

We estimated ranges for the unit cost per patient-year of facility-level overhead (e.g., for utilities) and personnel costs (e.g., for clinical health workers who directly deliver treatment interventions) based on a review of recent country-specific studies [28,53–55,57, 58,60–63]. The estimates of unit costs of facility-level overhead differ across studies because of the inclusion of different cost elements. We focused on studies where we could separate the unit costs of equipment, utilities, supplies, and other recurrent facility-level costs. For the types of costs included under the rubric of “overhead,” see Table A in S3 Text. The original data analysis plan estimated facility-level personnel and overhead costs as a proportion of the cost of ARVs and laboratory reagents; however, based on comments from peer reviewers, we decided to use stable dollar value estimates of personnel and overhead costs per patient per year.

The median unit cost of overhead and personnel, differentiated by the LIC, LMIC, and UMIC/HIC groups, was determined as the mode for Monte Carlo uncertainty analysis, with the lowest and highest unit costs within each group representing lower and upper bounds (see Table 2). A total of 23 and 19 data points were used to estimate personnel and overhead costs, respectively, eliminating extreme outliers. While we lack a methodology to rigorously model how overhead and personnel costs could change over time, we applied an uncertain, one-time modest efficiency gain factor to the 2014 estimate from the literature that ranges from 0.5 to 1, where 1 represents no efficiency gain (i.e., unit costs of personnel and overhead per patient-year do not decrease with increased patient volume). We set the bounds for simulating the efficiency gain factor by country income group, based on a prior study and recent evidence for efficiency improvement (see Table 2) [65,66]. For all but UMICs and HICs, the one-time efficiency reduction in the unit cost was 8% or less. For UMIC/HIC overhead costs, which at baseline were quite large, a larger reduction was taken. The unit value per patient-year for personnel and overhead costs is shown by country income level in Table 4.

### Estimating Financial Resources Available for ART

We estimated the financial resources available for ART based on country-specific funding trends from external and domestic sources. For external funding, we analyzed contributions from PEPFAR and the Global Fund. We also excluded any multi-country regional support from PEPFAR or the Global Fund where the funding values could not be disaggregated by country. Support for ART for adults and children from PEPFAR extended to 39 countries and regional programs in fiscal year 2014 [55]. This support included ARV drugs and laboratory commodities for many of these countries, as well as health system strengthening and temporary staff. The Global Fund has 252 active HIV-related grants and has dispensed US$14 billion for HIV prevention, treatment, and care since 2002. Approximately 40% of Global Fund grants support commodity procurement [13].

We used the Price and Quality Reporting Tool, a database with transaction-level procurement information, to estimate Global Fund contributions to ARV and laboratory commodity procurement (CD4 T cell count and viral load tests only) from 2011 to 2014 in countries eligible for Global Fund funding as of 2014 [77]. We also reviewed country allocations under the Global Fund’s new funding model (NFM) for the period 2014 to 2016 to reality-check the estimated contributions for commodities against a notional ceiling imposed by the allocation amount, which is for the entire national HIV response [78]. Many recent NFM grant applications have been focused on commodities. Global Fund disbursements can vary by year over the grant period, and grant-funded procurements may span needs across calendar years, so we calculated the recent average financial resources available for ART commodities per year unless these values were specified in the country-specific NFM grant documents we had access to. We assumed that annual Global Fund contributions as a dollar amount would remain constant from 2015 to 2020, unless specific country-level details were available on future Global Fund funding for ART, by year. For the 32 of our 97 countries that PEPFAR supports, PEPFAR resources for the cost categories included in our financial resource projections—ARVs, laboratory commodities, and facility-level overhead and personnel—were based on 2014 expenditure data from the interactive PEPFAR Dashboards database. This database includes planned budget and expenditure data from PEPFAR Country Operational Plans (see S4 Text) [55]. We estimated two scenarios: expansive versus conservative PEPFAR contribution to facility-level personnel and overhead in each country. These scenarios were needed to capture uncertainty related to the types of site-level overhead costs included in PEPFAR funding for facility-level care and treatment, and because of unclear information on the types of costs included in certain unit cost studies we reviewed on facility-level overhead costs. While the conservative contribution scenario included PEPFAR cost categories that are most aligned with the cost categories used to estimate facility-level overhead resource requirements, the expansive contribution scenario included additional PEPFAR cost categories that may include some funding for our definition of facility-level overhead. We assumed future funding levels from PEPFAR would remain constant each year at our estimated contribution levels. By keeping PEPFAR and Global Fund contributions constant as a dollar figure, we assumed that external funding for ART as a percentage of the total resources needed was decreasing over time. However, recent information on PEPFAR priorities suggests that its funding for ART and care for people living with HIV will maintain or increase its share of total PEPFAR resources [79].

Domestic resources available for ART were based on various sources [6,11]. We used country-reported contributions to ART commodity procurement for 15 countries that had this information publicly available or cited in the media (see S4 Text for specific analyses conducted for India and all sources of related data across countries). In the remaining countries, we estimated domestic contributions (DCs) to commodity procurement in countries eligible for Global Fund financing using the Global Fund’s counterpart financing thresholds (CFT), which require a minimum level of matching government contribution as a share of Global Fund financing for commodities, distinct by country income status and HIV disease burden. In applying these CFT proportions to future Global Fund support levels, we are making an ambitious assumption that countries are willing and able to contribute to ARV and laboratory commodity procurement at increasing levels. The Global Fund does not require the CFT to be based on commodity procurement, and countries can contribute their financial resources in different areas aligned to the national response. For countries not eligible for Global Fund financing, we estimated the proportional potential DC based on countries’ reported contributions to their overall HIV responses [24,40,80–96]. We multiplied the total ARV and laboratory resource need by this DC percentage to estimate such countries’ DCs to commodity procurement. DC toward the cost of facility-based personnel and overhead was estimated separately by multiplying the country-specific estimated total cost of facility-based overhead and personnel each year by the DC percentage. We assumed that the country-specific percentage contributions from CFT or DC, as applicable, to commodity procurement and facility-level costs would remain constant from 2015 to 2020 unless country-specific data suggested otherwise. As resource requirements increase over time, this implies DCs will increase. Overall, our methods imply an optimistic and large DC to ART commodities, beyond the traditional areas of health workforce and facility-level overhead that many country governments have funded. (See S4 Text for a description of the CFT levels and the sources for DC estimates.)

**Fig 4:**
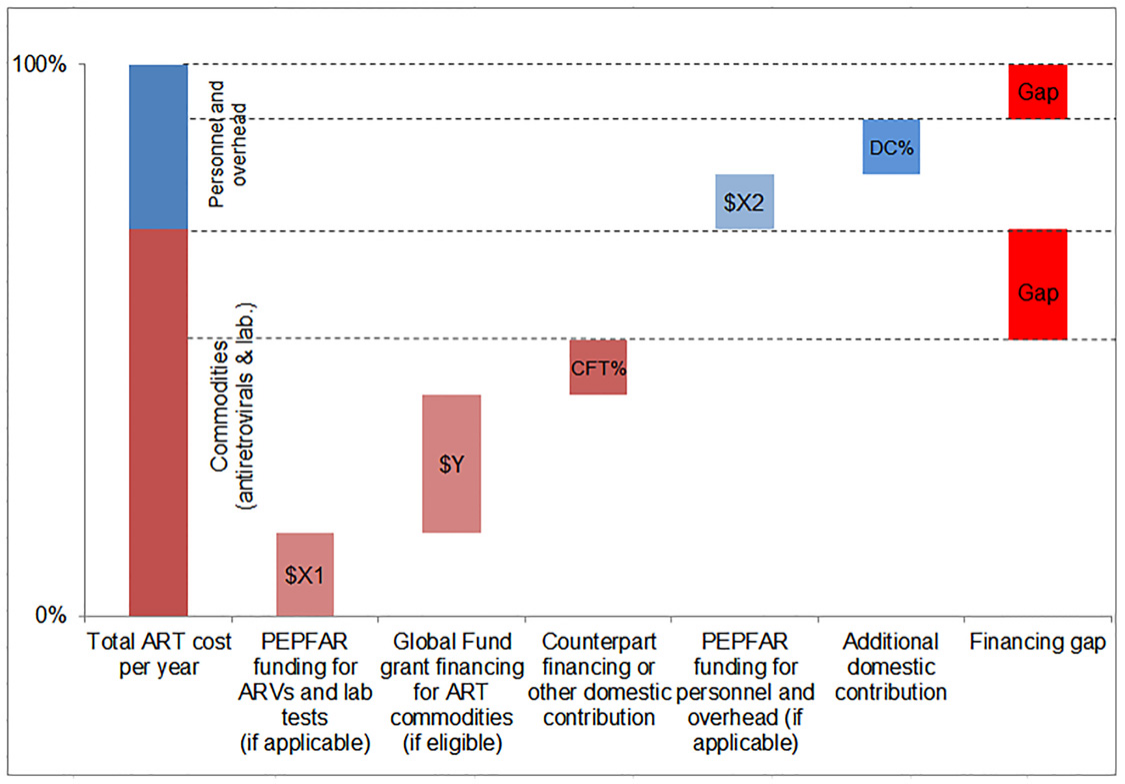
Estimating the funding gap. We separately calculated the funding gap for ARVs and laboratory commodities versus that for facility-level overhead and personnel. For each country, we considered Global Fund contributions to commodity procurement, as well as PEPFAR and DCs to commodity procurement and overhead and personnel, as applicable. DCs were estimated using Global Fund CFTs in eligible countries or country-reported proportional contributions to the HIV response.

We compared the simulated mean financial resources required by country for each of our three scenarios to resources available for ART to estimate the potential funding gap range. We estimated the commodity funding gap by subtracting Global Fund, PEPFAR, and domestic (CFT or other) contributions from the total cost of ARVs and laboratory commodities under each eligibility scenario, by year (Fig 4). We then estimated the funding gap for personnel and overhead costs by subtracting PEPFAR funding, where applicable, and DCs from the total cost of personnel and overhead under each scenario by year. The range in total funding gap for ART was estimated to be the commodity gap plus the gap for personnel and overhead for each scenario. See Fig 4 for a graphic representation of the methodology used to estimate the funding gap.

## Results

### Eligibility for ART

Under the current eligibility scenario and with the forecast increase in coverage annually, we estimate that the number of people eligible for treatment will increase from 21 (95% CI 20.6, 21.4) million adults living with HIV and 1.74 (95% CI 1.72, 1.77) million children living with HIV in 2015 to 27.9 (95% CI 27.4, 28.4) million adults and 1.93 (95% CI 1.89, 1.96) million children in 2020. Universal adoption of select aspects of the 2013 WHO guidelines in 2014 further expands the number of people living with HIV eligible for treatment to 24.5 (95% CI 24.0, 25.0) million adults and 1.66 (95% CI 1.63, 1.70) million children in 2015 and 31.3 (95% CI 30.7, 32.0) million adults and 1.90 (95% CI 1.85, 1.95) million children in 2020. There are more children eligible for treatment under the WHO 2013 scenario than the current scenario due to some countries having pediatric eligibility criteria that exceed the 2013 WHO guidelines and the fact that need for pediatric ART declines as the future incidence among this age group decreases with increases in ART coverage among mothers. Our 90-90-90 scenario assumes that countries will adopt the WHO 2015 guidelines and that all people living with HIV who know their status will be eligible for treatment. In this case, we estimate 35.3 (95% CI 34.7, 35.9) million adults and 2.54 (95% CI 2.49, 2.58) million children will be eligible for ART in 2020. By dividing the simulated mean number of people eligible for treatment under the current eligibility and WHO 2013 scenarios by the mean number of people eligible for treatment under the 90-90-90 scenario (i.e., mean number of people living with HIV), we are able to estimate whether or not the 90-90-90 targets can be met under certain eligibility criteria. Our results indicate that 79% of all people living with HIV could be eligible for treatment in 2020 if current eligibility criteria were maintained. In comparison, those eligible for treatment under the WHO 2013 scenario may represent 88% of all people living with HIV in 2020. These results suggest that the 90-90-90 target of 81% of all people living with HIV on treatment by 2020 would not be reached under the current eligibility scenario.

### Numbers of Patients on ART

**Fig 5:**
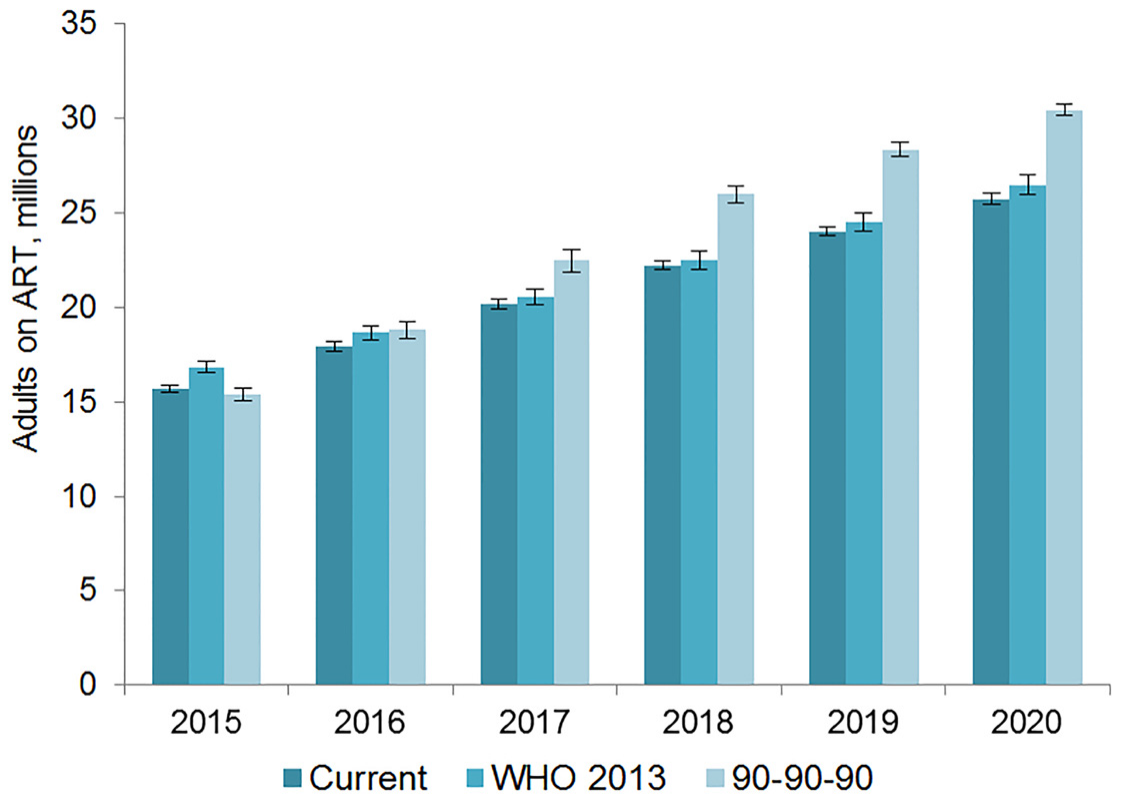
Estimated range of adults living with HIV on ART. The vertical axis shows the number of adults, in millions, who are estimated to be on ART each year, while the horizontal axis shows years. Each color represents a different scenario, and the whiskers on each bar represent the lower and upper bound of the 95% confidence interval.

**Fig 6:**
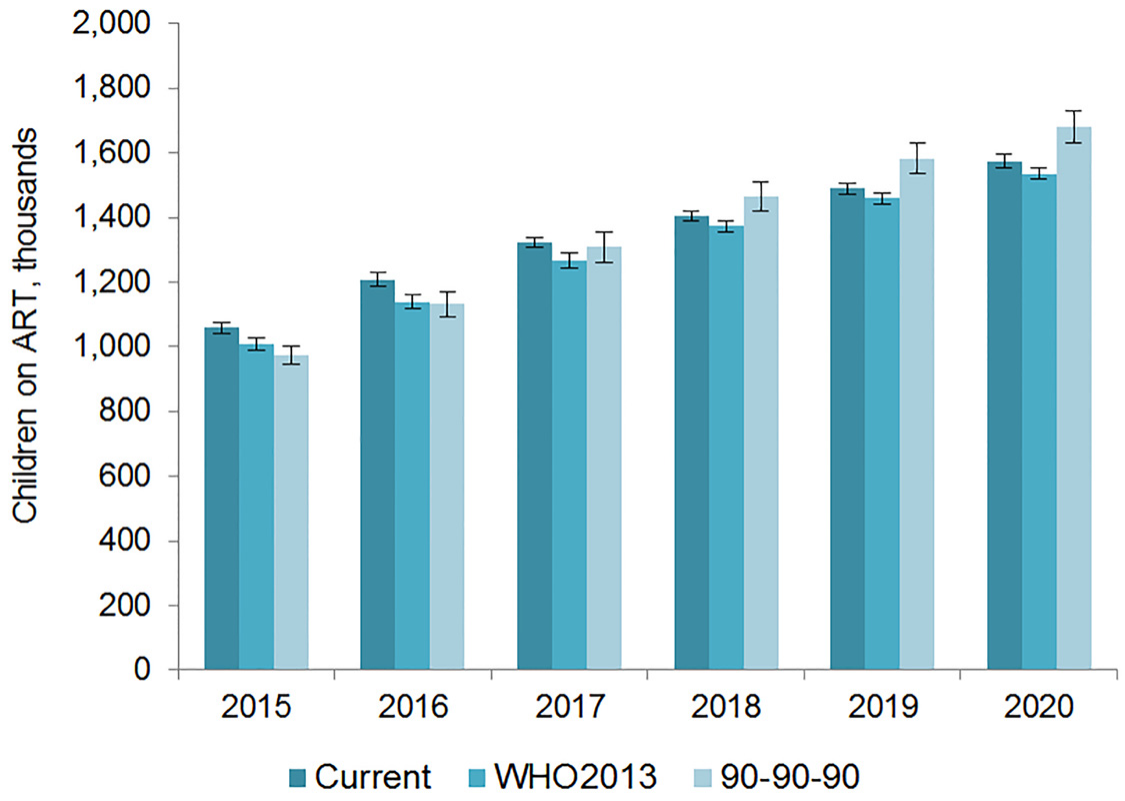
Estimated range of children living with HIV on ART. The vertical axis shows the number of children, in thousands, who are estimated to be on ART each year, while the horizontal axis shows years. Each color represents a different scenario, and the whiskers on each bar represent the lower and upper bound of the 95% confidence interval.

The number of adults and children who may be on ART each year is dependent on the mean number of treatment-eligible people and on the annual scale-up of coverage. Under the current eligibility scenario, given coverage increases based on historical rates, we estimate the numbers on treatment to grow to 25.7 (95% CI 25.5, 26.0) million adults and 1.57 (95% CI 1.55, 1.60) million children in 2020, meaning 92% (95% CI 91%, 94%) of adults and 82% (95% CI 80%, 83%) of children eligible for treatment would be on ART (Table 5). Our results indicate that coverage using all people living with HIV as the denominator would be 72% in 2020 under this scenario, which is short of the 81% target under 90-90-90. By 2020, 26.5 (95% CI 26.0, 27.0) million adults and 1.53 (95% CI 1.52, 1.55) million children could receive ART if countries universally adopt select elements from the 2013 WHO eligibility recommendations and scale up coverage (see Figs 5 and 6). Under this scenario, 85% (95% CI 82%, 86%) of adults and 81% (95% CI 79%, 83%) of children eligible for treatment would be on ART in 2020. Our results suggest that 90-90-90 targets may not be met under this scenario, as we estimate only 74% of people living with HIV would be on treatment in 2020. Under the 90-90-90 scenario, we forecast 86% (95% CI 85%, 88%) of adults living with HIV and 66% (95% CI 65%, 68%) of children living with HIV would be on treatment by December 2020, based on 30.4 (95% CI 30.1, 30.7) million adults living with HIV and 1.68 (95% CI 1.63, 1.73) million children living with HIV on ART.

**Table 5:** Number on treatment and commodity costs by eligibility scenario and country.

Country	Current Scenario			WHO 2013 Scenario			90-90-90 Scenario		
	Total on Treatment in 2020 (in Thousands)		2015–2020 Total Commodity Cost (in Millions of US Dollars)	Total on Treatment in 2020 (in Thousands)		2015–2020 Total Commodity Cost (in Millions of US Dollars)	Total on Treatment in 2020 (in Thousands)		2015–2020 Total Commodity Cost (in Millions of US Dollars)
	Adults	Children		Adults	Children		Adults	Children	
**Eastern and Southern Africa**									
Angola	210.4 (201.3, 220.6)	27.4 (22.6, 30.2)	$156.8 (150.9, 163.2)	223.1 (192.2, 254.1)	27.4 (24.4, 30.4)	$179.8 (156.9, 202.7)	343.6 (285.3, 365.5)	36.3 (27.9, 44.7)	$219.0 (182.4, 255.2)
Botswana	320.9 (320.7, 321.1)	8.8 (8.8, 8.8)	$342.7 (334.5, 351.1)	319.7 (319.4, 320.0)	8.8 (8.8, 8.8)	$341.7 (333.5, 349.9)	345.5 (345.4, 345.7)	9.2 (8.9, 9.4)	$372.7 (363.8, 381.8)
Burundi	52.6 (48.9, 56.7)	6.8 (5.4, 7.6)	$41.1 (39.0, 43.3)	48.8 (46.0, 51.7)	6.6 (5.6, 7.6)	$40.0 (38.2, 41.8)	60.1 (59.7, 60.2)	6.4 (4.8, 8.3)	$50.1 (48.2, 51.7)
Comoros	0.7 (0.6, 0.7)	0.1 (0.1, 0.1)	$0.3 (0.3, 0.4)	1.1 (1.0, 1.2)	0.1 (0.1, 0.1)	$0.6 (0.6, 0.7)	0.8 (0.7, 0.9)	0.2 (0.1, 0.2)	$0.4 (0.3, 0.4)
Eritrea	14.8 (14.7, 14.8)	0.8 (0.8, 0.8)	$12.2 (11.8, 12.5)	13.9 (11.2, 15.0)	0.8 (0.8, 0.8)	$11.5 (9.2, 13.0)	15.2 (15.1, 15.3)	1.4 (1.2, 1.6)	$13.3 (12.5, 13.9)
Ethiopia	661.5 (635.8, 664.9)	44.8 (39.1, 50.7)	$501.7 (478.1, 522.2)	575.8 (525.5, 629.9)	45.4 (42.3, 46.0)	$443.6 (409.4, 478.9)	688.6 (687.0, 689.2)	31.4 (27.9, 35.4)	$535.5 (512.3, 554.6)
Kenya	1,569.6 (1,566.4, 1,570.9)	138.9 (138.7, 139.7)	$1,379.8 (1,327.9, 1,427.2)	1,364.0 (1,290.1, 1,421.8)	122.2 (122.1, 122.7)	$1,161.3 (1,110.0, 1,213.4)	1,727.8 (1,726.0, 1,729.4)	155.3 (144.6, 166.8)	$1,366.1 (1,295.2, 1,423.6)
Lesotho	202.8 (186.5, 219.0)	8.5 (8.1, 8.5)	$148.6 (139.7, 157.6)	174.6 (163.0, 186.9)	10.4 (9.3, 10.9)	$136.3 (128.4, 144.7)	401.5 (376.4, 405.1)	11.0 (9.2, 13.1)	$238.4 (220.6, 253.4)
Madagascar	1.3 (1.2, 1.4)	0.04 (0.03, 0.05)	$1.0 (1.0, 1.1)	1.0 (0.8, 1.2)	0.04 (0.03, 0.1)	$0.8 (0.7, 1.0)	1.8 (1.5, 2.1)	0.04 (0.03, 0.1)	$1.0 (0.9, 1.2)
Malawi	869.9 (869.4, 870.6)	84.2 (84.2, 84.3)	$729.1 (706.5, 749.0)	858.1 (802.9, 882.1)	86.2 (86.2, 86.6)	$696.1 (657.6, 729.2)	943.4 (941.9, 944.2)	117.7 (110.9, 124.8)	$806.0 (790.6, 821.5)
Mauritius	4.2 (3.9, 4.5)	0.1 (0.1, 0.1)	$3.7 (3.5, 3.9)	3.7 (3.3, 4.0)	0.1 (0.1, 0.1)	$3.5 (3.2, 3.7)	7.2 (6.2, 7.9)	0.1 (0.1, 0.1)	$4.7 (4.1, 5.1)
Mozambique	1,321.1 (1,259.5, 1,332.7)	141.9 (141.8, 142.0)	$902.6 (859.1, 937.1)	1,244.3 (1,154.6, 1,338.2)	147.3 (146.7, 147.4)	$903.7 (853.2, 957.5)	1,786.9 (1,777.1, 1,789.9)	159.7 (130.9, 189.2)	$1,126.9 (1,054.6, 1,188.9)
Namibia	299.1 (298.5, 301.2)	15.7 (15.7, 15.7)	$302.8 (294.8, 310.5)	276.1 (274.4, 276.7)	12.8 (12.8, 12.8)	$269.7 (260.7, 278.1)	301.5 (301.0, 304.1)	16.6 (15.1, 18.4)	$290.3 (276.9, 302.7)
Rwanda	202.9 (201.8, 203.2)	9.0 (9.0, 9.0)	$168.9 (164.7, 172.7)	196.1 (170.6, 201.0)	9.2 (9.2, 9.2)	$160.3 (134.5, 171.1)	207.4 (207.2, 207.9)	12.6 (11.9, 13.3)	$178.3 (174.1, 182.3)
South Africa	5,465.7 (5,464.6, 5,468.6)	190.7 (190.7, 191.1)	$4,984.2 (4,836.1, 5,122.2)	6,420.7 (6,070.8, 6,707.2)	202.8 (202.8, 203.1)	$5,726.2 (5,469.1, 5,980.7)	7,361.8 (7,355.4, 7,366.2)	253.4 (241.8, 264.6)	$6,369.0 (6,103.4, 6,592.6)
South Sudan	17.5 (16.1, 18.9)	1.2 (0.7, 2.1)	$13.9 (13.0, 14.9)	15.7 (8.2, 30.2)	1.3 (0.6, 2.6)	$13.3 (7.3, 24.8)	27.8 (16.5, 45.7)	1.2 (0.6, 2.4)	$16.8 (10.4, 27.0)
Swaziland	184.9 (184.9, 185.0)	12.6 (12.6, 12.6)	$165.9 (160.8, 170.9)	187.2 (183.0, 188.0)	12.7 (12.6, 12.7)	$159.9 (153.4, 165.9)	218.5 (218.3, 218.8)	15.6 (15.2, 16.1)	$188.4 (182.8, 193.9)
Tanzania	1,102.5 (1,054.9, 1,110.6)	116.5 (116.4, 116.7)	$863.2 (824.5, 895.6)	1,208.1 (1,103.5, 1,271.0)	71.2 (71.2, 71.5)	$918.9 (844.1, 987.4)	1,377.0 (1,370.5, 1,379.5)	79.8 (66.1, 95.7)	$976.0 (876.7, 1,044.9)
Uganda	1,485.6 (1,380.7, 1,597.6)	128.4 (113.3, 143.5)	$1,040.5 (988.6, 1,095.6)	1,559.3 (1,457.6, 1,663.2)	86.9 (86.8, 87.0)	$1,103.4 (1,046.0, 1,161.7)	1,916.6 (1,914.2, 1,917.9)	133.3 (110.1, 157.9)	$1,313.7 (1,254.0, 1,361.6)
Zambia	859.6 (859.1, 860.2)	75.7 (75.6, 76.1)	$828.9 (805.3, 852.7)	856.9 (855.8, 857.6)	77.4 (77.3, 77.5)	$830.7 (807.0, 854.8)	999.0 (998.3, 1,000.3)	107.0 (100.8, 113.1)	$960.1 (932.6, 987.4)
Zimbabwe	1,567.6 (1,565.2, 1,569.2)	75.1 (75.0, 75.2)	$1,284.1 (1,262.5, 1,305.8)	1,464.1 (1,463.8, 1,467.8)	74.8 (74.7, 75.2)	$1,159.9 (1,134.0, 1,185.8)	1,585.9 (1,584.3, 1,587.2)	102.5 (93.3, 109.0)	$1,239.0 (1,214.3, 1,263.9)
**Asia and the Pacific**									
Bangladesh	5.1 (4.8, 5.4)	0.3 (0.3, 0.3)	$2.6 (2.4, 2.7)	5.7 (5.5, 6.0)	0.3 (0.3, 0.3)	$3.2 (3.1, 3.4)	5.4 (5.2, 5.7)	0.3 (0.3, 0.4)	$2.8 (2.7, 2.9)
Bhutan	0.6 (0.6, 0.6)	0.03 (0.02, 0.1)	$0.4 (0.4, 0.4)	1.3 (0.9, 1.6)	0.05 (0.02, 0.1)	$0.7 (0.5, 1.0)	0.9 (0.5, 1.3)	0.03 (0.01, 0.1)	$0.5 (0.3, 0.7)
Cambodia	67.8 (67.5, 68.0)	3.6 (3.6, 3.6)	$60.3 (58.7, 61.7)	62.5 (49.4, 67.2)	3.7 (3.7, 3.8)	$54.1 (41.8, 65.1)	74.4 (74.1, 75.3)	4.4 (2.8, 6.0)	$64.4 (54.5, 72.1)
China	794.1 (794.0, 795.2)	11.9 (11.2, 12.6)	$810.2 (779.9, 835.6)	861.2 (807.2, 915.5)	14.7 (13.8, 15.5)	$897.5 (852.6, 943.5)	832.3 (729.1, 941.1)	12.4 (11.2, 13.8)	$785.4 (713.2, 860.6)
India	1,946.7 (1,945.1, 1,950.2)	68.6 (68.6, 69.0)	$1,525.2 (1,491.5, 1,561.1)	2,399.0 (2,316.9, 2,409.6)	97.6 (96.1, 97.9)	$1,794.7 (1,654.0, 1,923.6)	2,702.6 (2,595.0, 2,720.7)	72.3 (61.3, 84.8)	$1,712.4 (1,589.8, 1,840.4)
Indonesia	217.8 (198.5, 236.5)	5.0 (4.0, 6.3)	$137.2 (127.8, 145.8)	268.7 (227.5, 314.1)	5.7 (4.3, 7.6)	$185.6 (158.9, 214.6)	248.0 (193.7, 314.9)	6.9 (4.9, 9.5)	$147.6 (118.9, 183.9)
Lao People’s Democratic Republic	5.1 (5.0, 5.1)	0.4 (0.4, 0.4)	$4.5 (4.4, 4.6)	7.9 (7.4, 8.0)	0.7 (0.6, 0.8)	$6.4 (5.5, 6.9)	8.6 (7.9, 8.7)	0.6 (0.4, 0.9)	$6.3 (5.1, 7.2)
Malaysia	72.0 (66.0, 74.5)	0.7 (0.7, 0.7)	$68.0 (63.6, 71.6)	63.0 (39.7, 84.0)	1.2 (1.2, 1.2)	$70.2 (45.8, 91.9)	65.4 (49.6, 82.2)	1.0 (0.8, 1.2)	$64.1 (50.1, 78.8)
Mongolia	1.0 (1.0, 1.0)	0.001 (0.001, 0.001)	$0.6 (0.6, 0.6)	0.9 (0.7, 1.0)	0.002 (0.002, 0.002)	$0.5 (0.4, 0.6)	0.5 (0.4, 0.7)	0.002 (0.001, 0.003)	$0.3 (0.2, 0.4)
Myanmar	140.9 (140.8, 141.0)	10.7 (10.7, 10.7)	$106.2 (103.6, 108.2)	156.3 (149.2, 157.8)	13.4 (13.4, 13.4)	$113.9 (109.0, 118.1)	160.1 (150.8, 161.1)	15.0 (14.7, 15.2)	$107.2 (99.6, 113.9)
Nepal	38.9 (38.8, 39.0)	1.4 (1.4, 1.4)	$23.0 (22.6, 23.4)	24.9 (21.5, 28.4)	1.1 (1.1, 1.1)	$16.3 (14.2, 18.4)	25.3 (20.6, 30.8)	1.8 (1.6, 1.9)	$15.4 (13.0, 18.2)
Pakistan	43.0 (39.1, 46.7)	0.6 (0.5, 0.9)	$19.9 (18.5, 21.3)	36.9 (24.8, 53.8)	0.7 (0.5, 1.1)	$18.2 (12.4, 26.2)	60.2 (43.5, 82.3)	0.8 (0.4, 1.3)	$26.0 (19.1, 35.2)
Papua New Guinea	30.7 (30.7, 31.0)	2.7 (2.4, 2.8)	$27.2 (26.6, 27.9)	36.6 (36.3, 36.6)	3.5 (3.1, 3.9)	$31.8 (31.1, 32.6)	39.8 (39.3, 39.9)	3.5 (2.8, 4.5)	$32.0 (30.0, 33.8)
Philippines	26.3 (26.3, 26.4)	0.1 (0.1, 0.2)	$18.0 (17.6, 18.3)	31.7 (31.6, 31.7)	0.2 (0.2, 0.2)	$22.1 (21.2, 22.9)	33.1 (29.5, 34.4)	0.2 (0.1, 0.2)	$19.4 (17.5, 21.0)
Sri Lanka	4.5 (4.5, 4.6)	0.1 (0.1, 0.1)	$3.0 (2.9, 3.1)	3.3 (2.4, 4.0)	0.1 (0.1, 0.1)	$2.1 (1.6, 2.6)	3.1 (2.0, 4.3)	0.1 (0.1, 0.2)	$1.8 (1.2, 2.5)
Thailand	316.6 (314.6, 318.3)	2.3 (2.3, 2.3)	$533.0 (523.0, 542.9)	326.9 (326.6, 329.0)	2.4 (2.4, 2.4)	$546.8 (531.8, 560.4)	380.1 (379.9, 380.6)	3.0 (2.9, 3.1)	$602.9 (569.6, 631.1)
Viet Nam	222.4 (222.2, 223.5)	3.9 (3.9, 3.9)	$172.0 (166.5, 177.6)	255.7 (222.3, 277.8)	4.1 (4.1, 4.1)	$186.9 (164.5, 204.9)	279.6 (250.6, 292.2)	4.6 (4.4, 4.8)	$178.5 (160.4, 194.0)
**Western and Central Africa**									
Benin	68.2 (68.2, 68.3)	4.4 (4.0, 4.8)	$50.5 (48.6, 52.0)	74.0 (52.9, 90.9)	5.1 (4.2, 6.4)	$52.1 (37.9, 70.0)	67.0 (60.6, 74.3)	2.8 (2.4, 3.4)	$41.6 (38.5, 44.9)
Burkina Faso	100.6 (88.9, 106.3)	14.1 (12.3, 16.1)	$68.1 (62.5, 73.1)	82.1 (71.0, 94.7)	9.7 (8.2, 11.3)	$58.7 (52.2, 66.2)	102.1 (88.9, 109.0)	7.6 (6.2, 9.1)	$66.0 (58.9, 73.4)
Cameroon	408.3 (359.3, 459.5)	20.1 (18.4, 21.8)	$245.7 (224.4, 267.1)	292.4 (253.8, 330.5)	19.0 (17.0, 20.9)	$212.6 (188.4, 234.9)	428.5 (392.0, 469.3)	15.5 (13.6, 17.7)	$241.9 (223.5, 261.2)
Central African Republic	37.9 (34.1, 41.8)	2.4 (2.1, 2.7)	$25.8 (24.0, 27.6)	35.8 (29.8, 42.5)	2.4 (2.0, 2.8)	$25.9 (22.1, 30.2)	35.1 (30.7, 40.0)	1.8 (1.4, 2.2)	$23.1 (20.6, 25.9)
Chad	96.2 (84.3, 108.9)	7.2 (6.3, 8.2)	$62.0 (56.6, 67.3)	78.1 (66.0, 91.4)	6.5 (5.5, 7.6)	$54.4 (47.4, 62.3)	119.0 (104.1, 135.1)	4.3 (3.4, 5.5)	$71.1 (63.4, 79.3)
Congo	35.5 (31.1, 40.1)	3.5 (3.0, 4.1)	$27.6 (25.3, 29.9)	29.0 (24.1, 35.6)	3.7 (3.0, 4.8)	$24.7 (21.3, 29.5)	41.7 (36.1, 48.9)	2.6 (2.0, 3.3)	$30.2 (26.7, 34.8)
Côte d’Ivoire	254.0 (249.1, 254.5)	14.1 (13.1, 15.1)	$190.1 (177.8, 199.6)	227.7 (193.9, 261.7)	12.0 (10.9, 13.2)	$177.6 (155.5, 198.9)	243.8 (209.1, 282.9)	7.7 (6.7, 8.8)	$171.8 (153.4, 192.3)
Democratic Republic of the Congo	460.1 (422.7, 467.2)	39.2 (35.1, 43.2)	$287.2 (265.7, 303.0)	266.0 (236.3, 294.6)	24.2 (21.4, 27.1)	$174.4 (158.6, 190.2)	207.3 (176.0, 244.1)	24.1 (19.8, 29.1)	$135.2 (119.6, 152.6)
Equatorial Guinea	18.8 (18.7, 18.8)	0.7 (0.6, 0.8)	$16.1 (15.9, 16.4)	23.8 (23.8, 23.9)	0.7 (0.6, 0.9)	$19.2 (18.4, 19.9)	20.9 (17.4, 25.0)	0.7 (0.5, 0.9)	$15.3 (13.2, 17.8)
Gabon	36.5 (36.4, 36.6)	2.1 (2.0, 2.2)	$36.5 (35.9, 37.2)	34.7 (34.7, 35.0)	2.2 (1.9, 2.4)	$34.3 (32.0, 36.0)	39.0 (38.8, 39.0)	1.7 (1.4, 2.0)	$37.2 (34.2, 39.5)
Gambia	10.1 (8.9, 11.4)	2.1 (1.6, 2.5)	$7.0 (6.4, 7.6)	8.0 (6.4, 9.9)	1.4 (1.2, 1.7)	$5.8 (4.8, 6.9)	12.6 (10.5, 14.0)	3.2 (2.1, 4.4)	$8.4 (7.1, 9.7)
Ghana	193.3 (192.8, 195.4)	13.2 (10.9, 14.2)	$162.2 (158.3, 165.8)	198.0 (196.3, 198.7)	11.8 (8.9, 14.6)	$156.5 (150.3, 162.0)	207.5 (202.5, 208.1)	18.4 (13.3, 24.5)	$149.9 (131.8, 166.7)
Guinea	147.2 (134.6, 149.9)	6.1 (5.4, 6.8)	$92.9 (85.2, 99.0)	103.0 (90.5, 115.5)	4.2 (3.7, 4.8)	$66.1 (59.2, 72.8)	78.2 (65.5, 93.3)	4.3 (3.5, 5.2)	$48.2 (41.9, 55.4)
Guinea Bissau	17.3 (15.2, 19.5)	2.2 (2.0, 2.5)	$11.3 (10.3, 12.2)	13.9 (12.3, 15.7)	2.2 (2.0, 2.6)	$9.9 (9.0, 10.9)	19.7 (17.8, 21.8)	3.1 (2.5, 3.8)	$12.6 (11.7, 13.7)
Liberia	11.0 (9.6, 12.4)	1.0 (0.9, 1.1)	$8.2 (7.5, 8.8)	8.9 (7.4, 10.5)	1.0 (0.8, 1.1)	$7.2 (6.2, 8.3)	12.5 (10.9, 14.3)	0.7 (0.6, 0.8)	$8.6 (7.7, 9.7)
Mali	70.1 (61.4, 79.1)	10.1 (7.9, 13.0)	$49.2 (45.0, 53.3)	62.0 (51.9, 71.7)	6.8 (5.5, 8.4)	$45.3 (38.9, 51.1)	70.7 (53.3, 90.0)	6.2 (4.0, 9.5)	$47.9 (38.1, 58.1)
Mauritania	5.8 (5.8, 5.9)	0.1 (0.1, 0.1)	$4.2 (4.1, 4.3)	5.8 (4.9, 6.6)	0.1 (0.1, 0.1)	$4.1 (3.5, 4.8)	4.9 (3.9, 6.1)	0.1 (0.1, 0.1)	$3.5 (2.9, 4.2)
Niger	26.1 (25.8, 26.1)	1.2 (1.0, 1.4)	$18.9 (18.6, 19.2)	29.3 (23.6, 32.5)	1.8 (1.5, 2.2)	$20.1 (16.4, 23.4)	25.1 (20.7, 30.1)	1.0 (0.8, 1.2)	$16.5 (14.1, 19.1)
Nigeria	1,849.7 (1,619.8, 2,092.8)	179.1 (168.5, 190.5)	$1,211.8 (1,107.9, 1,318.8)	1,601.5 (1,289.0, 1,880.4)	209.4 (193.7, 226.7)	$1,245.0 (1,032.2, 1,431.4)	1,431.8 (1,221.3, 1,669.2)	127.8 (114.2, 142.8)	$1,016.2 (905.5, 1,133.7)
Senegal	32.6 (32.0, 32.7)	2.4 (2.1, 2.9)	$24.8 (23.2, 26.1)	27.5 (20.1, 32.2)	2.2 (1.9, 2.8)	$21.5 (15.9, 27.6)	30.2 (23.4, 34.9)	2.7 (2.1, 3.5)	$21.9 (17.4, 28.1)
Sierra Leone	22.1 (19.3, 25.2)	1.5 (1.2, 1.8)	$14.2 (12.9, 15.4)	22.1 (19.2, 25.3)	1.3 (1.0, 1.6)	$15.5 (13.8, 17.4)	22.1 (18.5, 26.2)	1.1 (0.9, 1.4)	$14.0 (12.2, 16.1)
Togo	86.7 (75.9, 98.1)	8.6 (7.5, 8.8)	$58.8 (53.8, 63.7)	79.2 (58.2, 100.0)	7.6 (6.1, 7.9)	$57.7 (44.4, 71.8)	77.9 (55.4, 107.8)	5.8 (3.6, 8.8)	$51.5 (38.6, 69.1)
**Eastern Europe and Central Asia**									
Armenia	3.0 (3.0, 3.0)	0.02 (0.02, 0.02)	$1.9 (1.9, 2.0)	5.0 (3.9, 5.3)	0.03 (0.03, 0.03)	$2.7 (2.1, 3.2)	6.5 (4.5, 8.0)	0.02 (0.01, 0.04)	$2.9 (2.0, 3.8)
Azerbaijan	8.0 (6.4, 9.7)	0.2 (0.2, 0.3)	$6.9 (5.8, 8.0)	9.0 (7.4, 10.7)	0.3 (0.2, 0.3)	$8.6 (7.3, 10.0)	11.8 (9.8, 14.3)	0.3 (0.3, 0.4)	$9.1 (7.7, 10.8)
Belarus	20.3 (20.2, 20.3)	0.4 (0.4, 0.4)	$29.5 (27.1, 31.4)	17.0 (16.9, 17.0)	0.5 (0.4, 0.5)	$27.0 (25.0, 28.6)	36.9 (32.1, 38.2)	0.5 (0.4, 0.5)	$30.6 (26.9, 34.2)
Bulgaria	2.3 (2.3, 2.3)	0.1 (0.1, 0.1)	$2.6 (2.6, 2.7)	2.9 (2.9, 2.9)	0.1 (0.1, 0.1)	$3.5 (3.4, 3.6)	3.2 (3.2, 3.2)	0.1 (0.1, 0.1)	$3.5 (3.4, 3.7)
Georgia	5.2 (5.1, 5.2)	0.1 (0.1, 0.1)	$4.2 (4.1, 4.2)	7.2 (7.0, 7.2)	0.1 (0.1, 0.1)	$5.9 (5.8, 6.0)	9.4 (9.4, 9.4)	0.1 (0.1, 0.1)	$6.9 (6.5, 7.3)
Kazakhstan	6.5 (6.5, 6.5)	0.2 (0.2, 0.2)	$9.4 (9.2, 9.6)	7.5 (7.5, 7.5)	0.2 (0.2, 0.2)	$10.7 (10.5, 11.0)	13.1 (13.1, 13.1)	1.1 (1.1, 1.2)	$16.2 (15.6, 16.7)
Kyrgyzstan	5.2 (4.9, 5.2)	0.2 (0.2, 0.2)	$3.1 (2.7, 3.3)	6.0 (5.1, 7.1)	0.2 (0.2, 0.2)	$3.7 (3.3, 4.3)	6.4 (5.2, 7.9)	0.2 (0.1, 0.2)	$3.3 (2.8, 4.0)
Republic of Moldova	10.8 (10.8, 10.8)	0.3 (0.3, 0.3)	$7.0 (6.6, 7.3)	14.2 (12.8, 14.7)	0.4 (0.4, 0.4)	$8.5 (7.8, 9.1)	16.0 (13.9, 16.6)	0.4 (0.4, 0.4)	$8.5 (7.5, 9.4)
Romania	16.3 (16.3, 16.3)	0.4 (0.4, 0.4)	$23.0 (22.6, 23.3)	18.0 (18.0, 18.0)	0.5 (0.5, 0.5)	$25.6 (25.1, 26.1)	18.6 (18.6, 18.7)	0.6 (0.6, 0.6)	$26.4 (25.8, 27.0)
Russia	734.1 (734.0, 734.9)	12.5 (12.5, 12.5)	$4,377.5 (4,149.0, 4,473.1)	948.3 (891.0, 992.4)	16.1 (16.1, 16.1)	$5,054.2 (4,747.6, 5,317.6)	1,053.8 (901.2, 1,122.4)	18.8 (18.4, 19.3)	$5,114.9 (4,482.3, 5,757.4)
Serbia	3.0 (3.0, 3.0)	0.1 (0.1, 0.1)	$3.7 (3.6, 3.8)	3.5 (3.4, 3.5)	0.1 (0.1, 0.1)	$4.4 (4.3, 4.5)	3.7 (3.7, 3.7)	0.1 (0.1, 0.1)	$4.6 (4.4, 4.8)
Tajikistan	6.3 (5.6, 7.1)	0.9 (0.9, 0.9)	$3.6 (3.3, 3.9)	7.0 (5.0, 9.0)	1.0 (1.0, 1.0)	$4.4 (3.4, 5.5)	8.1 (5.5, 11.0)	1.2 (1.0, 1.5)	$4.4 (3.3, 5.6)
Ukraine	151.8 (151.5, 152.1)	3.4 (3.4, 3.4)	$121.6 (116.1, 126.0)	172.3 (144.3, 203.3)	3.7 (3.7, 3.7)	$116.5 (101.9, 132.8)	224.2 (223.5, 224.5)	4.4 (4.0, 4.9)	$148.6 (134.6, 162.4)
Uzbekistan	19.3 (16.6, 19.8)	3.8 (3.8, 3.8)	$16.0 (14.5, 17.3)	17.3 (14.7, 20.1)	3.9 (3.9, 3.9)	$16.2 (14.8, 17.7)	22.7 (19.0, 27.5)	4.2 (4.1, 4.3)	$17.7 (16.2, 19.7)
**Latin America and the Caribbean**									
Bahamas	4.1 (3.7, 4.5)	0.03 (0.03, 0.04)	$5.6 (5.3, 6.0)	4.6 (4.2, 4.9)	0.04 (0.03, 0.05)	$6.7 (6.3, 7.0)	4.5 (3.7, 5.3)	0.1 (0.1, 0.1)	$6.0 (5.4, 6.7)
Barbados	1.7 (1.6, 1.9)	0.02 (0.02, 0.02)	$2.6 (2.5, 2.7)	1.8 (1.4, 2.2)	0.04 (0.04, 0.04)	$2.7 (2.3, 3.3)	2.2 (1.8, 2.4)	0.03 (0.02, 0.03)	$3.0 (2.5, 3.5)
Belize	2.8 (2.5, 3.1)	0.1 (0.1, 0.1)	$3.9 (3.7, 4.2)	2.4 (2.1, 2.7)	0.1 (0.1, 0.1)	$3.5 (3.2, 3.9)	3.4 (2.4, 4.1)	0.1 (0.1, 0.2)	$4.4 (3.3, 5.4)
Bolivia	5.7 (5.1, 6.4)	0.1 (0.0, 0.2)	$5.3 (5.0, 5.7)	7.5 (2.6, 16.1)	0.1 (0.04, 0.4)	$7.4 (3.1, 14.8)	7.9 (2.9, 14.8)	0.1 (0.03, 0.4)	$6.7 (3.0, 11.8)
Cuba	26.4 (24.7, 28.4)	0.02 (0.01, 0.02)	$33.1 (31.6, 34.8)	22.0 (21.8, 22.0)	0.04 (0.04, 0.04)	$31.2 (30.8, 31.6)	27.4 (12.7, 35.1)	0.04 (0.02, 0.1)	$34.4 (16.9, 50.1)
Dominican Republic	36.5 (33.7, 37.5)	0.7 (0.7, 0.8)	$54.5 (51.4, 57.4)	35.9 (27.7, 42.9)	0.7 (0.6, 0.8)	$56.1 (45.1, 66.0)	42.8 (35.8, 44.3)	0.8 (0.7, 1.0)	$60.2 (49.9, 69.6)
Guatemala	33.7 (30.1, 37.5)	2.8 (1.1, 5.7)	$30.2 (27.7, 33.2)	36.6 (17.7, 67.5)	3.4 (1.1, 8.1)	$33.4 (18.0, 61.6)	59.2 (35.9, 76.8)	4.4 (0.8, 12.4)	$45.1 (28.3, 69.2)
Guyana	7.4 (7.4, 7.5)	0.6 (0.6, 0.6)	$7.5 (7.3, 7.7)	8.7 (8.4, 8.9)	0.8 (0.6, 0.8)	$8.9 (8.2, 9.4)	9.1 (7.2, 9.6)	0.7 (0.5, 0.9)	$8.4 (6.4, 10.1)
Haiti	137.3 (137.1, 138.1)	4.0 (3.2, 4.7)	$108.1 (106.2, 109.7)	135.8 (107.9, 142.2)	4.2 (3.2, 5.2)	$100.4 (77.3, 113.4)	127.5 (103.1, 150.4)	8.8 (6.8, 11.1)	$88.2 (74.6, 102.1)
Honduras	13.6 (12.2, 15.1)	0.9 (0.8, 0.9)	$14.9 (14.1, 15.8)	15.5 (13.4, 18.0)	1.0 (0.9, 1.1)	$17.2 (15.4, 19.3)	20.3 (17.9, 23.1)	1.1 (0.9, 1.4)	$18.4 (16.8, 20.2)
Jamaica	14.1 (12.7, 15.6)	0.5 (0.5, 0.5)	$21.4 (20.1, 22.8)	14.9 (10.0, 20.3)	0.5 (0.5, 0.5)	$23.7 (17.2, 30.8)	16.0 (12.7, 19.9)	0.5 (0.4, 0.6)	$22.7 (19.3, 26.6)
Nicaragua	8.6 (8.1, 9.3)	0.5 (0.4, 0.6)	$5.9 (5.6, 6.2)	10.1 (8.8, 11.1)	0.6 (0.5, 0.8)	$7.5 (6.7, 8.2)	9.4 (6.8, 12.3)	0.8 (0.6, 1.1)	$6.4 (4.9, 8.0)
Suriname	3.4 (3.2, 3.4)	0.2 (0.2, 0.3)	$4.6 (4.3, 4.8)	3.6 (3.4, 4.0)	0.3 (0.2, 0.3)	$5.1 (4.9, 5.4)	3.8 (3.2, 4.1)	0.3 (0.3, 0.4)	$4.7 (4.2, 5.3)
Trinidad and Tobago	14.2 (14.2, 14.3)	0.2 (0.1, 0.2)	$21.1 (20.7, 21.5)	14.1 (13.8, 14.1)	0.2 (0.1, 0.2)	$20.8 (20.1, 21.4)	14.5 (12.3, 15.8)	0.2 (0.2, 0.2)	$19.1 (17.2, 21.1)
**Middle East and North Africa**									
Algeria	10.2 (9.3, 11.2)	1.8 (1.5, 1.8)	$12.6 (11.7, 13.4)	9.0 (4.6, 16.5)	1.5 (1.3, 1.5)	$11.9 (6.8, 20.5)	17.4 (10.7, 27.6)	1.9 (1.3, 2.5)	$18.0 (11.8, 27.4)
Djibouti	2.2 (2.0, 2.4)	0.03 (0.02, 0.03)	$1.9 (1.8, 2.1)	1.8 (1.6, 2.1)	0.05 (0.04, 0.1)	$1.8 (1.5, 2.0)	3.7 (3.2, 4.2)	0.1 (0.1, 0.1)	$2.7 (2.4, 3.0)
Egypt	8.4 (8.2, 8.7)	0.2 (0.2, 0.3)	$4.9 (4.8, 5.0)	7.3 (5.9, 8.6)	0.3 (0.2, 0.3)	$4.4 (3.6, 5.2)	8.5 (6.5, 10.9)	0.4 (0.3, 0.5)	$4.4 (3.5, 5.5)
Iran	11.1 (10.1, 12.2)	0.8 (0.7, 0.8)	$13.2 (12.4, 14.0)	14.2 (13.2, 15.1)	0.8 (0.7, 0.8)	$17.8 (16.9, 18.7)	16.4 (15.0, 17.6)	1.2 (1.0, 1.5)	$17.1 (16.1, 18.0)
Morocco	30.9 (30.0, 31.9)	1.0 (0.9, 1.0)	$21.8 (21.3, 22.4)	26.0 (24.3, 27.7)	1.0 (1.0, 1.0)	$19.7 (18.6, 20.9)	32.1 (26.9, 38.4)	1.2 (0.8, 1.5)	$19.8 (17.0, 23.2)
Somalia	2.8 (2.5, 3.0)	1.0 (0.8, 1.3)	$2.2 (2.0, 2.4)	3.5 (2.6, 4.6)	1.3 (1.0, 1.8)	$2.9 (2.3, 3.7)	4.1 (3.2, 5.2)	1.5 (0.9, 2.4)	$2.9 (2.4, 3.5)
Sudan	12.9 (11.6, 14.0)	0.7 (0.5, 0.9)	$9.2 (8.6, 9.8)	10.4 (7.8, 13.4)	0.8 (0.5, 1.2)	$8.0 (6.1, 10.1)	13.4 (10.3, 16.7)	1.4 (0.8, 2.2)	$8.5 (6.8, 10.4)
Tunisia	1.7 (1.6, 1.9)	0.1 (0.1, 0.1)	$1.8 (1.7, 1.9)	2.4 (1.7, 3.3)	0.1 (0.1, 0.1)	$2.7 (1.9, 3.5)	2.8 (2.0, 3.7)	0.1 (0.1, 0.1)	$2.5 (1.9, 3.3)
Yemen	3.6 (3.3, 3.9)	0.6 (0.2, 1.2)	$2.6 (2.3, 2.9)	2.9 (0.4, 14.4)	0.6 (0.2, 1.5)	$2.6 (0.6, 11.8)	4.6 (1.0, 13.9)	0.8 (0.2, 2.1)	$3.2 (1.0, 8.8)

Under the current eligibility scenario, 64% of adults and 69% of children on ART in 2020 could be in Eastern and Southern Africa. Approximately 16% of all adults and 21% of all children on ART globally could reside in Western and Central Africa in 2020. About 15% of adults on ART could be in Asia and the Pacific under this scenario. The region with the smallest proportion of the global estimate of people on ART is the Middle East and North Africa: just 0.3% of adults and 0.4% of children on treatment globally could be from this region in 2020. Similar patterns are evident in the other two scenarios, although the Asia/Pacific region has the second highest number of adults on treatment under the WHO 2013 and 90-90-90 scenarios in 2020.

We estimate that approximately 0.5 million adults and 0.04 million children received second-line ARVs in 2013. By 2020, 2.5 (95% CI 2.3, 2.8) million adults living with HIV will be on second-line treatment under the current eligibility scenario. Under the WHO 2013 and 90-90-90 scenarios, we estimate that 2.6 (95% CI 2.4, 2.9) and 2.8 (95% CI 2.5, 3.x) million adults, respectively, will be on second-line treatment in 2020. Across all three scenarios, the number of children living with HIV on second-line treatment in 2020 is about the same, at 0.17 million (exact point estimates and confidence intervals vary slightly by scenario).

### Resource Requirements for HIV Treatment

**Fig 7:**
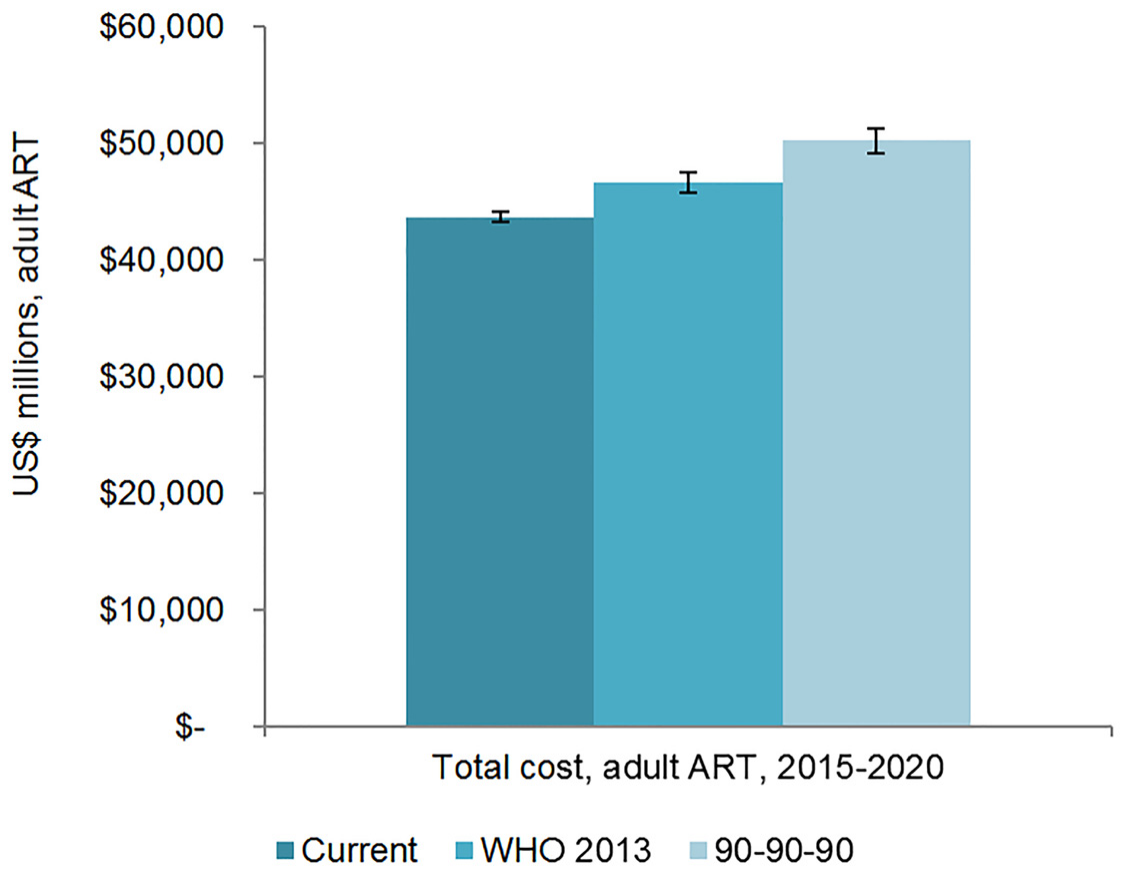
Total adult resource needs for HIV treatment by scenario. The vertical axis shows the cost of HIV treatment in millions of US dollars, and the horizontal axis shows the three scenarios. The whiskers show the upper and lower bounds of the 95% confidence interval.

**Fig 8:**
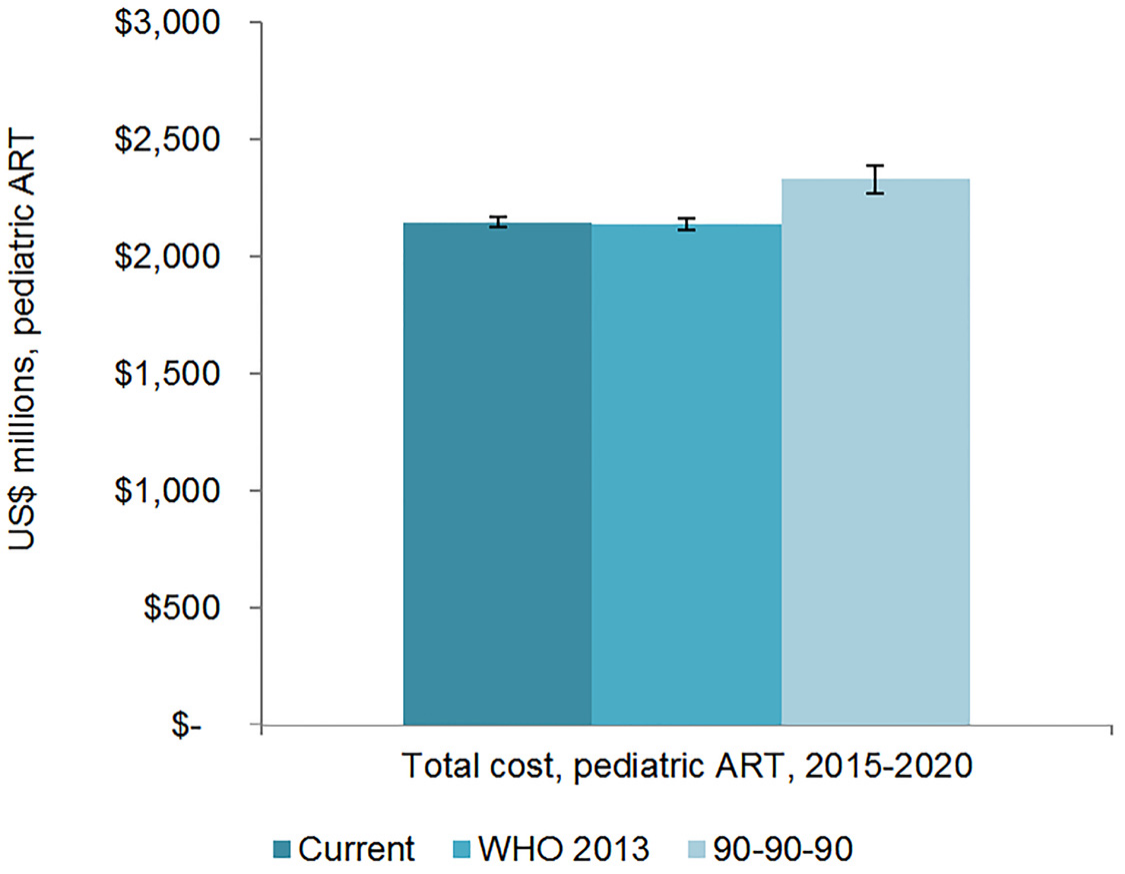
Total pediatric resource needs for HIV treatment by scenario. The vertical axis shows the cost of HIV treatment in millions of US dollars, and the horizontal axis shows the three scenarios. The whiskers show the upper and lower bounds of the 95% confidence interval.

The total 6-y cost of scaling up adult ART globally from 2015 to 2020 is estimated to be US$43.6 (95% CI 43.2, 44.1) billion under the current eligibility scenario, US$46.6 (95% CI 45.7, 47.5) billion under the WHO 2013 eligibility scenario, and US$50.2 (95% CI 49.1, 51.3) billion under the 90-90-90 scenario (Figs 7 and 8). The cost of ARVs and laboratory commodities for adult ART over the same time period is US$23.9 (95% CI 23.6, 24.1) billion, US$25.2 (95% CI 24.8, 25.7) billion, and US$26.9 (95% CI 26.2, 27.6) billion under the current eligibility, WHO 2013, and 90-90-90 scenarios, respectively. The cost of pediatric treatment across all 6 y for the three scenarios is US$2.14 (95% CI 2.12, 2.17) billion, US$2.14 (95% CI 2.11, 2.16) billion, and US$2.33 (95% CI 2.27, 2.39) billion, with ARVs and laboratory commodities costing US$1.17 (95% CI 1.15, 1.18) billion, US$1.15 (95% CI 1.13, 1.17) billion, and US$1.22 (95% CI 1.19, 1.24) billion under the current eligibility, WHO 2013, and 90-90-90 scenarios, respectively. If countries implemented targeted rather than routine viral load testing, we estimate that total 6-y laboratory costs would decrease by approximately US$1.52 billion, US$1.53 billion, and US$1.63 billion under the current eligibility, WHO 2013, and 90-90-90 scenarios, respectively.

Facility-level personnel and overhead costs represent 45% to 47% of the total 6-y ART costs for adult ART and 46% to 48% of the total ART costs for pediatric ART across the scenarios. ARVs are also a significant proportion of the resource requirements for ART; depending on the scenario, ARVs represent between 41% and 45% of total 6-y ART costs. By 2020, second-line adult ARVs cost US$0.67 (95% CI 0.62, 0.72) billion under the current eligibility scenario, US$0.70 (95% CI 0.64, 0.76) billion under the WHO 2013 scenario, and US$0.72 (95% CI 0.66, 0.78) billion under the 90-90-90 scenario. Across all scenarios, 6-y laboratory testing costs are about 9% to 10% of total adult ART costs and between 11% and 12% of pediatric ART costs.

**Fig 9:**
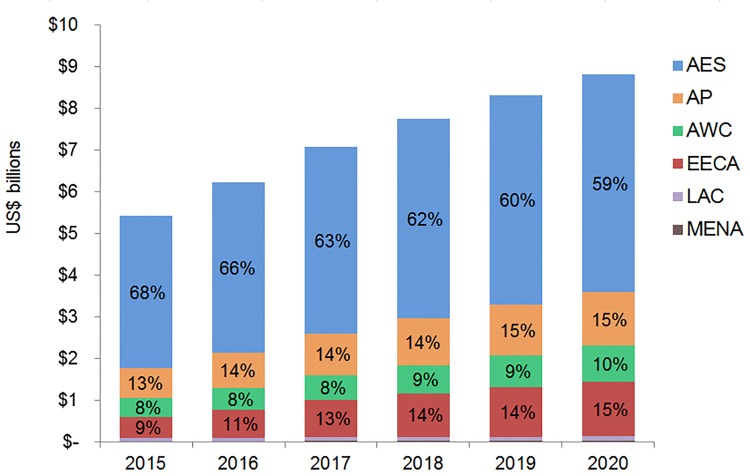
Total adult ART resource requirements by region. The graph shows percent of total adult ART resource requirements by region based on the current eligibility scenario. The vertical axis shows the cost of HIV treatment in billions of US dollars, and the horizontal axis shows the years of analysis. Labeled values do not equal 100% as only selected percentages are shown. AES, Eastern and Southern Africa; AP, Asia and the Pacific; AWC, Western and Central Africa; EECA, Eastern Europe and Central Asia; LAC, Latin America and the Caribbean; MENA, Middle East and North Africa.

**Fig 10:**
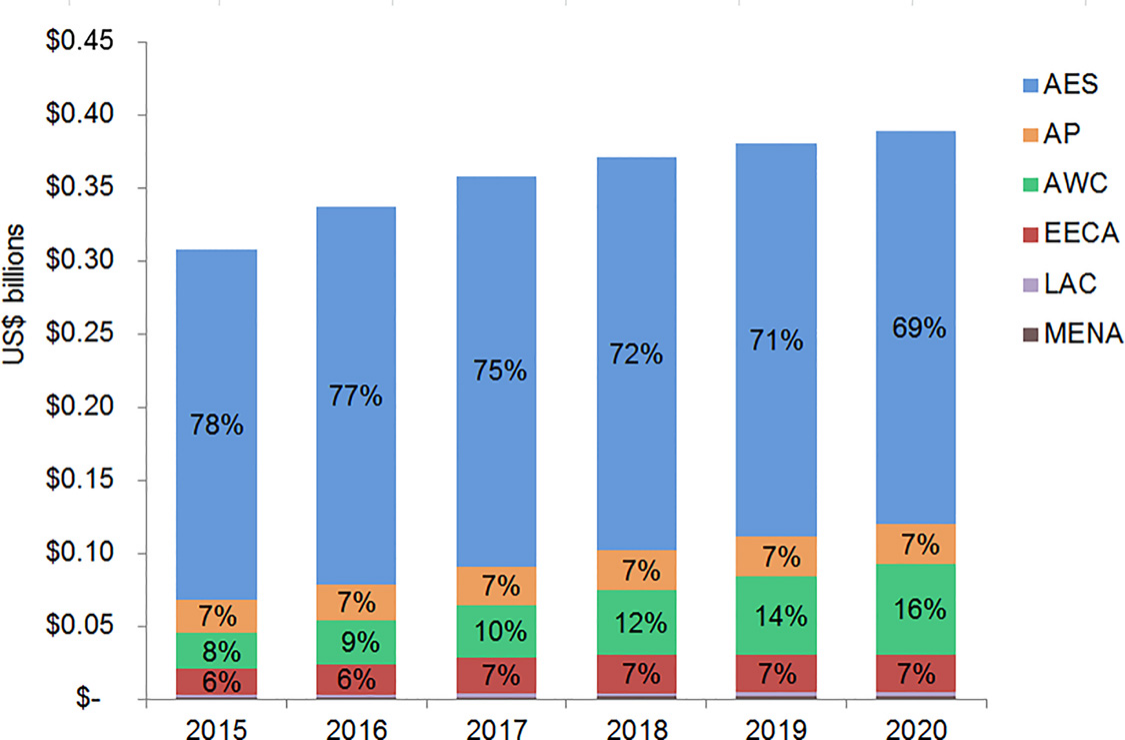
Total pediatric ART resource requirements by region. The graph shows percent of total pediatric ART resource requirements by region based on the current eligibility scenario. The vertical axis shows the cost of HIV treatment in billions of US dollars, and the horizontal axis shows the years of analysis. Labeled values do not equal 100% as only selected percentages are shown. AES, Eastern and Southern Africa; AP, Asia and the Pacific; AWC, Western and Central Africa; EECA, Eastern Europe and Central Asia; LAC, Latin America and the Caribbean; MENA, Middle East and North Africa.

The majority of adult and pediatric ART resource requirements are for people living with HIV in Eastern and Southern Africa. Under the current eligibility scenario, US$28.9 (95% CI 28.5, 29.3) billion is needed from 2015 to 2020 for adult and pediatric ART in Eastern and Southern Africa (Figs 9 and 10). The Asia/Pacific region has the second-highest estimated ART resource requirements under this scenario, at US$6.3 (95% CI 6.2, 6.4) billion. Due to high ARV costs in Russia, the Eastern Europe and Central Asia region has the third highest total 6-y costs under the current eligibility scenario, $5.8 (95% CI 5.6, 5.9) billion. The Middle East and North Africa region requires the lowest resources for adult and pediatric ART across our three scenarios. The proportion of total costs by region changes over time. For adult treatment under the current eligibility scenario, the cost for treatment in Eastern and Southern Africa as a percentage of total global costs declines from 68% in 2015 to 59% by 2020, while the proportionate cost for treatment in Eastern Europe and Central Asia increases from 9% to 15% of total global adult ART costs, driven by Russia. While the cost of adult ART for Western and Central Africa in relation to total global ART costs remains relatively constant from 2015 to 2020, the cost of pediatric ART in the region increases from 8% to 16% of total global pediatric costs.

### Financial Resources Available for ART

Based on our estimate of average annual funding levels, we project that the Global Fund will finance approximately US$0.4 billion of the annual ART commodity requirements for the countries eligible for Global Funding in our sample. This is in line with the Global Fund’s own estimates of support for commodities, which show that it expended US$0.47 billion on adult and pediatric ARVs in 2014 [97]. For the applicable countries in our study, total annual PEPFAR contributions to ARV and laboratory commodity procurement are estimated at US$0.46 billion, which may be an optimistic estimate [98]. Our estimate for annual PEPFAR contributions to personnel and overhead at the facility level as included in our analysis ranges from US$0.47 to US$0.65 billion per year globally based on fiscal year 2014 expenditures by country, which again may be optimistic, even given PEPFAR’s refocusing on this issue.

Estimated DCs vary by scenario. We estimated that countries will contribute US$6.2 billion in domestic resources to commodity procurement from 2015 to 2020 under all scenarios. Under the current eligibility scenario, DCs for facility-level personnel and overhead costs are estimated to increase from US$1.4 billion in 2015 to US$2.3 billion in 2020. DCs for these types of costs are estimated to increase from US$1.6 billion to US$2.6 billion under the WHO 2013 scenario and from US$1.5 billion to US$2.9 billion under the 90-90-90 scenario over the same time period.

**Fig 11:**
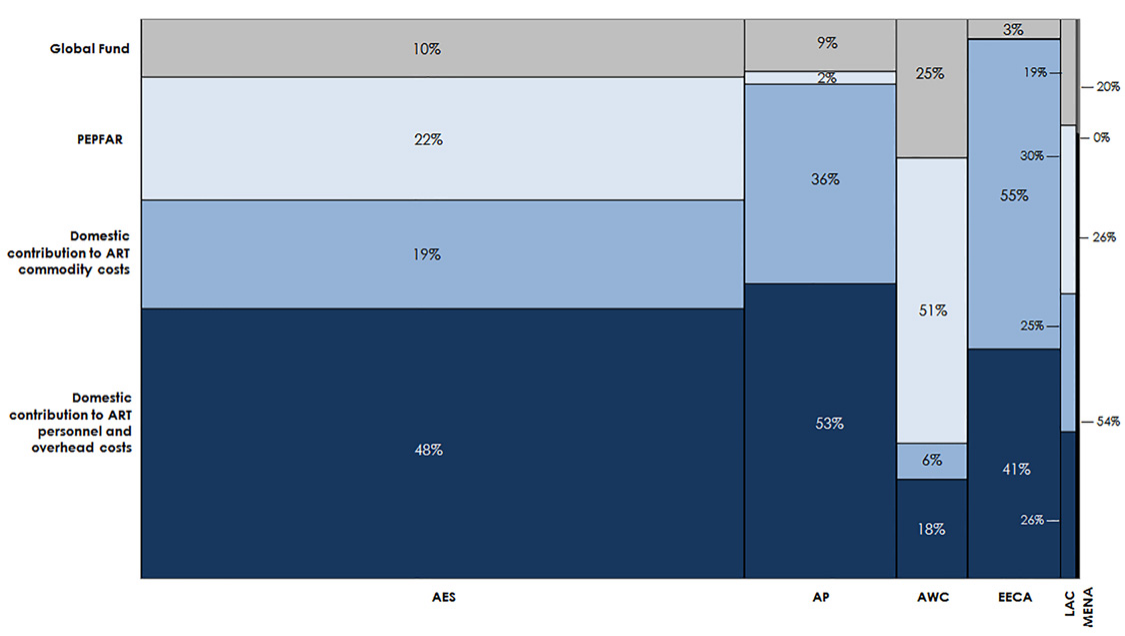
Estimated financial resources available for ART. This chart shows the total estimated financial resources available for ART by region, disaggregated by source, under the current eligibility scenario, assuming lower PEPFAR contributions to facility-level costs. The width of each region is proportional to the total volume of resources available. The MENA region has a narrow column because there are significantly fewer resources available for HIV in MENA than in the other regions in our analysis. AES, Eastern and Southern Africa; AP, Asia and the Pacific; AWC, Western and Central Africa; EECA, Eastern Europe and Central Asia; LAC, Latin America and the Caribbean; MENA, Middle East and North Africa.

Estimated funding available for ART varies greatly by region (Fig 11). Based on the current eligibility scenario and lower PEPFAR contributions to facility-level costs, we estimate that 70% of the external funding available for ART from 2015 to 2020 could go to countries in Eastern and Southern Africa. One-fifth of external funds during this period could support countries in Western and Central Africa. Under our optimistic assumptions, DCs could account for the majority of funding available for ART in Eastern and Southern Africa (68%), Asia and the Pacific (88%), Eastern Europe and Central Asia (96%), Latin America and the Caribbean (51%), and the Middle East and North Africa (80%). These values are ambitious, yet comparable with UNAIDS estimates [6]. The Western and Central Africa region is the only region that is estimated to be primarily funded by external sources (76%). DCs to ART costs in Eastern Europe and Central Asia are largely due to projected high DCs from Russia. South Africa is estimated to contribute US$7.8 billion for ART from 2015 to 2020 from domestic sources, representing 72% of the total domestic resources available for ART in the Eastern and Southern Africa region. Similarly, Nigeria’s potential contributions account for 32% of the total domestic resources available for ART in Western and Central Africa.

### HIV Treatment Funding Gap

Under the current eligibility scenario and given conservative PEPFAR and optimistic DC assumptions, the funding gap for ART is estimated to increase from US$2.1 billion to US$4.6 billion per year from 2015 to 2020. The funding gap for ART commodities in the same time frame increases from US$1.3 billion to US$3.1 billion per year. The total ART funding gap spanning the 6-y period from 2015 to 2020 is projected to be US$20.6 billion. Under more expansive assumptions for PEPFAR contributions to facility-level overhead and personnel costs, the total 6-y ART gap would decrease slightly to US$19.8 billion. Under the WHO 2013 scenario, the total funding gap for ART ranges from US$21.6 to US$22.4 billion, depending on PEPFAR contributions, and the 6-y commodity gap is estimated to be US$15.2 billion. The funding gap over the same time period for the 90-90-90 scenario is estimated to be US$24.2 billion to US$25.0 billion, depending on PEPFAR contributions, and the estimated commodity gap is US$16.8 billion.

The overwhelming share (61%) of the funding gap for ART is in Eastern and Southern African countries under the current eligibility and lower PEPFAR contribution scenario. Approximately 16% of the total ART gap emerges in Eastern Europe and Central Asia. Western and Central Africa and Asia and the Pacific account for significant portions the total funding gap (11% each). Funding gaps in Latin America and the Caribbean and the Middle East and North Africa are relatively small, representing just over 1% of the total ART funding gap combined. UNAIDS estimates these regions will fund up to 90% of their overall HIV response, and studies indicate that UMICs are capable of meeting their HIV resource requirements entirely through domestic sources [6,15].

**Fig 12:**
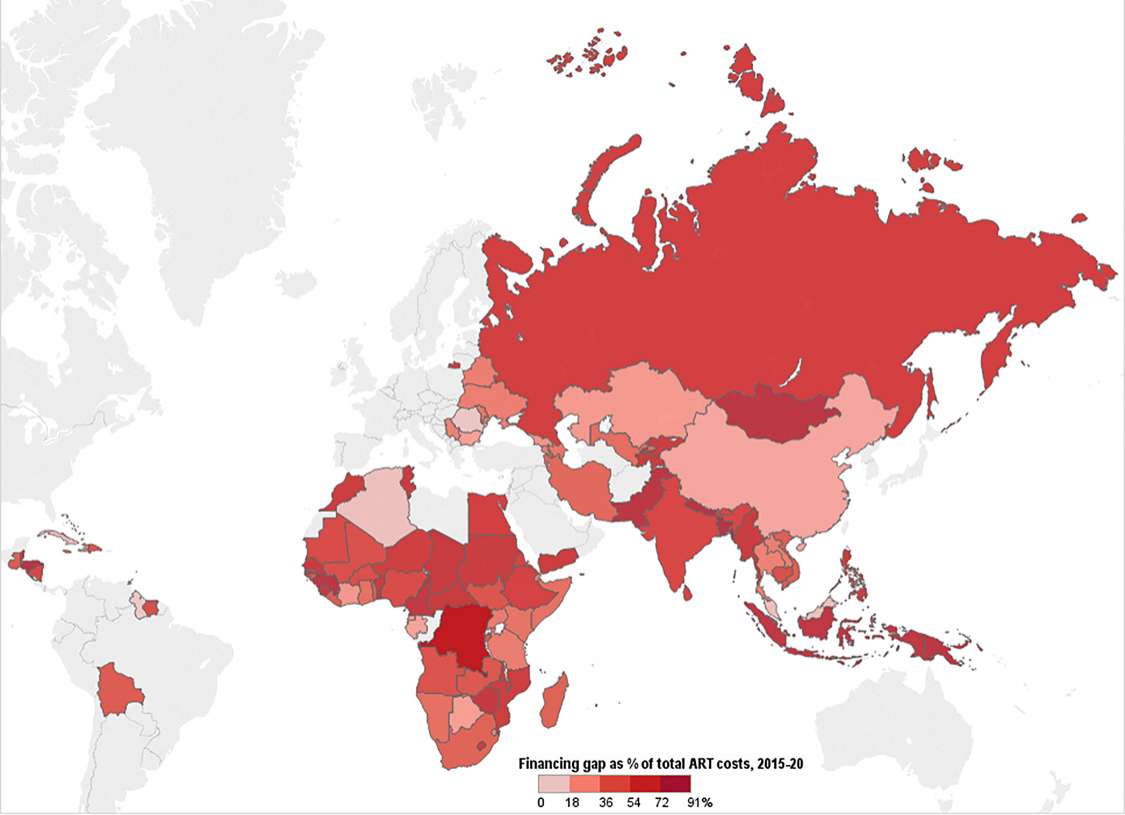
Funding gap for HIV treatment. This map shows the percentage of total costs under the current eligibility scenario—assuming conservative PEPFAR contributions—that remains unfunded after incorporating Global Fund, PEPFAR, and domestic contributions to ART. Countries' funding gaps are larger under the WHO 2013 and 90-90-90 scenarios.

Fig 12 shows the percentage of total ART costs under the current eligibility scenario that is not financed for the entire 6-y period, by country, with conservative PEPFAR contribution assumptions. Of the 97 countries in our analysis, 45 are estimated to have a total ART funding gap that exceeds 50% of the total resource requirements under this scenario. We estimate that 11 countries will have over 75% of their resource requirements unmet from 2015 to 2020. These funding gaps are even greater under the WHO 2013 and 90-90-90 scenarios, assuming the same level of PEPFAR contributions.

## Discussion

To our knowledge, this study is the first to incorporate an uncertainty analysis using recent country- and region-specific epidemiological, coverage, and unit cost data in projecting facility-level adult and pediatric ART resource requirements and funding gaps across multiple scenarios for the number of treatment-eligible people living with HIV. Our results indicate that the resources required for the global ART response may be less than previously estimated. However, financial resources mobilized for ART or technical efficiency will need to increase to meet the funding challenge. Even with optimistic assumptions about domestic source contributions, the financing gap is large.

Compared to other modeling studies, our study estimates that more people could be on ART in future years, given ambitious yet achievable coverage increases, if resources permit. A study assessing universal adoption of the 2013 WHO guidelines estimated that 28 million people could be on treatment in 2025; our results indicate that 28 (95% CI 27.5, 28.6) million adults and children could be on ART by 2020 under our WHO 2013 scenario, based on ambitious yet achievable coverage scale-up [8]. Our projections of the number of people eligible for treatment and coverage increases based on historical trends suggest that the 90-90-90 target of 81% of people living with HIV being on treatment by 2020 may not be met, even if all countries universally adopted aspects of the 2013 WHO guidelines beginning in 2014. To achieve the 90-90-90 target, UNAIDS estimated that US$35.6 billion would be needed for the entire HIV response in 2020, and care and treatment services have historically accounted for 55% of HIV spending globally [99]. However, our projections under the 90-90-90 scenario suggest that the facility-level costs of ART provision would amount to US$11.3 billion in 2020, less than what such historical trends suggest. Our results are comparable with other recent studies suggesting that the unit cost of providing ART services may be less than previously estimated [11,53].

In order to rapidly scale up ART coverage globally, other investments are needed. Primarily, investments are needed to scale up HIV testing. The 90-90-90 initiative calls for 90% of all people living with HIV to be diagnosed with HIV by 2020. While our study does not include all costs associated with scaling up HIV treatment, we estimated the financial resources required to diagnose the new patients on ART within each scenario. We calculated the number of new patients on treatment each year by region using the numbers of individuals on treatment in our model and adjusting for mortality in cohorts, and then applied region-specific incidence rates and HIV rapid test costs to forecast the number of people to be tested and the cost of testing (see S3 Text for additional details) [24,100]. We estimate that US$0.51 billion, US$0.55 billion, and US$0.73 billion is needed over the time period 2015 to 2020 for HIV testing under the current eligibility, WHO 2013, and 90-90-90 scenarios, respectively. In addition to investments in HIV diagnostics, the expected scale-up of ART coverage to ambitious levels will not occur in many countries with concentrated or mixed epidemics unless severe structural barriers related to ongoing levels of stigma and discrimination, as well as punitive policies targeting certain high-HIV-burden population groups, are removed [101–103].

Results from other studies indicate that investments in expanding eligibility for treatment and coverage are cost-effective. Stover et al. estimated that universal adoption of the 2013 WHO guidelines would avert 2.9 million deaths and 3.9 million new infections from 2013 to 2025, compared with implementation of the 2010 guidelines [8]. An analysis in four countries—India, South Africa, Zambia, and Viet Nam—found that expanding eligibility to individuals with CD4 count ≤ 500 cells/mm^3^ or to all people living with HIV is very cost-effective in all settings [104]. Vassall et al. estimated that expanding treatment eligibility to all people living with HIV in South Africa would decrease new HIV infections by 45% and save approximately US$10 billion over 40 y [14]. Expanding HIV treatment is particularly cost-effective compared with other interventions; the cost per quality-adjusted life year is between US$1,000 and US$2,000 for earlier ART initiation, compared with US$5,000 to US$25,000 for routine virological monitoring in eastern Africa [105].

Our estimates of the resource requirements for ART suggest that the financial sustainability of a scaled-up global ART response, where the scale-up itself is cost-effective, may be at risk without either additional resource mobilization or efficiency and effectiveness gains. We have already included ambitious domestic resource mobilization assumptions. However, improved technology and changes in service delivery models could reduce overall resource needs and, as a result, lessen the funding gap for HIV treatment.

There is evidence that the unit cost of ARV provision could decrease further than what we anticipated in this study. The introduction of new ARVs (such as the recently approved dolutegravir) that are less expensive to manufacture, are less toxic, and have a higher barrier to resistance than existing ARVs could be a “game changer” [106]. When we replaced the unit costs of adult first- and second-line regimens as used in our analysis with the average cost of these new regimens, the total cost of adult ART over the period 2015 to 2020 decreased by US$7.3 billion. This ARV price reduction would eliminate about half of our estimated 6-y commodity gap, which is projected to be US$14.0 billion under the current eligibility scenario (Table 6). Further, the development of long-acting injectable ARVs and once-daily regimens for HIV treatment may be possible in the near term, which could change how HIV treatment is currently administered and further lower the cost of treatment globally [107]. Dose optimization trials aiming to identify the smallest possible effective dose could lead to additional cost savings by reducing the number or frequency of pills each patient needs; one study found that a lower dose of EFV is effective and could reduce per-patient annual costs by US$16 [108]. Table 6 summarizes how some of these innovations could impact our cost and ART funding gap estimates from 2015 to 2020.

**Table 6:** Impact of new ARV developments on 2015–2020 ART funding gap under current eligibility scenario.

ARV Development	Potential Impact on Unit Cost	ARV Cost Savings (in Billions of US Dollars)	Commodity Gap (in Billions of US Dollars)
Improved three-drug regimens	First-line adult regimens could cost as low as US$60 per patient per year. Second-line adult regimens could cost US$266–US$357 per patient per year.	$1.6 (adult)	$12.7
Improved two-drug maintenance combinations	Cabotegravir/rilpivirine (long-acting injectable) and dolutegravir/rilpivirine are used in patients who are virally suppressed. These new regimens cost about US$40 per patient per year.	$7.0^1^ (adult), $0.3^1^ (children)	$7.5
EFV dose optimization	Dose optimization could reduce the unit cost of EFV by US$16 per patient per year.	$1.0 (adult), $0.02 (children)	$14.0

^1^Assuming 76% of all those on treatment are virally suppressed [6].

Decentralization and task-shifting from doctors to nurses could also lower the facility-level costs of managing HIV treatment without affecting health outcomes for patients [109,110]. Our analysis assumes that the unit cost of clinical personnel for ART, disaggregated by country income level, can be reduced from historical levels in 2015 via increased returns to scale with larger patient volumes. However, these costs may continue to decrease, and at a faster rate than we estimated, as more countries adopt task-shifting policies and improve service provision. These efficiency initiatives may require some short-term investments and could lead to long-term cost savings and improved health outcomes.

Despite opportunities for cost savings, the ART funding gap remains large. We estimate that approximately 43% to 48% of total facility-level resource requirements for ART from 2015 to 2020 will remain unfunded, depending on the cost scenario and assumed PEPFAR funding levels. This is despite the fact that our DC estimates are ambitious. As donors place greater emphasis on domestic financing for HIV treatment, particularly in middle-income countries, countries will need to mobilize additional resources to fill the funding gap. A recent analysis in 12 countries estimates that UMICs may have the capacity to fully fund their HIV programs from 2014 to 2018, although LICs and LMICs will still need significant donor support—even under a “maximum effort” scenario where (1) gross domestic product grows according to macroeconomic predictions, (2) countries increase health expenditure as per the Abuja target of 15% of gross domestic product, and (3) national budget allocations for HIV are 50% higher than HIV’s share of disease burden [15]. We included very ambitious domestic resource mobilization targets in our analysis based on the best available data on countries’ potential DCs to the HIV response. However, gaps still remain. Because of constrained resources for ART among external funders and among the governments of LICs and LMICs, efficiency gains through treatment optimization and the introduction of new low-cost regimens could be the most promising initiatives for reducing global ART costs and the funding gap.

Our study has several limitations. Our cost estimates exclude costs above the facility level, such as implementation of partner overhead and trainings, and costs of other services that may be integrated with ART provision, such as treatment of opportunistic infections, psychological counseling, or nutritional support. Additionally, the GPRM ARV prices are “ex works” prices that do not include the costs for in-country transportation or storage of ARVs, insurance, and taxes [34]. We are also limited by the availability and quality of country- and region-specific data. The epidemiological analysis in AIM assumes that country reports on the HIV epidemic and baseline numbers of people currently receiving treatment are accurate. Regional data were substituted for missing country-specific data when estimating coverage or the proportion of people receiving second-line treatment. We did not independently validate GPRM data on ARV prices with procurement agencies in countries or exclude any transactions in GPRM that could be potential outliers. However, an analysis of ARV prices using GPRM data found that the exclusion of outliers did not significantly impact the results [111]. Furthermore, not all HIV treatment formulations and regimens provided in countries were included in the cost analysis. Estimates of true country-specific DCs to commodity procurement and PEPFAR and Global Fund contributions to facility-level overhead and personnel are not reported to UNAIDS and hence are unavailable for most countries in our study. We assume PEPFAR and Global Fund contributions in the future will remain at current levels because of a lack of data on how these contributions will change. We estimated DCs for ART based on Global Fund CFTs and DCs to the entire HIV response. The DCs used in our analysis are ambitious and are not linked to an analysis of the future fiscal space for HIV financing in each country. We excluded any funding estimates that could not be disaggregated by country, such as regional ARV procurements. As a result, we could be underestimating the external financial resources available for ART.

Our analysis indicates that achieving the 90-90-90 treatment targets may be possible only through rapid expansions of ART eligibility to all people living with HIV as per the early-released WHO 2015 guidelines and increases in coverage; these targets are unlikely to be met under our current eligibility scenario or uniform application of aspects of the WHO 2013 guidelines. As the world moves to adopt the WHO 2015 guidelines, our analysis shows the need for increased financing for ART across both donors and governments [3,5]. While we have focused on additional funding needed for ART provision at the facility level, expanding ART eligibility and achieving the ambitious 90-90-90 targets globally will require overarching investments in the removal of policy barriers to scale-up, strong advocacy, necessary infrastructure and equipment, as well as other health-system-strengthening initiatives. Understanding these additional investments should be part of an urgent research agenda.

## Supporting Information

S1 Text Country inclusion criteria and methods for estimating annual numbers of people eligible for ART and on ART by country. (DOCX)

S2 Text Uncertainty analysis. (DOCX)

S3 Text Cost estimation. (DOCX)

S4 Text Estimating available financial resources. (DOCX)

S5 Text Data sources and availability. (DOCX)

## Abbreviations

ACT: Accelerating Children’s HIV/AIDS Treatment
AIM: AIDS Impact Model
ART: antiretroviral therapy
ARV: antiretroviral
CFT: counterpart financing thresholds
d4T: stavudine
DC: domestic contribution
EFV: efavirenz
EPP: Estimation Projection Package
Global Fund: Global Fund to Fight AIDS, Tuberculosis and Malaria
GPRM: Global Price Reporting Mechanism
HIC: high-income country
LIC: low-income country
LMIC: lower-middle-income country
NFM: new funding model
NVP: nevirapine
PEPFAR: U.S. President’s Emergency Plan for AIDS Relief
TDF: tenofovir
UMIC: upper-middle-income country
UNAIDS: Joint United Nations Programme on HIV/AIDS
WHO: World Health Organization
ZDV: zidovudine
