# Supplementary material for: The HIV Treatment Gap: Estimates of the Financial Resources Needed versus Available for Scale-Up of Antiretroviral Therapy in 97 Countries from 2015 to 2020
Source: PLoS Med. 2015 Nov 24;12(11):e1001907. doi: 10.1371/journal.pmed.1001907 (PMC4658189; doi:10.1371/journal.pmed.1001907)
Supplement: S1 Text — (DOCX) [file pmed.1001907.s001.docx]

**S1 Text. Country inclusion criteria and methods for estimating annual need for and number of people on ART by country**

1. Country inclusion

Of the *188 countries* that are listed as having HIV epidemics by UNAIDS, we excluded *42* countries that met any or all of the following criteria: insufficient PLHIV data, no official UNAIDS files (see below), or less than 1,000 PLHIV listed on their UNAIDS page or as per the AIDSInfo database [[1](#_ENREF_1)]: Seychelles, Cape Verde, Brunei, Micronesia, Fiji, Kiribati, Maldives, Marshall Islands, Nauru, Palau, Solomon Islands, Timor-Leste, Tonga, Vanuatu, Antigua and Barbuda, Dominica, Grenada, St. Kitts, St. Lucia, St. Vincent, Albania Bosnia, Turkmenistan, Croatia, Cyprus, Lithuania, Malta, Montenegro, Slovakia, Slovenia, Macedonia, Bahrain, Iraq, Jordan, Kuwait, Lebanon, Libya, Qatar, Saudi Arabia, Syria, and the United Arab Emirates.

We also excluded *31* high-income OECD countries as per the World Bank listing [[2](#_ENREF_2)]: Australia, Korea, Japan, New Zealand, Czech Republic, Denmark, Estonia, Hungary, Chile, Mexico, Austria, Belgium, Canada, Denmark, Finland, France, Germany, Greece, Iceland, Ireland, Israel, Italy, Luxembourg, Netherlands, Norway, Portugal, Spain, Sweden, Switzerland, the United Kingdom, and the United States of America.

We excluded an additional *15* countries which did not meet either of the criteria above, but for which we had inadequate data on critical parameters: South Korea, Singapore, Latvia, Poland, Turkey, Colombia, Costa Rica, Ecuador, El Salvador, Panama, Peru, Afghanistan, and Oman. Finally we excluded three Latin American countries: Brazil, Uruguay, and Venezuela where there is no indication of external financing of the HIV response. This selection process left 97 countries in the final sample (188 – 42 – 31 – 15 – 3 = 97).

1. Estimating future PLHIV and Need for ART using Spectrum

Spectrum is a suite of modular computer programs used to examine the effects of current demographic and epidemiological patterns for individual countries as well as various interventions in the health sector [[3](#_ENREF_3)]. Spectrum was originally developed by Futures Group with support from USAID and partners, and is modified, upgraded, and updated by Avenir Health. It can be downloaded freely online along with manuals and related datasets [[4](#_ENREF_4)]. The AIDS Impact Model, or AIM, is one of the modules in the Spectrum suite. AIM utilizes the Estimation Projection Package (EPP) algorithms to generate country-specific estimates of HIV prevalence and incidence trends, based on HIV sentinel surveillance site and population-level survey data. The EPP methodology and its integration into Spectrum has been described fully elsewhere [[5](#_ENREF_5)]. Some of the underlying raw data used in Spectrum projections is available upon request (see <http://alpha.lshtm.ac.uk/data/>). It is important to note that EPP-based estimates of the long-term trend in the HIV epidemic in a country, which is additionally modified within AIM to yield different estimates of interest, is via a country government-led process and represent the official estimates for most HIV-affected countries in the world. Sub-national estimates are also often developed. The EPP/AIM process is conducted annually in most countries, and forms the basis of the HIV epidemic characteristics reported to UNAIDS.

In AIM, the effects of future trends in the HIV epidemic, as developed using EPP, can be seen in the resulting numbers by age and sex of people living with HIV and new HIV infections. Other annual results include AIDS deaths, AIDS-related orphans, the annual need for antiretroviral treatment (ART) among adults and children, as well as for pediatric Cotrimoxazole prophylaxis. The impact of ART on reductions in AIDS-related mortality is incorporated in the model as it processes the national ART patient cohort from year to year (see Figure A, at end). AIM also generates other epidemiological outputs related to the HIV epidemic in a country that can be used for research.

The major inputs and outputs of AIM are shown in Figure A. Projections begin with an estimate of adult incidence, which is used to update HIV prevalence, given the AIDS deaths. AIDS deaths are related to the natural history of the HIV disease, untreated, as well as the reduction in mortality with treatment. Rates of mother-to child transmission provide the basis for estimations of new infant infections. These pediatric infections are kept track in the model and are aged to adult infections at an appropriate stage. Underlying demographic data, include age and sex splits, are supplied via the DemProj module within Spectrum, which uses UN Population Division ‘mid-variant’ estimates, which can be further edited/modified in-country to reflect superior Census data. With the demographic projection, the future projections of HIV-positive population, as well as the need for ART given eligibility norms edited within AIM also reflect growth in population, ageing, and change in the gender ratio, etc.

*Spectrum data files*

Official Spectrum data files with the AIM module updated are the result of country-led estimation exercises and are subject to restricted access for research. We obtained 81 of the 97 files required for our analysis via a request to the UNAIDS via its website. The remaining 16 countries are not included in the Spectrum files available from UNAIDS [[6](#_ENREF_6)]. For 15 countries we used the Country Data Package, which contains demographic and HIV epidemiological data for enabling Spectrum and AIM analysis, including all countries with HIV epidemics as reported to UNAIDS. While the resulting data files are not equivalent to official national files, they do have updated population estimates. The Country Data Package used for this analysis was updated to December 2013, and is the latest available to date [[7](#_ENREF_7)]. For India, in addition to the AIM projection file generated from the Country Data Package, we incorporated information on uncertainty bounds on the national people living with HIV (PLHIV) and need for ART from the official Government of India technical report on HIV estimates, which is updated up to 2011 [[8](#_ENREF_8)]. Similarly, we also reviewed national government HIV estimates reports for Kenya, Tanzania, and Ghana.

Official national Spectrum files contain information on national guidelines for adult and pediatric ART initiation, in the form of parameters by year of implementation selected across items such as the CD4 T-cell count threshold used for initiating treatment, age of children below which initiation is allowed regardless of CD4 counts, and data on whether other aspects of WHO 2013 treatment guidelines are followed, and if so from which year (see Table A).

*Preparation of Spectrum data files for analysis*

Many official national Spectrum data files have assumptions on future numbers of adults and pediatric patients on ART included, from 2014 up to 2020 or earlier years. These indicate a certain path for increased coverage. Where available, we retained these figures. When missing, we calculated high future ART coverage (%) rates up to 2020 among adults and children in AIM separately and entered this in AIM program statistics. This initializes AIM and allows it to incorporate effects from reduced mortality among PLHIV living with HIV as it calculates future populations living with HIV, eligibility for ART, etc., given different assumptions on eligibility corresponding to scenarios in our analysis. These percentage coverage assumptions are not used in the main model estimated for reporting future numbers of PLHIV or numbers eligible for treatment. These were entered to ensure that future eligibility read from AIM would be under sustained ART coverage increase, which has commensurate effects in the model on survival and transition between CD4 T-cell stages.

**Table A. Treatment eligibility based on WHO 2010 and 2013 guidelines**

| **Population** | **WHO 2010 Guidelines** | **WHO 2013 Guidelines** | **WHO 2013 as modeled in current analysis** | **WHO 2015 Guidelines*** |
| --- | --- | --- | --- | --- |
| Adults & adolescents  ≥ 14 years old | Initiate if CD4 < 350 cells/mm^3^ | Initiate if CD4 < 500 cells/mm^3^ | Initiate if CD4 < 500 cells/mm^3^ | Initiate if any CD4 count. As a priority, initiate if severe or advanced HIV  clinical disease (WHO clinical stage 3 or  4) and CD4 count ≤350  cells/mm3 |
|  | Initiate regardless of CD4 count or WHO stage if active TB disease or Hepatitis B co-infection | Initiate regardless of CD4 count or WHO stage if:   - active TB disease, - *Hepatitis B co-infection,* - pregnant & breastfeeding in women with HIV, or - *HIV+ individual in a serodiscordant partnership* | Initiate regardless of CD4 count or WHO stage if:   - active TB disease *[from 2011]* - pregnant & breastfeeding in women with HIV *[from 2013]* |  |
|  | Initiate if WHO stage 3 or 4 if pregnant or breastfeeding in women with HIV |  |  |  |
| Children ≥ 5 years old | Initiate if WHO stage 3 or 4, active TB disease, or CD4 < 350 cells/mm^3^ | Initiate if WHO stage 3 or 4, active TB disease, or CD4 <500 cells/mm^3^ | Initiate if WHO stage 3 or 4, active TB disease, or CD4 <500 cells/mm^3^ | Initiate if any CD4 count. As a priority, initiate  those with  WHO clinical stage 3 or  4 and individuals with CD4 count ≤350 cells/  mm3. |
| Children 2–5 years old | Initiate if CD4 < 750 cells/mm^3^ or <25% CD4 | Initiate ART in all regardless of WHO clinical stage and CD4 cell count | Initiate ART in all regardless of WHO clinical stage and CD4 cell count | Initiate if any CD4 count. As a priority, initiate  those with  WHO clinical stage 3 or  4 and individuals with CD4% <25% |
| Infants <1–2 year(s) old | Initiate ART in all regardless of WHO clinical stage and CD4 cell count | Initiate ART in all regardless of WHO clinical stage and CD4 cell count | Initiate ART in all regardless of WHO clinical stage and CD4 cell count | Initiate if any CD4 count |

**Note: The WHO defines adolescents as people ages 10-19 in the 2015 guidelines.*

We then generated ranges of PLHIV needing ART based on two of the three model scenarios; first, “*current eligibility*”: maintaining official eligibility criteria as set in the national Spectrum file, from 2014 through 2020. We note that many countries had expansive current eligibility criteria, including offering ART to PLHIV in all serodiscordant partnerships, key populations (men who have sex with men, sex workers). Examples of such include Zimbabwe.

Second, “*WHO 2013*”, which is based on certain aspects of the WHO 2013 guidelines and was harmonized to begin most changes from 2014 across all countries. Under the current scenario eligibility criteria were not altered, except for the case of Tanzania where new guidelines released in 2015 now allow for universal “test and offer” of ART for pediatric patients. We amended the eligibility criterion in the Tanzania analysis to reflect this change from 2015 onwards. As shown in Table A, the modeled scenario reflects the eligibility guideline for initiating ART irrespective of CD4 T-cell count among pregnant women living with HIV, which was assumed to be uniformly initiated in year 2013 across countries; whereas TB/HIV co-infection and eligibility for ART was assumed to have been a norm since the WHO 2010 guidelines update, and hence was set as having begun from 2011 across countries. We made changes to pediatric eligibility as per the critical differences in the WHO treatment guidelines.

We also derived the estimate of adults and children living with HIV per year 2013-2020, which were used as the eligible population in the third “*90-90-90*” scenario. This eligibility scenario is in line with the WHO 2015 guidelines, which recommend initiation of ART at any CD4 count for all age groups. The numbers of people living with HIV in this scenario were derived from the Spectrum files left with their original settings as obtained from UNAIDS, or if utilizing the Country Data Package, then they maintained WHO 2010 guidelines in order to initialize Spectrum AIM.

*Uncertainty Ranges in Spectrum Outputs*

AIM also incorporates an uncertainty analysis tool that enables users to estimate the uncertainty associated with critical epidemiological outputs of the model. The requirement to conduct this analysis appropriately is that the official national Spectrum file with AIM data be available, which would include EPP data and related estimates. The uncertainty tool engages with the uncertainty analysis within EPP in order to estimate the overall uncertainty in estimates for adult and pediatric HIV epidemics in a country over time.

The Spectrum AIM uncertainty analysis estimates the plausible range of values associated with several output indicators. While countries and even UNAIDS have historically reported the median values from such uncertainty analysis and used them for setting national targets, the ranges around the mode/median can be quite large depending on the lack and/or imprecision in the underlying data used to construct Spectrum estimates. Spectrum results based on such medians include national HIV prevalence, number of people living with HIV, and the adult and pediatric population eligible for ART based on eligibility thresholds. Pediatric HIV prevalence in specific is further subject to uncertainty in Spectrum due to differing mother-to-child transmission rates and estimates of survival among HIV-positive infants.

In order to generate input ranges for the Monte Carlo simulation analysis for our model, described in the next section, we utilized output from Spectrum’s uncertainty analysis tool for AIM. Using this tool, we ran the Spectrum AIM projection for 250-1,000 trials, depending on assessment of the underlying imprecision of the country data and the width of the range from running just 250 trials. From this analysis we derived the median and the 95% confidence interval for eligibility for ART and PLHIV, which were tabulated by country, year, and scenario.

For the 16 countries for which we did not have official national Spectrum files (and hence lacked underlying EPP data), we generated Spectrum projection files using the Country Data Package, as discussed before. While the uncertainty analysis tool can be used with the data package files, the median of the resulting range does not have the same interpretation and cannot be used as-is. For each of the three scenarios, we ran 250 trials, and calculated the annual percentage difference from the median of the uncertainty analysis and the lower/upper bounds from the uncertainty analysis results in Spectrum. We assumed that the results for PLHIV and estimated need for ART as available from the projection file *prior* to uncertainty analysis, and as reported to UNAIDS, would be preferred as the mode. In order to establish usable bounds around this mode, we looked to the percentage bounds from the uncertainty analysis, and combined these sets of information.

Final data generated from these analyses, which are replicable for those with access to the official Spectrum AIM files and the Country Data Package, are reported in Tables C and D at the end of this document for all 97 countries included in this analysis. The median value under the current scenario reflects the need used to determine baseline coverage.

1. Estimating 2013 baseline coverage for adult and pediatric ART

To establish baseline treatment coverage as a percentage for all countries with official UNAIDS AIM files, we used numbers of men, women, and children receiving treatment and needing treatment recorded for 2013 in program statistics when possible. For the remaining countries without UNAIDS AIM files, treatment and need numbers were pulled from either the UNAIDS AIDSinfo database, from Spectrum’s country data package, or were estimated from historical ART totals found in the AIDSinfo database [[1](#_ENREF_1)]. Numbers found in the AIDSinfo database claim a source in the UNAIDS Global AIDS Response Progress Reporting (GARPR) documents, which are country specific numbers from official reports submitted by countries to the UNAIDS Secretariat annually or bi-annually. The AIDSinfo database provides the total number receiving ART, the total number of adults aged 15+ receiving ART, and the total number children ages 0-14, in the form of annual data starting in year 2004. Numbers receiving treatment are recorded as the value as of December 31, of each respective year, except for 2014 where the numbers of people receiving ART are as of June 30, 2014. We used the numbers of adults and children receiving ART as of December 31, 2013 to establish our baseline ART coverage. Where necessary we supplemented all information with reported figures in the UNAIDS Gap Report [[9](#_ENREF_9)].

For Bulgaria, China, Romania, Russia, Serbia, Bangladesh we used ART totals for 2014 from the AIDSinfo database as historical ART data was unavailable. For Cuba, Equatorial Guinea, Iran, Kazakhstan, Myanmar, Nicaragua, Moldova, and Uzbekistan, ART totals were pulled from program statistics using Spectrum’s country data package. For Comoros, the Philippines, and Suriname, 2013 ART totals were estimated from historical ART data in AIDSinfo. For all other countries we used the 2013 recorded ART totals in the UNAIDS AIM files. To determine total numbers of adults and children needing ART we used the value for the 2013 median need generated by the uncertainty tool in AIM when possible. Table E contains ART totals used to generate baseline coverage as well as the final baseline coverage percentages.

In our search for data to establish ART baseline coverage we came across multiple sources listing coverage as a percent [[10](#_ENREF_10)]. Coverage data points in these sources do not indicate how need was established which may be the main reason why our calculated coverage baseline differs. Coverage percentages from AIDSinfo applied WHO 2013 eligibility guidelines to develop 2013 need numbers which seem to underestimate coverage when compared to our baseline.

1. Baseline 1st and 2nd line total number on treatment, mortality on ART

Our future ART numbers by country and year also reflect the baseline numbers of adults and children on 1^st^ and 2^nd^ line treatment. In cases where country specific data was available in either 2014 or 2012 GARPR reports, these numbers were used. In most cases region-specific percentages of adults on 1^st^ line, adults on 2^nd^ line, children on 1^st^ line and children on 2^nd^ line treatments from the March 2014, WHO report titled “Antiretroviral Medicines in Low-and Middle-Income Countries: Forecasts of Global and Regional Demand for 2013-2016” were applied to the 2013 totals on ART [[11](#_ENREF_11)]. In some cases, the study team had access to country specific data which was used to develop country specific percentage splits of adults on 1^st^ line, adults on 2^nd^ line, children on 1^st^ line and children on 2^nd^ line treatments [[12](#_ENREF_12),[13](#_ENREF_13)].

Estimated rates of both adult and pediatric migration from 1^st^ to 2^nd^ line were also calculated annually using the projected number of adults and children on first and second line from 2014 through 2016 in the WHO report. Averages of the estimated projected annual rates of migration by region were individually compared against a global average to establish trends of either high or low migration. These trends coupled with data in the Stanford Drug Resistance Database sorted by country and region allowed us to make assumptions on the current state of migration rates, by region, from 1^st^ line to 2^nd^ line treatment [[14](#_ENREF_14)]. Table F contains the high, median, and low values used to predict migration to 2^nd^ line treatment by region.

*Mortality*: Mortality estimates for patients on ART were based on default values in Spectrum for the southern African, West African, Middle East/North African, Latin America/Caribbean, Eastern European, and Asian epidemics. Ranges were taken from Spectrum rates across five-year age bands and CD4 T-cell count categories, with differentiation by length on treatment. Using these, averages were used to generate Table B for patients with greater than 12 months on treatment, appropriate for use in our model to generate the ranges for uncertainty analysis for a parameter related to year-to-year mortality among patients on second line treatment only:

**Table B. ART patients’ annual probability of death**

| Region | Annual probability of death, patients >12 months on ART | |
| --- | --- | --- |
|  | *CD4 count 350 to 500 cells/mm^3^* | *CD4 count 200 to 350 cells/mm^3^* |
| AES | 0.6% | 0.7% |
| AWC | 0.1% | 0.2% |
| AP | 0.2% | 0.3% |
| LAC | 0.4% | 0.5% |
| EECA | 0.3% | 0.4% |
| MENA | 0.8% | 0.9% |

**Figure A. Diagram of the AIDS Impact Model (AIM) in Spectrum software**


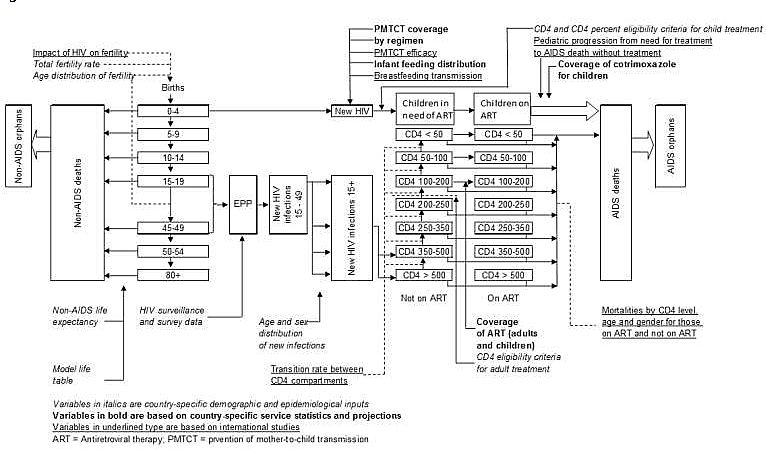


Source: [[15](#_ENREF_15)]

**Table C. Need for adult ART, based on results from Spectrum uncertainty analysis (in thousands)***“90-90-90” scenario results below reflect adult PLHIV*

| **Country** |  | **Thousands** | | | | | | | |
| --- | --- | --- | --- | --- | --- | --- | --- | --- | --- |
|  | **Scenario** | **2013** | **2014** | **2015** | **2016** | **2017** | **2018** | **2019** | **2020** |
|  | Current | 113.4 (99, 145) | 127.2 (109, 168) | 143.6 (114, 194) | 164.6 (124, 226) | 191.1 (141, 267) | 215.2 (155, 299) | 237.7 (168, 331) | 259.3 (180, 364) |
| **Angola** | WHO 2013 | 113.7 (99, 145) | 180.4 (135, 250) | 199.2 (147, 276) | 220.8 (160, 307) | 246.5 (176, 344) | 269.9 (188, 379) | 292.5 (200, 410) | 314.5 (213, 451) |
|  | 90-90-90 | 223.2 (160, 324) | 242.7 (173, 339) | 260.5 (186, 368) | 279.7 (197, 400) | 298.9 (206, 426) | 318.7 (218, 454) | 339.1 (227, 483) | 359.7 (237, 521) |
|  | Current | 225.8 (211, 240) | 278.1 (260, 294) | 287.8 (269, 303) | 296.5 (277, 313) | 304.2 (284, 321) | 311.1 (290, 328) | 316.8 (295, 334) | 321.5 (298, 340) |
| **Botswana** | WHO 2013 | 226.9 (221, 232) | 278.0 (264, 290) | 287.4 (272, 300) | 295.9 (280, 309) | 303.6 (286, 318) | 310.4 (292, 325) | 316.0 (296, 331) | 320.6 (300, 337) |
|  | 90-90-90 | 316.3 (297, 336) | 321.0 (302, 341) | 326.1 (305, 347) | 331.1 (309, 352) | 335.6 (311, 357) | 339.8 (315, 362) | 343.1 (317, 366) | 345.7 (319, 371) |
|  | Current | 49.0 (38, 74) | 55.1 (50, 77) | 54.6 (50, 76) | 54.5 (49, 75) | 54.7 (49, 75) | 55.2 (50, 76) | 56.0 (50, 75) | 57.3 (51, 76) |
| **Burundi** | WHO 2013 | 45.7 (44, 48) | 53.6 (49, 60) | 53.1 (49, 59) | 53.1 (49, 59) | 53.4 (49, 60) | 54.1 (49, 60) | 55.1 (50, 61) | 56.2 (51, 62) |
|  | 90-90-90 | 64.1 (57, 74) | 62.2 (56, 81) | 60.9 (54, 79) | 60.0 (53, 78) | 59.5 (52, 77) | 59.3 (52, 77) | 59.3 (52, 77) | 59.3 (52, 77) |
|  | Current | 2.5 (2, 3) | 5.2 (5, 6) | 9.6 (8, 10) | 15.4 (13, 15) | 22.2 (19, 22) | 29.4 (26, 29) | 36.7 (37, 43) | 43.6 (44, 51) |
| **Comoros** | WHO 2013 | 2.5 (2, 3) | 15.0 (14, 17) | 26.4 (24, 29) | 38.3 (34, 42) | 50.3 (40, 55) | 61.9 (49, 68) | 72.8 (57, 80) | 82.8 (65, 91) |
|  | 90-90-90 | 15.7 (14, 20) | 29.9 (27, 38) | 51.4 (46, 65) | 71.5 (64, 79) | 90.1 (81, 99) | 106.9 (84, 118) | 122.0 (96, 134) | 135.2 (106, 149) |
|  | Current | 10.5 (9, 17) | 12.7 (9, 19) | 13.2 (10, 19) | 13.5 (10, 19) | 13.8 (10, 20) | 14.1 (10, 20) | 14.4 (10, 20) | 14.6 (10, 20) |
| **Eritrea** | WHO 2013 | 10.5 (9, 17) | 12.7 (9, 19) | 13.2 (10, 19) | 13.5 (10, 19) | 13.8 (10, 20) | 14.1 (10, 20) | 14.4 (10, 20) | 14.6 (10, 21) |
|  | 90-90-90 | 14.3 (10, 21) | 14.4 (10, 21) | 14.5 (10, 21) | 14.7 (10, 21) | 14.8 (10, 21) | 14.9 (10, 21) | 15.0 (10, 22) | 15.1 (10, 22) |
|  | Current | 423.9 (338, 486) | 534.1 (436, 725) | 560.4 (460, 745) | 587.2 (484, 766) | 610.6 (506, 788) | 630.3 (524, 808) | 646.3 (536, 822) | 659.5 (547, 833) |
| **Ethiopia** | WHO 2013 | 434.7 (393, 482) | 529.9 (462, 606) | 555.1 (482, 639) | 580.3 (504, 672) | 601.9 (522, 697) | 620.0 (537, 716) | 634.8 (549, 732) | 647.1 (558, 745) |
|  | 90-90-90 | 622.4 (537, 727) | 627.7 (514, 781) | 640.2 (523, 794) | 652.4 (533, 807) | 663.2 (542, 819) | 672.2 (550, 830) | 679.6 (558, 839) | 685.9 (566, 846) |
|  | Current | 776.8 (707, 873) | 827.2 (755, 920) | 1,365.6 (1,272, 1,448) | 1,409.5 (1,313, 1,500) | 1,452.3 (1,354, 1,547) | 1,492.6 (1,391, 1,601) | 1,531.3 (1,427, 1,654) | 1,569.2 (1,450, 1,703) |
| **Kenya** | WHO 2013 | 799.7 (786, 819) | 1,169.6 (1,090, 1,248) | 1,213.6 (1,129, 1,301) | 1,254.8 (1,163, 1,349) | 1,296.9 (1,195, 1,399) | 1,339.2 (1,226, 1,449) | 1,381.7 (1,255, 1,496) | 1,424.7 (1,286, 1,548) |
|  | 90-90-90 | 1,452.0 (1,359, 1,558) | 1,499.7 (1,395, 1,611) | 1,543.8 (1,417, 1,668) | 1,583.2 (1,438, 1,725) | 1,623.4 (1,457, 1,779) | 1,661.3 (1,479, 1,829) | 1,697.5 (1,503, 1,876) | 1,732.9 (1,522, 1,917) |
|  | Current | 263.1 (254, 273) | 274.8 (264, 302) | 286.4 (274, 315) | 299.5 (285, 329) | 314.9 (299, 347) | 336.3 (317, 367) | 357.4 (335, 392) | 376.7 (351, 415) |
| **Lesotho** | WHO 2013 | 244.5 (233, 262) | 254.3 (241, 271) | 264.2 (250, 283) | 275.8 (261, 297) | 289.3 (274, 312) | 307.3 (290, 333) | 325.2 (307, 353) | 342.5 (323, 371) |
|  | 90-90-90 | 331.7 (313, 359) | 342.1 (321, 375) | 350.6 (329, 384) | 359.5 (337, 393) | 368.9 (346, 402) | 379.7 (354, 414) | 391.4 (364, 427) | 403.8 (372, 442) |
|  | Current | 18.7 (15, 25) | 37.6 (30, 56) | 36.5 (30, 54) | 35.5 (30, 53) | 34.6 (29, 52) | 33.9 (28, 52) | 33.5 (28, 50) | 33.4 (28, 50) |
| **Madagascar** | WHO 2013 | 18.9 (15, 26) | 31.3 (25, 46) | 30.4 (25, 45) | 29.5 (25, 43) | 28.9 (24, 42) | 28.4 (23, 43) | 28.1 (23, 42) | 28.2 (23, 42) |
|  | 90-90-90 | 46.2 (36, 69) | 44.6 (36, 66) | 43.3 (36, 65) | 42.2 (36, 64) | 41.1 (35, 62) | 40.2 (34, 62) | 39.6 (33, 60) | 39.2 (32, 59) |
|  | Current | 553.4 (530, 609) | 731.7 (680, 783) | 765.9 (713, 818) | 793.8 (738, 847) | 817.3 (757, 876) | 837.4 (773, 900) | 854.9 (786, 920) | 870.8 (796, 937) |
| **Malawi** | WHO 2013 | 562.1 (541, 612) | 737.6 (694, 785) | 774.2 (727, 823) | 803.2 (754, 852) | 827.3 (774, 875) | 847.7 (792, 898) | 865.5 (807, 914) | 881.4 (820, 931) |
|  | 90-90-90 | 884.3 (833, 940) | 889.6 (834, 943) | 900.6 (846, 953) | 910.2 (855, 963) | 919.3 (862, 976) | 927.8 (867, 986) | 936.0 (871, 998) | 944.2 (874, 1,007) |
|  | Current | 4.7 (5, 6) | 4.8 (5, 6) | 7.0 (6, 10) | 7.1 (6, 10) | 7.1 (6, 10) | 7.2 (6, 10) | 7.2 (7, 10) | 7.3 (7, 10) |
| **Mauritius** | WHO 2013 | 4.7 (5, 6) | 7.0 (6, 10) | 7.0 (6, 10) | 7.1 (6, 10) | 7.1 (6, 10) | 7.1 (6, 10) | 7.2 (6, 10) | 7.3 (7, 10) |
|  | 90-90-90 | 9.7 (9, 14) | 9.6 (9, 14) | 9.6 (9, 14) | 9.5 (9, 13) | 9.5 (9, 13) | 9.4 (8, 13) | 9.3 (8, 13) | 9.3 (8, 13) |
|  | Current | 652.4 (633, 678) | 788.8 (753, 835) | 858.5 (821, 906) | 937.9 (899, 986) | 1,021.6 (980, 1,072) | 1,122.1 (1,052, 1,210) | 1,228.9 (1,126, 1,352) | 1,327.5 (1,195, 1,485) |
| **Mozambique** | WHO 2013 | 728.3 (676, 780) | 1,051.6 (942, 1,177) | 1,107.4 (987, 1,242) | 1,168.3 (1,036, 1,312) | 1,232.9 (1,091, 1,393) | 1,332.2 (1,163, 1,525) | 1,433.9 (1,240, 1,656) | 1,529.8 (1,311, 1,787) |
|  | 90-90-90 | 1,327.4 (1,179, 1,513) | 1,382.7 (1,220, 1,581) | 1,442.4 (1,263, 1,653) | 1,504.2 (1,305, 1,733) | 1,567.9 (1,344, 1,824) | 1,635.3 (1,391, 1,916) | 1,707.6 (1,442, 2,010) | 1,782.7 (1,498, 2,109) |
|  | Current | 137.3 (121, 156) | 145.6 (120, 180) | 273.3 (221, 404) | 279.9 (221, 408) | 284.1 (223, 413) | 287.8 (227, 415) | 291.5 (231, 413) | 294.7 (236, 412) |
| **Namibia** | WHO 2013 | 138.3 (136, 142) | 207.9 (173, 250) | 223.7 (182, 294) | 250.1 (205, 382) | 255.8 (209, 376) | 260.1 (211, 374) | 264.3 (213, 372) | 268.4 (215, 372) |
|  | 90-90-90 | 223.3 (186, 276) | 154.5 (127, 192) | 276.5 (225, 409) | 282.4 (223, 409) | 286.4 (225, 415) | 290.2 (230, 416) | 293.7 (234, 415) | 296.9 (238, 414) |
|  | Current | 140.7 (133, 218) | 169.9 (156, 225) | 178.0 (163, 232) | 184.2 (169, 237) | 189.2 (173, 240) | 193.4 (176, 244) | 197.2 (180, 246) | 200.5 (182, 249) |
| **Rwanda** | WHO 2013 | 132.0 (126, 225) | 162.5 (150, 222) | 169.7 (156, 228) | 175.8 (162, 232) | 181.1 (166, 236) | 185.7 (170, 239) | 190.0 (174, 241) | 193.8 (177, 244) |
|  | 90-90-90 | 182.4 (167, 234) | 186.0 (169, 237) | 190.1 (172, 241) | 193.5 (176, 244) | 196.6 (178, 247) | 199.5 (181, 250) | 202.2 (183, 252) | 204.8 (184, 254) |
|  | Current | 3,101.3 (3,052, 3,172) | 3,446.5 (3,359, 3,569) | 3,841.7 (3,722, 3,997) | 4,218.0 (4,064, 4,410) | 4,564.0 (4,382, 4,764) | 4,884.9 (4,669, 5,107) | 5,185.3 (4,935, 5,420) | 5,468.8 (5,193, 5,717) |
| **South Africa** | WHO 2013 | 3,311.6 (3,247, 3,443) | 4,843.6 (4,577, 5,158) | 5,215.8 (4,930, 5,561) | 5,561.0 (5,256, 5,918) | 5,875.3 (5,547, 6,240) | 6,167.4 (5,817, 6,538) | 6,443.1 (6,077, 6,819) | 6,706.9 (6,321, 7,089) |
|  | 90-90-90 | 5,956.6 (5,607, 6,428) | 6,128.6 (5,779, 6,580) | 6,327.7 (5,968, 6,774) | 6,535.9 (6,162, 7,002) | 6,742.5 (6,353, 7,220) | 6,948.6 (6,538, 7,394) | 7,155.1 (6,718, 7,607) | 7,363.3 (6,901, 7,834) |
|  | Current | 61.6 (26, 158) | 98.6 (41, 252) | 101.6 (43, 255) | 103.8 (44, 257) | 105.7 (45, 261) | 107.5 (46, 263) | 109.6 (46, 265) | 112.5 (47, 274) |
| **South Sudan** | WHO 2013 | 69.8 (30, 182) | 103.3 (43, 264) | 106.4 (45, 267) | 108.6 (46, 270) | 110.5 (48, 273) | 112.1 (48, 274) | 114.2 (48, 276) | 117.1 (49, 286) |
|  | 90-90-90 | 134.7 (56, 350) | 139.7 (59, 355) | 142.6 (61, 358) | 144.0 (62, 359) | 144.8 (62, 358) | 145.1 (62, 355) | 145.9 (63, 355) | 147.6 (63, 360) |
|  | Current | 107.0 (106, 108) | 151.3 (146, 158) | 158.1 (152, 166) | 164.2 (158, 173) | 169.8 (162, 180) | 175.1 (167, 186) | 180.0 (172, 192) | 184.6 (176, 198) |
| **Swaziland** | WHO 2013 | 115.2 (114, 117) | 155.3 (150, 162) | 161.7 (156, 170) | 167.5 (161, 177) | 172.9 (165, 183) | 177.9 (170, 189) | 182.6 (173, 195) | 187.1 (177, 201) |
|  | 90-90-90 | 189.1 (182, 201) | 195.2 (187, 207) | 200.5 (191, 214) | 205.2 (195, 219) | 209.2 (198, 225) | 212.7 (200, 230) | 215.7 (201, 236) | 218.3 (201, 240) |
|  | Current | 631.0 (551, 1,225) | 749.4 (667, 1,219) | 827.8 (741, 1,256) | 898.5 (809, 1,299) | 959.7 (865, 1,341) | 1,011.6 (914, 1,380) | 1,055.6 (956, 1,413) | 1,092.9 (990, 1,427) |
| **Tanzania** | WHO 2013 | 636.5 (579, 718) | 977.2 (895, 1,076) | 1,045.1 (956, 1,150) | 1,104.4 (1,010, 1,212) | 1,155.8 (1,056, 1,268) | 1,199.7 (1,091, 1,318) | 1,237.4 (1,123, 1,360) | 1,269.9 (1,150, 1,398) |
|  | 90-90-90 | 1,192.6 (1,061, 1,890) | 1,214.1 (1,081, 1,876) | 1,241.6 (1,106, 1,869) | 1,269.3 (1,132, 1,864) | 1,295.0 (1,152, 1,857) | 1,316.9 (1,166, 1,844) | 1,336.2 (1,177, 1,826) | 1,353.3 (1,191, 1,794) |
|  | Current | 1,034.1 (959, 1,119) | 1,294.7 (1,203, 1,403) | 1,401.3 (1,307, 1,519) | 1,521.9 (1,413, 1,649) | 1,625.2 (1,497, 1,765) | 1,732.6 (1,575, 1,878) | 1,817.8 (1,635, 1,976) | 1,889.9 (1,688, 2,069) |
| **Uganda** | WHO 2013 | 778.7 (748, 839) | 1,120.8 (1,048, 1,213) | 1,215.4 (1,135, 1,311) | 1,331.5 (1,237, 1,431) | 1,435.5 (1,332, 1,547) | 1,537.2 (1,414, 1,653) | 1,628.0 (1,483, 1,744) | 1,709.7 (1,553, 1,846) |
|  | 90-90-90 | 1,370.9 (1,277, 1,483) | 1,444.3 (1,350, 1,562) | 1,534.9 (1,430, 1,662) | 1,622.5 (1,501, 1,762) | 1,705.1 (1,562, 1,853) | 1,784.8 (1,616, 1,937) | 1,856.9 (1,668, 2,023) | 1,921.4 (1,708, 2,106) |
|  | Current | 558.6 (552, 564) | 612.5 (605, 619) | 795.6 (750, 836) | 814.4 (766, 857) | 829.4 (776, 875) | 841.3 (781, 890) | 851.1 (794, 903) | 859.8 (796, 921) |
| **Zambia** | WHO 2013 | 563.4 (556, 572) | 747.7 (705, 784) | 788.3 (739, 830) | 806.8 (754, 851) | 822.2 (763, 869) | 835.1 (775, 886) | 846.5 (787, 900) | 856.9 (792, 921) |
|  | 90-90-90 | 913.6 (851, 969) | 932.2 (864, 988) | 949.4 (875, 1,005) | 963.8 (888, 1,022) | 975.3 (900, 1,037) | 984.6 (906, 1,054) | 992.2 (910, 1,069) | 999.2 (914, 1,090) |
|  | Current | 739.0 (701, 772) | 1,207.0  (1,134, 1,265) | 1,311.3  (1,237, 1,380) | 1,384.8  (1,303, 1,454) | 1,442.8  (1,346, 1,517) | 1,492.0  (1,390, 1,573) | 1,534.4  (1,417, 1,623) | 1,569.9  (1,443, 1,667) |
| **Zimbabwe** | WHO 2013 | 739.0 (701, 772) | 1,082.1  (1,014, 1,131) | 1,170.7  (1,100, 1,220) | 1,246.0  (1,174, 1,298) | 1,312.4  (1,237, 1,366) | 1,371.3 (1,293, 1,430) | 1,423.1  (1,344, 1,487) | 1,467.1  (1,382, 1,534) |
|  | 90-90-90 | 1,257.7  (1,179, 1,321) | 1,310.0  (1,229, 1,377) | 1,375.2  (1,295, 1,449) | 1,427.3  (1,332, 1,503) | 1,473.6  (1,372, 1,554) | 1,516.7  (1,404, 1,604) | 1,555.9  (1,436, 1,649) | 1,589.5  (1,461, 1,690) |
|  | Current | 3.8 (4, 4) | 4.1 (4, 4) | 4.4 (4, 4) | 4.8 (5, 5) | 5.3 (5, 6) | 6.0 (6, 6) | 6.8 (6, 7) | 7.7 (7, 8) |
| **Bangladesh** | WHO 2013 | 3.8 (4, 4) | 6.9 (7, 7) | 7.5 (7, 8) | 8.2 (8, 8) | 9.0 (9, 9) | 10.0 (9, 10) | 11.1 (10, 11) | 12.4 (12, 13) |
|  | 90-90-90 | 9.5 (9, 10) | 10.3 (10, 10) | 11.0 (11, 11) | 11.9 (12, 12) | 12.8 (12, 13) | 13.9 (13, 14) | 15.0 (14, 15) | 16.3 (16, 17) |
|  | Current | 0.2 (0, 0) | 0.2 (0, 0) | 0.3 (0, 1) | 0.3 (0, 1) | 0.4 (0, 1) | 0.4 (0, 1) | 0.5 (0, 1) | 0.5 (0, 2) |
| **Bhutan** | WHO 2013 | 0.2 (0, 0) | 0.4 (0, 1) | 0.5 (0, 2) | 0.6 (0, 2) | 0.6 (0, 3) | 0.7 (0, 4) | 0.8 (0, 4) | 0.8 (0, 5) |
|  | 90-90-90 | 0.6 (0, 2) | 0.6 (0, 2) | 0.7 (0, 2) | 0.8 (0, 3) | 0.8 (0, 4) | 0.9 (0, 5) | 1.0 (1, 6) | 1.0 (1, 7) |
|  | Current | 76.5 (31, 115) | 76.0 (31, 114) | 75.3 (31, 113) | 74.2 (31, 112) | 72.9 (30, 109) | 71.4 (29, 107) | 69.9 (28, 105) | 68.3 (27, 102) |
| **Cambodia** | WHO 2013 | 78.1 (41, 115) | 77.4 (41, 113) | 76.5 (40, 111) | 75.2 (40, 107) | 73.7 (39, 104) | 72.1 (38, 105) | 70.5 (35, 99) | 68.8 (33, 94) |
|  | 90-90-90 | 90.6 (37, 143) | 88.8 (37, 140) | 86.8 (36, 136) | 84.5 (36, 129) | 82.1 (34, 125) | 79.7 (33, 119) | 77.3 (31, 114) | 75.1 (30, 109) |
|  | Current | 389.1 (375, 399) | 404.5 (387, 418) | 441.2 (421, 456) | 494.9 (473, 512) | 565.7 (540, 585) | 650.2 (621, 673) | 728.8 (696, 756) | 794.7 (757, 824) |
| **China** | WHO 2013 | 389.1 (375, 399) | 588.0 (565, 602) | 638.0 (611, 655) | 699.4 (667, 719) | 773.2 (738, 795) | 855.4 (816, 881) | 930.0 (886, 958) | 992.7  (945, 1,022) |
|  | 90-90-90 | 737.3 (716, 747) | 770.4 (745, 783) | 809.2 (780, 824) | 853.6 (820, 871) | 903.3 (864, 924) | 957.0 (913, 981) | 1,012.0  (961, 1,040) | 1,066.8  (1,007, 1,099) |
|  | Current | 1,143.2  (971, 1,298) | 1,201.4  (1,021, 1,364) | 1,284.6  (1,091, 1,459) | 1,400.3  (1,190, 1,590) | 1,537.5  (1,306, 1,746) | 1,682.5  (1,429, 1,911) | 1,819.4  (1,546, 2,066) | 1,950.5  (1,657, 2,215) |
| **India** | WHO 2013 | 1,143.2  (971, 1,298) | 1,649.0  (1,401, 1,873) | 1,746.4  (1,484, 1,983) | 1,864.4  (1,584, 2,117) | 1,999.8  (1,699, 2,271) | 2,141.8  (1,819, 2,432) | 2,277.8  (1,935, 2,587) | 2,410.1  (2,047, 2,737) |
|  | 90-90-90 | 2,090.2  (1,752, 2,433) | 2,114.5  (1,773, 2,462) | 2,170.0  (1,819, 2,526) | 2,256.3  (1,892, 2,627) | 2,364.4  (1,982, 2,753) | 2,478.8  (2,078, 2,886) | 2,595.1  (2,176, 3,021) | 2,711.9  (2,274, 3,157) |
|  | Current | 183.8 (129, 282) | 206.4 (145, 310) | 229.9 (160, 346) | 251.0 (175, 382) | 270.4 (188, 411) | 289.3 (200, 444) | 308.0 (213, 471) | 326.9 (225, 496) |
| **Indonesia** | WHO 2013 | 218.3 (180, 275) | 428.9 (296, 631) | 465.6 (321, 705) | 500.8 (346, 761) | 536.0 (370, 815) | 572.0 (395, 874) | 609.4 (422, 936) | 648.2 (448, 996) |
|  | 90-90-90 | 617.0 (405, 893) | 668.7  (441, 1,011) | 723.2  (481, 1,126) | 777.2  (517, 1,211) | 831.9  (553, 1,297) | 888.0 (590, 1,386) | 946.2  (630, 1,481) | 1,006.9  (672, 1,579) |
|  | Current | 2.8 (2, 7) | 3.0 (2, 7) | 3.3 (2, 7) | 3.6 (2, 8) | 3.8 (3, 8) | 4.1 (3, 9) | 4.4 (3, 9) | 4.8 (3, 10) |
| **Laos** | WHO 2013 | 3.2 (3, 5) | 4.6 (3, 9) | 5.0 (4, 10) | 5.4 (4, 11) | 5.9 (4, 11) | 6.3 (4, 12) | 6.7 (4, 14) | 7.1 (5, 15) |
|  | 90-90-90 | 5.3 (4, 12) | 5.7 (4, 12) | 6.1 (4, 12) | 6.5 (4, 13) | 6.9 (4, 14) | 7.3 (4, 15) | 7.7 (5, 17) | 8.1 (5, 18) |
|  | Current | 39.3 (33, 59) | 41.8 (35, 64) | 45.0 (37, 69) | 49.0 (40, 75) | 53.8 (44, 82) | 59.6 (49, 91) | 66.1 (54, 100) | 72.7 (59, 109) |
| **Malaysia** | WHO 2013 | 39.5 (35, 105) | 65.1 (51, 123) | 70.1 (55, 130) | 75.5 (59, 137) | 81.4 (63, 142) | 87.8 (68, 149) | 94.4 (73, 156) | 100.9 (79, 163) |
|  | 90-90-90 | 87.1 (68, 154) | 90.5 (70, 151) | 94.1 (73, 149) | 98.1 (76, 149) | 102.7 (80, 155) | 107.9 (85, 163) | 113.5 (89, 169) | 119.1 (94, 174) |
|  | Current | 0.2 (0, 0) | 0.2 (0, 0) | 0.6 (0, 1) | 0.6 (0, 1) | 0.7 (0, 1) | 0.8 (1, 1) | 0.9 (1, 1) | 1.0 (1, 2) |
| **Mongolia** | WHO 2013 | 0.2 (0, 0) | 0.5 (0, 1) | 0.6 (0, 1) | 0.7 (0, 1) | 0.8 (0, 1) | 0.9 (1, 1) | 1.0 (1, 2) | 1.1 (1, 2) |
|  | 90-90-90 | 0.7 (0, 1) | 0.8 (1, 1) | 0.9 (1, 1) | 1.0 (1, 2) | 1.1 (1, 2) | 1.3 (1, 2) | 1.3 (1, 2) | 1.4 (1, 2) |
|  | Current | 103.8 (100, 107) | 104.1 (100, 107) | 108.4 (104, 112) | 116.3 (111, 120) | 124.7 (119, 129) | 133.0 (127, 137) | 138.0 (132, 142) | 141.1 (135, 146) |
| **Myanmar** | WHO 2013 | 103.8 (100, 107) | 136.1 (131, 139) | 139.3 (133, 143) | 144.7 (138, 149) | 149.9 (143, 154) | 155.1 (148, 159) | 157.2 (150, 162) | 158.3 (151, 163) |
|  | 90-90-90 | 167.6 (163, 170) | 164.3 (159, 167) | 162.3 (156, 165) | 161.3 (155, 165) | 161.1 (155, 165) | 161.2 (154, 165) | 161.3 (154, 166) | 161.3 (153, 166) |
|  | Current | 20.3 (17, 26) | 33.1 (25, 43) | 33.7 (25, 44) | 34.1 (25, 44) | 34.7 (27, 46) | 35.6 (28, 47) | 36.8 (29, 49) | 38.6 (30, 51) |
| **Nepal** | WHO 2013 | 20.3 (18, 26) | 28.6 (23, 39) | 29.0 (23, 39) | 29.4 (23, 39) | 30.0 (24, 40) | 30.9 (24, 41) | 32.0 (25, 42) | 33.4 (26, 44) |
|  | 90-90-90 | 37.1 (27, 47) | 37.1 (28, 48) | 37.1 (28, 48) | 37.2 (28, 49) | 37.5 (29, 50) | 38.0 (30, 51) | 38.8 (30, 52) | 39.6 (31, 53) |
|  | Current | 38.4 (24, 69) | 46.4 (28, 89) | 56.0 (33, 117) | 68.0 (39, 151) | 82.9 (45, 197) | 101.7 (52, 254) | 125.6 (60, 324) | 155.9 (70, 409) |
| **Pakistan** | WHO 2013 | 38.6 (23, 70) | 47.5 (28, 90) | 58.5 (34, 123) | 72.2 (39, 162) | 89.3 (47, 215) | 110.9 (54, 282) | 138.1 (62, 352) | 172.5 (73, 456) |
|  | 90-90-90 | 66.2 (39, 122) | 81.8 (47, 159) | 100.6 (56, 215) | 123.8 (67, 283) | 152.5 (78, 370) | 188.2 (89, 479) | 232.8 (104, 609) | 288.9 (122, 779) |
| **Papua New Guinea** | Current | 17.7 (14, 25) | 19.3 (16, 27) | 21.4 (17, 29) | 23.3 (19, 31) | 25.1 (20, 32) | 26.9 (22, 35) | 28.6 (23, 38) | 30.2 (24, 42) |
|  | WHO 2013 | 17.7 (17, 19) | 23.9 (20, 27) | 25.8 (22, 30) | 27.7 (23, 33) | 29.5 (24, 36) | 31.2 (25, 40) | 32.9 (27, 47) | 34.6 (28, 54) |
|  | 90-90-90 | 27.7 (23, 34) | 28.9 (24, 35) | 30.4 (25, 37) | 32.0 (26, 39) | 33.6 (27, 43) | 35.2 (28, 48) | 36.7 (29, 56) | 38.3 (30, 66) |
|  | Current | 7.0 (7, 8) | 8.0 (8, 9) | 10.8 (10, 12) | 16.2 (16, 18) | 18.8 (18, 21) | 21.6 (21, 23) | 24.1 (23, 26) | 26.3 (25, 28) |
| **Philippines** | WHO 2013 | 7.0 (7, 8) | 13.6 (13, 15) | 17.7 (17, 20) | 21.7 (21, 24) | 24.7 (24, 28) | 27.1 (26, 30) | 29.4 (28, 32) | 31.5 (30, 34) |
|  | 90-90-90 | 13.9 (14, 16) | 20.6 (20, 23) | 24.5 (24, 28) | 26.5 (25, 30) | 28.4 (27, 32) | 30.3 (29, 34) | 32.3 (30, 36) | 34.3 (32, 38) |
|  | Current | 0.9 (1, 3) | 2.3 (0, 4) | 2.7 (2, 6) | 3.0 (2, 6) | 3.4 (2, 7) | 3.7 (2, 8) | 4.0 (2, 8) | 4.3 (3, 9) |
| **Sri Lanka** | WHO 2013 | 1.0 (1, 2) | 2.0 (1, 4) | 2.3 (1, 5) | 2.7 (2, 5) | 3.0 (2, 5) | 3.3 (2, 6) | 3.6 (2, 7) | 3.9 (2, 8) |
|  | 90-90-90 | 2.7 (2, 6) | 3.2 (2, 7) | 3.6 (2, 8) | 4.0 (2, 9) | 4.5 (3, 10) | 4.9 (3, 11) | 5.3 (3, 11) | 5.6 (3, 12) |
|  | Current | 297.3 (253, 684) | 302.1 (256, 679) | 307.3 (257, 671) | 310.5 (260, 661) | 310.6 (260, 645) | 308.2 (259, 604) | 304.7 (256, 564) | 301.2 (253, 527) |
| **Thailand** | WHO 2013 | 308.8 (284, 328) | 359.2 (323, 385) | 357.2 (320, 383) | 353.5 (315, 379) | 348.0 (310, 373) | 341.4 (304, 367) | 334.8 (297, 361) | 328.9 (291, 355) |
|  | 90-90-90 | 427.9 (379, 560) | 421.5 (373, 552) | 416.6 (369, 545) | 411.1 (364, 539) | 403.4 (358, 523) | 394.1 (350, 503) | 384.8 (340, 484) | 376.2 (332, 464) |
|  | Current | 133.3 (129, 157) | 142.5 (137, 172) | 154.2 (146, 191) | 168.5 (157, 209) | 183.3 (168, 226) | 196.5 (179, 241) | 208.7 (190, 255) | 219.9 (199, 268) |
| **Viet Nam** | WHO 2013 | 135.1 (129, 177) | 194.5 (174, 257) | 206.3 (185, 267) | 219.7 (197, 283) | 233.6 (208, 298) | 246.5 (220, 317) | 258.7 (229, 327) | 270.2 (237, 348) |
|  | 90-90-90 | 244.6 (219, 317) | 246.8 (221, 314) | 250.8 (223, 312) | 257.1 (227, 317) | 264.5 (234, 330) | 272.2 (240, 344) | 280.0 (244, 360) | 287.8 (247, 376) |
|  | Current | 35.9 (33, 40) | 38.0 (35, 43) | 54.2 (50, 60) | 57.4 (52, 63) | 60.3 (55, 66) | 63.2 (57, 70) | 65.8 (60, 73) | 68.2 (61, 75) |
| **Benin** | WHO 2013 | 44.6 (26, 71) | 57.9 (47, 132) | 62.5 (53, 143) | 66.5 (58, 149) | 70.3 (62, 153) | 73.4 (65, 156) | 76.1 (68, 159) | 78.4 (70, 161) |
|  | 90-90-90 | 65.3 (61, 72) | 67.4 (63, 75) | 70.3 (65, 77) | 73.1 (67, 80) | 75.3 (69, 83) | 77.5 (71, 85) | 79.5 (73, 87) | 81.1 (74, 89) |
|  | Current | 83.2 (70, 99) | 85.3 (71, 101) | 88.0 (73, 103) | 92.0 (76, 108) | 95.7 (79, 113) | 99.3 (82, 117) | 103.0 (85, 122) | 106.0 (88, 126) |
| **Burkina Faso** | WHO 2013 | 76.2 (65, 90) | 78.3 (67, 92) | 80.7 (68, 95) | 84.3 (70, 99) | 88.1 (73, 104) | 91.6 (76, 108) | 95.0 (78, 113) | 98.0 (81, 117) |
|  | 90-90-90 | 95.4 (80, 113) | 96.9 (81, 114) | 98.1 (82, 115) | 99.9 (83, 117) | 102.0 (84, 119) | 104.3 (86, 123) | 106.6 (88, 126) | 108.8 (91, 130) |
|  | Current | 471.7 (435, 567) | 527.5 (483, 618) | 573.5 (521, 671) | 586.2 (530, 689) | 600.0 (543, 709) | 614.2 (553, 720) | 628.9 (564, 743) | 764.5 (681, 1,028) |
| **Cameroon** | WHO 2013 | 402.5 (375, 503) | 477.2 (437, 568) | 490.2 (447, 571) | 504.6 (459, 582) | 523.0 (474, 602) | 545.8 (494, 628) | 577.1 (519, 673) | 601.0 (539, 699) |
|  | 90-90-90 | 537.0 (494, 635) | 552.0 (504, 645) | 566.2 (516, 663) | 580.0 (524, 681) | 595.3 (539, 703) | 611.5 (551, 717) | 627.7 (563, 741) | 763.9 (681, 1,028) |
| **Central African Republic** | Current | 75.8 (62, 95) | 74.0 (61, 92) | 87.1 (72, 108) | 87.6 (72, 108) | 88.1 (73, 109) | 88.6 (73, 109) | 89.3 (73, 108) | 90.1 (75, 109) |
|  | WHO 2013 | 56.7 (46, 73) | 75.7 (62, 93) | 74.3 (61, 91) | 75.4 (62, 92) | 76.5 (63, 94) | 77.7 (64, 95) | 79.1 (65, 96) | 80.6 (66, 98) |
|  | 90-90-90 | 102.3 (84, 127) | 99.7 (82, 123) | 97.4 (80, 120) | 97.1 (80, 120) | 97.0 (79, 120) | 97.0 (79, 120) | 97.2 (79, 119) | 97.6 (80, 119) |
|  | Current | 132.2 (108, 160) | 131.4 (108, 160) | 133.2 (110, 163) | 136.4 (113, 168) | 140.5 (117, 174) | 145.1 (122, 181) | 150.9 (128, 188) | 157.3 (136, 195) |
| **Chad** | WHO 2013 | 132.2 (109, 160) | 131.4 (109, 160) | 133.2 (111, 164) | 136.5 (114, 169) | 140.7 (118, 175) | 145.3 (122, 181) | 151.1 (129, 188) | 157.5 (137, 196) |
|  | 90-90-90 | 173.5 (144, 209) | 170.4 (141, 206) | 169.4 (140, 207) | 169.6 (140, 209) | 170.5 (140, 210) | 171.5 (140, 214) | 172.6 (141, 218) | 173.7 (143, 222) |
|  | Current | 57.0 (38, 66) | 57.1 (39, 66) | 57.0 (39, 66) | 56.7 (40, 66) | 56.3 (40, 65) | 56.0 (40, 65) | 55.6 (40, 65) | 55.2 (40, 64) |
| **Congo** | WHO 2013 | 49.1 (34, 57) | 49.2 (34, 57) | 49.2 (35, 57) | 49.0 (35, 56) | 48.7 (35, 56) | 48.5 (35, 56) | 48.2 (35, 56) | 48.1 (35, 56) |
|  | 90-90-90 | 60.7 (40, 70) | 60.8 (41, 70) | 60.7 (42, 70) | 60.4 (42, 70) | 59.9 (42, 70) | 59.4 (42, 69) | 58.9 (42, 68) | 58.4 (42, 68) |
|  | Current | 190.5 (172, 211) | 194.4 (176, 214) | 211.8 (194, 232) | 223.3 (205, 243) | 232.7 (217, 250) | 244.0 (232, 259) | 250.1 (237, 265) | 254.1 (241, 269) |
| **Cote d'Ivoire** | WHO 2013 | 202.3 (183, 255) | 253.5 (224, 283) | 260.2 (230, 291) | 264.4 (233, 296) | 268.3 (236, 302) | 273.5 (240, 307) | 276.4 (243, 312) | 277.9 (244, 313) |
|  | 90-90-90 | 316.2 (277, 355) | 311.4 (274, 350) | 315.1 (278, 355) | 319.8 (281, 361) | 322.4 (282, 367) | 324.5 (282, 369) | 325.1 (282, 371) | 324.1 (281, 370) |
| **Democratic Republic of Congo** | Current | 167.1 (140, 204) | 323.8 (270, 386) | 373.6 (312, 444) | 395.1 (329, 464) | 417.0 (346, 489) | 434.9 (360, 510) | 453.0 (375, 534) | 465.4 (386, 552) |
|  | WHO 2013 | 209.1 (191, 240) | 301.0 (261, 356) | 320.5 (277, 382) | 342.1 (296, 411) | 367.1 (316, 441) | 388.5 (333, 466) | 408.7 (349, 491) | 425.2 (362, 512) |
|  | 90-90-90 | 376.4 (318, 445) | 387.7 (325, 458) | 404.8 (337, 475) | 421.2 (350, 496) | 435.6 (360, 513) | 448.9 (372, 529) | 460.7 (382, 544) | 470.6 (390, 559) |
|  | Current | 10.7 (9, 14) | 11.8 (10, 16) | 13.0 (11, 17) | 14.2 (12, 19) | 15.4 (13, 20) | 16.5 (14, 22) | 17.6 (15, 23) | 18.7 (15, 24) |
| **Equatorial Guinea** | WHO 2013 | 12.3 (12, 13) | 18.3 (16, 23) | 19.3 (16, 24) | 20.2 (17, 25) | 21.0 (17, 26) | 21.8 (18, 28) | 22.6 (18, 29) | 23.4 (19, 30) |
|  | 90-90-90 | 23.4 (20, 31) | 24.4 (20, 32) | 25.4 (21, 33) | 26.3 (21, 34) | 27.2 (21, 35) | 28.1 (22, 37) | 28.9 (22, 38) | 29.8 (22, 39) |
|  | Current | 33.7 (29, 42) | 35.0 (30, 43) | 35.7 (31, 44) | 36.2 (31, 44) | 36.5 (31, 44) | 36.5 (31, 44) | 36.5 (31, 44) | 36.3 (30, 44) |
| **Gabon** | WHO 2013 | 30.5 (26, 36) | 31.8 (27, 38) | 32.8 (28, 39) | 33.5 (29, 40) | 34.0 (29, 41) | 34.3 (29, 41) | 34.4 (29, 41) | 34.4 (29, 41) |
|  | 90-90-90 | 37.0 (31, 48) | 37.6 (32, 48) | 38.3 (32, 49) | 38.8 (32, 48) | 39.0 (33, 48) | 39.1 (32, 48) | 39.0 (32, 48) | 38.8 (32, 47) |
|  | Current | 10.0 (4, 14) | 10.3 (7, 22) | 10.6 (8, 23) | 10.9 (8, 23) | 11.4 (8, 24) | 12.0 (8, 24) | 12.5 (8, 25) | 13.0 (8, 26) |
| **Gambia** | WHO 2013 | 8.8 (7, 12) | 9.0 (7, 12) | 9.2 (7, 12) | 9.5 (7, 13) | 10.0 (7, 13) | 10.5 (7, 15) | 11.1 (8, 16) | 11.6 (8, 17) |
|  | 90-90-90 | 11.4 (8, 16) | 11.5 (8, 25) | 11.7 (8, 25) | 11.9 (8, 25) | 12.2 (8, 25) | 12.6 (8, 25) | 12.9 (8, 25) | 13.2 (9, 26) |
|  | Current | 105.9 (81, 138) | 120.9 (90, 165) | 163.3 (121, 231) | 170.9 (127, 243) | 177.1 (131, 252) | 182.3 (134, 258) | 186.6 (136, 263) | 190.1 (138, 269) |
| **Ghana** | WHO 2013 | 107.4 (105, 115) | 157.3 (126, 213) | 166.7 (132, 227) | 174.5 (137, 238) | 180.8 (141, 248) | 186.0 (143, 254) | 190.2 (146, 261) | 193.7 (148, 267) |
|  | 90-90-90 | 191.3 (142, 269) | 193.3 (142, 272) | 197.3 (145, 277) | 200.5 (147, 285) | 202.5 (148, 285) | 204.1 (148, 288) | 205.4 (148, 291) | 206.2 (149, 294) |
|  | Current | 49.9 (36, 76) | 107.1 (88, 130) | 114.8 (94, 138) | 123.0 (101, 148) | 131.7 (108, 159) | 138.2 (113, 166) | 143.8 (117, 173) | 149.2 (120, 179) |
| **Guinea** | WHO 2013 | 54.2 (50, 64) | 90.9 (75, 110) | 98.1 (81, 117) | 105.9 (87, 127) | 114.6 (94, 136) | 122.1 (100, 145) | 128.9 (106, 154) | 135.2 (110, 162) |
|  | 90-90-90 | 112.8 (93, 137) | 119.4 (98, 145) | 125.2 (103, 150) | 130.4 (106, 157) | 135.8 (111, 164) | 141.2 (114, 170) | 146.4 (118, 176) | 151.7 (122, 182) |
| **Guinea-Bissau** | Current | 25.3 (23, 29) | 26.1 (23, 31) | 27.0 (24, 32) | 28.0 (25, 34) | 29.1 (25, 36) | 30.4 (26, 38) | 32.0 (28, 40) | 33.8 (29, 43) |
|  | WHO 2013 | 25.3 (23, 29) | 26.1 (23, 31) | 27.0 (24, 32) | 28.0 (25, 34) | 29.1 (25, 36) | 30.5 (26, 38) | 32.1 (28, 40) | 33.9 (29, 43) |
|  | 90-90-90 | 35.3 (32, 41) | 36.2 (32, 43) | 36.9 (33, 44) | 37.7 (33, 45) | 38.5 (34, 47) | 39.4 (34, 49) | 40.3 (35, 51) | 41.1 (35, 52) |
|  | Current | 19.4 (16, 24) | 19.0 (16, 24) | 19.1 (16, 24) | 18.7 (15, 24) | 18.4 (15, 23) | 18.1 (15, 23) | 17.9 (15, 23) | 17.8 (14, 22) |
| **Liberia** | WHO 2013 | 19.4 (16, 24) | 19.0 (15, 24) | 19.1 (15, 24) | 18.7 (15, 24) | 18.4 (15, 23) | 18.1 (15, 23) | 17.9 (14, 23) | 17.8 (14, 23) |
|  | 90-90-90 | 25.4 (21, 32) | 24.9 (20, 31) | 24.3 (20, 30) | 23.8 (19, 30) | 23.3 (19, 29) | 22.8 (19, 29) | 22.5 (18, 28) | 22.2 (18, 28) |
|  | Current | 66.4 (61, 82) | 73.9 (59, 174) | 74.5 (59, 173) | 75.5 (60, 173) | 77.4 (60, 173) | 79.6 (61, 174) | 81.8 (62, 174) | 83.4 (63, 175) |
| **Mali** | WHO 2013 | 52.0 (48, 70) | 65.7 (55, 99) | 66.0 (55, 99) | 67.0 (56, 101) | 68.9 (56, 104) | 71.3 (58, 108) | 73.5 (59, 109) | 75.4 (61, 114) |
|  | 90-90-90 | 81.9 (67, 138) | 81.6 (61, 352) | 81.3 (61, 346) | 81.2 (61, 343) | 81.7 (61, 342) | 82.5 (62, 350) | 83.5 (63, 354) | 84.4 (64, 359) |
|  | Current | 4.9 (4, 7) | 4.9 (4, 7) | 4.9 (4, 7) | 4.9 (4, 7) | 5.1 (4, 7) | 5.3 (4, 7) | 5.6 (4, 7) | 5.8 (5, 7) |
| **Mauritania** | WHO 2013 | 5.1 (4, 7) | 6.4 (5, 9) | 6.3 (5, 8) | 6.1 (5, 9) | 6.1 (5, 8) | 6.2 (5, 9) | 6.3 (5, 9) | 6.4 (5, 9) |
|  | 90-90-90 | 8.2 (6, 11) | 7.9 (6, 11) | 7.6 (6, 11) | 7.4 (5, 10) | 7.3 (5, 10) | 7.2 (5, 10) | 7.2 (5, 10) | 7.3 (5, 10) |
|  | Current | 19.8 (15, 36) | 20.2 (16, 35) | 20.8 (16, 35) | 21.7 (17, 35) | 22.7 (18, 36) | 23.7 (19, 37) | 24.7 (19, 38) | 25.5 (20, 37) |
| **Niger** | WHO 2013 | 22.2 (18, 32) | 27.6 (22, 35) | 28.4 (23, 36) | 29.1 (24, 37) | 29.9 (24, 38) | 30.7 (25, 39) | 31.4 (25, 40) | 32.1 (26, 41) |
|  | 90-90-90 | 32.6 (26, 42) | 31.6 (26, 41) | 31.1 (25, 40) | 31.0 (25, 39) | 31.1 (25, 40) | 31.4 (25, 40) | 31.7 (26, 40) | 32.0 (26, 41) |
|  | Current | 1,278.9  (994, 2,634) | 1,350.1  (1,063, 2,649) | 1,443.3  (1,147, 2,692) | 1,557.3  (1,259, 2,793) | 1,684.5  (1,376, 2,912) | 1,817.3  (1,503, 3,051) | 1,948.3  (1,638, 3,170) | 2,070.9  (1,755, 3,274) |
| **Nigeria** | WHO 2013 | 1,487.4  (1,328, 2,228) | 2,210.5  (1,972, 2,698) | 2,331.0  (2,086, 2,810) | 2,464.9  (2,212, 2,945) | 2,611.8  (2,342, 3,100) | 2,740.3  (2,454, 3,229) | 2,855.9  (2,550, 3,337) | 2,955.0  (2,637, 3,444) |
|  | 90-90-90 | 2,830.1  (2,544, 3,339) | 2,850.5  (2,561, 3,329) | 2,871.6  (2,580, 3,339) | 2,901.9  (2,604, 3,353) | 2,945.4  (2,638, 3,383) | 2,996.4  (2,677, 3,442) | 3,048.7  (2,721, 3,498) | 3,096.1  (2,758, 3,577) |
|  | Current | 22.7 (12, 36) | 28.5 (13, 41) | 28.9 (13, 42) | 29.5 (14, 42) | 30.3 (15, 43) | 31.2 (15, 44) | 32.3 (15, 44) | 33.2 (16, 45) |
| **Senegal** | WHO 2013 | 22.8 (14, 36) | 28.0 (13, 41) | 28.4 (14, 41) | 28.9 (14, 42) | 29.7 (15, 43) | 30.6 (15, 44) | 31.6 (16, 43) | 32.5 (16, 44) |
|  | 90-90-90 | 33.1 (14, 46) | 33.2 (14, 46) | 33.4 (14, 46) | 33.5 (15, 46) | 33.9 (15, 46) | 34.3 (15, 46) | 34.8 (16, 47) | 35.2 (16, 48) |
|  | Current | 22.7 (19, 32) | 23.7 (20, 33) | 24.4 (20, 33) | 25.3 (21, 33) | 26.3 (22, 34) | 27.4 (23, 36) | 28.6 (23, 37) | 29.9 (24, 39) |
| **Sierra Leone** | WHO 2013 | 28.0 (25, 33) | 39.5 (33, 50) | 40.0 (32, 51) | 40.5 (32, 53) | 41.2 (32, 56) | 42.0 (32, 60) | 42.9 (32, 64) | 43.9 (32, 68) |
|  | 90-90-90 | 52.0 (42, 66) | 52.6 (42, 67) | 53.1 (41, 69) | 53.7 (41, 73) | 54.5 (41, 77) | 55.5 (41, 82) | 56.6 (41, 87) | 57.8 (41, 93) |
|  | Current | 73.4 (50, 135) | 75.1 (51, 137) | 86.9 (55, 160) | 90.4 (56, 162) | 94.8 (58, 169) | 98.0 (60, 174) | 101.1 (61, 178) | 104.1 (63, 184) |
| **Togo** | WHO 2013 | 58.8 (46, 101) | 79.1 (49, 145) | 79.2 (48, 144) | 81.8 (49, 147) | 85.4 (50, 153) | 88.6 (52, 157) | 91.7 (54, 162) | 94.6 (55, 166) |
|  | 90-90-90 | 102.2 (56, 183) | 101.5 (61, 182) | 100.3 (61, 181) | 101.0 (61, 181) | 102.1 (62, 181) | 103.5 (63, 183) | 104.9 (64, 185) | 106.4 (64, 188) |
|  | Current | 1.3 (1, 3) | 1.5 (1, 3) | 1.6 (1, 3) | 1.8 (1, 4) | 2.0 (1, 4) | 2.3 (1, 4) | 2.5 (2, 5) | 2.9 (2, 5) |
| **Armenia** | WHO 2013 | 1.4 (1, 2) | 2.6 (2, 5) | 2.9 (2, 5) | 3.2 (2, 5) | 3.5 (2, 6) | 4.0 (3, 6) | 4.4 (3, 8) | 5.0 (3, 9) |
|  | 90-90-90 | 3.6 (2, 8) | 4.0 (3, 7) | 4.4 (3, 8) | 4.9 (3, 8) | 5.4 (3, 9) | 6.1 (4, 10) | 6.9 (4, 12) | 7.7 (5, 14) |
|  | Current | 5.8 (5, 8) | 6.6 (5, 8) | 7.3 (6, 9) | 7.9 (6, 10) | 8.5 (7, 11) | 9.1 (7, 12) | 9.8 (8, 13) | 10.4 (8, 13) |
| **Azerbaijan** | WHO 2013 | 3.4 (3, 4) | 6.6 (5, 8) | 7.3 (6, 9) | 8.0 (6, 10) | 8.6 (7, 11) | 9.3 (7, 12) | 9.9 (8, 13) | 10.6 (8, 14) |
|  | 90-90-90 | 9.0 (7, 12) | 10.0 (8, 13) | 11.0 (8, 14) | 11.9 (9, 15) | 12.9 (10, 17) | 13.8 (11, 18) | 14.8 (12, 19) | 15.9 (12, 21) |
|  | Current | 12.7 (12, 13) | 14.1 (13, 15) | 21.5 (20, 23) | 22.9 (21, 25) | 24.2 (22, 27) | 25.4 (23, 29) | 26.7 (24, 31) | 20.2 (18, 23) |
| **Belarus** | WHO 2013 | 10.3 (10, 11) | 18.6 (17, 20) | 20.0 (19, 21) | 21.3 (20, 23) | 22.5 (20, 25) | 23.7 (21, 27) | 24.8 (22, 29) | 16.9 (16, 18) |
|  | 90-90-90 | 25.8 (24, 28) | 27.8 (26, 30) | 29.7 (27, 32) | 31.4 (28, 34) | 33.0 (30, 37) | 34.5 (31, 40) | 36.2 (32, 43) | 37.9 (33, 47) |
|  | Current | 0.9 (1, 1) | 1.0 (1, 1) | 1.2 (1, 1) | 1.4 (1, 1) | 1.6 (2, 2) | 1.9 (2, 2) | 2.1 (2, 2) | 2.3 (2, 2) |
| **Bulgaria** | WHO 2013 | 0.9 (1, 1) | 1.6 (2, 2) | 1.8 (2, 2) | 2.0 (2, 2) | 2.2 (2, 2) | 2.5 (2, 3) | 2.7 (3, 3) | 2.9 (3, 3) |
|  | 90-90-90 | 2.0 (2, 2) | 2.1 (2, 2) | 2.3 (2, 3) | 2.5 (2, 3) | 2.6 (3, 3) | 2.8 (3, 3) | 3.0 (3, 3) | 3.2 (3, 3) |
|  | Current | 2.5 (2, 3) | 2.9 (2, 4) | 3.3 (3, 4) | 3.7 (3, 5) | 4.0 (3, 5) | 4.4 (3, 6) | 4.8 (4, 6) | 5.2 (4, 7) |
| **Georgia** | WHO 2013 | 2.7 (3, 3) | 4.7 (4, 6) | 5.1 (4, 6) | 5.5 (4, 7) | 5.9 (5, 8) | 6.3 (5, 8) | 6.7 (5, 9) | 7.0 (5, 10) |
|  | 90-90-90 | 6.2 (5, 8) | 6.8 (5, 9) | 7.3 (6, 10) | 7.7 (6, 11) | 8.1 (6, 12) | 8.5 (6, 12) | 8.9 (6, 13) | 9.3 (6, 14) |
|  | Current | 6.1 (6, 6) | 6.0 (6, 6) | 5.9 (6, 6) | 5.9 (6, 6) | 5.9 (6, 6) | 6.1 (6, 6) | 6.4 (6, 7) | 6.5 (6, 7) |
| **Kazakhstan** | WHO 2013 | 6.1 (6, 6) | 7.8 (7, 8) | 7.6 (7, 8) | 7.5 (7, 8) | 7.4 (7, 8) | 7.5 (7, 8) | 7.5 (7, 8) | 7.6 (7, 8) |
|  | 90-90-90 | 8.5 (8, 9) | 9.1 (9, 9) | 9.7 (9, 10) | 10.3 (10, 10) | 11.0 (11, 11) | 11.7 (11, 12) | 12.4 (12, 13) | 13.1 (12, 13) |
|  | Current | 2.5 (2, 3) | 2.9 (3, 3) | 3.3 (3, 4) | 3.7 (3, 4) | 4.1 (4, 4) | 4.5 (4, 5) | 4.9 (4, 5) | 5.2 (5, 6) |
| **Kyrgyzstan** | WHO 2013 | 2.7 (2, 3) | 5.6 (4, 7) | 6.1 (5, 7) | 6.6 (5, 8) | 7.1 (6, 9) | 7.6 (6, 10) | 8.1 (6, 10) | 8.5 (6, 11) |
|  | 90-90-90 | 8.2 (7, 10) | 8.9 (7, 11) | 9.6 (8, 12) | 10.3 (8, 13) | 11.0 (8, 14) | 11.7 (9, 15) | 12.3 (9, 17) | 12.9 (9, 17) |
| **Republic of Moldova** | Current | 6.1 (6, 6) | 6.3 (6, 6) | 6.5 (6, 7) | 6.9 (7, 7) | 7.6 (7, 8) | 8.5 (8, 9) | 9.6 (9, 10) | 10.8 (10, 11) |
|  | WHO 2013 | 6.1 (6, 6) | 9.4 (9, 10) | 10.0 (10, 10) | 10.6 (10, 11) | 11.4 (11, 12) | 12.4 (12, 13) | 13.5 (13, 14) | 14.7 (14, 15) |
|  | 90-90-90 | 12.6 (12, 13) | 13.0 (13, 13) | 13.4 (13, 14) | 13.9 (13, 14) | 14.5 (14, 15) | 15.1 (14, 15) | 15.9 (15, 16) | 16.7 (16, 17) |
|  | Current | 11.8 (11, 12) | 12.0 (12, 12) | 13.0 (13, 13) | 14.1 (14, 15) | 14.9 (14, 15) | 15.4 (15, 16) | 15.9 (15, 16) | 16.3 (16, 17) |
| **Romania** | WHO 2013 | 11.8 (11, 12) | 14.5 (14, 15) | 15.4 (15, 16) | 16.2 (16, 17) | 16.8 (16, 17) | 17.3 (16, 18) | 17.7 (17, 18) | 18.1 (17, 19) |
|  | 90-90-90 | 16.6 (16, 17) | 16.6 (16, 17) | 16.9 (16, 17) | 17.2 (17, 18) | 17.6 (17, 18) | 17.9 (17, 19) | 18.3 (17, 19) | 18.7 (18, 19) |
|  | Current | 381.2 (369, 390) | 396.9 (382, 408) | 411.0 (393, 424) | 440.1 (420, 456) | 487.7 (465, 506) | 554.6 (529, 576) | 648.5 (619, 674) | 734.6 (700, 763) |
| **Russia** | WHO 2013 | 381.2 (375, 390) | 579.5 (562, 591) | 621.9 (600, 636) | 673.3 (649, 692) | 735.6 (708, 756) | 812.1 (780, 836) | 909.6 (873, 937) | 994.7 (952, 1,025) |
|  | 90-90-90 | 761.8 (744, 771) | 801.1 (779, 812) | 842.3 (816, 855) | 888.2 (857, 903) | 939.5 (903, 957) | 996.6 (956, 1,017) | 1,059.0 (1,012, 1,083) | 1,124.3 (1,069, 1,152) |
|  | Current | 1.8 (2, 2) | 1.8 (2, 2) | 1.9 (2, 2) | 2.1 (2, 2) | 2.4 (2, 2) | 2.6 (3, 3) | 2.8 (3, 3) | 3.0 (3, 3) |
| **Serbia** | WHO 2013 | 1.8 (2, 2) | 2.4 (2, 2) | 2.6 (2, 3) | 2.8 (3, 3) | 3.0 (3, 3) | 3.2 (3, 3) | 3.4 (3, 3) | 3.5 (3, 4) |
|  | 90-90-90 | 3.0 (3, 3) | 3.0 (3, 3) | 3.1 (3, 3) | 3.2 (3, 3) | 3.3 (3, 3) | 3.5 (3, 4) | 3.6 (3, 4) | 3.7 (4, 4) |
|  | Current | 8.7 (6, 18) | 9.2 (7, 18) | 9.6 (7, 18) | 10.1 (7, 18) | 10.7 (7, 18) | 11.2 (8, 19) | 11.7 (8, 20) | 12.2 (8, 20) |
| **Tajikistan** | WHO 2013 | 5.0 (4, 9) | 8.9 (7, 18) | 9.4 (7, 18) | 9.9 (7, 18) | 10.4 (7, 18) | 10.9 (8, 19) | 11.4 (8, 20) | 11.9 (8, 20) |
|  | 90-90-90 | 12.9 (9, 28) | 13.5 (10, 27) | 14.1 (10, 27) | 14.8 (10, 27) | 15.4 (10, 28) | 16.1 (11, 28) | 16.8 (11, 29) | 17.5 (11, 30) |
|  | Current | 103.0 (87, 120) | 107.5 (91, 126) | 112.2 (96, 130) | 118.2 (101, 136) | 125.1 (107, 144) | 134.2 (116, 154) | 143.3 (124, 165) | 151.6 (132, 176) |
| **Ukraine** | WHO 2013 | 161.1 (143, 188) | 187.8 (165, 217) | 186.0 (163, 215) | 185.8 (163, 215) | 186.6 (164, 218) | 202.9 (177, 239) | 212.1 (184, 249) | 218.9 (189, 260) |
|  | 90-90-90 | 218.4 (190, 251) | 215.3 (186, 249) | 213.1 (184, 248) | 212.5 (184, 249) | 213.2 (184, 251) | 216.4 (187, 254) | 220.0 (189, 258) | 223.6 (191, 264) |
|  | Current | 18.2 (17, 19) | 17.8 (16, 19) | 17.5 (16, 18) | 17.3 (16, 18) | 17.6 (16, 19) | 18.4 (17, 20) | 19.1 (17, 20) | 19.9 (18, 21) |
| **Uzbekistan** | WHO 2013 | 18.2 (15, 19) | 25.4 (19, 26) | 24.8 (19, 26) | 24.4 (19, 25) | 24.4 (19, 25) | 24.9 (19, 26) | 25.3 (19, 26) | 25.9 (20, 27) |
|  | 90-90-90 | 35.4 (34, 36) | 33.8 (32, 35) | 32.3 (31, 33) | 31.2 (29, 32) | 30.4 (29, 31) | 30.0 (28, 31) | 29.9 (28, 31) | 30.0 (28, 31) |
|  | Current | 4.1 (4, 4) | 4.2 (4, 5) | 4.4 (4, 5) | 4.7 (4, 5) | 5.0 (5, 5) | 5.4 (5, 6) | 5.7 (5, 6) | 6.0 (5, 7) |
| **Bahamas** | WHO 2013 | 4.4 (4, 5) | 6.1 (6, 7) | 6.4 (6, 7) | 6.7 (6, 7) | 7.1 (7, 8) | 7.4 (7, 8) | 7.7 (7, 9) | 7.9 (7, 9) |
|  | 90-90-90 | 7.7 (7, 8) | 7.7 (7, 8) | 7.7 (7, 8) | 7.8 (7, 9) | 8.0 (7, 9) | 8.1 (8, 9) | 8.2 (8, 9) | 8.3 (8, 9) |
|  | Current | 1.8 (1, 2) | 1.8 (1, 2) | 1.9 (1, 3) | 1.9 (1, 3) | 1.9 (1, 3) | 1.9 (1, 3) | 2.0 (1, 3) | 2.0 (1, 3) |
| **Barbados** | WHO 2013 | 1.8 (1, 2) | 2.1 (2, 3) | 2.1 (2, 3) | 2.1 (2, 3) | 2.2 (2, 3) | 2.2 (2, 3) | 2.2 (2, 3) | 2.2 (2, 3) |
|  | 90-90-90 | 2.4 (2, 3) | 2.4 (2, 3) | 2.4 (2, 3) | 2.4 (2, 3) | 2.4 (2, 3) | 2.4 (2, 3) | 2.4 (2, 4) | 2.4 (2, 4) |
|  | Current | 2.7 (2, 5) | 2.8 (2, 5) | 2.9 (3, 6) | 3.1 (3, 6) | 3.3 (3, 7) | 3.5 (3, 7) | 3.6 (3, 7) | 3.8 (3, 7) |
| **Belize** | WHO 2013 | 2.4 (2, 3) | 2.4 (2, 3) | 2.5 (2, 3) | 2.7 (2, 3) | 2.8 (3, 4) | 3.0 (3, 4) | 3.2 (3, 4) | 3.3 (3, 4) |
|  | 90-90-90 | 3.0 (3, 7) | 3.1 (3, 7) | 3.2 (3, 7) | 3.3 (3, 7) | 3.5 (3, 7) | 3.6 (3, 7) | 3.7 (3, 7) | 3.9 (3, 8) |
|  | Current | 6.8 (5, 41) | 7.0 (5, 43) | 7.3 (5, 45) | 7.5 (5, 47) | 7.8 (5, 49) | 8.2 (6, 51) | 8.5 (6, 54) | 8.8 (6, 57) |
| **Bolivia** | WHO 2013 | 7.1 (5, 54) | 10.6 (7, 70) | 10.8 (7, 72) | 11.0 (7, 74) | 11.2 (7, 77) | 11.5 (7, 78) | 11.8 (7, 79) | 12.1 (7, 81) |
|  | 90-90-90 | 14.4 (10, 134) | 14.7 (10, 133) | 14.9 (10, 134) | 15.2 (10, 135) | 15.5 (9, 134) | 15.8 (9, 134) | 16.2 (9, 133) | 16.5 (9, 132) |
|  | Current | 14.6 (11, 71) | 16.9 (13, 104) | 18.2 (14, 105) | 19.4 (15, 107) | 20.6 (16, 108) | 22.0 (17, 110) | 23.4 (18, 111) | 24.8 (19, 113) |
| **Cuba** | WHO 2013 | 8.0 (7, 9) | 12.6 (11, 15) | 14.1 (12, 25) | 15.5 (13, 25) | 16.8 (14, 26) | 18.2 (15, 26) | 19.6 (16, 27) | 21.0 (17, 31) |
|  | 90-90-90 | 18.0 (13, 134) | 18.8 (14, 131) | 20.0 (15, 131) | 21.3 (16, 131) | 22.6 (17, 132) | 23.9 (18, 134) | 25.3 (18, 134) | 26.8 (19, 136) |
| **Dominican Republic** | Current | 28.9 (18, 52) | 29.9 (18, 52) | 31.5 (20, 54) | 32.9 (21, 55) | 34.1 (22, 56) | 35.2 (22, 56) | 36.1 (23, 55) | 36.8 (24, 56) |
|  | WHO 2013 | 29.6 (25, 48) | 36.8 (26, 53) | 38.1 (27, 56) | 39.1 (28, 57) | 40.0 (28, 59) | 40.7 (28, 59) | 41.3 (29, 61) | 41.8 (29, 61) |
|  | 90-90-90 | 42.8 (28, 64) | 42.7 (28, 63) | 42.9 (28, 63) | 43.1 (29, 64) | 43.2 (29, 64) | 43.4 (29, 65) | 43.5 (29, 65) | 43.5 (29, 65) |
|  | Current | 38.5 (10, 101) | 39.9 (11, 101) | 41.5 (12, 103) | 43.3 (14, 112) | 45.3 (15, 132) | 47.1 (15, 149) | 48.8 (18, 171) | 50.6 (18, 186) |
| **Guatemala** | WHO 2013 | 38.5 (10, 101) | 39.9 (11, 101) | 41.4 (12, 103) | 43.3 (14, 112) | 45.3 (15, 132) | 47.1 (15, 149) | 48.8 (18, 171) | 50.6 (18, 186) |
|  | 90-90-90 | 49.9 (13, 127) | 51.7 (15, 127) | 53.6 (17, 140) | 55.8 (18, 168) | 58.1 (20, 199) | 60.2 (21, 227) | 62.2 (23, 244) | 64.2 (23, 265) |
|  | Current | 4.7 (4, 5) | 5.1 (4, 6) | 5.5 (4, 7) | 5.9 (4, 8) | 6.3 (4, 9) | 6.7 (4, 10) | 7.0 (5, 11) | 7.3 (5, 11) |
| **Guyana** | WHO 2013 | 4.6 (3, 5) | 6.4 (4, 10) | 6.8 (4, 11) | 7.2 (4, 12) | 7.6 (4, 13) | 7.9 (4, 13) | 8.1 (5, 14) | 8.4 (5, 14) |
|  | 90-90-90 | 7.7 (4, 14) | 8.0 (4, 15) | 8.3 (4, 15) | 8.6 (4, 16) | 8.8 (4, 16) | 9.0 (5, 16) | 9.2 (5, 17) | 9.3 (5, 17) |
|  | Current | 74.0 (67, 137) | 103.8 (92, 157) | 111.1 (100, 166) | 117.2 (106, 174) | 122.4 (111, 180) | 126.8 (115, 185) | 130.5 (119, 189) | 133.5 (122, 194) |
| **Haiti** | WHO 2013 | 74.2 (68, 138) | 103.9 (92, 157) | 111.1 (100, 166) | 117.3 (106, 173) | 122.5 (111, 180) | 126.9 (115, 185) | 130.6 (119, 190) | 133.7 (122, 194) |
|  | 90-90-90 | 126.3 (114, 190) | 130.4 (118, 194) | 134.5 (122, 199) | 137.7 (124, 203) | 140.5 (127, 206) | 142.8 (129, 209) | 144.7 (131, 212) | 146.1 (133, 213) |
|  | Current | 18.3 (15, 22) | 18.2 (15, 22) | 18.2 (15, 22) | 18.3 (15, 22) | 18.5 (15, 22) | 18.7 (15, 23) | 19.1 (15, 23) | 19.5 (16, 24) |
| **Honduras** | WHO 2013 | 14.2 (12, 17) | 18.3 (15, 22) | 18.3 (15, 22) | 18.4 (15, 22) | 18.5 (15, 22) | 18.8 (15, 23) | 19.1 (15, 23) | 19.5 (16, 24) |
|  | 90-90-90 | 23.2 (19, 27) | 22.9 (19, 27) | 22.7 (19, 27) | 22.6 (19, 27) | 22.6 (19, 27) | 22.7 (19, 27) | 23.0 (19, 28) | 23.3 (19, 28) |
|  | Current | 16.3 (12, 35) | 17.0 (13, 35) | 18.1 (14, 36) | 18.5 (14, 35) | 18.9 (14, 35) | 19.5 (15, 35) | 20.3 (16, 35) | 21.1 (16, 36) |
| **Jamaica** | WHO 2013 | 16.3 (13, 33) | 22.8 (18, 33) | 23.3 (19, 34) | 23.6 (19, 34) | 24.1 (19, 35) | 24.7 (20, 36) | 25.3 (20, 37) | 25.9 (21, 38) |
|  | 90-90-90 | 29.6 (24, 41) | 29.6 (24, 41) | 29.3 (23, 41) | 28.8 (23, 40) | 28.4 (23, 40) | 28.2 (23, 39) | 28.4 (23, 39) | 28.6 (23, 40) |
|  | Current | 4.0 (4, 5) | 4.2 (4, 6) | 4.5 (4, 7) | 5.1 (4, 8) | 6.2 (5, 10) | 7.3 (6, 13) | 8.6 (7, 15) | 9.6 (8, 17) |
| **Nicaragua** | WHO 2013 | 4.0 (4, 5) | 5.9 (5, 11) | 6.6 (5, 12) | 7.5 (6, 14) | 8.7 (7, 16) | 9.9 (8, 18) | 11.1 (9, 20) | 12.4 (9, 22) |
|  | 90-90-90 | 7.4 (6, 14) | 7.9 (6, 16) | 8.6 (7, 17) | 9.4 (7, 18) | 10.3 (8, 20) | 11.4 (8, 22) | 12.6 (9, 23) | 13.9 (10, 26) |
|  | Current | 2.0 (2, 2) | 2.0 (2, 2) | 2.3 (2, 2) | 2.5 (2, 3) | 2.8 (3, 3) | 3.0 (3, 3) | 3.2 (3, 4) | 3.4 (3, 4) |
| **Suriname** | WHO 2013 | 2.0 (2, 2) | 2.7 (2, 3) | 2.9 (3, 3) | 3.1 (3, 4) | 3.4 (3, 4) | 3.6 (3, 4) | 3.7 (3, 5) | 3.9 (4, 5) |
|  | 90-90-90 | 3.2 (3, 4) | 3.3 (3, 4) | 3.4 (3, 4) | 3.5 (3, 4) | 3.6 (3, 5) | 3.8 (3, 5) | 3.9 (3, 5) | 4.1 (4, 5) |
| **Trinidad and Tobago** | Current | 8.1 (8, 8) | 8.5 (8, 9) | 10.7 (10, 11) | 11.8 (11, 12) | 12.6 (12, 13) | 13.2 (12, 14) | 13.8 (13, 15) | 14.2 (13, 15) |
|  | WHO 2013 | 8.1 (7, 8) | 11.1 (10, 12) | 11.7 (11, 12) | 12.2 (11, 13) | 12.8 (12, 14) | 13.2 (12, 14) | 13.7 (13, 15) | 14.1 (13, 15) |
|  | 90-90-90 | 13.7 (13, 15) | 13.9 (13, 15) | 14.2 (13, 15) | 14.6 (13, 16) | 14.9 (14, 16) | 15.2 (14, 16) | 15.5 (14, 17) | 15.8 (14, 17) |
|  | Current | 16.2 (8, 45) | 17.2 (8, 45) | 18.3 (9, 46) | 19.5 (10, 46) | 20.7 (11, 46) | 22.0 (12, 47) | 23.4 (14, 48) | 25.0 (15, 49) |
| **Algeria** | WHO 2013 | 16.2 (8, 45) | 17.3 (8, 46) | 18.4 (9, 46) | 19.6 (10, 46) | 20.8 (11, 46) | 22.1 (12, 47) | 23.6 (14, 48) | 25.1 (15, 50) |
|  | 90-90-90 | 24.2 (11, 62) | 25.4 (13, 61) | 26.5 (14, 59) | 27.6 (15, 59) | 28.7 (16, 59) | 29.9 (18, 58) | 31.1 (18, 58) | 32.3 (19, 59) |
|  | Current | 5.0 (4, 6) | 4.8 (4, 6) | 4.6 (4, 6) | 4.4 (4, 5) | 4.3 (4, 5) | 4.1 (3, 5) | 4.0 (3, 5) | 3.9 (3, 5) |
| **Djibouti** | WHO 2013 | 5.1 (4, 6) | 4.9 (4, 6) | 4.7 (4, 6) | 4.5 (4, 5) | 4.4 (4, 5) | 4.3 (4, 5) | 4.2 (3, 5) | 4.0 (3, 5) |
|  | 90-90-90 | 6.0 (5, 7) | 5.6 (5, 7) | 5.3 (4, 7) | 5.1 (4, 6) | 4.8 (4, 6) | 4.6 (4, 6) | 4.5 (4, 5) | 4.3 (4, 5) |
|  | Current | 2.9 (2, 4) | 5.6 (4, 9) | 6.4 (4, 11) | 7.2 (5, 13) | 8.2 (5, 14) | 9.3 (6, 17) | 10.7 (6, 19) | 12.2 (7, 23) |
| **Egypt** | WHO 2013 | 2.9 (3, 4) | 5.6 (4, 10) | 6.4 (4, 11) | 7.2 (5, 13) | 8.2 (5, 15) | 9.3 (6, 17) | 10.7 (6, 20) | 12.2 (7, 24) |
|  | 90-90-90 | 7.3 (5, 12) | 8.4 (5, 14) | 9.6 (6, 17) | 10.6 (7, 19) | 11.9 (7, 22) | 13.5 (8, 25) | 15.2 (9, 28) | 17.0 (9, 32) |
|  | Current | 32.0 (30, 33) | 34.0 (32, 36) | 35.9 (33, 38) | 37.8 (35, 40) | 40.0 (36, 42) | 42.5 (39, 45) | 45.5 (41, 49) | 49.4 (45, 53) |
| **Iran** | WHO 2013 | 32.0 (30, 33) | 56.5 (54, 58) | 59.2 (56, 61) | 61.9 (58, 64) | 64.8 (60, 67) | 68.1 (63, 71) | 72.0 (66, 75) | 76.7 (71, 80) |
|  | 90-90-90 | 79.3 (77, 81) | 81.9 (79, 83) | 84.4 (81, 86) | 86.7 (83, 89) | 89.2 (84, 92) | 91.8 (87, 94) | 94.8 (89, 98) | 98.3 (92, 102) |
|  | Current | 12.5 (11, 14) | 22.2 (16, 30) | 24.2 (18, 33) | 26.4 (20, 36) | 28.7 (21, 39) | 31.0 (23, 42) | 33.1 (24, 45) | 35.2 (26, 47) |
| **Morocco** | WHO 2013 | 12.5 (12, 14) | 22.2 (17, 30) | 24.2 (18, 33) | 26.4 (20, 36) | 28.7 (22, 39) | 31.0 (23, 42) | 33.1 (25, 45) | 35.2 (26, 47) |
|  | 90-90-90 | 30.0 (21, 41) | 32.2 (23, 45) | 34.5 (25, 48) | 36.5 (26, 51) | 38.5 (28, 53) | 40.5 (29, 56) | 42.3 (30, 58) | 44.0 (31, 60) |
|  | Current | 9.7 (6, 14) | 9.9 (6, 15) | 10.3 (7, 15) | 10.7 (7, 16) | 11.2 (7, 17) | 11.9 (8, 18) | 12.6 (8, 19) | 13.6 (9, 20) |
| **Somalia** | WHO 2013 | 12.5 (8, 18) | 19.5 (13, 28) | 20.3 (13, 30) | 21.2 (14, 31) | 22.1 (14, 32) | 23.2 (15, 35) | 24.6 (15, 37) | 26.1 (16, 39) |
|  | 90-90-90 | 26.8 (18, 39) | 27.3 (18, 40) | 27.8 (18, 40) | 28.4 (18, 41) | 29.1 (18, 42) | 30.0 (19, 44) | 30.9 (19, 46) | 32.0 (19, 50) |
|  | Current | 17.7 (13, 29) | 31.4 (22, 52) | 33.2 (21, 55) | 35.4 (21, 58) | 37.9 (21, 63) | 40.2 (21, 70) | 42.4 (21, 77) | 44.4 (21, 85) |
| **Sudan** | WHO 2013 | 18.4 (14, 29) | 31.4 (22, 52) | 33.2 (21, 55) | 35.4 (21, 58) | 37.9 (21, 63) | 40.2 (21, 70) | 42.4 (21, 77) | 44.4 (21, 85) |
|  | 90-90-90 | 44.7 (32, 76) | 46.7 (31, 80) | 48.7 (30, 82) | 51.2 (29, 87) | 53.8 (29, 93) | 56.3 (28, 102) | 58.6 (27, 110) | 60.8 (26, 120) |
|  | Current | 2.3 (1, 4) | 2.6 (1, 4) | 2.9 (2, 5) | 3.1 (2, 5) | 3.5 (2, 5) | 3.8 (2, 6) | 4.1 (2, 6) | 4.5 (3, 7) |
| **Tunisia** | WHO 2013 | 1.2 (1, 2) | 2.4 (2, 4) | 2.7 (2, 4) | 3.0 (2, 5) | 3.3 (2, 5) | 3.6 (2, 5) | 3.9 (2, 6) | 4.3 (2, 6) |
|  | 90-90-90 | 3.4 (2, 6) | 3.8 (2, 7) | 4.2 (2, 7) | 4.5 (3, 7) | 4.9 (3, 7) | 5.2 (3, 8) | 5.5 (3, 8) | 5.9 (3, 9) |
|  | Current | 2.8 (1, 7) | 4.6 (2, 12) | 4.9 (2, 12) | 5.2 (2, 13) | 5.6 (3, 14) | 6.0 (3, 15) | 6.5 (3, 16) | 7.2 (4, 18) |
| **Yemen** | WHO 2013 | 3.0 (1, 122) | 4.7 (2, 117) | 5.0 (2, 114) | 5.4 (2, 113) | 5.8 (3, 114) | 6.2 (3, 112) | 6.8 (3, 110) | 7.4 (4, 113) |
|  | 90-90-90 | 6.3 (3, 139) | 6.6 (3, 134) | 6.9 (3, 131) | 7.2 (3, 127) | 7.6 (3, 122) | 8.1 (4, 119) | 8.6 (4, 117) | 9.1 (4, 114) |

**Table D. Need for pediatric ART, based on results from Spectrum uncertainty analysis (in thousands)***“90-90-90” scenario results below reflect adult PLHIV*

| **Country** | **Scenario** | **2013** | **2014** | **2015** | **2016** | **2017** | **2018** | **2019** | **2020** |
| --- | --- | --- | --- | --- | --- | --- | --- | --- | --- |
|  | Current | 15.2 (10.7, 20.7) | 14.1 (10.5, 18.6) | 15.0 (11.2, 20.0) | 17.4 (12.9, 23.5) | 20.6 (14.8, 27.8) | 24.2 (17.1, 33.2) | 28.1 (19.4, 38.9) | 30.0 (20.3, 41.3) |
| **Angola** | WHO 2013 | 16.8 (12.0, 23.4) | 17.3 (12.5, 23.5) | 18.2 (13.2, 24.4) | 20.6 (14.9, 28.0) | 23.7 (17.0, 32.2) | 27.4 (19.2, 37.5) | 31.3 (21.6, 43.6) | 33.3 (22.7, 46.2) |
|  | 90-90-90 | 26.4 (18.1, 38.6) | 24.8 (17.4, 35.5) | 26.2 (18.2, 37.4) | 27.5 (19.1, 38.8) | 29.6 (20.5, 41.7) | 32.1 (22.1, 44.7) | 34.8 (23.9, 48.2) | 37.6 (25.4, 53.0) |
|  | Current | 10.0 (9.9, 10.1) | 10.5 (10.3, 10.6) | 10.5 (10.4, 10.7) | 10.4 (10.2, 10.6) | 10.0 (9.7, 10.2) | 9.4 (9.2, 9.7) | 9.0 (8.7, 9.4) | 8.8 (8.5, 9.2) |
| **Botswana** | WHO 2013 | 10.1 (10.0, 10.2) | 10.7 (10.5, 10.8) | 10.7 (10.5, 10.8) | 10.4 (10.2, 10.6) | 10.0 (9.7, 10.2) | 9.5 (9.2, 9.8) | 9.0 (8.7, 9.4) | 8.8 (8.5, 9.2) |
|  | 90-90-90 | 16.5 (15.5, 17.4) | 15.3 (14.5, 16.1) | 14.1 (13.4, 14.7) | 12.8 (12.3, 13.4) | 11.6 (11.1, 12.1) | 10.4 (10.0, 10.8) | 9.6 (9.2, 10.0) | 9.2 (8.7, 9.6) |
|  | Current | 7.5 (6.6, 13.7) | 6.9 (6.1, 13.4) | 6.4 (5.4, 12.9) | 5.9 (5.0, 13.2) | 5.9 (4.8, 13.0) | 6.0 (4.8, 13.1) | 6.6 (5.2, 14.2) | 7.0 (5.5, 15.2) |
| **Burundi** | WHO 2013 | 8.4 (7.5, 9.3) | 8.6 (7.7, 9.6) | 8.0 (7.1, 9.0) | 7.5 (6.6, 8.6) | 7.4 (6.4, 8.5) | 7.3 (6.3, 8.6) | 7.5 (6.4, 8.8) | 7.6 (6.4, 9.1) |
|  | 90-90-90 | 16.4 (14.4, 21.8) | 15.9 (14.0, 22.2) | 15.3 (13.5, 22.1) | 14.6 (12.7, 22.1) | 14.1 (12.1, 22.1) | 13.4 (11.4, 21.6) | 12.9 (10.7, 21.6) | 13.0 (10.7, 22.7) |
|  | Current | 0.5 (0.5, 0.6) | 1.0 (0.5, 1.1) | 1.6 (1.5, 1.8) | 2.3 (2.1, 2.5) | 3.0 (2.7, 3.3) | 3.7 (3.3, 4.0) | 4.3 (3.9, 4.8) | 4.7 (4.2, 5.1) |
| **Comoros** | WHO 2013 | 0.6 (0.5, 0.6) | 1.0 (0.5, 1.1) | 1.7 (1.5, 1.8) | 2.4 (2.1, 2.6) | 3.1 (2.8, 3.4) | 3.9 (3.5, 4.3) | 4.7 (4.2, 5.2) | 5.1 (4.6, 5.6) |
|  | 90-90-90 | 0.3 (0.3, 0.3) | 0.6 (0.3, 0.7) | 1.1 (1.0, 1.2) | 1.7 (1.5, 1.9) | 2.4 (2.2, 2.6) | 3.2 (2.9, 3.5) | 4.0 (3.6, 4.4) | 4.9 (4.4, 5.4) |
|  | Current | 1.5 (1.3, 1.8) | 1.3 (1.2, 1.6) | 1.2 (1.1, 1.4) | 1.1 (1.0, 1.2) | 1.0 (0.9, 1.1) | 0.9 (0.9, 1.0) | 0.8 (0.8, 0.9) | 0.8 (0.7, 0.9) |
| **Eritrea** | WHO 2013 | 1.6 (1.4, 2.0) | 1.6 (1.3, 1.9) | 1.4 (1.2, 1.7) | 1.3 (1.1, 1.5) | 1.1 (1.0, 1.3) | 1.0 (0.9, 1.1) | 0.9 (0.8, 1.0) | 0.8 (0.7, 0.9) |
|  | 90-90-90 | 2.9 (2.2, 3.9) | 2.7 (2.0, 3.5) | 2.4 (1.9, 3.2) | 2.2 (1.7, 2.9) | 2.0 (1.6, 2.6) | 1.8 (1.4, 2.3) | 1.6 (1.3, 2.1) | 1.4 (1.1, 1.8) |
|  | Current | 104.5  (91.8, 120.1) | 149.7  (131.4, 174.0) | 131.2  (115.0, 152.4) | 114.9  (100.8, 132.0) | 101.9  (89.5, 117.8) | 91.4  (80.1, 106.8) | 82.0 (71.5, 96.5) | 73.2 (63.6, 86.7) |
| **Ethiopia** | WHO 2013 | 59.2 (52.3, 68.2) | 64.2 (56.9, 73.3) | 56.0 (50.0, 63.9) | 50.7 (45.0, 57.8) | 48.4 (42.8, 55.5) | 47.7 (41.9, 55.2) | 46.7 (40.9, 55.2) | 45.4 (39.4, 54.2) |
|  | 90-90-90 | 171.5  (150.5, 196.2) | 152.3  (134.2, 176.1) | 133.8  (117.7, 155.1) | 117.8  (103.5, 135.1) | 105.7  (92.6, 121.2) | 95.5  (83.6, 111.3) | 85.7  (75.0, 100.6) | 76.6 (66.6, 90.3) |
|  | Current | 107.5  (99.2, 116.7) | 124.3  (113.8, 136.3) | 139.5  (126.6, 154.2) | 138.4  (124.5, 155.7) | 138.0  (122.8, 157.1) | 138.0  (121.8, 158.0) | 138.4  (121.5, 159.0) | 138.6  (120.8, 159.8) |
| **Kenya** | WHO 2013 | 108.9  (100.3, 118.3) | 112.0  (103.1, 121.6) | 114.3  (104.7, 124.9) | 117.0  (106.6, 128.5) | 119.2  (108.4, 132.2) | 120.8  (108.8, 134.0) | 122.0  (108.9, 135.6) | 122.6  (108.4, 136.2) |
|  | 90-90-90 | 196.1  (182.4, 212.8) | 189.3  (176.3, 206.4) | 182.6  (169.5, 200.0) | 175.7  (161.1, 193.1) | 169.7 (154.2, 188.2) | 164.2  (147.0, 184.7) | 159.4  (142.0, 181.2) | 154.9  (136.3, 177.5) |
|  | Current | 16.5 (15.5, 17.7) | 14.3 (13.5, 15.1) | 12.7 (12.1, 13.5) | 11.5 (11.0, 12.1) | 10.2 (9.8, 10.7) | 9.3 (9.0, 9.9) | 8.8 (8.4, 9.4) | 8.5 (8.1, 9.1) |
| **Lesotho** | WHO 2013 | 18.1 (17.0, 19.4) | 17.4 (16.5, 18.4) | 15.8 (15.0, 16.7) | 14.4 (13.8, 15.2) | 13.0 (12.5, 13.7) | 12.0 (11.5, 12.6) | 11.3 (10.7, 11.9) | 10.8 (10.3, 11.5) |
|  | 90-90-90 | 33.5 (31.5, 35.7) | 30.8 (29.0, 32.8) | 29.0 (27.3, 30.7) | 27.2 (25.7, 28.9) | 25.5 (24.0, 27.3) | 23.8 (22.5, 25.5) | 22.1 (20.8, 23.8) | 20.3 (19.1, 21.9) |
|  | Current | 3.0 (2.2, 4.8) | 3.2 (2.4, 5.1) | 2.9 (2.2, 4.8) | 2.7 (2.0, 4.5) | 2.4 (1.7, 4.2) | 2.1 (1.4, 3.8) | 1.9 (1.3, 3.5) | 1.8 (1.3, 3.4) |
| **Madagascar** | WHO 2013 | 3.0 (2.3, 4.9) | 3.3 (2.5, 5.4) | 3.1 (2.3, 5.0) | 2.8 (2.1, 4.7) | 2.5 (1.8, 4.4) | 2.2 (1.5, 4.0) | 2.0 (1.3, 3.7) | 1.9 (1.3, 3.6) |
|  | 90-90-90 | 6.4 (5.2, 9.6) | 6.2 (5.0, 9.3) | 6.0 (4.8, 9.1) | 5.7 (4.5, 8.8) | 5.3 (4.2, 8.5) | 4.9 (3.8, 8.1) | 4.5 (3.4, 7.7) | 4.2 (3.1, 7.3) |
|  | Current | 77.9 (72.9, 84.0) | 87.1 (81.8, 94.2) | 86.5 (80.8, 93.9) | 86.2 (79.7, 94.6) | 86.5 (79.1, 95.9) | 86.5 (78.3, 96.9) | 85.9 (76.8, 97.1) | 84.1 (74.0, 96.3) |
| **Malawi** | WHO 2013 | 78.0 (73.2, 85.0) | 87.3 (82.1, 94.8) | 86.8 (81.1, 94.5) | 86.6 (80.2, 95.3) | 87.2 (79.8, 96.9) | 87.5 (79.3, 98.8) | 87.2  (78.0, 100.0) | 85.5 (75.3, 99.7) |
|  | 90-90-90 | 155.5  (145.8, 166.4) | 151.1  (142.0, 161.2) | 145.5  (136.1, 155.7) | 139.8  (129.6, 151.2) | 134.3  (124.0, 146.4) | 128.8  (118.0, 141.6) | 123.3  (112.1, 137.1) | 117.5  (105.9, 132.1) |
|  | Current | 0.0 (0.0, 0.0) | 0.0 (0.0, 0.0) | 0.0 (0.0, 0.0) | 0.0 (0.0, 0.1) | 0.1 (0.1, 0.1) | 0.1 (0.1, 0.1) | 0.1 (0.1, 0.1) | 0.1 (0.1, 0.1) |
| **Mauritius** | WHO 2013 | 0.0 (0.0, 0.0) | 0.0 (0.0, 0.0) | 0.0 (0.0, 0.0) | 0.1 (0.0, 0.1) | 0.1 (0.1, 0.1) | 0.1 (0.1, 0.1) | 0.1 (0.1, 0.1) | 0.1 (0.1, 0.1) |
|  | 90-90-90 | 0.1 (0.1, 0.1) | 0.1 (0.1, 0.1) | 0.1 (0.1, 0.1) | 0.1 (0.1, 0.1) | 0.1 (0.1, 0.1) | 0.1 (0.1, 0.1) | 0.1 (0.1, 0.1) | 0.1 (0.1, 0.2) |
|  | Current | 80.8 (72.1, 93.0) | 84.0 (75.4, 95.0) | 92.5  (83.0, 103.3) | 105.9  (94.8, 117.8) | 118.9  (106.3, 132.4) | 130.1  (115.9, 145.6) | 138.2  (122.7, 156.3) | 141.6  (125.3, 161.0) |
| **Mozambique** | WHO 2013 | 89.0  (78.9, 102.3) | 99.1  (89.0, 111.6) | 104.4  (94.8, 115.1) | 115.0  (104.5, 125.9) | 125.9  (114.1, 138.3) | 135.5  (122.5, 150.3) | 143.5  (128.9, 161.1) | 146.9  (131.3, 165.9) |
|  | 90-90-90 | 167.4  (146.8, 190.9) | 167.2  (147.9, 189.4) | 175.2  (155.1, 197.4) | 181.4  (160.0, 203.2) | 186.1  (164.2, 208.4) | 188.0  (166.3, 211.6) | 187.6  (165.3, 213.1) | 185.6  (162.2, 212.8) |
|  | Current | 16.7 (15.3, 19.1) | 19.5 (17.5, 22.7) | 19.4 (17.4, 22.8) | 19.4 (17.3, 23.1) | 18.9 (16.7, 22.7) | 17.8 (15.7, 21.5) | 16.3 (14.3, 20.0) | 15.5 (13.6, 19.3) |
| **Namibia** | WHO 2013 | 14.7 (13.9, 16.3) | 15.5 (14.6, 17.1) | 15.6 (14.8, 17.2) | 15.7 (14.8, 17.8) | 15.3 (14.3, 17.9) | 14.4 (13.3, 17.2) | 13.2 (12.0, 16.1) | 12.5 (11.3, 15.6) |
|  | 90-90-90 | 23.1 (20.3, 27.3) | 22.5 (19.9, 26.7) | 22.2 (19.6, 26.4) | 22.1 (19.4, 26.7) | 21.4 (18.6, 26.1) | 20.2 (17.6, 24.6) | 18.4 (16.0, 22.7) | 16.4 (14.3, 20.6) |
|  | Current | 10.4 (9.8, 11.1) | 10.4 (10.0, 11.0) | 10.6 (10.1, 11.0) | 10.6 (10.3, 11.0) | 10.5 (10.2, 10.9) | 10.1 (9.8, 10.5) | 9.4 (9.1, 9.8) | 9.0 (8.7, 9.4) |
| **Rwanda** | WHO 2013 | 11.3 (10.6, 12.2) | 12.0 (11.3, 12.9) | 11.7 (11.0, 12.4) | 11.4 (10.8, 12.0) | 11.0 (10.4, 11.6) | 10.4 (9.9, 10.9) | 9.6 (9.1, 10.1) | 9.2 (8.8, 9.7) |
|  | 90-90-90 | 21.8 (19.7, 24.1) | 20.4 (18.5, 22.6) | 19.0 (17.1, 21.0) | 17.6 (15.8, 19.4) | 16.3 (14.6, 18.1) | 15.1 (13.5, 16.6) | 13.8 (12.5, 15.2) | 12.6 (11.4, 13.9) |
|  | Current | 149.2  (136.3, 157.4) | 167.0  (153.0, 177.5) | 176.0  (163.8, 186.6) | 178.2  (168.0, 188.3) | 181.9  (171.5, 193.2) | 187.0  (176.0, 199.0) | 189.7  (177.3, 202.2) | 190.6  (177.7, 203.8) |
| **South Africa** | WHO 2013 | 163.7  (150.7, 172.7) | 193.5  (177.8, 204.9) | 198.6  (183.0, 210.6) | 199.3  (184.3, 212.3) | 201.2  (185.5, 215.5) | 203.4 (187.7, 217.8) | 203.6 (187.4, 218.5) | 203.2  (187.0, 218.6) |
|  | 90-90-90 | 367.4  (341.4, 388.5) | 363.1  (335.2, 386.0) | 350.9  (325.4, 372.9) | 331.3  (305.6, 353.2) | 315.3  (289.8, 336.8) | 296.0  (270.8, 318.1) | 275.4  (252.2, 296.7) | 253.4  (231.6, 274.0) |
|  | Current | 9.6 (3.7, 27.0) | 11.1 (4.3, 30.2) | 11.0 (4.3, 29.9) | 11.0 (4.5, 29.6) | 11.1 (4.9, 29.9) | 11.1 (5.5, 29.2) | 11.2 (5.9, 29.5) | 11.3 (6.1, 29.8) |
| **South Sudan** | WHO 2013 | 9.2 (3.5, 26.0) | 10.2 (3.9, 28.4) | 10.2 (3.9, 28.0) | 10.3 (4.2, 28.0) | 10.5 (4.7, 27.9) | 10.6 (5.3, 27.8) | 10.7 (5.8, 28.2) | 10.7 (6.0, 28.6) |
|  | 90-90-90 | 15.8 (6.8, 39.2) | 16.3 (6.9, 40.1) | 16.8 (6.9, 41.2) | 17.3 (7.2, 42.7) | 17.7 (7.7, 44.9) | 17.8 (8.2, 45.6) | 17.9 (8.6, 46.5) | 18.0 (9.0, 46.6) |
|  | Current | 10.9 (10.6, 11.2) | 12.1 (11.7, 12.4) | 12.4 (12.0, 12.8) | 12.5 (12.1, 13.1) | 12.7 (12.2, 13.3) | 12.7 (12.2, 13.3) | 12.7 (12.1, 13.3) | 12.6 (12.0, 13.2) |
| **Swaziland** | WHO 2013 | 11.0 (10.7, 11.3) | 12.2 (11.8, 12.5) | 12.5 (12.0, 12.9) | 12.6 (12.2, 13.2) | 12.8 (12.3, 13.4) | 12.8 (12.3, 13.4) | 12.7 (12.2, 13.3) | 12.7 (12.1, 13.3) |
|  | 90-90-90 | 17.6 (16.9, 18.3) | 17.8 (17.1, 18.4) | 17.6 (16.9, 18.3) | 17.3 (16.6, 18.1) | 17.0 (16.3, 17.8) | 16.6 (15.9, 17.4) | 16.1 (15.3, 17.0) | 15.6 (14.7, 16.5) |
|  | Current | 68.9 (61.7, 76.6) | 119.6  (109.0, 131.5) | 165.5  (150.5, 181.9) | 155.9  (140.9, 171.9) | 146.8  (131.9, 162.1) | 135.5  (121.5, 149.9) | 123.2  (109.9, 136.9) | 116.4  (103.5, 129.4) |
| **Tanzania** | WHO 2013 | 87.2 (77.1, 96.6) | 93.7  (84.5, 101.8) | 87.3 (78.9, 94.4) | 82.0 (74.5, 89.3) | 77.9 (70.8, 84.9) | 74.8 (67.5, 81.8) | 72.3 (65.0, 79.7) | 71.2 (63.9, 78.8) |
|  | 90-90-90 | 204.1  (184.8, 226.4) | 199.5  (181.3, 219.9) | 185.4  (168.0, 205.1) | 171.0  (154.4, 190.3) | 158.2  (141.2, 176.0) | 145.7  (129.6, 162.4) | 133.4  (118.5, 148.7) | 120.7  (106.2, 135.1) |
|  | Current | 181.8  (166.4, 198.0) | 172.0  (158.0, 186.5) | 166.7  (152.8, 180.3) | 164.2  (149.8, 178.0) | 162.5  (147.6, 175.9) | 161.2  (146.1, 174.1) | 160.2  (144.1, 174.0) | 159.7  (142.9, 173.7) |
| **Uganda** | WHO 2013 | 96.3 (87.8, 105.9) | 99.6  (92.6, 108.1) | 92.5 (86.4, 99.8) | 86.5 (80.9, 93.0) | 83.9 (77.8, 90.3) | 84.1 (77.2, 91.0) | 86.1 (77.6, 93.6) | 87.1 (78.4, 95.1) |
|  | 90-90-90 | 179.7  (164.7, 195.9) | 169.4  (156.0, 184.1) | 163.9  (150.4, 177.5) | 160.8  (146.6, 174.5) | 159.5  (145.2, 173.0) | 158.8  (143.9, 171.9) | 158.6  (143.2, 171.5) | 158.8  (142.3, 172.8) |
|  | Current | 63.6 (58.7, 68.3) | 64.8 (59.5, 69.6) | 67.8 (62.5, 73.1) | 70.6 (65.4, 76.2) | 72.1 (67.2, 78.4) | 73.6 (68.7, 80.2) | 75.0 (69.6, 82.1) | 75.6 (69.5, 82.1) |
| **Zambia** | WHO 2013 | 70.7 (65.0, 75.9) | 77.7 (71.2, 83.8) | 79.2 (72.1, 85.6) | 80.4 (73.1, 86.9) | 79.7 (72.2, 87.0) | 78.9 (71.1, 86.7) | 77.8 (70.4, 86.7) | 77.3 (69.7, 86.4) |
|  | 90-90-90 | 128.2  (118.5, 137.5) | 127.0  (117.1, 136.6) | 125.0  (114.8, 135.2) | 122.0  (111.5, 132.0) | 118.3  (107.5, 129.1) | 114.8  (104.3, 126.0) | 111.4  (100.9, 122.4) | 107.2  (94.6, 117.9) |
|  | Current | 81.3 (76.7, 86.2) | 84.3 (79.9, 88.7) | 80.2 (75.7, 84.4) | 78.8 (73.8, 83.6) | 78.7 (73.7, 84.3) | 78.4 (73.3, 84.4) | 76.6 (71.0, 82.6) | 75.1 (69.6, 81.1) |
| **Zimbabwe** | WHO 2013 | 81.3 (76.7, 86.2) | 84.3 (79.9, 88.7) | 80.1 (75.6, 84.3) | 78.6 (73.7, 83.4) | 78.5 (73.6, 84.0) | 78.2 (73.1, 84.1) | 76.3 (70.6, 82.2) | 74.8 (69.2, 80.7) |
|  | 90-90-90 | 169.2  (158.4, 179.7) | 155.0  (145.6, 164.5) | 145.0  (135.8, 154.5) | 136.3  (127.0, 145.5) | 129.3  (120.4, 138.6) | 122.2  (113.3, 131.3) | 113.7  (105.4, 122.5) | 105.1  (97.0, 113.8) |
|  | Current | 0.2 (0.2, 0.3) | 0.3 (0.3, 0.3) | 0.3 (0.3, 0.3) | 0.3 (0.3, 0.3) | 0.4 (0.4, 0.4) | 0.4 (0.4, 0.4) | 0.5 (0.5, 0.5) | 0.5 (0.5, 0.6) |
| **Bangladesh** | WHO 2013 | 0.3 (0.3, 0.3) | 0.3 (0.3, 0.3) | 0.3 (0.3, 0.3) | 0.4 (0.3, 0.4) | 0.4 (0.4, 0.4) | 0.5 (0.5, 0.5) | 0.5 (0.5, 0.6) | 0.6 (0.6, 0.6) |
|  | 90-90-90 | 0.3 (0.3, 0.4) | 0.4 (0.3, 0.4) | 0.4 (0.4, 0.4) | 0.4 (0.4, 0.4) | 0.4 (0.4, 0.5) | 0.5 (0.5, 0.5) | 0.6 (0.6, 0.6) | 0.7 (0.6, 0.7) |
|  | Current | 0.0 (0.0, 0.1) | 0.0 (0.0, 0.1) | 0.0 (0.0, 0.2) | 0.0 (0.0, 0.2) | 0.0 (0.0, 0.2) | 0.0 (0.0, 0.3) | 0.0 (0.0, 0.3) | 0.0 (0.0, 0.4) |
| **Bhutan** | WHO 2013 | 0.0 (0.0, 0.1) | 0.0 (0.0, 0.2) | 0.1 (0.0, 0.2) | 0.1 (0.0, 0.3) | 0.1 (0.0, 0.3) | 0.1 (0.0, 0.4) | 0.1 (0.0, 0.5) | 0.1 (0.0, 0.5) |
|  | 90-90-90 | 0.1 (0.0, 0.1) | 0.1 (0.0, 0.2) | 0.1 (0.0, 0.2) | 0.1 (0.0, 0.3) | 0.1 (0.0, 0.3) | 0.1 (0.0, 0.4) | 0.1 (0.0, 0.4) | 0.1 (0.0, 0.5) |
|  | Current | 5.4 (2.0, 9.6) | 5.2 (1.9, 9.4) | 5.1 (1.9, 9.3) | 5.0 (1.8, 9.2) | 4.8 (1.8, 9.1) | 4.5 (1.7, 8.4) | 4.1 (1.6, 7.1) | 3.6 (1.4, 6.0) |
| **Cambodia** | WHO 2013 | 5.5 (2.0, 9.9) | 5.4 (2.0, 10.0) | 5.3 (2.0, 9.9) | 5.2 (2.0, 9.9) | 5.0 (2.0, 9.5) | 4.7 (1.9, 8.4) | 4.2 (1.7, 6.8) | 3.7 (1.6, 5.9) |
|  | 90-90-90 | 6.8 (2.4, 12.2) | 6.4 (2.3, 11.6) | 6.1 (2.2, 11.0) | 5.9 (2.2, 10.9) | 5.6 (2.1, 10.4) | 5.3 (2.0, 10.2) | 4.8 (1.9, 8.8) | 4.3 (1.7, 7.5) |
|  | Current | 8.9 (8.6, 9.2) | 9.6 (9.3, 9.9) | 10.3 (10.0, 10.6) | 10.9 (10.6, 11.3) | 11.8 (11.5, 12.2) | 13.0 (12.7, 13.4) | 14.2 (13.8, 14.5) | 14.7 (14.4, 15.1) |
| **China** | WHO 2013 | 10.1 (9.8, 10.4) | 12.2 (11.9, 12.5) | 13.3 (13.0, 13.8) | 14.4 (14.0, 14.9) | 15.8 (15.3, 16.4) | 17.7 (17.2, 18.4) | 19.6 (19.0, 20.5) | 20.6 (19.9, 21.5) |
|  | 90-90-90 | 14.8 (14.5, 15.2) | 15.9 (15.5, 16.2) | 16.8 (16.4, 17.2) | 16.5 (16.1, 17.0) | 17.9 (17.4, 18.5) | 19.7 (19.1, 20.4) | 21.5 (20.8, 22.4) | 23.3 (22.4, 24.3) |
|  | Current | 58.8 (46.3, 76.0) | 61.8 (48.7, 79.9) | 63.8 (50.2, 82.4) | 65.0 (51.2, 84.0) | 66.1 (52.1, 85.5) | 67.0 (52.7, 86.5) | 67.7 (53.3, 87.4) | 68.1 (53.6, 88.0) |
| **India** | WHO 2013 | 66.9 (52.7, 86.5) | 77.6  (61.1, 100.3) | 79.0  (62.2, 102.1) | 80.4  (63.3, 104.0) | 84.1  (66.2, 108.7) | 89.2  (70.2, 115.3) | 94.1  (74.1, 121.6) | 96.5  (76.0, 124.7) |
|  | 90-90-90 | 120.6  (101.1, 140.4) | 118.2  (99.1, 137.6) | 113.9  (95.5, 132.6) | 110.1  (92.3, 128.2) | 107.1  (89.8, 124.7) | 104.7  (87.8, 121.9) | 104.1  (87.3, 121.3) | 103.6  (86.9, 120.6) |
|  | Current | 17.7 (12.0, 27.0) | 18.9 (12.7, 29.5) | 20.0 (13.5, 31.4) | 20.9 (14.3, 32.6) | 21.8 (15.0, 34.1) | 22.7 (15.7, 36.0) | 23.4 (16.4, 37.0) | 23.8 (16.9, 37.8) |
| **Indonesia** | WHO 2013 | 19.3 (13.1, 28.9) | 22.2 (15.0, 33.8) | 23.7 (15.9, 36.5) | 25.0 (16.8, 38.6) | 26.2 (17.7, 40.7) | 27.3 (18.5, 42.8) | 28.4 (19.2, 44.9) | 29.0 (19.7, 46.1) |
|  | 90-90-90 | 23.2 (16.0, 33.4) | 25.7 (17.3, 37.0) | 28.1 (18.7, 40.9) | 30.4 (20.2, 45.2) | 32.7 (21.5, 49.8) | 34.9 (22.7, 54.0) | 37.0 (23.9, 58.7) | 39.0 (25.3, 63.7) |
|  | Current | 0.3 (0.3, 0.6) | 0.4 (0.3, 0.6) | 0.4 (0.3, 0.6) | 0.4 (0.3, 0.7) | 0.4 (0.3, 0.7) | 0.4 (0.3, 0.8) | 0.4 (0.3, 0.8) | 0.4 (0.3, 0.9) |
| **Laos** | WHO 2013 | 0.4 (0.3, 0.7) | 0.5 (0.4, 0.8) | 0.5 (0.4, 0.9) | 0.6 (0.4, 1.1) | 0.6 (0.4, 1.3) | 0.6 (0.4, 1.4) | 0.7 (0.4, 1.6) | 0.7 (0.4, 1.7) |
|  | 90-90-90 | 0.6 (0.4, 1.1) | 0.6 (0.5, 1.2) | 0.6 (0.5, 1.2) | 0.6 (0.5, 1.2) | 0.6 (0.5, 1.2) | 0.6 (0.4, 1.2) | 0.7 (0.4, 1.3) | 0.7 (0.4, 1.4) |
|  | Current | 0.6 (0.4, 0.7) | 0.6 (0.5, 0.7) | 0.6 (0.5, 0.8) | 0.7 (0.5, 0.8) | 0.7 (0.5, 0.8) | 0.7 (0.5, 0.9) | 0.7 (0.6, 1.0) | 0.7 (0.6, 1.0) |
| **Malaysia** | WHO 2013 | 0.7 (0.5, 1.0) | 0.7 (0.5, 1.1) | 0.8 (0.6, 1.2) | 0.8 (0.6, 1.3) | 0.9 (0.7, 1.5) | 1.0 (0.7, 1.7) | 1.0 (0.8, 1.9) | 1.0 (0.8, 2.1) |
|  | 90-90-90 | 0.9 (0.6, 1.2) | 0.9 (0.6, 1.2) | 0.9 (0.7, 1.2) | 0.9 (0.7, 1.2) | 0.9 (0.7, 1.2) | 0.9 (0.7, 1.3) | 0.9 (0.7, 1.4) | 1.0 (0.7, 1.4) |
|  | Current | 0.0 (0.0, 0.0) | 0.0 (0.0, 0.0) | 0.0 (0.0, 0.0) | 0.0 (0.0, 0.0) | 0.0 (0.0, 0.0) | 0.0 (0.0, 0.0) | 0.0 (0.0, 0.0) | 0.0 (0.0, 0.0) |
| **Mongolia** | WHO 2013 | 0.0 (0.0, 0.0) | 0.0 (0.0, 0.0) | 0.0 (0.0, 0.0) | 0.0 (0.0, 0.0) | 0.0 (0.0, 0.0) | 0.0 (0.0, 0.0) | 0.0 (0.0, 0.0) | 0.0 (0.0, 0.0) |
|  | 90-90-90 | 0.0 (0.0, 0.0) | 0.0 (0.0, 0.0) | 0.0 (0.0, 0.0) | 0.0 (0.0, 0.0) | 0.0 (0.0, 0.0) | 0.0 (0.0, 0.0) | 0.0 (0.0, 0.0) | 0.0 (0.0, 0.0) |
|  | Current | 5.2 (5.1, 5.3) | 5.6 (5.5, 5.7) | 6.2 (6.1, 6.3) | 6.9 (6.8, 7.1) | 7.9 (7.8, 8.1) | 9.1 (8.8, 9.3) | 10.2 (9.9, 10.4) | 10.7 (10.4, 11.0) |
| **Myanmar** | WHO 2013 | 5.9 (5.7, 6.0) | 6.6 (6.5, 6.7) | 6.7 (6.6, 6.8) | 7.5 (7.3, 7.6) | 9.1 (8.8, 9.3) | 10.8 (10.5, 11.1) | 12.5 (12.2, 12.9) | 13.4 (13.0, 13.8) |
|  | 90-90-90 | 9.7 (9.5, 9.8) | 9.1 (9.0, 9.3) | 8.8 (8.7, 9.0) | 9.2 (9.0, 9.4) | 10.2 (9.9, 10.4) | 11.6 (11.3, 11.9) | 13.2 (12.9, 13.6) | 15.0 (14.5, 15.5) |
|  | Current | 0.8 (0.6, 0.9) | 0.8 (0.7, 0.9) | 0.9 (0.7, 1.0) | 1.0 (0.8, 1.2) | 1.2 (0.9, 1.3) | 1.3 (1.0, 1.4) | 1.4 (1.1, 1.5) | 1.4 (1.1, 1.5) |
| **Nepal** | WHO 2013 | 0.9 (0.8, 1.1) | 1.0 (0.9, 1.2) | 1.0 (0.9, 1.1) | 1.0 (0.9, 1.1) | 1.0 (0.9, 1.1) | 1.1 (1.0, 1.1) | 1.1 (1.0, 1.2) | 1.1 (1.0, 1.2) |
|  | 90-90-90 | 1.7 (1.3, 2.0) | 1.7 (1.3, 1.9) | 1.6 (1.3, 1.9) | 1.7 (1.4, 1.9) | 1.7 (1.4, 2.0) | 1.8 (1.3, 2.1) | 1.8 (1.4, 2.1) | 1.8 (1.4, 2.1) |
|  | Current | 1.5 (0.9, 2.9) | 1.9 (1.1, 4.0) | 2.3 (1.2, 5.4) | 2.8 (1.4, 7.1) | 3.5 (1.7, 9.3) | 4.3 (2.0, 12.4) | 5.4 (2.3, 15.2) | 6.0 (2.4, 16.9) |
| **Pakistan** | WHO 2013 | 1.6 (1.0, 3.1) | 2.1 (1.2, 4.3) | 2.6 (1.4, 5.8) | 3.1 (1.6, 7.7) | 3.9 (1.9, 10.2) | 4.8 (2.2, 13.5) | 6.0 (2.6, 16.6) | 6.6 (2.7, 18.4) |
|  | 90-90-90 | 1.6 (1.0, 2.8) | 2.0 (1.2, 3.6) | 2.4 (1.4, 4.9) | 3.0 (1.7, 6.7) | 3.7 (2.0, 8.6) | 4.5 (2.3, 11.6) | 5.6 (2.7, 15.2) | 7.0 (3.1, 18.6) |
| **Papua New Guinea** | Current | 1.9 (1.6, 2.6) | 2.1 (1.8, 2.8) | 2.3 (1.9, 3.0) | 2.5 (2.1, 3.4) | 2.7 (2.1, 3.9) | 2.9 (2.2, 4.8) | 2.9 (2.1, 5.1) | 2.7 (1.9, 4.8) |
|  | WHO 2013 | 2.3 (2.0, 2.6) | 2.9 (2.5, 3.3) | 3.1 (2.7, 3.7) | 3.4 (2.9, 4.3) | 3.8 (3.0, 5.0) | 4.1 (3.2, 6.2) | 4.1 (3.1, 6.7) | 3.9 (2.9, 6.6) |
|  | 90-90-90 | 3.9 (3.3, 4.9) | 4.0 (3.5, 5.1) | 4.1 (3.5, 5.2) | 4.1 (3.5, 5.4) | 4.2 (3.5, 5.8) | 4.4 (3.5, 6.2) | 4.5 (3.5, 7.1) | 4.3 (3.3, 7.4) |
|  | Current | 0.2 (0.1, 0.2) | 0.2 (0.2, 0.2) | 0.2 (0.2, 0.3) | 0.3 (0.3, 0.3) | 0.4 (0.4, 0.4) | 0.5 (0.5, 0.5) | 0.6 (0.5, 0.6) | 0.6 (0.6, 0.7) |
| **Philippines** | WHO 2013 | 0.2 (0.2, 0.2) | 0.2 (0.2, 0.2) | 0.3 (0.3, 0.3) | 0.4 (0.4, 0.4) | 0.5 (0.5, 0.5) | 0.6 (0.6, 0.6) | 0.7 (0.7, 0.8) | 0.8 (0.7, 0.8) |
|  | 90-90-90 | 0.2 (0.2, 0.2) | 0.3 (0.2, 0.3) | 0.3 (0.3, 0.3) | 0.4 (0.4, 0.4) | 0.5 (0.5, 0.5) | 0.6 (0.6, 0.7) | 0.7 (0.7, 0.8) | 0.9 (0.8, 0.9) |
|  | Current | 0.1 (0.0, 0.1) | 0.1 (0.0, 0.1) | 0.1 (0.1, 0.1) | 0.1 (0.1, 0.1) | 0.1 (0.1, 0.2) | 0.1 (0.1, 0.2) | 0.1 (0.1, 0.2) | 0.1 (0.1, 0.2) |
| **Sri Lanka** | WHO 2013 | 0.1 (0.0, 0.1) | 0.1 (0.1, 0.1) | 0.1 (0.1, 0.2) | 0.1 (0.1, 0.2) | 0.1 (0.1, 0.2) | 0.1 (0.1, 0.2) | 0.1 (0.1, 0.2) | 0.1 (0.1, 0.2) |
|  | 90-90-90 | 0.1 (0.1, 0.2) | 0.1 (0.1, 0.1) | 0.1 (0.1, 0.2) | 0.1 (0.1, 0.2) | 0.1 (0.1, 0.2) | 0.1 (0.1, 0.2) | 0.1 (0.1, 0.2) | 0.2 (0.1, 0.3) |
|  | Current | 6.9 (6.8, 6.9) | 6.3 (6.2, 6.4) | 5.6 (5.5, 5.7) | 4.9 (4.8, 5.0) | 4.2 (4.2, 4.3) | 3.6 (3.5, 3.7) | 2.9 (2.8, 3.0) | 2.3 (2.2, 2.4) |
| **Thailand** | WHO 2013 | 6.9 (6.8, 7.0) | 6.4 (6.3, 6.4) | 5.7 (5.6, 5.8) | 5.0 (4.9, 5.0) | 4.3 (4.2, 4.4) | 3.6 (3.5, 3.7) | 3.0 (2.8, 3.1) | 2.4 (2.2, 2.5) |
|  | 90-90-90 | 7.7 (7.6, 7.9) | 7.1 (7.0, 7.3) | 6.4 (6.3, 6.6) | 5.7 (5.6, 5.9) | 5.1 (5.0, 5.2) | 4.4 (4.3, 4.6) | 3.7 (3.5, 3.9) | 3.0 (2.8, 3.2) |
|  | Current | 4.4 (4.1, 4.7) | 4.7 (4.4, 5.0) | 4.7 (4.4, 5.0) | 4.6 (4.4, 4.9) | 4.5 (4.2, 4.8) | 4.3 (4.1, 4.6) | 4.0 (3.8, 4.3) | 3.9 (3.7, 4.2) |
| **Viet Nam** | WHO 2013 | 4.5 (4.2, 4.9) | 4.9 (4.6, 5.3) | 5.0 (4.6, 5.3) | 4.8 (4.5, 5.2) | 4.7 (4.4, 5.0) | 4.5 (4.2, 4.9) | 4.2 (3.9, 4.6) | 4.1 (3.8, 4.5) |
|  | 90-90-90 | 6.1 (5.7, 6.7) | 6.2 (5.8, 6.7) | 6.1 (5.6, 6.6) | 5.8 (5.4, 6.3) | 5.6 (5.2, 6.1) | 5.3 (4.9, 5.7) | 5.0 (4.6, 5.4) | 4.6 (4.3, 5.0) |
|  | Current | 4.2 (3.8, 4.6) | 4.2 (3.9, 4.6) | 4.4 (4.0, 4.9) | 4.5 (4.1, 5.1) | 4.7 (4.2, 5.4) | 4.9 (4.4, 5.7) | 5.0 (4.5, 6.1) | 5.1 (4.6, 6.2) |
| **Benin** | WHO 2013 | 4.6 (3.1, 5.5) | 4.6 (3.3, 5.8) | 4.6 (3.4, 6.3) | 4.7 (3.6, 7.9) | 4.9 (3.9, 9.4) | 5.2 (4.1, 11.2) | 5.4 (4.3, 12.1) | 5.5 (4.4, 12.4) |
|  | 90-90-90 | 7.9 (7.1, 8.6) | 7.3 (6.6, 8.0) | 7.0 (6.3, 7.7) | 6.9 (6.2, 7.7) | 6.7 (6.0, 7.6) | 6.6 (5.9, 7.7) | 6.6 (5.9, 7.7) | 6.6 (5.9, 7.9) |
|  | Current | 7.0 (5.8, 8.8) | 7.4 (6.2, 9.2) | 8.5 (7.0, 10.8) | 10.7 (8.6, 13.9) | 13.5 (10.6, 17.9) | 16.4 (12.6, 21.8) | 19.2 (14.5, 26.0) | 20.6 (15.4, 27.8) |
| **Burkina Faso** | WHO 2013 | 7.7 (6.4, 9.6) | 8.9 (7.4, 11.0) | 9.4 (7.8, 11.7) | 10.7 (8.8, 13.6) | 12.1 (9.7, 15.7) | 13.5 (10.4, 17.7) | 14.8 (11.2, 20.1) | 15.5 (11.5, 21.1) |
|  | 90-90-90 | 16.1 (13.5, 19.6) | 15.7 (13.1, 19.2) | 15.6 (13.1, 19.2) | 16.7 (13.8, 20.7) | 18.4 (15.1, 23.1) | 20.7 (16.8, 26.3) | 23.1 (18.6, 29.8) | 25.6 (19.8, 33.5) |
|  | Current | 33.1 (29.3, 40.3) | 32.3 (28.3, 38.7) | 32.1 (27.9, 38.6) | 32.5 (28.2, 39.4) | 33.7 (29.1, 40.6) | 36.1 (31.1, 43.7) | 40.5 (34.7, 49.5) | 43.3 (36.9, 53.2) |
| **Cameroon** | WHO 2013 | 37.6 (33.5, 45.0) | 41.1 (36.6, 48.3) | 40.5 (35.8, 47.8) | 40.7 (35.7, 48.2) | 41.4 (36.1, 49.2) | 42.8 (37.1, 50.8) | 45.1 (38.9, 54.2) | 46.5 (39.9, 56.1) |
|  | 90-90-90 | 75.0 (68.6, 83.6) | 74.6 (68.3, 82.8) | 73.8 (67.2, 82.9) | 73.2 (66.4, 84.0) | 72.0 (64.7, 83.3) | 71.2 (63.5, 83.2) | 71.9 (63.3, 85.3) | 75.8 (65.8, 91.1) |
| **Central African Republic** | Current | 5.3 (4.2, 6.4) | 4.9 (3.8, 5.9) | 4.7 (3.7, 5.6) | 4.7 (3.7, 5.6) | 4.9 (4.0, 5.7) | 5.0 (4.3, 5.9) | 5.2 (4.4, 6.1) | 5.2 (4.4, 6.1) |
|  | WHO 2013 | 6.2 (4.9, 7.4) | 6.6 (5.2, 7.9) | 6.2 (4.8, 7.5) | 6.1 (4.8, 7.2) | 6.1 (4.9, 7.2) | 6.1 (5.0, 7.1) | 6.1 (5.0, 7.2) | 6.1 (5.1, 7.2) |
|  | 90-90-90 | 14.1 (11.2, 17.4) | 13.2 (10.5, 16.3) | 12.7 (10.0, 15.4) | 12.2 (9.6, 14.7) | 11.9 (9.4, 14.2) | 11.5 (9.1, 13.5) | 11.2 (9.0, 13.1) | 10.9 (9.0, 12.8) |
|  | Current | 14.2 (11.8, 17.9) | 13.2 (10.9, 16.9) | 13.4 (11.3, 16.6) | 15.4 (13.3, 18.5) | 18.2 (15.3, 22.1) | 21.0 (17.1, 26.1) | 23.8 (18.9, 30.6) | 25.1 (19.8, 32.9) |
| **Chad** | WHO 2013 | 15.9 (13.2, 19.8) | 16.4 (13.6, 20.5) | 15.9 (13.2, 20.0) | 17.1 (14.1, 20.7) | 19.4 (15.7, 22.9) | 21.9 (17.3, 26.2) | 24.3 (18.9, 30.1) | 25.5 (19.6, 32.2) |
|  | 90-90-90 | 29.5 (25.1, 35.5) | 28.3 (23.9, 34.4) | 28.0 (24.3, 33.4) | 29.6 (25.8, 35.1) | 30.7 (26.4, 37.4) | 31.9 (26.6, 39.8) | 33.0 (26.5, 41.9) | 33.8 (26.5, 43.5) |
|  | Current | 4.2 (2.4, 4.9) | 4.3 (2.6, 5.0) | 4.5 (2.8, 5.1) | 4.6 (3.0, 5.2) | 4.6 (3.2, 5.2) | 4.6 (3.3, 5.3) | 4.6 (3.4, 5.4) | 4.6 (3.5, 5.3) |
| **Congo** | WHO 2013 | 5.0 (2.8, 5.8) | 5.9 (3.3, 6.8) | 5.9 (3.4, 6.8) | 5.9 (3.5, 6.9) | 5.8 (3.6, 6.7) | 5.8 (3.8, 6.7) | 5.7 (3.9, 6.7) | 5.7 (3.9, 6.7) |
|  | 90-90-90 | 10.0 (5.8, 11.9) | 9.8 (5.6, 11.8) | 9.6 (5.4, 11.4) | 9.4 (5.3, 11.1) | 9.2 (5.3, 10.7) | 9.1 (5.2, 10.5) | 8.9 (5.3, 10.3) | 8.8 (5.4, 10.2) |
|  | Current | 20.8 (18.4, 23.6) | 18.4 (16.3, 20.8) | 17.0 (15.1, 19.1) | 16.6 (15.0, 18.4) | 16.4 (15.1, 17.9) | 17.4 (16.1, 18.6) | 19.3 (17.9, 20.3) | 20.0 (18.8, 21.2) |
| **Cote d'Ivoire** | WHO 2013 | 24.3 (21.7, 27.6) | 25.4 (22.7, 28.7) | 23.1 (20.5, 26.1) | 21.4 (19.0, 24.0) | 20.0 (18.0, 22.3) | 19.3 (17.8, 21.3) | 19.7 (18.5, 21.4) | 19.9 (18.8, 21.7) |
|  | 90-90-90 | 60.5 (53.7, 67.9) | 54.7 (49.2, 61.3) | 49.8 (45.1, 55.9) | 45.6 (41.3, 51.1) | 40.9 (37.1, 46.0) | 38.6 (35.3, 42.5) | 36.4 (33.4, 40.0) | 34.1 (31.4, 37.5) |
| **Democratic Republic of Congo** | Current | 31.5 (27.2, 36.5) | 33.2 (28.3, 39.0) | 36.7 (31.1, 43.5) | 46.4 (38.4, 55.8) | 60.6 (49.4, 73.8) | 75.0 (61.5, 92.5) | 88.4 (72.2, 109.2) | 94.7 (77.3, 117.0) |
|  | WHO 2013 | 34.6 (30.6, 39.9) | 38.5 (33.6, 44.8) | 40.2 (34.4, 47.2) | 44.7 (37.9, 53.1) | 50.7 (42.4, 60.4) | 56.1 (46.8, 67.5) | 61.3 (50.7, 75.2) | 63.7 (52.6, 79.0) |
|  | 90-90-90 | 58.6 (50.2, 68.1) | 58.0 (49.7, 67.0) | 59.8 (50.5, 70.2) | 65.2 (54.9, 77.3) | 75.5 (63.2, 91.0) | 88.4 (72.4, 106.7) | 100.9 (82.9, 123.8) | 112.2 (91.8, 138.2) |
| **Equatorial Guinea** | Current | 1.2 (0.9, 1.7) | 1.3 (1.0, 1.8) | 1.4 (1.1, 2.0) | 1.6 (1.2, 2.1) | 1.7 (1.2, 2.3) | 1.8 (1.3, 2.4) | 1.7 (1.3, 2.4) | 1.7 (1.3, 2.3) |
|  | WHO 2013 | 1.5 (1.1, 2.0) | 1.8 (1.4, 2.4) | 1.9 (1.5, 2.6) | 2.0 (1.5, 2.8) | 2.1 (1.6, 2.9) | 2.2 (1.6, 3.1) | 2.2 (1.6, 3.0) | 2.1 (1.5, 2.9) |
|  | 90-90-90 | 2.3 (1.9, 3.0) | 2.6 (2.0, 3.3) | 2.7 (2.1, 3.5) | 2.9 (2.3, 3.8) | 3.1 (2.4, 4.1) | 3.2 (2.5, 4.4) | 3.4 (2.5, 4.6) | 3.3 (2.5, 4.6) |
|  | Current | 1.8 (1.4, 2.2) | 1.8 (1.5, 2.3) | 1.9 (1.6, 2.3) | 1.9 (1.7, 2.3) | 2.0 (1.8, 2.4) | 2.1 (1.9, 2.5) | 2.1 (2.0, 2.5) | 2.1 (2.0, 2.5) |
| **Gabon** | WHO 2013 | 2.0 (1.6, 2.5) | 2.2 (1.7, 2.7) | 2.2 (1.7, 2.7) | 2.2 (1.8, 2.7) | 2.3 (1.9, 2.7) | 2.3 (2.0, 2.8) | 2.3 (2.1, 2.8) | 2.3 (2.1, 2.8) |
|  | 90-90-90 | 4.0 (3.4, 5.0) | 4.0 (3.4, 5.0) | 4.0 (3.4, 5.0) | 4.0 (3.4, 4.9) | 4.0 (3.4, 4.9) | 3.9 (3.4, 4.8) | 3.8 (3.4, 4.7) | 3.7 (3.3, 4.6) |
|  | Current | 0.9 (0.5, 1.4) | 0.9 (0.5, 1.5) | 1.0 (0.5, 1.7) | 1.1 (0.5, 2.1) | 1.3 (0.7, 2.5) | 1.7 (0.8, 3.5) | 2.1 (1.0, 4.5) | 2.3 (1.2, 5.1) |
| **Gambia** | WHO 2013 | 1.0 (0.8, 1.3) | 1.1 (0.8, 1.4) | 1.1 (0.8, 1.5) | 1.2 (0.9, 1.7) | 1.3 (0.9, 2.1) | 1.5 (1.0, 2.4) | 1.6 (1.1, 2.9) | 1.7 (1.1, 3.1) |
|  | 90-90-90 | 1.6 (1.0, 2.3) | 1.7 (1.0, 2.4) | 1.7 (1.0, 2.7) | 1.8 (1.0, 3.0) | 2.0 (1.1, 3.4) | 2.3 (1.2, 4.1) | 2.7 (1.3, 5.1) | 3.1 (1.5, 6.0) |
|  | Current | 10.3 (8.1, 16.3) | 10.7 (8.6, 15.8) | 11.8 (9.4, 17.4) | 12.1 (9.4, 18.1) | 12.6 (9.5, 19.2) | 13.1 (9.9, 20.2) | 13.5 (10.0, 21.3) | 13.8 (10.1, 21.8) |
| **Ghana** | WHO 2013 | 12.0 (9.3, 18.9) | 12.4 (9.8, 18.4) | 11.9 (9.4, 17.3) | 12.2 (9.4, 18.1) | 12.7 (9.6, 19.1) | 13.1 (9.9, 20.1) | 13.6 (10.1, 21.2) | 13.9 (10.1, 21.8) |
|  | 90-90-90 | 28.4 (21.1, 40.6) | 26.3 (19.7, 37.5) | 25.0 (18.5, 35.9) | 24.1 (17.4, 35.7) | 23.1 (16.6, 35.0) | 22.1 (15.5, 34.7) | 21.2 (14.5, 34.1) | 20.5 (13.7, 33.2) |
|  | Current | 5.9 (4.9, 6.8) | 5.8 (4.8, 6.7) | 6.0 (5.0, 6.9) | 6.9 (5.6, 8.1) | 8.3 (6.7, 10.0) | 9.8 (7.8, 11.9) | 11.4 (9.0, 13.7) | 12.2 (9.7, 14.7) |
| **Guinea** | WHO 2013 | 6.7 (5.6, 7.7) | 7.3 (6.1, 8.3) | 7.3 (6.0, 8.4) | 7.6 (6.3, 8.8) | 8.1 (6.7, 9.5) | 8.6 (7.0, 10.2) | 9.3 (7.3, 11.0) | 9.6 (7.5, 11.4) |
|  | 90-90-90 | 11.7 (9.7, 13.6) | 11.7 (9.7, 13.6) | 12.0 (9.8, 13.8) | 12.6 (10.4, 14.4) | 13.6 (11.2, 15.7) | 15.0 (12.1, 17.6) | 16.4 (13.0, 19.5) | 17.8 (14.0, 21.3) |
|  | Current | 2.6 (2.1, 3.3) | 2.7 (2.1, 3.6) | 2.8 (2.1, 3.9) | 3.1 (2.2, 4.4) | 3.4 (2.4, 4.9) | 3.8 (2.6, 5.6) | 4.3 (2.9, 6.6) | 4.6 (3.1, 7.2) |
| **Guinea-Bissau** | WHO 2013 | 3.0 (2.5, 3.7) | 3.6 (2.9, 4.5) | 3.7 (2.9, 4.8) | 3.9 (3.0, 5.2) | 4.2 (3.1, 5.9) | 4.6 (3.3, 6.4) | 5.1 (3.5, 7.4) | 5.4 (3.7, 8.0) |
|  | 90-90-90 | 5.4 (4.8, 6.0) | 5.6 (4.9, 6.2) | 5.6 (4.9, 6.5) | 5.7 (4.8, 6.9) | 5.9 (4.8, 7.3) | 6.1 (4.8, 7.9) | 6.5 (4.9, 8.7) | 7.0 (5.1, 9.8) |
|  | Current | 1.6 (1.3, 1.9) | 1.6 (1.3, 1.9) | 1.6 (1.3, 2.0) | 1.7 (1.4, 2.0) | 1.7 (1.4, 2.1) | 1.7 (1.4, 2.1) | 1.7 (1.4, 2.1) | 1.6 (1.3, 2.1) |
| **Liberia** | WHO 2013 | 1.9 (1.5, 2.3) | 2.1 (1.7, 2.5) | 2.0 (1.7, 2.5) | 2.0 (1.6, 2.5) | 2.0 (1.6, 2.5) | 2.0 (1.6, 2.5) | 1.9 (1.6, 2.4) | 1.9 (1.5, 2.4) |
|  | 90-90-90 | 4.5 (3.8, 5.6) | 4.3 (3.6, 5.3) | 4.1 (3.4, 5.0) | 3.9 (3.3, 4.8) | 3.7 (3.1, 4.6) | 3.6 (3.0, 4.4) | 3.5 (2.9, 4.3) | 3.3 (2.7, 4.1) |
|  | Current | 7.1 (4.4, 10.8) | 7.2 (4.7, 11.3) | 7.4 (4.7, 11.7) | 7.9 (5.0, 14.4) | 8.9 (5.1, 18.0) | 10.2 (6.0, 26.3) | 11.6 (6.4, 32.8) | 12.2 (6.6, 36.1) |
| **Mali** | WHO 2013 | 7.8 (6.1, 10.7) | 8.6 (6.8, 12.0) | 8.6 (6.8, 12.3) | 8.9 (6.8, 13.1) | 9.3 (6.9, 14.1) | 9.6 (6.8, 14.9) | 9.8 (6.7, 15.6) | 9.8 (6.7, 16.2) |
|  | 90-90-90 | 14.8 (10.9, 20.2) | 14.6 (10.3, 20.0) | 14.4 (9.9, 20.4) | 14.5 (9.7, 21.5) | 14.5 (9.8, 24.0) | 15.1 (9.6, 27.4) | 16.0 (9.6, 34.8) | 16.9 (10.0, 41.2) |
|  | Current | 0.4 (0.3, 0.5) | 0.3 (0.2, 0.4) | 0.2 (0.2, 0.3) | 0.2 (0.2, 0.3) | 0.2 (0.2, 0.3) | 0.2 (0.1, 0.3) | 0.2 (0.1, 0.2) | 0.2 (0.1, 0.2) |
| **Mauritania** | WHO 2013 | 0.4 (0.3, 0.6) | 0.4 (0.3, 0.6) | 0.4 (0.3, 0.5) | 0.3 (0.3, 0.4) | 0.3 (0.2, 0.4) | 0.3 (0.2, 0.4) | 0.3 (0.2, 0.3) | 0.2 (0.2, 0.3) |
|  | 90-90-90 | 1.1 (0.8, 1.5) | 1.0 (0.8, 1.4) | 0.9 (0.7, 1.3) | 0.9 (0.6, 1.2) | 0.8 (0.6, 1.1) | 0.7 (0.5, 1.0) | 0.6 (0.5, 0.9) | 0.6 (0.4, 0.8) |
|  | Current | 2.4 (1.9, 3.1) | 2.2 (1.8, 2.8) | 2.0 (1.7, 2.5) | 2.0 (1.7, 2.4) | 1.9 (1.6, 2.4) | 1.9 (1.6, 2.4) | 1.9 (1.6, 2.5) | 1.9 (1.6, 2.6) |
| **Niger** | WHO 2013 | 2.8 (2.3, 3.6) | 3.1 (2.6, 3.9) | 3.0 (2.5, 3.7) | 3.0 (2.5, 3.7) | 3.1 (2.6, 3.8) | 3.2 (2.7, 4.1) | 3.4 (2.8, 4.4) | 3.5 (2.8, 4.6) |
|  | 90-90-90 | 6.7 (5.5, 8.6) | 6.5 (5.4, 8.2) | 6.2 (5.1, 7.7) | 5.9 (4.9, 7.3) | 5.6 (4.6, 6.9) | 5.3 (4.4, 6.5) | 4.9 (4.1, 6.2) | 4.6 (3.8, 6.0) |
|  | Current | 200.1 (178.8, 223.9) | 189.7 (170.7, 209.8) | 192.5 (173.1, 214.1) | 210.5 (189.3, 235.8) | 229.4 (205.5, 258.3) | 246.7 (220.3, 278.8) | 262.7 (233.2, 298.1) | 277.2 (246.1, 317.0) |
| **Nigeria** | WHO 2013 | 222.6 (198.8, 248.5) | 235.4 (210.0, 259.5) | 241.8 (215.7, 269.4) | 265.9 (236.8, 297.6) | 291.7 (259.5, 327.0) | 315.4 (280.5, 355.8) | 338.2 (299.2, 384.8) | 359.8 (316.6, 413.5) |
|  | 90-90-90 | 374.6 (337.2, 416.1) | 375.1 (339.2, 416.7) | 381.0 (341.5, 422.6) | 390.8 (349.7, 437.4) | 396.3 (352.3, 441.7) | 399.0 (353.5, 445.5) | 400.0 (353.1, 452.0) | 399.1 (352.6, 454.9) |
|  | Current | 2.7 (1.7, 3.2) | 2.5 (1.7, 3.0) | 2.4 (1.7, 2.7) | 2.4 (1.9, 2.7) | 2.7 (2.1, 2.9) | 2.9 (2.3, 3.1) | 3.1 (2.5, 3.3) | 3.1 (2.5, 3.4) |
| **Senegal** | WHO 2013 | 3.0 (1.9, 3.6) | 3.1 (2.0, 3.6) | 2.9 (2.0, 3.4) | 2.8 (2.1, 3.3) | 2.9 (2.3, 3.3) | 3.0 (2.4, 3.4) | 3.1 (2.5, 3.5) | 3.1 (2.5, 3.5) |
|  | 90-90-90 | 5.4 (3.0, 6.4) | 5.2 (3.0, 6.3) | 5.1 (3.0, 6.1) | 5.1 (3.1, 6.0) | 5.1 (3.3, 6.0) | 5.1 (3.4, 5.9) | 5.0 (3.4, 5.8) | 4.9 (3.3, 5.7) |
|  | Current | 2.3 (1.8, 3.3) | 2.2 (1.7, 3.2) | 2.2 (1.7, 3.1) | 2.3 (1.8, 3.1) | 2.6 (2.0, 3.3) | 2.9 (2.1, 3.7) | 3.3 (2.3, 4.3) | 3.5 (2.4, 4.7) |
| **Sierra Leone** | WHO 2013 | 2.6 (2.0, 3.7) | 2.8 (2.2, 4.0) | 2.7 (2.1, 3.9) | 2.8 (2.2, 3.9) | 3.0 (2.4, 4.0) | 3.2 (2.4, 4.2) | 3.4 (2.5, 4.4) | 3.5 (2.6, 4.6) |
|  | 90-90-90 | 4.5 (3.8, 5.6) | 4.5 (3.7, 5.8) | 4.5 (3.7, 5.8) | 4.7 (3.9, 5.8) | 4.8 (3.8, 6.0) | 5.0 (3.8, 6.3) | 5.2 (3.8, 6.6) | 5.4 (3.8, 7.2) |
|  | Current | 7.1 (3.9, 12.7) | 7.7 (4.4, 14.0) | 7.5 (4.6, 12.9) | 8.7 (5.4, 13.1) | 9.6 (5.9, 13.5) | 9.3 (5.8, 13.0) | 8.9 (5.5, 12.7) | 8.7 (5.4, 12.5) |
| **Togo** | WHO 2013 | 7.1 (3.8, 12.7) | 7.7 (4.2, 13.9) | 7.4 (4.4, 12.9) | 8.6 (5.1, 12.9) | 9.3 (5.4, 13.1) | 8.8 (5.1, 12.3) | 8.2 (4.8, 11.3) | 7.9 (4.6, 10.8) |
|  | 90-90-90 | 18.1 (10.1, 32.2) | 16.9 (9.7, 30.3) | 15.7 (9.2, 28.2) | 16.9 (9.8, 27.5) | 16.1 (9.4, 26.3) | 14.9 (8.6, 24.1) | 13.8 (8.2, 22.1) | 12.7 (7.5, 20.5) |
|  | Current | 0.0 (0.0, 0.0) | 0.0 (0.0, 0.0) | 0.0 (0.0, 0.0) | 0.0 (0.0, 0.0) | 0.0 (0.0, 0.0) | 0.0 (0.0, 0.0) | 0.0 (0.0, 0.1) | 0.0 (0.0, 0.1) |
| **Armenia** | WHO 2013 | 0.0 (0.0, 0.0) | 0.0 (0.0, 0.0) | 0.0 (0.0, 0.0) | 0.0 (0.0, 0.0) | 0.0 (0.0, 0.0) | 0.0 (0.0, 0.0) | 0.0 (0.0, 0.1) | 0.0 (0.0, 0.1) |
|  | 90-90-90 | 0.0 (0.0, 0.0) | 0.0 (0.0, 0.0) | 0.0 (0.0, 0.0) | 0.0 (0.0, 0.0) | 0.0 (0.0, 0.0) | 0.0 (0.0, 0.0) | 0.0 (0.0, 0.0) | 0.0 (0.0, 0.1) |
|  | Current | 0.1 (0.1, 0.2) | 0.2 (0.1, 0.2) | 0.2 (0.1, 0.2) | 0.2 (0.2, 0.2) | 0.2 (0.2, 0.3) | 0.2 (0.2, 0.3) | 0.2 (0.2, 0.3) | 0.3 (0.2, 0.3) |
| **Azerbaijan** | WHO 2013 | 0.1 (0.1, 0.2) | 0.2 (0.1, 0.2) | 0.2 (0.2, 0.2) | 0.2 (0.2, 0.3) | 0.2 (0.2, 0.3) | 0.3 (0.2, 0.3) | 0.3 (0.2, 0.3) | 0.3 (0.2, 0.3) |
|  | 90-90-90 | 0.2 (0.1, 0.2) | 0.2 (0.2, 0.2) | 0.2 (0.2, 0.3) | 0.2 (0.2, 0.3) | 0.3 (0.2, 0.3) | 0.3 (0.2, 0.4) | 0.3 (0.3, 0.4) | 0.3 (0.3, 0.4) |
|  | Current | 0.1 (0.1, 0.2) | 0.2 (0.2, 0.2) | 0.3 (0.2, 0.3) | 0.3 (0.3, 0.4) | 0.3 (0.3, 0.4) | 0.4 (0.3, 0.5) | 0.4 (0.3, 0.5) | 0.4 (0.3, 0.5) |
| **Belarus** | WHO 2013 | 0.1 (0.1, 0.2) | 0.2 (0.2, 0.2) | 0.3 (0.2, 0.3) | 0.3 (0.3, 0.4) | 0.4 (0.3, 0.4) | 0.4 (0.3, 0.5) | 0.4 (0.4, 0.5) | 0.4 (0.4, 0.6) |
|  | 90-90-90 | 0.1 (0.1, 0.2) | 0.2 (0.2, 0.2) | 0.2 (0.2, 0.3) | 0.3 (0.3, 0.3) | 0.3 (0.3, 0.4) | 0.4 (0.3, 0.5) | 0.4 (0.4, 0.5) | 0.5 (0.4, 0.6) |
|  | Current | 0.0 (0.0, 0.0) | 0.0 (0.0, 0.0) | 0.0 (0.0, 0.0) | 0.0 (0.0, 0.0) | 0.0 (0.0, 0.0) | 0.0 (0.0, 0.0) | 0.1 (0.1, 0.1) | 0.1 (0.1, 0.1) |
| **Bulgaria** | WHO 2013 | 0.0 (0.0, 0.0) | 0.0 (0.0, 0.0) | 0.0 (0.0, 0.0) | 0.0 (0.0, 0.0) | 0.0 (0.0, 0.1) | 0.1 (0.1, 0.1) | 0.1 (0.1, 0.1) | 0.1 (0.1, 0.1) |
|  | 90-90-90 | 0.0 (0.0, 0.0) | 0.0 (0.0, 0.0) | 0.0 (0.0, 0.0) | 0.0 (0.0, 0.0) | 0.0 (0.0, 0.0) | 0.1 (0.1, 0.1) | 0.1 (0.1, 0.1) | 0.1 (0.1, 0.1) |
|  | Current | 0.1 (0.1, 0.1) | 0.1 (0.1, 0.1) | 0.1 (0.1, 0.1) | 0.1 (0.1, 0.1) | 0.1 (0.1, 0.1) | 0.1 (0.1, 0.1) | 0.1 (0.1, 0.1) | 0.1 (0.1, 0.1) |
| **Georgia** | WHO 2013 | 0.1 (0.1, 0.1) | 0.1 (0.1, 0.1) | 0.1 (0.1, 0.1) | 0.1 (0.1, 0.1) | 0.1 (0.1, 0.1) | 0.1 (0.1, 0.1) | 0.1 (0.1, 0.1) | 0.1 (0.1, 0.1) |
|  | 90-90-90 | 0.1 (0.1, 0.1) | 0.1 (0.1, 0.1) | 0.1 (0.1, 0.1) | 0.1 (0.1, 0.1) | 0.1 (0.1, 0.1) | 0.1 (0.1, 0.1) | 0.1 (0.1, 0.1) | 0.1 (0.1, 0.1) |
|  | Current | 0.2 (0.2, 0.3) | 0.2 (0.2, 0.3) | 0.2 (0.2, 0.3) | 0.2 (0.2, 0.3) | 0.2 (0.2, 0.2) | 0.2 (0.2, 0.2) | 0.2 (0.2, 0.2) | 0.2 (0.2, 0.2) |
| **Kazakhstan** | WHO 2013 | 0.3 (0.3, 0.3) | 0.3 (0.3, 0.3) | 0.3 (0.3, 0.3) | 0.3 (0.3, 0.3) | 0.3 (0.3, 0.3) | 0.3 (0.3, 0.3) | 0.3 (0.2, 0.3) | 0.2 (0.2, 0.3) |
|  | 90-90-90 | 0.7 (0.7, 0.7) | 0.7 (0.7, 0.8) | 0.8 (0.8, 0.8) | 0.9 (0.9, 0.9) | 1.0 (0.9, 1.0) | 1.0 (1.0, 1.1) | 1.1 (1.1, 1.1) | 1.1 (1.1, 1.2) |
|  | Current | 0.1 (0.1, 0.1) | 0.1 (0.1, 0.1) | 0.1 (0.1, 0.1) | 0.1 (0.1, 0.1) | 0.1 (0.1, 0.2) | 0.1 (0.1, 0.2) | 0.1 (0.1, 0.2) | 0.2 (0.1, 0.2) |
| **Kyrgyzstan** | WHO 2013 | 0.1 (0.1, 0.1) | 0.1 (0.1, 0.1) | 0.1 (0.1, 0.1) | 0.1 (0.1, 0.1) | 0.1 (0.1, 0.2) | 0.1 (0.1, 0.2) | 0.1 (0.1, 0.2) | 0.2 (0.1, 0.2) |
|  | 90-90-90 | 0.1 (0.1, 0.2) | 0.1 (0.1, 0.2) | 0.1 (0.1, 0.2) | 0.1 (0.1, 0.2) | 0.2 (0.1, 0.2) | 0.2 (0.1, 0.2) | 0.2 (0.1, 0.2) | 0.2 (0.1, 0.2) |
| **Republic of Moldova** | Current | 0.1 (0.1, 0.1) | 0.1 (0.1, 0.1) | 0.1 (0.1, 0.1) | 0.1 (0.1, 0.1) | 0.2 (0.2, 0.2) | 0.2 (0.2, 0.2) | 0.3 (0.3, 0.3) | 0.3 (0.3, 0.3) |
|  | WHO 2013 | 0.1 (0.1, 0.1) | 0.1 (0.1, 0.1) | 0.1 (0.1, 0.1) | 0.2 (0.1, 0.2) | 0.2 (0.2, 0.2) | 0.2 (0.2, 0.2) | 0.3 (0.3, 0.3) | 0.4 (0.4, 0.4) |
|  | 90-90-90 | 0.2 (0.2, 0.2) | 0.2 (0.2, 0.2) | 0.2 (0.2, 0.2) | 0.2 (0.2, 0.2) | 0.2 (0.2, 0.2) | 0.2 (0.2, 0.3) | 0.3 (0.3, 0.3) | 0.4 (0.4, 0.4) |
|  | Current | 0.3 (0.3, 0.3) | 0.3 (0.3, 0.3) | 0.3 (0.3, 0.3) | 0.3 (0.3, 0.3) | 0.3 (0.3, 0.3) | 0.3 (0.3, 0.3) | 0.3 (0.3, 0.4) | 0.4 (0.4, 0.4) |
| **Romania** | WHO 2013 | 0.3 (0.3, 0.3) | 0.3 (0.3, 0.3) | 0.3 (0.3, 0.3) | 0.3 (0.3, 0.3) | 0.4 (0.4, 0.4) | 0.4 (0.4, 0.4) | 0.5 (0.5, 0.5) | 0.5 (0.5, 0.5) |
|  | 90-90-90 | 0.5 (0.5, 0.5) | 0.4 (0.4, 0.4) | 0.4 (0.4, 0.4) | 0.4 (0.4, 0.4) | 0.4 (0.4, 0.4) | 0.4 (0.4, 0.4) | 0.5 (0.5, 0.5) | 0.6 (0.6, 0.6) |
|  | Current | 9.2 (8.9, 9.7) | 9.7 (9.4, 10.2) | 10.3 (10.0, 10.8) | 10.9 (10.6, 11.3) | 11.4 (11.1, 11.9) | 11.9 (11.6, 12.2) | 12.3 (12.0, 12.6) | 12.5 (12.2, 12.9) |
| **Russia** | WHO 2013 | 10.4 (10.0, 10.8) | 12.0 (11.6, 12.5) | 12.4 (12.0, 13.0) | 13.1 (12.7, 13.6) | 13.8 (13.4, 14.4) | 14.7 (14.2, 15.3) | 15.6 (15.1, 16.3) | 16.1 (15.5, 16.9) |
|  | 90-90-90 | 13.3 (12.9, 13.7) | 13.8 (13.5, 14.2) | 14.4 (14.0, 14.9) | 15.2 (14.7, 15.7) | 16.0 (15.5, 16.6) | 16.8 (16.2, 17.5) | 17.7 (17.1, 18.5) | 18.8 (18.1, 19.8) |
|  | Current | 0.0 (0.0, 0.0) | 0.0 (0.0, 0.0) | 0.0 (0.0, 0.0) | 0.0 (0.0, 0.0) | 0.0 (0.0, 0.0) | 0.1 (0.0, 0.1) | 0.1 (0.1, 0.1) | 0.1 (0.1, 0.1) |
| **Serbia** | WHO 2013 | 0.0 (0.0, 0.0) | 0.0 (0.0, 0.0) | 0.0 (0.0, 0.0) | 0.0 (0.0, 0.0) | 0.1 (0.1, 0.1) | 0.1 (0.1, 0.1) | 0.1 (0.1, 0.1) | 0.1 (0.1, 0.1) |
|  | 90-90-90 | 0.1 (0.1, 0.1) | 0.1 (0.1, 0.1) | 0.1 (0.1, 0.1) | 0.1 (0.1, 0.1) | 0.1 (0.1, 0.1) | 0.1 (0.1, 0.1) | 0.1 (0.1, 0.1) | 0.1 (0.1, 0.1) |
|  | Current | 0.4 (0.3, 0.9) | 0.5 (0.3, 1.0) | 0.6 (0.3, 1.0) | 0.7 (0.4, 1.0) | 0.7 (0.4, 1.0) | 0.8 (0.5, 1.1) | 0.9 (0.6, 1.2) | 0.9 (0.6, 1.3) |
| **Tajikistan** | WHO 2013 | 0.5 (0.3, 1.0) | 0.6 (0.4, 1.2) | 0.7 (0.4, 1.2) | 0.8 (0.4, 1.2) | 0.8 (0.5, 1.2) | 0.9 (0.5, 1.2) | 0.9 (0.6, 1.3) | 1.0 (0.7, 1.4) |
|  | 90-90-90 | 0.9 (0.6, 1.6) | 0.9 (0.6, 1.7) | 1.0 (0.6, 1.7) | 1.0 (0.6, 1.6) | 1.1 (0.6, 1.6) | 1.1 (0.7, 1.6) | 1.2 (0.7, 1.7) | 1.2 (0.8, 1.8) |
|  | Current | 3.0 (2.7, 3.5) | 3.2 (2.9, 3.7) | 3.4 (3.0, 3.9) | 3.6 (3.1, 4.1) | 3.6 (3.2, 4.3) | 3.6 (3.1, 4.3) | 3.5 (3.0, 4.2) | 3.4 (2.9, 4.2) |
| **Ukraine** | WHO 2013 | 3.4 (3.0, 3.8) | 3.8 (3.4, 4.3) | 3.8 (3.4, 4.5) | 3.9 (3.4, 4.5) | 3.9 (3.3, 4.6) | 3.8 (3.2, 4.6) | 3.7 (3.1, 4.5) | 3.6 (3.0, 4.4) |
|  | 90-90-90 | 5.7 (5.1, 6.6) | 5.7 (5.0, 6.6) | 5.6 (4.9, 6.5) | 5.5 (4.8, 6.5) | 5.4 (4.6, 6.4) | 5.1 (4.4, 6.1) | 4.7 (4.0, 5.7) | 4.4 (3.6, 5.4) |
|  | Current | 3.2 (3.0, 3.3) | 3.8 (3.6, 3.9) | 4.2 (4.1, 4.4) | 4.6 (4.4, 4.8) | 4.6 (4.5, 4.9) | 4.4 (4.2, 4.7) | 4.0 (3.9, 4.2) | 3.8 (3.6, 4.0) |
| **Uzbekistan** | WHO 2013 | 3.2 (3.1, 3.4) | 3.8 (3.6, 4.0) | 4.2 (4.1, 4.4) | 4.6 (4.4, 4.8) | 4.7 (4.5, 4.9) | 4.5 (4.3, 4.7) | 4.1 (3.9, 4.3) | 3.9 (3.7, 4.1) |
|  | 90-90-90 | 4.1 (3.9, 4.2) | 4.4 (4.3, 4.6) | 4.9 (4.7, 5.1) | 5.2 (5.0, 5.5) | 5.3 (5.1, 5.5) | 5.0 (4.8, 5.3) | 4.6 (4.4, 4.8) | 4.2 (4.0, 4.4) |
|  | Current | 0.1 (0.1, 0.2) | 0.1 (0.1, 0.1) | 0.1 (0.1, 0.1) | 0.1 (0.1, 0.1) | 0.1 (0.1, 0.1) | 0.1 (0.1, 0.1) | 0.1 (0.1, 0.1) | 0.1 (0.1, 0.1) |
| **Bahamas** | WHO 2013 | 0.1 (0.1, 0.2) | 0.1 (0.1, 0.1) | 0.1 (0.1, 0.1) | 0.1 (0.1, 0.1) | 0.1 (0.1, 0.1) | 0.1 (0.1, 0.1) | 0.1 (0.1, 0.1) | 0.1 (0.1, 0.1) |
|  | 90-90-90 | 0.2 (0.1, 0.2) | 0.1 (0.1, 0.2) | 0.1 (0.1, 0.1) | 0.1 (0.1, 0.1) | 0.1 (0.1, 0.1) | 0.1 (0.1, 0.1) | 0.1 (0.1, 0.1) | 0.1 (0.1, 0.1) |
|  | Current | 0.0 (0.0, 0.0) | 0.0 (0.0, 0.0) | 0.0 (0.0, 0.0) | 0.0 (0.0, 0.0) | 0.0 (0.0, 0.0) | 0.0 (0.0, 0.0) | 0.0 (0.0, 0.0) | 0.0 (0.0, 0.0) |
| **Barbados** | WHO 2013 | 0.0 (0.0, 0.0) | 0.0 (0.0, 0.0) | 0.0 (0.0, 0.0) | 0.0 (0.0, 0.0) | 0.0 (0.0, 0.0) | 0.0 (0.0, 0.0) | 0.0 (0.0, 0.1) | 0.0 (0.0, 0.1) |
|  | 90-90-90 | 0.0 (0.0, 0.0) | 0.0 (0.0, 0.0) | 0.0 (0.0, 0.0) | 0.0 (0.0, 0.0) | 0.0 (0.0, 0.0) | 0.0 (0.0, 0.0) | 0.0 (0.0, 0.0) | 0.0 (0.0, 0.0) |
|  | Current | 0.1 (0.1, 0.2) | 0.1 (0.1, 0.2) | 0.1 (0.1, 0.2) | 0.1 (0.1, 0.2) | 0.1 (0.1, 0.2) | 0.1 (0.1, 0.2) | 0.1 (0.1, 0.2) | 0.1 (0.1, 0.2) |
| **Belize** | WHO 2013 | 0.1 (0.1, 0.2) | 0.1 (0.1, 0.1) | 0.1 (0.1, 0.1) | 0.1 (0.1, 0.1) | 0.1 (0.1, 0.1) | 0.1 (0.1, 0.1) | 0.1 (0.1, 0.1) | 0.1 (0.1, 0.1) |
|  | 90-90-90 | 0.2 (0.2, 0.3) | 0.2 (0.2, 0.2) | 0.2 (0.2, 0.2) | 0.2 (0.1, 0.2) | 0.2 (0.1, 0.2) | 0.2 (0.1, 0.2) | 0.2 (0.1, 0.2) | 0.1 (0.1, 0.2) |
|  | Current | 0.2 (0.1, 2.3) | 0.2 (0.1, 2.1) | 0.2 (0.1, 1.9) | 0.2 (0.1, 1.7) | 0.2 (0.1, 1.4) | 0.2 (0.1, 1.3) | 0.2 (0.1, 1.2) | 0.2 (0.1, 1.1) |
| **Bolivia** | WHO 2013 | 0.3 (0.1, 2.8) | 0.3 (0.2, 3.1) | 0.3 (0.2, 2.8) | 0.3 (0.2, 2.5) | 0.2 (0.1, 2.1) | 0.2 (0.1, 2.0) | 0.2 (0.1, 1.8) | 0.2 (0.1, 1.5) |
|  | 90-90-90 | 0.9 (0.5, 7.8) | 0.8 (0.5, 7.4) | 0.7 (0.4, 6.9) | 0.6 (0.4, 6.2) | 0.6 (0.3, 5.5) | 0.5 (0.3, 5.0) | 0.4 (0.2, 4.4) | 0.4 (0.2, 3.9) |
|  | Current | 0.1 (0.1, 0.8) | 0.1 (0.1, 0.6) | 0.1 (0.1, 0.4) | 0.1 (0.1, 0.3) | 0.1 (0.0, 0.2) | 0.0 (0.0, 0.1) | 0.1 (0.0, 0.2) | 0.1 (0.0, 0.1) |
| **Cuba** | WHO 2013 | 0.0 (0.0, 0.0) | 0.0 (0.0, 0.0) | 0.0 (0.0, 0.0) | 0.0 (0.0, 0.0) | 0.0 (0.0, 0.0) | 0.0 (0.0, 0.0) | 0.0 (0.0, 0.0) | 0.0 (0.0, 0.0) |
|  | 90-90-90 | 0.2 (0.1, 0.6) | 0.2 (0.1, 0.5) | 0.1 (0.1, 0.5) | 0.1 (0.1, 0.5) | 0.1 (0.1, 0.5) | 0.1 (0.1, 0.5) | 0.1 (0.1, 0.5) | 0.1 (0.1, 0.4) |
| **Dominican Republic** | Current | 1.3 (1.3, 1.4) | 1.3 (1.3, 1.4) | 1.3 (1.2, 1.4) | 1.2 (1.2, 1.3) | 1.2 (1.1, 1.2) | 1.0 (1.0, 1.1) | 0.9 (0.9, 1.0) | 0.9 (0.9, 1.0) |
|  | WHO 2013 | 1.4 (1.4, 1.5) | 1.5 (1.4, 1.6) | 1.4 (1.3, 1.5) | 1.3 (1.3, 1.5) | 1.2 (1.2, 1.3) | 1.1 (1.1, 1.2) | 1.0 (0.9, 1.1) | 0.9 (0.9, 1.0) |
|  | 90-90-90 | 2.5 (2.2, 3.4) | 2.3 (2.0, 3.0) | 2.1 (1.9, 2.7) | 1.9 (1.7, 2.4) | 1.7 (1.6, 2.1) | 1.5 (1.4, 1.9) | 1.4 (1.3, 1.6) | 1.3 (1.1, 1.5) |
|  | Current | 2.6 (0.9, 16.7) | 2.8 (0.9, 20.0) | 2.9 (0.8, 22.4) | 3.0 (0.8, 23.4) | 3.1 (0.7, 25.5) | 3.3 (0.7, 28.5) | 3.4 (0.7, 31.0) | 3.5 (0.8, 32.3) |
| **Guatemala** | WHO 2013 | 2.7 (0.9, 17.0) | 2.9 (0.9, 20.7) | 3.0 (0.8, 23.2) | 3.1 (0.8, 24.3) | 3.2 (0.8, 26.4) | 3.4 (0.7, 29.6) | 3.5 (0.7, 32.2) | 3.6 (0.8, 33.7) |
|  | 90-90-90 | 3.3 (1.4, 16.2) | 3.6 (1.3, 20.7) | 3.8 (1.2, 25.1) | 4.0 (1.2, 29.7) | 4.1 (1.1, 31.6) | 4.3 (1.1, 33.8) | 4.5 (1.1, 36.5) | 4.7 (1.0, 39.5) |
|  | Current | 0.2 (0.1, 0.4) | 0.2 (0.1, 0.4) | 0.3 (0.1, 0.4) | 0.3 (0.2, 0.6) | 0.4 (0.3, 0.7) | 0.5 (0.3, 0.8) | 0.6 (0.4, 0.9) | 0.6 (0.4, 1.0) |
| **Guyana** | WHO 2013 | 0.2 (0.1, 0.5) | 0.2 (0.1, 0.5) | 0.3 (0.1, 0.5) | 0.4 (0.2, 0.7) | 0.5 (0.3, 0.9) | 0.6 (0.4, 1.1) | 0.7 (0.4, 1.2) | 0.8 (0.5, 1.3) |
|  | 90-90-90 | 0.3 (0.1, 0.6) | 0.3 (0.1, 0.6) | 0.3 (0.1, 0.5) | 0.3 (0.2, 0.6) | 0.4 (0.3, 0.8) | 0.5 (0.3, 0.9) | 0.6 (0.4, 1.0) | 0.7 (0.4, 1.1) |
|  | Current | 4.8 (4.4, 8.0) | 4.5 (4.2, 6.9) | 4.3 (4.1, 6.2) | 4.2 (4.0, 6.0) | 4.3 (4.0, 6.0) | 4.4 (4.0, 6.3) | 4.6 (4.1, 6.6) | 4.7 (4.2, 6.8) |
| **Haiti** | WHO 2013 | 5.5 (5.0, 9.4) | 5.8 (5.4, 9.6) | 5.6 (5.2, 8.8) | 5.5 (5.1, 8.5) | 5.4 (5.0, 8.4) | 5.5 (5.0, 8.5) | 5.6 (5.0, 8.7) | 5.7 (5.0, 8.8) |
|  | 90-90-90 | 12.9 (11.6, 22.9) | 12.1 (10.9, 21.3) | 11.4 (10.2, 20.3) | 10.7 (9.6, 19.1) | 10.1 (9.0, 18.4) | 9.6 (8.5, 17.5) | 9.1 (8.1, 16.5) | 8.8 (7.7, 15.7) |
|  | Current | 1.1 (1.0, 1.2) | 1.0 (0.9, 1.1) | 0.9 (0.9, 1.0) | 0.9 (0.8, 1.1) | 0.9 (0.8, 1.1) | 0.9 (0.7, 1.2) | 0.9 (0.7, 1.2) | 0.9 (0.6, 1.2) |
| **Honduras** | WHO 2013 | 1.1 (1.0, 1.2) | 1.2 (1.1, 1.4) | 1.1 (1.0, 1.2) | 1.1 (0.9, 1.2) | 1.0 (0.8, 1.2) | 1.1 (0.8, 1.3) | 1.1 (0.8, 1.3) | 1.1 (0.7, 1.4) |
|  | 90-90-90 | 2.2 (1.9, 2.6) | 2.0 (1.7, 2.3) | 1.8 (1.6, 2.2) | 1.8 (1.5, 2.1) | 1.7 (1.3, 2.1) | 1.6 (1.2, 2.0) | 1.5 (1.1, 2.0) | 1.4 (1.0, 2.0) |
|  | Current | 0.6 (0.4, 0.6) | 0.5 (0.4, 0.6) | 0.6 (0.4, 0.6) | 0.6 (0.4, 0.6) | 0.6 (0.4, 0.6) | 0.5 (0.4, 0.6) | 0.5 (0.3, 0.6) | 0.5 (0.3, 0.5) |
| **Jamaica** | WHO 2013 | 0.6 (0.4, 0.6) | 0.6 (0.4, 0.6) | 0.6 (0.4, 0.6) | 0.6 (0.4, 0.6) | 0.6 (0.4, 0.6) | 0.6 (0.4, 0.6) | 0.5 (0.3, 0.6) | 0.5 (0.3, 0.6) |
|  | 90-90-90 | 0.8 (0.6, 0.9) | 0.7 (0.5, 0.8) | 0.7 (0.5, 0.8) | 0.7 (0.5, 0.8) | 0.6 (0.4, 0.7) | 0.6 (0.4, 0.7) | 0.6 (0.4, 0.6) | 0.5 (0.3, 0.6) |
|  | Current | 0.2 (0.2, 0.3) | 0.2 (0.2, 0.4) | 0.2 (0.2, 0.4) | 0.3 (0.2, 0.5) | 0.3 (0.3, 0.7) | 0.4 (0.3, 0.8) | 0.5 (0.4, 1.0) | 0.5 (0.4, 1.2) |
| **Nicaragua** | WHO 2013 | 0.2 (0.2, 0.3) | 0.2 (0.2, 0.4) | 0.2 (0.2, 0.5) | 0.3 (0.2, 0.6) | 0.4 (0.3, 0.8) | 0.5 (0.4, 1.0) | 0.6 (0.5, 1.3) | 0.7 (0.5, 1.5) |
|  | 90-90-90 | 0.2 (0.2, 0.4) | 0.2 (0.2, 0.4) | 0.3 (0.2, 0.5) | 0.3 (0.3, 0.6) | 0.4 (0.3, 0.8) | 0.5 (0.4, 1.0) | 0.6 (0.5, 1.3) | 0.8 (0.6, 1.7) |
|  | Current | 0.1 (0.1, 0.2) | 0.1 (0.1, 0.2) | 0.1 (0.1, 0.2) | 0.2 (0.1, 0.2) | 0.2 (0.2, 0.2) | 0.2 (0.2, 0.3) | 0.2 (0.2, 0.3) | 0.2 (0.2, 0.3) |
| **Suriname** | WHO 2013 | 0.1 (0.1, 0.2) | 0.2 (0.1, 0.2) | 0.2 (0.1, 0.2) | 0.2 (0.2, 0.2) | 0.2 (0.2, 0.3) | 0.2 (0.2, 0.3) | 0.3 (0.2, 0.4) | 0.3 (0.3, 0.4) |
|  | 90-90-90 | 0.2 (0.2, 0.2) | 0.2 (0.2, 0.2) | 0.2 (0.2, 0.2) | 0.2 (0.2, 0.2) | 0.2 (0.2, 0.2) | 0.2 (0.2, 0.3) | 0.3 (0.3, 0.4) | 0.4 (0.3, 0.5) |
| **Trinidad and Tobago** | Current | 0.2 (0.2, 0.3) | 0.2 (0.2, 0.3) | 0.2 (0.2, 0.2) | 0.2 (0.2, 0.2) | 0.2 (0.2, 0.2) | 0.2 (0.2, 0.2) | 0.2 (0.1, 0.2) | 0.2 (0.1, 0.2) |
|  | WHO 2013 | 0.2 (0.2, 0.3) | 0.2 (0.2, 0.3) | 0.2 (0.2, 0.2) | 0.2 (0.2, 0.2) | 0.2 (0.2, 0.2) | 0.2 (0.2, 0.2) | 0.2 (0.2, 0.2) | 0.2 (0.1, 0.2) |
|  | 90-90-90 | 0.3 (0.3, 0.3) | 0.3 (0.2, 0.3) | 0.3 (0.2, 0.3) | 0.2 (0.2, 0.3) | 0.2 (0.2, 0.3) | 0.2 (0.2, 0.3) | 0.2 (0.2, 0.2) | 0.2 (0.2, 0.2) |
|  | Current | 0.9 (0.5, 2.0) | 1.2 (0.6, 2.5) | 1.4 (0.7, 2.7) | 1.5 (0.8, 2.9) | 1.6 (0.9, 3.1) | 1.8 (0.9, 3.3) | 1.8 (1.0, 3.3) | 1.8 (0.9, 3.2) |
| **Algeria** | WHO 2013 | 0.8 (0.4, 1.7) | 1.0 (0.6, 1.8) | 1.1 (0.7, 2.0) | 1.2 (0.7, 2.1) | 1.3 (0.8, 2.3) | 1.5 (0.9, 2.5) | 1.5 (0.9, 2.6) | 1.4 (0.9, 2.5) |
|  | 90-90-90 | 1.1 (0.5, 2.8) | 1.2 (0.6, 2.7) | 1.3 (0.7, 2.6) | 1.4 (0.7, 2.8) | 1.5 (0.8, 3.0) | 1.7 (0.9, 3.2) | 1.8 (1.0, 3.4) | 1.8 (1.0, 3.3) |
|  | Current | 0.3 (0.3, 0.4) | 0.3 (0.2, 0.4) | 0.2 (0.2, 0.3) | 0.2 (0.1, 0.3) | 0.1 (0.1, 0.2) | 0.1 (0.1, 0.2) | 0.1 (0.1, 0.1) | 0.1 (0.0, 0.1) |
| **Djibouti** | WHO 2013 | 0.4 (0.3, 0.5) | 0.4 (0.3, 0.5) | 0.4 (0.3, 0.5) | 0.3 (0.2, 0.4) | 0.2 (0.2, 0.3) | 0.2 (0.1, 0.3) | 0.2 (0.1, 0.2) | 0.2 (0.1, 0.2) |
|  | 90-90-90 | 0.9 (0.8, 1.2) | 0.9 (0.7, 1.1) | 0.8 (0.7, 1.0) | 0.7 (0.6, 1.0) | 0.7 (0.5, 0.9) | 0.6 (0.5, 0.8) | 0.5 (0.4, 0.7) | 0.4 (0.3, 0.6) |
|  | Current | 0.2 (0.1, 0.3) | 0.2 (0.1, 0.3) | 0.2 (0.1, 0.3) | 0.2 (0.1, 0.4) | 0.2 (0.1, 0.4) | 0.2 (0.2, 0.4) | 0.3 (0.2, 0.5) | 0.3 (0.2, 0.5) |
| **Egypt** | WHO 2013 | 0.2 (0.1, 0.3) | 0.2 (0.1, 0.3) | 0.2 (0.1, 0.3) | 0.2 (0.1, 0.4) | 0.2 (0.2, 0.4) | 0.3 (0.2, 0.5) | 0.3 (0.2, 0.5) | 0.3 (0.2, 0.5) |
|  | 90-90-90 | 0.2 (0.2, 0.3) | 0.2 (0.2, 0.4) | 0.2 (0.2, 0.4) | 0.3 (0.2, 0.4) | 0.3 (0.2, 0.5) | 0.3 (0.2, 0.5) | 0.3 (0.2, 0.6) | 0.4 (0.2, 0.7) |
|  | Current | 1.2 (1.1, 1.2) | 1.3 (1.2, 1.3) | 1.3 (1.3, 1.4) | 1.4 (1.3, 1.5) | 1.5 (1.4, 1.6) | 1.7 (1.6, 1.8) | 1.8 (1.7, 2.0) | 1.9 (1.8, 2.1) |
| **Iran** | WHO 2013 | 1.3 (1.3, 1.4) | 1.6 (1.5, 1.7) | 1.7 (1.6, 1.7) | 1.7 (1.7, 1.8) | 1.8 (1.8, 1.9) | 2.0 (1.9, 2.1) | 2.1 (2.0, 2.3) | 2.2 (2.1, 2.4) |
|  | 90-90-90 | 1.8 (1.8, 1.9) | 1.9 (1.9, 2.0) | 2.0 (2.0, 2.1) | 2.1 (2.1, 2.2) | 2.3 (2.2, 2.3) | 2.4 (2.3, 2.5) | 2.5 (2.4, 2.6) | 2.7 (2.6, 2.8) |
|  | Current | 0.5 (0.4, 0.7) | 0.6 (0.5, 0.8) | 0.7 (0.5, 0.9) | 0.8 (0.5, 1.1) | 0.8 (0.5, 1.3) | 0.9 (0.5, 1.4) | 0.9 (0.5, 1.5) | 0.9 (0.5, 1.6) |
| **Morocco** | WHO 2013 | 0.6 (0.4, 0.7) | 0.6 (0.5, 0.8) | 0.7 (0.5, 1.0) | 0.8 (0.5, 1.1) | 0.9 (0.5, 1.3) | 0.9 (0.5, 1.4) | 1.0 (0.5, 1.6) | 1.0 (0.5, 1.6) |
|  | 90-90-90 | 0.8 (0.6, 1.0) | 0.8 (0.6, 1.1) | 0.9 (0.6, 1.2) | 1.0 (0.7, 1.4) | 1.0 (0.7, 1.5) | 1.1 (0.7, 1.7) | 1.1 (0.6, 1.8) | 1.2 (0.6, 1.9) |
|  | Current | 2.1 (1.3, 3.2) | 2.1 (1.3, 3.4) | 2.1 (1.2, 3.6) | 2.2 (1.2, 3.8) | 2.2 (1.1, 4.0) | 2.3 (1.0, 4.4) | 2.3 (0.9, 4.7) | 2.3 (0.9, 5.0) |
| **Somalia** | WHO 2013 | 2.6 (1.7, 4.0) | 3.1 (1.9, 4.9) | 3.2 (1.9, 5.2) | 3.4 (1.9, 5.6) | 3.5 (1.9, 6.0) | 3.6 (1.8, 6.7) | 3.8 (1.7, 7.3) | 3.9 (1.7, 7.7) |
|  | 90-90-90 | 4.9 (3.4, 7.2) | 4.9 (3.4, 7.3) | 5.0 (3.3, 7.6) | 5.0 (3.2, 7.9) | 5.0 (3.1, 8.1) | 5.0 (2.9, 8.2) | 4.9 (2.7, 8.6) | 4.8 (2.4, 9.0) |
|  | Current | 2.7 (1.6, 5.0) | 2.8 (1.4, 5.2) | 2.7 (1.2, 5.2) | 2.7 (1.0, 5.2) | 2.7 (0.9, 5.4) | 2.7 (0.8, 5.8) | 2.7 (0.8, 6.2) | 2.7 (0.7, 6.3) |
| **Sudan** | WHO 2013 | 2.9 (1.8, 5.3) | 3.2 (1.8, 5.9) | 3.1 (1.5, 5.8) | 3.1 (1.3, 6.0) | 3.1 (1.2, 6.2) | 3.1 (1.1, 6.6) | 3.1 (1.0, 7.1) | 3.2 (0.9, 7.3) |
|  | 90-90-90 | 4.2 (3.3, 6.8) | 4.3 (3.2, 7.2) | 4.5 (3.0, 7.8) | 4.6 (2.8, 8.2) | 4.7 (2.5, 8.7) | 4.8 (2.3, 9.1) | 4.9 (2.1, 9.5) | 5.0 (1.9, 10.2) |
|  | Current | 0.0 (0.0, 0.1) | 0.1 (0.0, 0.1) | 0.1 (0.0, 0.1) | 0.1 (0.0, 0.1) | 0.1 (0.0, 0.1) | 0.1 (0.0, 0.1) | 0.1 (0.0, 0.1) | 0.1 (0.0, 0.1) |
| **Tunisia** | WHO 2013 | 0.1 (0.0, 0.1) | 0.1 (0.0, 0.1) | 0.1 (0.0, 0.1) | 0.1 (0.0, 0.1) | 0.1 (0.0, 0.1) | 0.1 (0.0, 0.1) | 0.1 (0.0, 0.1) | 0.1 (0.0, 0.1) |
|  | 90-90-90 | 0.1 (0.0, 0.1) | 0.1 (0.0, 0.1) | 0.1 (0.0, 0.1) | 0.1 (0.0, 0.1) | 0.1 (0.0, 0.1) | 0.1 (0.1, 0.1) | 0.1 (0.1, 0.1) | 0.1 (0.1, 0.2) |
|  | Current | 0.2 (0.1, 2.1) | 0.3 (0.2, 2.3) | 0.4 (0.2, 2.4) | 0.5 (0.3, 2.6) | 0.6 (0.4, 2.9) | 0.8 (0.5, 3.2) | 0.9 (0.6, 3.5) | 1.0 (0.6, 3.7) |
| **Yemen** | WHO 2013 | 0.3 (0.1, 2.1) | 0.3 (0.2, 2.4) | 0.4 (0.2, 2.5) | 0.5 (0.3, 2.8) | 0.6 (0.4, 3.0) | 0.8 (0.5, 3.3) | 1.0 (0.6, 3.6) | 1.1 (0.6, 3.9) |
|  | 90-90-90 | 0.4 (0.2, 4.2) | 0.4 (0.2, 4.1) | 0.4 (0.2, 4.0) | 0.6 (0.3, 4.5) | 0.7 (0.4, 4.5) | 0.8 (0.5, 4.7) | 1.0 (0.6, 5.0) | 1.2 (0.7, 5.4) |

Note: Values appearing as zero in this table were less than 100 persons and due to rounding up for purposes of this table, they are not visible. Full numbers were used in the analysis.

**Table E. Baseline ART totals and ART coverage, December 2013**

| **Region** | **Country** | **Adult ART Total** | **Adult Baseline Coverage** | **Children ART Total** | **Children Baseline Coverage** |
| --- | --- | --- | --- | --- | --- |
| MENA | Algeria | 4,205 | 26% | 437 | 40% |
| AES | Angola | 60,738 | 51% | 4,167 | 25% |
| EECA | Armenia | 564 | 43% | 15 | 100% |
| EECA | Azerbaijan | 1,216 | 21% | 36 | 28% |
| LAC | Bahamas | 1,941 | 50% | 49 | 47% |
| AP | Bangladesh | 1,023 | 27% | 60 | 24% |
| LAC | Barbados | 1,067 | 100% | 10 | 96% |
| EECA | Belarus | 5,008 | 39% | 173 | 100% |
| LAC | Belize | 1,335 | 50% | 98 | 63% |
| AWC | Benin | 23,436 | 65% | 1,374 | 24% |
| AP | Bhutan | 120 | 62% | 7 | 18% |
| LAC | Bolivia | 2,921 | 41% | 104 | 19% |
| AES | Botswana | 213,953 | 87% | 9,553 | 96% |
| EECA | Bulgaria | 682 | 72% | 14 | 78% |
| AWC | Burkina Faso | 40,276 | 48% | 1,869 | 15% |
| AES | Burundi | 30,612 | 62% | 2,189 | 18% |
| AP | Cambodia | 46,607 | 78% | 4,052 | 91% |
| AWC | Cameroon | 125,963 | 28% | 5,631 | 10% |
| AWC | Central African Republic | 15,646 | 21% | 922 | 10% |
| AWC | Chad | 40,585 | 31% | 1,573 | 7% |
| AP | China | 202,869 | 52% | 3,337 | 38% |
| AES | Comoros | 27 | 1% | 3 | 1% |
| AWC | Congo | 18,393 | 35% | 1,170 | 16% |
| AWC | Cote d'Ivoire | 107,453 | 56% | 5,467 | 14% |
| LAC | Cuba | 9,629 | 77% | 25 | 48% |
| AWC | Democratic Republic of the Congo | 74,923 | 45% | 5,055 | 11% |
| MENA | Djibouti | 1,683 | 40% | 46 | 8% |
| LAC | Dominican Republic | 20,712 | 71% | 700 | 44% |
| MENA | Egypt | 1,118 | 33% | 53 | 28% |
| AWC | Equatorial Guinea | 7,431 | 69% | 194 | 14% |
| AES | Eritrea | 8,362 | 76% | 692 | 33% |
| AES | Ethiopia | 298,512 | 69% | 18,931 | 13% |
| AWC | Gabon | 22,106 | 66% | 732 | 32% |
| AWC | Gambia | 3,708 | 37% | 289 | 24% |
| EECA | Georgia | 2,047 | 80% | 45 | 85% |
| AWC | Ghana | 71,855 | 67% | 3,907 | 21% |
| LAC | Guatemala | 16,386 | 42% | 951 | 37% |
| AWC | Guinea | 26,459 | 53% | 1,138 | 15% |
| AWC | Guinea-Bissau | 6,485 | 25% | 428 | 10% |
| LAC | Guyana | 3,870 | 79% | 184 | 97% |
| LAC | Haiti | 52,120 | 67% | 2,625 | 37% |
| LAC | Honduras | 8,844 | 49% | 725 | 44% |
| AP | India | 705,537 | 61% | 35,504 | 49% |
| AP | Indonesia | 52,449 | 29% | 1,695 | 10% |
| MENA | Iran | 4,300 | 13% | 172 | 15% |
| LAC | Jamaica | 7,772 | 48% | 515 | 100% |
| EECA | Kazakhstan | 3,432 | 56% | 192 | 78% |
| AES | Kenya | 596,228 | 78% | 60,141 | 42% |
| EECA | Kyrgyzstan | 779 | 31% | 89 | 98% |
| AP | Laos | 2,448 | 86% | 183 | 56% |
| AES | Lesotho | 96,392 | 37% | 5,243 | 21% |
| AWC | Liberia | 6,051 | 31% | 378 | 13% |
| AES | Madagascar | 502 | 3% | 17 | 0% |
| AES | Malawi | 430,645 | 76% | 42,220 | 37% |
| AP | Malaysia | 16,862 | 42% | 507 | 93% |
| AWC | Mali | 26,724 | 41% | 2,001 | 20% |
| AWC | Mauritania | 2,473 | 50% | 51 | 8% |
| AES | Mauritius | 1,818 | 37% | 12 | 38% |
| EECA | Moldova | 2,411 | 39% | 82 | 70% |
| AP | Mongolia | 85 | 50% | 0 | 0% |
| MENA | Morocco | 6,131 | 46% | 333 | 65% |
| AES | Mozambique | 456,055 | 63% | 41,400 | 36% |
| AP | Myanmar | 62,718 | 60% | 4,925 | 95% |
| AES | Namibia | 116,532 | 81% | 10,247 | 54% |
| AP | Nepal | 8,228 | 40% | 632 | 80% |
| LAC | Nicaragua | 2,346 | 59% | 112 | 59% |
| AWC | Niger | 11,517 | 58% | 554 | 13% |
| AWC | Nigeria | 592,084 | 46% | 47,313 | 20% |
| AP | Pakistan | 4,321 | 11% | 70 | 5% |
| AP | Papua New Guinea | 13,905 | 74% | 874 | 34% |
| AP | Philippines | 4,840 | 69% | 17 | 11% |
| EECA | Romania | 8,609 | 73% | 215 | 77% |
| EECA | Russia | 140,145 | 37% | 4,000 | 43% |
| AES | Rwanda | 121,452 | 85% | 8,116 | 65% |
| AWC | Senegal | 12,893 | 56% | 823 | 26% |
| EECA | Serbia | 1,178 | 65% | 11 | 31% |
| AWC | Sierra Leone | 8,680 | 38% | 385 | 14% |
| MENA | Somalia | 1,177 | 12% | 315 | 10% |
| AES | South Africa | 2,466,570 | 73% | 156,706 | 75% |
| AES | South Sudan | 6,613 | 11% | 286 | 2% |
| AP | Sri Lanka | 492 | 53% | 27 | 49% |
| MENA | Sudan | 3,058 | 17% | 250 | 7% |
| LAC | Suriname | 1,415 | 71% | 80 | 59% |
| AES | Swaziland | 92,240 | 81% | 7,898 | 61% |
| EECA | Tajikistan | 1,145 | 13% | 254 | 42% |
| AES | Tanzania | 473,707 | 67% | 38,848 | 29% |
| AP | Thailand | 240,907 | 81% | 5,142 | 79% |
| AWC | Togo | 31,231 | 45% | 3,258 | 25% |
| LAC | Trinidad and Tobago | 5,988 | 69% | 146 | 78% |
| MENA | Tunisia | 525 | 23% | 21 | 44% |
| AES | Uganda | 551,650 | 52% | 43,525 | 22% |
| EECA | Ukraine | 52,840 | 52% | 2,945 | 99% |
| EECA | Uzbekistan | 5,146 | 28% | 3,145 | 99% |
| AP | Vietnam | 78,483 | 57% | 4,204 | 99% |
| MENA | Yemen | 842 | 29% | 59 | 23% |
| AES | Zambia | 530,702 | 86% | 49,389 | 55% |
| AES | Zimbabwe | 618,980 | 77% | 46,319 | 40% |

**Table F. Range for first to second line annual migration estimates**

| **Region** | **High** | **Med** | **Low** | **Sources** |
| --- | --- | --- | --- | --- |
| AES | 3% | 2% | 1% | [[16-19](#_ENREF_16)] |
| AWC | 2% | 1.5% | 1% |  |
| AP | 2% | 1.3% | 0.8% |  |
| LAC | 3.5% | 3% | 2.5% |  |
| EECA | 1.5% | 1% | 0.5% |  |
| MENA | 1.5% | 1% | 0.5% |  |

**REFERENCES**

1. UNAIDS (2015) AIDSInfo Online Database <http://www.aidsinfoonline.org/> Accessed: May 11, 2015. Geneva.

2. The World Bank (2015) Country and Lending Groups: <http://data.worldbank.org/about/country-and-lending-groups#OECD_members>. Accessed May 11, 2015. Washington, DC: The World Bank Group.

3. Stover J, McKinnon R, Winfrey B (2010) Spectrum: a model platform for linking maternal and child survival interventions with AIDS, family planning and demographic projections. International journal of epidemiology 39: i7-i10.

4. Avenir Health (2015) Download link for Spectrum: <http://www.avenirhealth.org/software-spectrum.php>. Glastonbury: Connecticut.

5. Stover J, Brown T, Marston M (2012) Updates to the Spectrum/Estimation and Projection Package (EPP) model to estimate HIV trends for adults and children. Sexually Transmitted Infections 88: i11-i16.

6. UNAIDS (2015) National HIV estimates files. Available via request: <http://apps.unaids.org/spectrum/>. Accessed May 11, 2015. Geneva.

7. Avenir Health (2015) Download link for Country Data Package: <http://www.avenirhealth.org/Download/Spectrum/CountryDataInstall.EXE>. Glastonbury: Connecticut.

8. NACO, NIMS (2012) Technical Report: India HIV Estimates-2012. New Delhi, India: National Institute of Medical Sciences (NIMS), National AIDS Control Organization (NACO), Ministry of Health and Family Welfare, Government of India.

9. UNAIDS (2014) The Gap Report.

10. UNAIDS (2014) 90-90-90 ACT Initiative Regional Workshop.

11. WHO (2014) Antiretroviral Medicines in Low- and Middle-Income Countries: Forecasts of Global and Regional Demand for 2013-2016. Geneva, Switzerland: World Health Organization (WHO).

12. A. Dutta NP, O. Semeryk, O. Balakireva, T. Aleksandrina, O. Ieshchenko, and M. Zelenska (2014) Lives on the Line: Funding Needs and Impacts of Ukraine's National HIV/AIDS Program, 2014-2018.

13. MOH (2013) National Forecasting and Quantification Report for HIV and AIDS Commodities for FY 2013/14 and 2014/15. National AIDS and STI Control Program, Ministry of Health, Government of Kenya. Nairobi, Kenya.

14. RW S (2006) Rationale and Uses of a Public HIV Drug-Resistance Database. Journal of Infectious Diseases 194: S51-58.

15. Avenir Health (2015) Spectrum Version 5.22. Glastonbury, Connecticut.

16. Stanford University (2015) HIV Drug Resistance Database. <http://hivdb.stanford.edu/> Accessed May 11, 2015. Stanford, California: Stanford University.

17. WHO (2012) The HIV drug resistance report. Geneva: World Health Organization.

18. WHO (2014) Antiretroviral Medicines in Low- and Middle-Income Countries: Forecasts of Global and Regional Demand for 2013-2016.

19. UNAIDS (2015) Country Progress Reporting. <http://www.unaids.org/en/dataanalysis/knowyourresponse/countryprogressreports/2012countries>. Accessed May 11, 2015. Geneva: UNAIDS.
