# Supplementary material for: The HIV Treatment Gap: Estimates of the Financial Resources Needed versus Available for Scale-Up of Antiretroviral Therapy in 97 Countries from 2015 to 2020
Source: PLoS Med. 2015 Nov 24;12(11):e1001907. doi: 10.1371/journal.pmed.1001907 (PMC4658189; doi:10.1371/journal.pmed.1001907)
Supplement: S2 Text — (DOCX) [file pmed.1001907.s002.docx]

**S2 Text. Uncertainty analysis**

Primary sources of uncertainty in future estimates of numbers of individuals on ART and the costs of these services derive from uncertain estimates of PLHIV and the need for treatment, future coverage achieved by countries, switching rates from first- to second-line ART, future unit costs, as well as other parameters. The range in the uncertainty in the projections of future eligible populations for ART and the number of PLHIV depends fundamentally on the methods and quality of data used in Spectrum to estimate national prevalence curves from historic point HIV prevalence estimates in sentinel surveillance and population-based surveys. We conducted probabilistic sensitivity analysis to examine the impact of uncertain epidemiological, coverage, and unit cost inputs in the model. Expected mean values were derived from sampling events in 5,000 second-order Monte Carlo simulation trials performed using RiskAmp software (©Structured Data, LLC) integrated with Microsoft Excel [[1](#_ENREF_1)]. These means were used for analytical results reported from the model. Each simulation was run simultaneously over all uncertain parameters. Simulation utilized distribution types with bounds and modes derived from country-specific Spectrum and/or other secondary data as described in main manuscript Table 2 (also see more information in this SI). Confidence intervals were estimated for most results to describe the overall uncertainty in the estimates. We used simple random sampling, rather than Latin Hypercube, over 5,000 trials for the Monte Carlo methodology. Figures A and B show some scatter plots of randomly selected variables of interest across our modeled eligibility scenarios as a demonstration (N: 5,000).

Table 2 in the main manuscript relates to the types of distributions used, ranges and sources. For many of the uncertain parameters as well underlying epidemiological estimates, the sampling distribution used to model the variability is the beta PERT distribution. This section will discuss the beta PERT distribution in specific.

The PERT distribution is a special implementation of the beta distribution and is derived from three parameters: mode, minimum and maximum [[2](#_ENREF_2)]. The beta PERT distribution has several attractive attributes which makes it well-suited to model the unknown parameter distributions for public health and costing studies in developing country settings, where precise parameter estimates are not available. This distribution is less sensitive to the two extreme values vs. the mode, compared to the often used triangular distribution, and better fits the situation where either due to expert opinion or the data generation process, a most likely value or a measure of central tendency is available. This compares to situations where just minimum/maximum values are available, that would otherwise suggest applying a uniform distribution. It is uniquely suited to model expert opinion and situations where a mode (most likely) value has high currency in real world use. As discussed in the paper, countries, UN institutions and researchers place a large emphasis on the median value from the Spectrum AIM estimation for most epidemiological outputs, such as PLHIV and need for ART. Therefore, referencing this value as the mode to generate a beta PERT distribution is appropriate. For cost inputs (see Table 2, main paper), again the most likely value is suggested by a review of the current literature and the dominant value as used in other analyses.

Applying the beta PERT distribution for Monte Carlo simulation trials leads to sampling distributions of values of parameters of interest that resemble a normal distribution especially with relatively un-skewed setting of the mode around the minimum and maximum values and with the default “scale value” ( λ) for the distribution (in RiskAmp, the default is 4). Therefore, they may be preferred to model situations as described for Spectrum output, cost inputs, etc., compared to the triangular distribution that is commonly used for second order Monte Carlo simulations in public health literature. The beta PERT distribution produces lower standard deviation compared to the triangular distribution and smaller confidence intervals. This does not necessarily represent over-confidence in the estimate, but rather a conscious choice privileging the most likely value. The general density function for the beta distribution is:


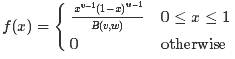


Where *v* and *w* are two shape parameters

For the PERT distribution, the mean is calculated as:


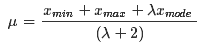
Which can be used to generate the shape parameters as follows:


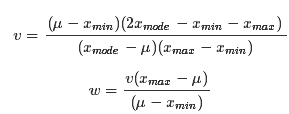


**Figure A. Scatter plot of adults on ART (1^st^ line) vs. total cost of 1^st^ line ARVs in 2020, current eligibility – 5,000 simulations**

| 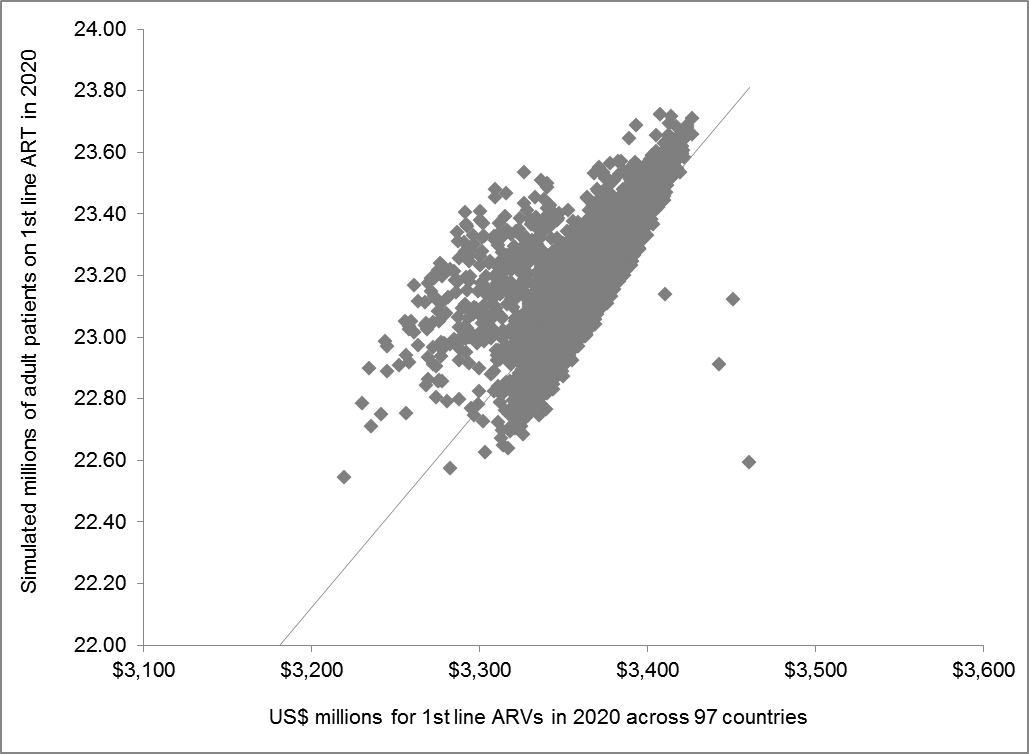 |
| --- |

**Figure B. Scatter plot of total patients on ART vs. total cost of ART in 2020, current eligibility scenario**


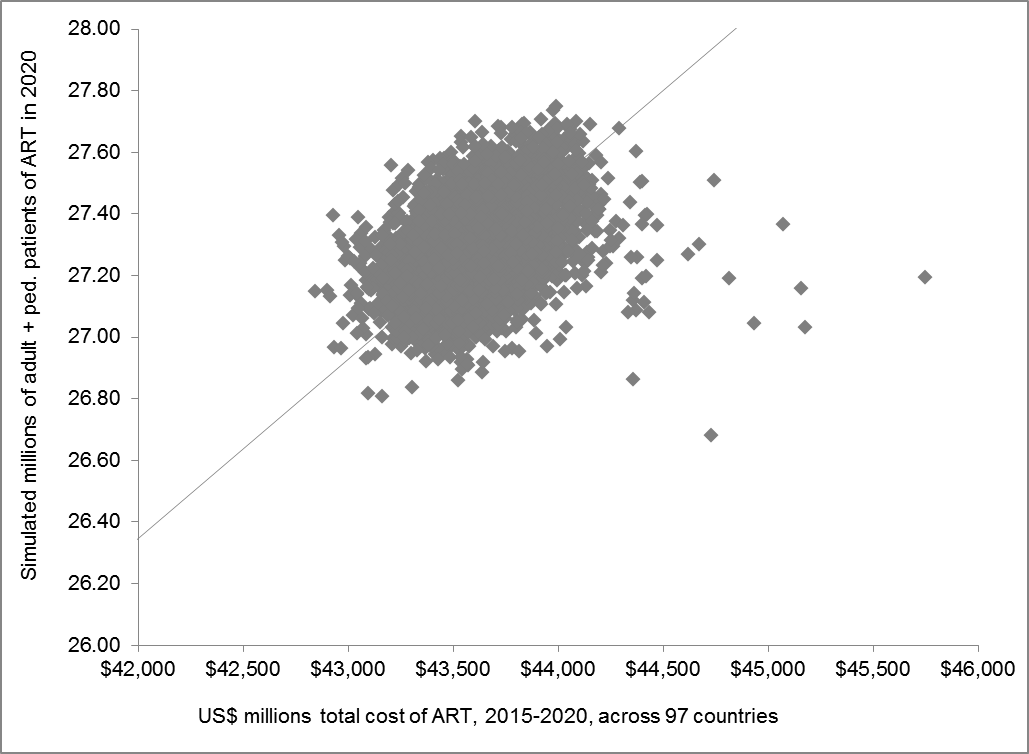


**REFERENCES**

1. Structured Data LLC (2015) RiskAmp v. 4.72 Professional Edition <http://riskamp.com/>. San Francisco, California.

2. McBride WJ, McClelland CW (1967) PERT and the beta distribution. Engineering Management, IEEE Transactions on: 166-169.
