# Supplementary material for: The HIV Treatment Gap: Estimates of the Financial Resources Needed versus Available for Scale-Up of Antiretroviral Therapy in 97 Countries from 2015 to 2020
Source: PLoS Med. 2015 Nov 24;12(11):e1001907. doi: 10.1371/journal.pmed.1001907 (PMC4658189; doi:10.1371/journal.pmed.1001907)
Supplement: S3 Text — (DOCX) [file pmed.1001907.s003.docx]

**S3 Text. Cost estimation**

1. Antiretroviral drugs (ARVs) costs

Average ARV prices, separated by country income level and zone, are primarily from the Global Price Reporting Mechanism (GPRM). The prices include the purchases of generic and brand ARVs. Data from 2010 to 2012 include about 70% of public ARV procurements. The data in GPRM were provided by: the Clinton Foundation HIV/AIDS Initiative; the Global Fund to Fight AIDS, Tuberculosis, and Malaria; the International Dispensary Association; Management Sciences for Health; Mission Pharma; PEPFAR; Partnership for Supply Chain Management System; UNITAID; the United Nations Children’s Fund (UNICEF); USAID/Deliver (formerly John Snow Inc./Deliver); and the World Health Organization’s Contracting and Procurement Service [[1](#_ENREF_1)]. The baseline unit cost ranges and range in annual cost declines used to calculate the annual simulated weighted average unit cost for first- and second- line adult and pediatric treatment are included in Tables B and C. We ran 55 queries in GPRM to estimate the cost of various regimens in LIC, LMIC, and UMIC, and a total of 60 regimen prices were generated from this data. We used sources other than GPRM to set the range in ARV unit costs in HIC and the upper bounds in unit cost in LIC, as explained in the manuscript.

**2. Laboratory drugs and reagents costs**

The costs of one hematology and one clinical chemistry test were combined. The unit cost of viral load tests, CD4 T-cell tests, and hematology and clinical chemistry tests were based on estimates from 11, 8 and 5 countries, respectively. Data were lacking for UMIC and HIC countries. As a result, we set the range in CD4 T-cell count and viral load laboratory costs in these countries based on the estimated range in unit costs from UNITAID and the WHO[[2](#_ENREF_2),[3](#_ENREF_3)].

**3. Site-level personnel and facility overhead costs**

The list of estimates and sources used to set the ranges for the uncertainty analysis of the component of site-level costs of ART related to personnel and overhead are in Table D. We used the highest, lowest, and median estimates, by income category, across the various values from the literature to set the ranges used in the uncertainty analysis for personnel and overhead costs across cost scenarios. An overall summary of included cost headings is provided in Table A below.

**Table A. Summary of costs assessed for the total unit cost per patient year of ART**

| **Cost heading** | **Included from estimates in literature** | **Excluded** |
| --- | --- | --- |
| Site-level direct costs of service delivery | - Antiretroviral drugs (differs by adult vs. pediatric, by income group, and by region) - Laboratory reagents and consumables: CD4, viral load, hematology and clinical chemistry tests (frequency and cost as per scenario) - Personnel: salary costs per patient year of clinical personnel – nurses, doctors, clinical officers: varies by income group & region. | - Drugs for treatment of opportunistic infections, including TB |
| Site-level indirect costs supporting service delivery | - Facility utilities (water, electricity, other) - Facility support staff (guards, cleaners) - Facility-level administrative staff - General consumables / other supplies at the site - Transport/monthly running costs of vehicles (if used for ART) | - In-service training - Vehicle purchase - Depreciation - M&E - Data clerks, data systems |

**4. Additional cost analysis estimates in Discussion section**

We estimate the financial resource requirement for HIV rapid test kits across the three scenarios given the need to scale-up diagnostic services in tandem with scale-up of ART. We calculated the number of new patients on treatment each year by region by subtracting the mean numbers on treatment and adding the estimated number of people on ART who died from one year to the next. We estimated mortality using the same region-specific mortality rates used in estimating the number of patients who switch from first- to second-line treatment each year (see above). We divided the regional number of new people on treatment by region-specific incidence rates reported in the AIDS Info Database to estimate the number of people to be tested each year [[4](#_ENREF_4)]. We multiplied the number to be tested by region-specific HIV test kit costs from the WHO database on procurement of HIV and hepatitis products to forecast the financial resource requirements [[5](#_ENREF_5)]. Cost estimates derived from the WHO procurement data were adjusted to 2014 US dollars, and transactions from 2010 or earlier were excluded from analysis.

We present possible impacts ARV price reductions will have on the overall cost and funding gap for ART in the Discussion section. To estimate the impact of new ARVs, we substituted the baseline unit costs of adult first- and second- line regimens based on the estimated unit costs per patient per year of two new regimens: dolutegravir + TAF + lamivudine or emtricitabine (first-line) and dolutegravir + boosted protease inhibitor + TAF (second-line). We assumed the maximum, minimum, or mode baseline unit cost for first-line treatment would be $60, depending on country income level and current costs. For instance, if the current mode for first line treatment was already below $60, but the maximum exceeded $60, we changed the maximum unit cost to be $60. Unit cost estimates for the new second-line regimen range from $266 to $357 [[6](#_ENREF_6)]. We estimated that LIC and LMI would pay $266 per patient, whereas UMIC and HIC would pay $357 per patient. We changed the maximum, minimum, or mode baseline unit cost for second-line treatment based on country income level and current costs.

We estimated the cost of introducing improved 2-drug maintenance combinations. These new ARVs are estimated to cost about $40 [[6](#_ENREF_6)]. We assumed 76% of those on treatment in 2020 would be virally suppressed based on recent UNAIDS estimates and that all these patients would receive improved 2-drug maintenance combinations [[7](#_ENREF_7)]. We assume a linear scale-up in use of these combinations from a base of 0% in 2015. The remaining 24% of adult and pediatric patients in 2020 receive the other treatment regimens at the same cost used in our main analysis.

Lastly, we estimate the possible impact of treatment optimization on ART resource needs. One study suggests efavirenz would be effective at lower doses, which would result in cost savings of $16 per person per year [[8](#_ENREF_8)]. We subtracted $16 from the range in unit costs of all regimens that include efavirenz to analyze the impact of this price reduction.

**Table B. First- and second- line adult ARV prices by country income level and zone.**

| **Income status and zone** | **Regimen** | **First- vs. second-line** | **Baseline range in unit cost** | | | **Range in annual cost reductions** | | |
| --- | --- | --- | --- | --- | --- | --- | --- | --- |
|  |  |  | Minimum | Mode | Maximum | Minimum | Mode | Maximum |
| LIC | [TDF + 3TC + EFV] | First | $82.7 | $128.6 | $129.6 | 1.0% | 2.0% | 10.0% |
| LIC | [ZDV+3TC+NVP] | First | $58.3 | $98.6 | $105.0 | 0% | 1.0% | 8.0% |
| LIC | [d4T+3TC+NVP] | First | $37.3 | $52.6 | $79.0 | 0% | 0.1% | 5.0% |
| LIC | [ZDV+3TC]+[LPV/r] | Second | $211.0 | $314.0 | $375.0 | 0% | 9.3% | 13.0% |
| LIC | [TDF+FTC]+[LPV/r] | Second | $203.2 | $309.2 | $363.0 | 0% | 9.3% | 15.0% |
| LMIC-Africa | [TDF + 3TC + EFV] | First | $95.2 | $133.2 | $146.0 | 1.0% | 2.0% | 10.0% |
| LMIC-Africa | [ZDV+3TC+NVP] | First | $98.6 | $98.7 | $112.7 | 0% | 1.0% | 9.0% |
| LMIC-Africa | [d4T+3TC+NVP] | First | $37.3 | $45.9 | $79.0 | 0% | 0.1% | 5.7% |
| LMIC-Africa | [ZDV+3TC]+[LPV/r] | Second | $275.4 | $317.9 | $399.2 | 0% | 1.0% | 9.3% |
| LMIC-Africa | [TDF+FTC]+[LPV/r] | Second | $270.0 | $308.7 | $400.3 | 0% | 1.0% | 9.3% |
| LMIC-Other | [TDF + 3TC + EFV] | First | $122.7 | $128.5 | $172.9 | 1.0% | 2.0% | 10.0% |
| LMIC-Other | [ZDV+3TC+NVP] | First | $98.6 | $99.2 | $103.9 | 0% | 1.0% | 9.0% |
| LMIC-Other | [d4T+3TC+NVP] | First | $37.3 | $55.6 | $79.0 | 0% | 0.1% | 5.7% |
| LMIC-Other | [ZDV+3TC]+[LPV/r] | Second | $266.8 | $342.8 | $831.8 | 0% | 1.0% | 9.3% |
| LMIC-Other | [TDF+FTC]+[LPV/r] | Second | $265.1 | $334.7 | $879.1 | 0% | 1.0% | 9.3% |
| UMIC-Africa | [TDF + 3TC + EFV] | First | $133.7 | $136.3 | $138.8 | 0% | 1.0% | 10.0% |
| UMIC-Africa | [ZDV+3TC+NVP] | First | $92.9 | $95.8 | $100.4 | 0% | 0.5% | 7.0% |
| UMIC-Africa | [d4T+3TC+NVP] | First | $53.0 | $53.0 | $299.7 | 0% | 0.1% | 5.3% |
| UMIC-Africa | [ZDV+3TC]+[LPV/r] | Second | $278.4 | $537.3 | $589.6 | 5.7% | 9.3% | 12.3% |
| UMIC-Africa | [TDF+FTC]+[LPV/r] | Second | $227.8 | $297.5 | $596.9 | 5.7% | 9.3% | 14.0% |
| UMIC-Other | [TDF + 3TC + EFV] | First | $136.0 | $145.5 | $823.0 | 0% | 1.0% | 10.0% |
| UMIC-Other | [ZDV+3TC+NVP] | First | $92.9 | $98.8 | $121.6 | 0% | 0.5% | 7.0% |
| UMIC-Other | [d4T+3TC+NVP] | First | $52.4 | $52.4 | $52.4 | 0% | 0.1% | 5.3% |
| UMIC-Other | [ZDV+3TC]+[LPV/r] | Second | $318.4 | $739.1 | $765.9 | 5.7% | 9.3% | 12.3% |
| UMIC-Other | [TDF+FTC]+[LPV/r] | Second | $309.3 | $726.6 | $780.0 | 5.7% | 9.3% | 14.0% |
| HIC | [TDF + 3TC + EFV] | First | $145.5 | $823.0 | $4,111.5 | 0% | 2.0% | 17.2% |
| HIC | [ZDV+3TC+NVP] | First | $163.7 | $1,910.5 | $4,111.5 | 0% | 0.5% | 7.0% |
| HIC | [d4T+3TC+NVP] | First | $299.7 | $1,406.7 | $4,111.5 | 0% | 0.1% | 5.3% |
| HIC | [ZDV+3TC]+[LPV/r] | Second | $419.0 | $3,062.0 | $4,111.5 | 5.7% | 9.3% | 12.3% |
| HIC | [TDF+FTC]+[LPV/r] | Second | $303.1 | $1,079.0 | $1,288.0 | 5.7% | 9.3% | 14.0% |

**Table C. First- and second- line pediatric ARV prices by region and country income level.**

| **Income status and zone** | **Regimen** | **First- vs. second-line** | **Baseline range in unit cost** | | | **Range in annual cost reductions** | | |
| --- | --- | --- | --- | --- | --- | --- | --- | --- |
|  |  |  | Minimum | Mode | Maximum | Minimum | Mode | Maximum |
| LIC | [ABC+3TC]+LPV/r | First / second | $216.8 | $260.5 | $366.0 | 2.0% | 7.3% | 14.0% |
| LIC | [ZDV +3TC+NVP] | First | $73.2 | $88.2 | $96.0 | 0.0% | 2.0% | 18.0% |
| LIC | [ABC + 3TC] + EFV | First / second | $100.4 | $147.5 | $211.1 | 3.0% | 9.3% | 24.5% |
| LIC | [d4T+3TC+NVP] | First | $44.3 | $46.4 | $55.0 | 0.0% | 0.3% | 0.5% |
| LIC | [ZDV + 3TC] + EFV | Second | $54.9 | $91.7 | $93.0 | 3.0% | 8.0% | 31.0% |
| LIC | [ZDV + 3TC]+LPV/r | Second | $171.4 | $204.7 | $336.0 | 2.0% | 6.3% | 14.0% |
| LMIC-Africa | [ABC+3TC]+LPV/r | First / second | $229.1 | $273.1 | $302.8 | 2.0% | 10.3% | 14.0% |
| LMIC-Africa | [ZDV +3TC+NVP] | First | $76.8 | $91.3 | $98.3 | 0.0% | 3.3% | 18.0% |
| LMIC-Africa | [ABC + 3TC] + EFV | First / second | $101.4 | $160.1 | $184.9 | 2.0% | 9.3% | 18.0% |
| LMIC-Africa | [d4T+3TC+NVP] | First | $49.7 | $54.2 | $57.1 | 0.0% | 0.1% | 1.7% |
| LMIC-Africa | [ZDV + 3TC] + EFV | Second | $70.7 | $96.6 | $110.2 | 3.0% | 11.0% | 31.0% |
| LMIC-Africa | [ZDV + 3TC]+LPV/r | Second | $198.4 | $209.6 | $228.1 | 2.0% | 10.7% | 14.0% |
| LMIC-Other | [ABC+3TC]+LPV/r | First / second | $272.6 | $406.4 | $457.9 | 2.0% | 10.3% | 14.0% |
| LMIC-Other | [ZDV +3TC+NVP] | First | $87.9 | $117.3 | $147.0 | 0.0% | 3.3% | 18.0% |
| LMIC-Other | [ABC + 3TC] + EFV | First / second | $142.2 | $184.7 | $198.0 | 2.0% | 9.3% | 18.0% |
| LMIC-Other | [d4T+3TC+NVP] | First | $49.7 | $56.3 | $57.1 | 0.0% | 0.1% | 1.7% |
| LMIC-Other | [ZDV + 3TC] + EFV | Second | $72.8 | $108.2 | $117.7 | 3.0% | 11.0% | 31.0% |
| LMIC-Other | [ZDV + 3TC]+LPV/r | Second | $203.3 | $329.9 | $377.7 | 2.0% | 10.7% | 14.0% |
| UMIC-Africa | [ABC+3TC]+LPV/r | First / second | $175.4 | $194.9 | $199.0 | 2.0% | 7.7% | 14.0% |
| UMIC-Africa | [ZDV +3TC+NVP] | First | $76.8 | $88.3 | $99.8 | 0.0% | 1.0% | 5.0% |
| UMIC-Africa | [ABC + 3TC] + EFV | First / second | $106.4 | $108.0 | $175.1 | 0.0% | 0.5% | 24.5% |
| UMIC-Africa | [d4T+3TC+NVP] | First | $51.0 | $57.6 | $63.1 | 0.0% | 0.0% | 0.0% |
| UMIC-Africa | [ZDV + 3TC] + EFV | Second | $90.6 | $92.2 | $159.3 | 0.0% | 2.0% | 14.3% |
| UMIC-Africa | [ZDV + 3TC]+LPV/r | Second | $159.7 | $179.1 | $183.3 | 0.0% | 2.0% | 11.3% |
| UMIC-Other | [ABC+3TC]+LPV/r | First / second | $284.0 | $330.2 | $347.4 | 2.0% | 7.7% | 14.0% |
| UMIC-Other | [ZDV +3TC+NVP] | First | $92.5 | $93.0 | $102.3 | 0.5% | 1.0% | 5.0% |
| UMIC-Other | [ABC + 3TC] + EFV | First / second | $105.6 | $118.5 | $292.3 | 0.0% | 0.5% | 24.5% |
| UMIC-Other | [d4T+3TC+NVP] | First | $51.0 | $57.6 | $63.1 | 0.0% | 0.0% | 0.0% |
| UMIC-Other | [ZDV + 3TC] + EFV | Second | $66.2 | $82.1 | $255.9 | 0.0% | 0.5% | 14.3% |
| UMIC-Other | [ZDV + 3TC]+LPV/r | Second | $244.6 | $293.7 | $310.9 | 0.0% | 2.0% | 11.3% |
| HIC | [ABC+3TC]+LPV/r | First / second | $469.4 | $525.0 | $4,916.6 | 2.0% | 7.7% | 14.0% |
| HIC | [ZDV +3TC+NVP] | First | $147.0 | $2,018.9 | $4,111.5 | 0.5% | 1.0% | 10.0% |
| HIC | [ABC + 3TC] + EFV | First / second | $623.0 | $2,350.8 | $4,111.5 | 0.0% | 9.0% | 24.5% |
| HIC | [d4T+3TC+NVP] | First | $98.8 | $1,406.7 | $4,111.5 | 0.0% | 0.0% | 0.0% |
| HIC | [ZDV + 3TC] + EFV | Second | $450.0 | $1,545.8 | $4,111.5 | 2.0% | 14.3% | 31.0% |
| HIC | [ZDV + 3TC]+LPV/r | Second | $377.7 | $4,111.5 | $4,111.5 | 2.0% | 11.3% | 31.0% |

Source: [[1](#_ENREF_1),[9-12](#_ENREF_9)]

**Table D. Unit cost estimates (2014 US$) used to set ranges for uncertainty analysis and ultimate parameter applied for site-level costs of personnel and overhead**

| **Income status** | **Personnel** | | **Overhead** | |
| --- | --- | --- | --- | --- |
|  | **Adjusted constant 2014 $** | **Source** | **Adjusted constant 2014 $** | **Source** |
| **Low income** | $33.13 (Haiti) | [[13](#_ENREF_13)] | $48.09 (Haiti) | [[13](#_ENREF_13)] |
|  | $56.50 (Tanzania) | [[14](#_ENREF_14)] | $43.5 (Tanzania) | [[14](#_ENREF_14)] |
|  | $55.73 (Benin) | [[15](#_ENREF_15)] | $22.88 (Benin) | [[15](#_ENREF_15)] |
|  | $40.98 (Ethiopia) | [[15](#_ENREF_15)] | $41.29 (Uganda) | [[16](#_ENREF_16)] |
|  | $26.71 (Uganda) | [[16](#_ENREF_16)] | $18.94 (Uganda) | [[16](#_ENREF_16)] |
|  | $19.91 (Uganda) | [[16](#_ENREF_16)] | $64.95 (Ethiopia) | [[15](#_ENREF_15)] |
|  | $23.64 (Ethiopia) | [[15](#_ENREF_15)] | $50.91 (Haiti) | [[14](#_ENREF_14)] |
|  | $29.83 (Malawi) | [[17](#_ENREF_17)] | $38.05 (Rwanda) | [[17](#_ENREF_17)] |
|  | $68.91 (Rwanda) | [[17](#_ENREF_17)] | $40.11 (Ethiopia) | [[17](#_ENREF_17)] |
|  | $28.80 (Ethiopia) | [[17](#_ENREF_17)] |  |  |
| **Lower-middle income** | $38.11 (Zambia) | [[18](#_ENREF_18)] | $67.01 (Zambia) | [[19](#_ENREF_19)] |
|  | $70.10 (Zambia) | [[19](#_ENREF_19)] | $26.52 (Kenya) | [[20](#_ENREF_20)] |
|  | $40.25 (Kenya) | [[20](#_ENREF_20)] | $35.02 (Kenya) | [[21](#_ENREF_21)] |
|  | $26.67 (Kenya) | [[21](#_ENREF_21)] | $55.85 (Nigeria) | [[22](#_ENREF_22)] |
|  | $29.65 (Swaziland) | [[23](#_ENREF_23)] | $22.44 (Vietnam) | [[24](#_ENREF_24)] |
|  | $34.84 (Nigeria) | [[22](#_ENREF_22)] | $25.13 (Zambia) | [[25](#_ENREF_25)] |
|  | $63.05 (Vietnam) | [[24](#_ENREF_24)] | $38.05 (Zambia) | [[17](#_ENREF_17)] |
|  | $29.32 (Zambia) | [[25](#_ENREF_25)] |  |  |
|  | $75.08 (Zambia) | [[17](#_ENREF_17)] |  |  |
| **Upper-middle and high income** | $472.19 (South Africa) | [[15](#_ENREF_15)] | $206.55 (South Africa) | [[15](#_ENREF_15)] |
|  | $67.34 (South Africa) | [[26](#_ENREF_26)] | $18 (South Africa) | [[15](#_ENREF_15)] |
|  | $325.28 (South Africa) | [[15](#_ENREF_15)] | $66.85 (South Africa) | [[17](#_ENREF_17)] |
|  | $334 (South Africa) | [[17](#_ENREF_17)] |  |  |

Two values from studies for personnel costs excluded as extreme outliers (Low Income). Two values from studies excluded for overhead from lower-middle income countries as extreme outliers. One value excluded from personnel costs for lower-middle income countries as extreme outlier. Ranges for uncertainty analysis constructed by country income group using listed sources. Mode (most likely) = median of values for personnel or overhead costs, as needed. Minimum and maximum as defined. US$ GDP deflators were sourced from the IMF [[27](#_ENREF_27)].

**REFERENCES**

1. WHO (2013) Transaction Prices for Antiretroviral Medicines from 2010 to 2013: Global Price Reporting Mechanism.

2. UNITAID (2014) HIV/AIDS Diagnostics Technology Landscape - 4th edition.

3. World Health Organization (2014) Technical and operational considerations for implementing HIV viral load testing.

4. (2014) AIDSinfo Online Database.

5. World Health Organization Database on procurement of HIV and hepatitis products.

6. Barnhart M, Shelton JD (2015) ARVs: The Next Generation. Going Boldly Together to New Frontiers of HIV Treatment. Global Health: Science and Practice: ghs1400243.

7. UNAIDS (2014) The Gap Report.

8. Heger M (2013) Trials challenging HIV drug doses could usher in huge cost cuts. Nature Medicine 19: 953.

9. Godlevskiy D, Volgina A, Golovin S, Girchenko P, Skvortsov A (2012) To treat or not to treat? ARV treatment procurement and provision in Russia. Saint Petersburg, Russia.

10. Clinton Health Access Initiative (2014) 2014 Antiretroviral (ARV) Ceiling Price List.

11. Clinton Health Access Initiative (2013) The State of the Antiretroviral Drug Market in Low- and Middle-Income Countries: ARV Market Report.

12. Perriëns JH, Habiyambere V, Dongmo-Nguimfack B, Hirnschall G (2014) Prices paid for adult and paediatric antiretroviral treatment by low-and middle-income countries in 2012: high, low or just right? Antiviral therapy 19: 39-47.

13. Riviere C, Faust E, Miller T, Beck EJ, Baruwa E, et al. (2014) Superior Outcomes and Lower Outpatient Costs With Scale-Up of Antiretroviral Therapy at the GHESKIO Clinic in Port-au-Prince, Haiti. JAIDS Journal of Acquired Immune Deficiency Syndromes 66: e72-e79.

14. Paxton A, Kallarakal A, Dutta A, Emmanuel E, Cleghorn F (2015) A Standardized Methodology for Outcome-adjusted Unit Expenditure Analysis of HIV Treatment Using Electronic Medical Records: Haitian and Tanzanian case studies. Forthcoming.

15. Galárraga O, Wirtz V, Figueroa-Lara A, Santa-Ana-Tellez Y, Coulibaly I, et al. (2011) Unit Costs for Delivery of Antiretroviral Treatment and Prevention of Mother-to-Child Transmission of HIV. PharmacoEconomics 29: 579-599.

16. Moreland S, Namisango E, Paxton A, RA P (2013) The Costs of HIV Treatment, Care, and Support Services in Uganda.

17. Tagar E, Sundaram M, Condliffe K, Matatiyo B, Chimbwandira F, et al. (2014) Multi-Country Analysis of Treatment Costs for HIV/AIDS (MATCH): Facility-Level ART Unit Cost Analysis in Ethiopia, Malawi, Rwanda, South Africa and Zambia. PLoS ONE 9: e108304.

18. Marseille E, Giganti MJ, Mwango A, Chisembele-Taylor A, Mulenga L, et al. (2012) Taking ART to scale: determinants of the cost and cost-effectiveness of antiretroviral therapy in 45 clinical sites in Zambia.

19. Scott CA, Iyer H, Bwalya DL, McCoy K, Meyer-Rath G, et al. (2013) Retention in care and outpatient costs for children receiving antiretroviral therapy in Zambia: a retrospective cohort analysis. PloS one 8: e67910.

20. U.S. Centers for Disease Control, Health KMo (2013) The Costs of Comprehensive HIV Treatment in Kenya. Atlanta, GA (USA) and Nairobi, Kenya.

21. Larson BA, Bii M, Henly-Thomas S, McCoy K, Sawe F, et al. (2013) ART treatment costs and retention in care in Kenya: a cohort study in three rural outpatient clinics. Journal of the International AIDS Society 16.

22. Aliyu HB, Chuku NN, Kola-Jebutu A, Abubakar Z, Torpey K, et al. (2012) What is the cost of providing outpatient HIV counseling and testing and antiretroviral therapy services in selected public health facilities in Nigeria? JAIDS Journal of Acquired Immune Deficiency Syndromes 61: 221-225.

23. Obure CD, Sweeney S, Darsamo V, Michaels-Igbokwe C, Guinness L, et al. (2015) The Costs of Delivering Integrated HIV and Sexual Reproductive Health Services in Limited Resource Settings. PloS one 10.

24. Duong AT, Kato M, Bales S, Do NT, Minh Nguyen TT, et al. (2014) Costing Analysis of National HIV Treatment and Care Program in Vietnam. Journal of Acquired Immune Deficiency Syndromes (1999) 65: e1-e7.

25. Scott CA, Iyer HS, McCoy K, Moyo C, Long L, et al. (2014) Retention in care, resource utilization, and costs for adults receiving antiretroviral therapy in Zambia: a retrospective cohort study. BMC Public Health 14: 296.

26. Meyer-Rath G (2015) National ART Cost Model, South Africa. Johannesburg: Health Economics and Epidemiology Research Office, Boston University of the Witwatersrand.

27. IMF (2014) International Financial Statistics. Available at: <http://elibrary-data.imf.org/FindDataReports.aspx?d=33061&e=169393>.
