# Supplementary material for: The HIV Treatment Gap: Estimates of the Financial Resources Needed versus Available for Scale-Up of Antiretroviral Therapy in 97 Countries from 2015 to 2020
Source: PLoS Med. 2015 Nov 24;12(11):e1001907. doi: 10.1371/journal.pmed.1001907 (PMC4658189; doi:10.1371/journal.pmed.1001907)
Supplement: S4 Text — (DOCX) [file pmed.1001907.s004.docx]

**S4 Text. Estimating available financial resources**

1. Global Fund

We estimated financial resources available for ART based on country specific funding trends from Global Fund, PEPFAR, and domestic sources. We assumed all countries eligible for Global Fund funding would maintain constant funding at an estimated annual average. This average was generated based on the recent values of total annual Global Fund contributions to ARV and laboratory commodity procurement per country as per specific information on Global Fund grants (available for a few countries), or based on annual data extracted from the Price and Quality Reporting database for the years 2011, 2012, 2013, and 2014 [[1](#_ENREF_1),[2](#_ENREF_2)]. We used the annual averages of the Global Fund New Funding Model’s 2014-2016 country allocations to HIV, which includes values other than for ART, inclusive of any incentive funding awarded, to reality check if our estimated annual Global Fund contribution to ART commodity costs was appropriate for each country. All values for Global Fund-related data and our estimates are shown in Table A at the end of this supporting information note.

The annual global total generated from our estimated per-country annual average of projected Global Fund contributions to ART commodities suggests that approximately $445 million can be expected from the Global Fund portfolios to cover its portion of the total cost of ART commodities. Projections on Global Fund funding on ARV volumes suggested an overall demand for ARVs that was set to rise from $450 million in 2014, to $525 million for adults, and from $17.8 million in 2014 to $24.8 million in 2015 for pediatric treatments [[3](#_ENREF_3)]. These projected values are global, and include certain countries we did not include our sample, though the variance due to this will be small. More generally, it is possible that we may underestimate future Global Fund contributions to ART commodities. Our estimate is based on publicly available data and is closer to historical values of Global Fund contributions to these cost categories. Also, while the PQR database is fairly complete, many principal recipients do not report in – we estimated India’s procurements in 2013 using Global Fund grants separately (see notes to Table A). In addition, we were not able to account for commodities procured historically under the Voluntary Pooled Procurement Mechanism, which did not report all transactions into PQR [[2](#_ENREF_2)]. Table A lists Global Fund contributions by year for each country’s total HIV commodity transactions for 2011-2014 and adjusted averages for the period, and can be compared to the NFM allocation as a whole and the calculated NFM annual averages.

In the case of Kenya, Mozambique, Tanzania, The Gambia, Ghana, and Ukraine, the study team had access to actual country NFM proposals or NFM grant approval notices. We applied annual Global Fund disbursements aligned with these country-specific requests. The annual totals applied are shown Table B and were either taken directly from the NFM proposals or calculated by applying NFM unit-costs to the annual targets listed in the NFM. Most of these select countries had disbursements listed for 2015, 2016, and 2017. Each subsequent year’s anticipated GFATM contribution was estimated as the average of the preceding two years’ disbursements.

1. Domestic resources

Global Fund eligibility criteria extend funding to all lower income countries and lower-middle income countries. Disease burden is factored in for upper middle income counties who are only eligible for funding if their reported disease burden is measured as ‘High,’ ‘Severe,’ or ‘Extreme.’ High income countries are generally not eligible for Global Fund grants unless special eligibility applies, such as the NGO rule (see Table C) [[4](#_ENREF_4)]. Eligibility criteria also define Global Fund’s Counterpart Financing policy. All Global Fund applicants must meet a minimum level of government contribution as a share of the total global government and Global fund financing for that disease. Minimum Counterpart Financing Thresholds (CFT) are assigned as 5 percent for LICs, 20 percent for lower-LICs, 40 percent for upper-LICs, and 60 percent for UMIC [[5](#_ENREF_5)]. Table C shows Global Fund eligibility classifications for the 93 countries included in this analysis including minimum CFT percentages.

Where applicable, CFTs account for the main source of domestic funding for commodities. In cases where countries were either not eligible for Global Fund funding or were not expecting to receive further HIV specific funds, the estimated country CFT contributions were not included as part of total domestic resources. Rather, country specific commodity domestic contributions (DC) in the PEPFAR 2014 resource, or the most recent available domestic contribution percent was used from the AIDSinfo database which sites each respective year’s GARPR report [[6](#_ENREF_6)]. Domestic contribution was estimated as the share of domestic *public* sources in the total of domestic public plus international contributions to the country’s HIV response in the year of the report. These are reported by countries and may be prone to errors in the country’s own accounting or calculations. Table D indicates whether anticipated domestic resources for ART comes either from the countries’ Global Fund counterpart financing commitment, a country specific domestic contribution, or a combination of the two across various ART costs. Counterpart financing threshold allocations were only applied to commodity cost while domestic contribution could have been applied to either commodity costs or non-commodity costs, depending on the case. Table C shows how the DC percentage is applied for each country.

For some countries other secondary literature provided details on total domestic resources used to procure ARVs and other commodities. These countries are listed in Table E along with the annual domestic contribution dollar total applicable to finance the ART commodity needs.

**3. PEPFAR**

PEPFAR resources have been adjusted in recent Country Operational Plan cycles following a policy shift towards an enhanced focus on epidemic control and maximally reduced mortality [[7](#_ENREF_7)]. Country Operational Plans are organized by country and across the agencies of the US government involved in PEPFAR response, which are in the main: U.S. Centers for Disease Control, USAID, and the Department of Defense. Under the rubric of “right things, right places, at the right time”, there is increased emphasis on a core set of activities that generate the highest outcomes. Resources for site-level support have also been emphasized in 2015 vs. those at above-site level. However, quantitative data to track these changes are not fully in the public domain. In terms of data that are in public domain and available for this study, PEPFAR expenditures by country and by area of spending are available for PEPFAR financial year (FY) 2013 (*http://data.pepfar.net* as of August 2015). PEPFAR proposed budgets for financial year 2014 are available from the Congressional Budget Justification Supplement [[8](#_ENREF_8)]. We used these two sets of data to project PEPFAR annual support, by country for the period 2015-20.

In FY 2014, 32 of our 97 countries modeled for this study were receiving PEPFAR support as per the budget justification document. Of these, 19 had PEPFAR expenditure analysis data publicly available for FY 2013. PEPFAR expenditures often vary for commodities as well as non-commodity costs at the site level from the original planned budgets, because of country-level exigencies and requests from government. Therefore, we attempted to use the FY 2013 expenditure data in conjunction with the FY 2014 planned budgets for all 32 countries to provide the most robust estimates for PEPFAR support to ART-related commodities and site-level costs.

Our approach to estimating PEPFAR funding is as follows:

1. Use the most recent publicly available data as the primary basis (FY 2014 plan budget)
2. Utilize the allocations/proportions from the FY 2013 expenditure analysis for 19 countries to extract detailed information on the areas of support that match costs in our study
3. Reconcile differences in the organization of categories/sub-categories in the expenditure data for 19 countries with the high-level aggregates available from the planned budgets for FY 2014 for all 32 countries.
4. Avoid over-estimation of PEPFAR funding to countries given the specific nature of the costs included in our study. However, due to the organization of PEPFAR support, it is difficult to isolate support to facilities purely for treatment, i.e., excluding pre-ART care, as many personnel supported perform functions for both. Therefore, some element of over-estimation is unavoidable.

*General difference in organization of FY 2014 planned budget vs. FY 2013 expenditure data*

| **PEPFAR FY 2014 Congressional Budget Justification** | **FY 2013 Expenditure Analysis data** |
| --- | --- |
| Countries: 32 | Countries: 19 |
| Total dollar allocation: by country and by:   - *Prevention* - **Care**: budget codes HBHC, PDCS, HVTB, HKID - **Treatment**: budget codes HTXD, HTXS, PDTX - *Governance & Systems* | Total dollars spent: by country and by:   - Program area (19 choices) - Major cost category (5 choices) - Cost category (19 choices) |

HBHC: adult care & support; PDCS: pediatric care & support; HVTB: TB/HIV; HKID: OVC. HTXD: ARV drugs; HTXS: adult treatment; PDTX: pediatric treatment.

*PEPFAR support to ART-related commodities:* From the FY 2013 expenditure data, we chose specifically to look at PEPFAR spending on ARVs and laboratory reagents (i.e., “non-ARV commodities” in pivot tables of the data, excluding all HIV test kit procurement) as reported under the program areas of community-based care treatment and support, facility-based care treatment and support, and laboratory program areas. This definition means that some element of drugs for opportunistic infection care and treatment could not be excluded. By selecting these program areas we anticipated only capturing commodity costs as appropriate to compare against the values used in developing our estimated total commodity resource needs. ARVs were assessed across all 19 program areas (see table above on disaggregation of PEPFAR expenditure data).

Based on FY 2013 data, several countries which receive PEPFAR support for treatment do not receive any PEPFAR funds for ARVs: Angola, Dominican Republic, Ghana, India, Indonesia, Myanmar, Papua New Guinea, South Sudan, Swaziland, and Ukraine. Though Tanzania was slated to receive no support for ARVs in FY 2014, recent policy shift suggests this will restart.

For countries that had no expenditure analysis data (13 of the 32 total PEPFAR countries), we looked at the detailed PEPFAR planned budgets for FY 2013 (<http://data.pepfar.net>) to identify the planned value. These supplemented the expenditure analysis dataset. Finally, we assessed whether the FY 2014 planned budget figures included resources to support treatment at all. Of these, Angola and Ghana were slated to receive no funds to support treatment, regardless of commodity or non-commodity costs. Therefore, we assumed that there would be no support to ARVs in these countries as well in the future.

For countries that we could assume would have continued PEPFAR contributions for commodities (FY 2014 planned funding for treatment is non-zero), and who had a FY 2014 planned treatment funding total exceeding the FY 2013 calculated commodity expenditures (or, for a few FY 2013 planned budget for commodities), we assumed that PEPFAR would continue to match this contribution, which may be optimistic.

In a few cases where countries did not have FY 2013 expenditure data for either ARVs and/or laboratory reagents, we applied the ARV total available from the similarly disaggregated FY 2012 planned funding, as long as they met the criteria based on comparison to FY 2014 plans. Countries that did not have any, or had minimal ART commodity costs for 2012 or 2013 were not expected to have continuing PEPFAR contributions in this cost area. In certain countries, PEPFAR plans indicate procurement of ARVs would cease in a certain year (e.g., Vietnam after 2016). This information was incorporated in the analysis when projecting resources.

*PEPFAR support to other site-level costs (non-commodity costs)*

As is apparent from the table above, the organization of the FY 2014 planned budget data and the FY 2013 expenditure analysis data do not match; causing a difficulty for the analyst attempting to use the planned budget data for forecasting in specific expenditure areas only. We resolved this by disaggregating the expenditure data and creating appropriate ratios which could be applied to the “care” and “treatment” totals of the FY 2014 planned budget, keeping in mind the types of budget codes that were included in the latter. The step-down schematic shows this.

*Defining facility-based care & treatment support as a proportion of “care + treatment”*: We approach the difference in organization by creating a two-step analysis. First, we identified the proportion of the FY 2014 “care” and “treatment” allocation that could justifiably be allocated to facility-based care and treatment support, given the trends in FY 2013 expenditure data – this is the ***FCTS%***. The next figure shows how this was determined in terms of a denominator and numerator for the ratio, using FY 2013 expenditure data.

Denominator of ***FCTS%***: The denominator for the ratio is the total value by each country of the 19 expenditure analysis countries, of FY 2013 expenditure in the program areas: community-based care, treatment & support; facility-based care, treatment & support, + OVC – matching the budget codes under FY 2014 headings of “care” and “treatment”. Cost category elements for “ARVs” and “non-ARVs drugs and reagents” were excluded from the total, as these were assessed separately.

Numerator for ***FCTS%***: As shown in the figure below. We excluded cost category elements for “ARVs” and “non-ARVs drugs and reagents” and extracted the totals for facility-based care, treatment & support alone.

*Defining specific areas of facility-based costs that would be supported:* We obtained the values for specific areas within facility-based care, treatment and support that relate to the areas of cost which were estimated in our study. The figure below follows the same disaggregation, and shows the elements within “cost category” that were included for a “***high%***” and a “***low%***” calculation of a ratio that could be applied to the estimated value of FCTS from above. The percentages were calculated by dividing the total from a pivot table following the selection below by the FCTS total for the country from the selection above.

*Summary:* FY 2013 expenditure data were used to generate two possible scenarios regarding future PEPFAR contributions to non-commodity resource needs at the site level. For the conservative scenario (***low%***) we chose to look only at certain areas of facility-level expenditure under the Program Area “Facility-based Care, Treatment, and Support”. For the second and more expansive scenario of PEPFAR contributions to non-commodity site-level costs (***high%***), we included expenditure for certain other site-level costs which may be slightly over-estimating the resources applicable to our estimated costs, though there is some uncertainty given lack of specificity in the PEPFAR data. Across both scenarios, we excluded support to “strategic information” within the major cost categories. This was done in order to attempt to capture only the types of facility level non-commodity costs we included in our analysis driven by the costing literature.

Finally, we applied the ***FCTS%*** to the sum of “care” and “treatment” from the FY 2014 planned budget, first subtracting the amount spent on commodities from the total prior to multiplication. For the 13 countries which lacked the FCTS% from the expenditure analysis, we made assumptions based on region and income group to be able to find an appropriate ratio.

As a second step, we multiplied the FCTS allocation by the “***high%***” and a “***low%***” for each country, again making assumptions based on region for the countries missing expenditure analysis data.

**Table A. PQR-based Historical ART Commodity Transactions with Global Fund grants, and New Funding Model (NFM) allocations.**

| **Region** | **Country** | **PQR-reported GFATM Transaction Totals** | | | | **GFATM Avg. Annual Allocation for ART  *(2014 US$)*** | Estimated Total for allocation for ART, 2014-2016  *(2014 US$)* | *Vs. NFM Total HIV allocation* | ***Vs. NFM Annual average*** | *Vs. NFM Incentive Funding (if known)* |
| --- | --- | --- | --- | --- | --- | --- | --- | --- | --- | --- |
|  |  | **2011** | **2012** | **2013** | **2014** |  |  |  |  |  |
| AES | Angola | $3,485,153 | $2,012,742 | $5,843,312 | $- | **$3,780,402** | $11,341,206 | *$23,272,987* | ***$7,757,662*** |  |
| AES | Botswana | $- | $- | $- | $- | **None** | $- | *$18,087,964* |  |  |
| AES | Comoros | $4,422 | $- | $6,132 | $2,780 | **$4,445** | $13,334 | *$3,347,444* | ***$1,115,815*** |  |
| AES | Eritrea | $111,202 | $973,387 | $2,717,269 | $1,037,530 | **$1,209,847** | $3,629,542 | *$39,166,328* | ***$13,055,443*** |  |
| AES | Ethiopia | $42,299,963 | $21,500,215 | $40,232,883 | $10,733,443 | **$28,691,626** | $86,074,878 | *$377,424,533* | ***$125,808,178*** |  |
| AES | Kenya | $4,590,458 | $28,019,846 | $27,535,632 | $17,692,684 | **$19,459,655** | $58,378,965 | *$337,301,097* | ***$112,433,699*** |  |
| AES | Lesotho | $714,382 | $4,281,656 | $8,145,470 | $7,667,967 | **$5,202,369** | $15,607,106 | *$86,231,015* | ***$28,743,672*** |  |
| AES | Madagascar | $- | $128,490 | $113,398 | $167,367 | **$136,418** | $409,255 | *$17,239,872* | ***$5,746,624*** |  |
| AES | Malawi | $26,435,726 | $45,114,616 | $62,407,017 | $47,536,235 | **$45,373,398** | $136,120,195 | *$471,311,098* | ***$157,103,699*** |  |
| AES | Mauritius | $27,033 | $29,989 | $208,337 | $222,071 | **$121,858** | $365,573 | *$5,128,597* | ***$1,709,532*** |  |
| AES | Mozambique ***** | $- | $13,332,265 | $40,079,918 | $9,027,986 | **$20,813,390** | $62,440,169 | *$52,002,749* | ***$17,334,249.67*** | *$43,560,000* |
| AES | Namibia | $4,062,777 | $4,888,852 | $4,619,178 | $8,557,289 | **$5,532,024** | $16,596,072 | *$87,684,999* | ***$29,228,333*** |  |
| AES | Rwanda | $5,291,460 | $4,793,517 | $12,632,902 | $- | **$7,572,626** | $22,717,879 | *$294,584,494* | ***$98,194,831*** |  |
| AES | South Africa | $5,342,808 | $15,041,135 | $26,697,138 | $21,796,188 | **$17,219,317** | $51,657,952 | *$386,675,743* | ***$128,891,914*** |  |
| AES | South Sudan | $355,753 | $838,065 | $250,790 | $1,826,810 | **$817,854** | $2,453,563 | *$46,142,248* | ***$15,380,749*** |  |
| AES | Swaziland | $8,530 | $- | $- | $- | **None** | $- | *$47,869,254* | ***N/A*** |  |
| AES | Tanzania | $732,884 | $51,326,995 | $74,189,968 | $9,825,876 | **$34,018,931** | $102,056,793 | *$172,448,214* | ***$57,482,738*** | *$78,608,549* |
| AES | Uganda ***** | $5,899,524 | $33,423,834 | $34,092,047 | $6,621,999 | **$20,009,351** | $60,028,053 | *$138,211,600* | ***$46,070,533.33*** | *$19,842,776* |
| AES | Zambia | $35,024,216 | $35,066,990 | $26,794,824 | $1,807,020 | **$24,673,263** | $74,019,788 | *$228,874,259* | ***$76,291,420*** |  |
| AES | Zimbabwe | $1,341,315 | $93,511,493 | $8,302,305 | $49,297,987 | **$38,113,275** | $114,339,825 | *$398,186,892* | ***$132,728,964*** |  |
| AWC | Benin | $2,311,054 | $697,136 | $4,219,438 | $310,685 | **$1,884,578** | $5,653,735 | *$62,585,069* | ***$20,861,690*** |  |
| AWC | Burkina Faso | $6,866,729 | $7,435,034 | $8,554,361 | $7,209,483 | **$7,516,401** | $22,549,204 | *$77,127,516* | ***$25,709,172*** |  |
| AWC | Burundi | $1,238,086 | $5,994,907 | $4,970,227 | $5,113,400 | **$4,329,155** | $12,987,465 | *$82,313,605* | ***$27,437,868*** |  |
| AWC | Cameroon | $4,795,474 | $4,918,659 | $12,095,653 | $13,997,226 | **$8,951,753** | $26,855,259 | *$155,188,052* | ***$51,729,351*** |  |
| AWC | Central Afr. Republic | $1,472,039 | $1,149,024 | $2,096,051 | $1,631,819 | **$1,587,233** | $4,761,700 | *$36,057,133* | ***$12,019,044*** |  |
| AWC | Chad | $4,998,517 | $472,685 | $1,245,547 | $2,139,291 | **$2,214,010** | $6,642,030 | *$64,144,667* | ***$21,381,556*** |  |
| AWC | Congo | $405,580 | $115,732 | $1,940,933 | $- | **$820,748** | $2,462,244 | *$27,539,011* | ***$9,179,670*** |  |
| AWC | Côte d'Ivoire | $1,723,045 | $1,962,582 | $2,350,334 | $201,513 | **$1,559,369** | $4,678,106 | *$112,936,762* | ***$37,645,587*** |  |
| AWC | Democratic Republic of Congo | $3,211,513 | $11,644,894 | $9,997,207 | $5,591,307 | **$7,611,230** | $22,833,691 | *$164,600,722* | ***$54,866,907*** |  |
| AWC | Equatorial Guinea | $- | $- | $- | $- | **None** | $- | *$-* | ***$-*** |  |
| AWC | Gabon | $232,857 | $- | $- | $- | **None** | $- | *$208,014* |  |  |
| AWC | Gambia ***** | $742,474 | $491,511 | $1,348,580 | $676,438 | **$814,751** | $2,444,252 | *$12,386,594* | ***$4,128,864.67*** |  |
| AWC | Ghana | $7,780,929 | $- | $17,034,873 | $- | **$12,407,901** | $37,223,703 | *$121,199,505* | ***$40,399,835*** |  |
| AWC | Guinea | $1,498,801 | $2,906,874 | $3,477,931 | $2,980,321 | **$2,715,982** | $8,147,946 | *$40,961,363* | ***$13,653,788*** |  |
| AWC | Guinea-Bissau | $761,639 | $497,486 | $2,421,313 | $1,661,279 | **$1,335,429** | $4,006,287 | *$18,153,166* | ***$6,051,055*** |  |
| AWC | Liberia | $1,071,129 | $- | $1,103,113 | $- | **$1,087,121** | $3,261,362 | *$45,238,243* | ***$15,079,414*** |  |
| AWC | Mauritania | $191,688 | $480,875 | $100,608 | $636,622 | **$352,448** | $1,057,345 | *$11,514,802* | ***$3,838,267*** |  |
| AWC | Niger | $1,266,644 | $402,079 | $1,657,318 | $1,857,688 | **$1,295,932** | $3,887,797 | *$24,277,913* | ***$8,092,638*** |  |
| AWC | Nigeria | $10,101,175 | $25,861,171 | $18,448,208 | $84,279 | **$13,623,708** | $40,871,125 | *$477,385,351* | ***$159,128,450*** |  |
| AWC | Senegal ***** | $1,935,456 | $709,747 | $915,565 | $- | **$1,186,923** | $3,560,768 | *$9,651,658* | ***$3,217,219*** |  |
| AWC | Sierra Leone | $961,725 | $436,704 | $1,168,076 | $- | **$855,501** | $2,566,504 | *$48,182,645* | ***$16,060,882*** |  |
| AWC | Togo | $- | $2,365,301 | $9,878,427 | $- | **$6,121,864** | $18,365,592 | *$52,323,470* | ***$17,441,157*** |  |
| AP | Bangladesh | $116,041 | $14,924 | $17,355 | $- | **$49,440** | $148,320 | *$34,547,968* | ***$11,515,989*** |  |
| AP | Bhutan | $14,755 | $18,225 | $16,585 | $48,082 | **$24,412** | $73,235 | *$2,441,702* | ***$813,901*** |  |
| AP | Cambodia | $9,898,191 | $3,635,103 | $4,313,260 | $- | **$5,948,851** | $17,846,554 | *$75,336,794* | ***$25,112,265*** |  |
| AP | China | $6,163,840 | $16,299,916 | $1,143,210 | $- | **None** | $- | *$-* | ***$-*** |  |
| AP | India | $84,920,136 | $35,815,218 | ***$73,197,577^a^*** | ***$4,227,264^b^*** | **$37,746,686** | $113,240,059 | *$562,317,917* | ***$187,439,306*** |  |
| AP | Indonesia | $2,042,784 | $2,438,324 | $- | $794,218 | **$1,758,442** | $5,275,326 | *$107,435,615* | ***$35,811,872*** |  |
| AP | Lao PDR | $1,172,781 | $- | $425,150 | $433,883 | **$677,271** | $2,031,814 | *$13,773,112* | ***$4,591,037*** |  |
| AP | Malaysia | $- | $- | $- | $- | **None** | $- | *$6,827,691* |  |  |
| AP | Myanmar | $2,613,759 | $4,668,943 | $12,737,355 | $2,768,477 | **$5,697,133** | $17,091,400 | *$117,663,027* | ***$39,221,009*** |  |
| AP | Nepal | $1,056,431 | $1,186,328 | $735,302 | $669,635 | **$911,924** | $2,735,771 | *$38,212,748* | ***$12,737,583*** |  |
| AP | Pakistan | $57,912 | $452,495 | $708,622 | $154,933 | **$343,490** | $1,030,471 | *$28,468,596* | ***$9,489,532*** |  |
| AP | Papua New Guinea ***** | $- | $- | $- | $- | **None** | $- | *$7,754,208* | ***$2,584,736*** |  |
| AP | Philippines ***** | $156,162 | $1,417,337 | $98,481 | $279,396 | **$487,844** | $1,463,532 | *$12,008,333* | ***$4,002,777.67*** | *$1,118,947* |
| AP | Sri Lanka | $70,385 | $96,187 | $187,592 | $135,315 | **$122,370** | $367,109 | *$12,905,398* | ***$4,301,799*** |  |
| AP | Thailand | $1,708,463 | $528,681 | $1,085,634 | $648,225 | **$992,751** | $2,978,252 | *$50,626,176* | ***$16,875,392*** |  |
| AP | Viet Nam ***** | $3,437,690 | $3,293,673 | $7,890,526 | $2,019,526 | **$4,160,354** | $12,481,061 | *$7,997,928* | ***$2,665,976*** |  |
| LAC | Bahamas | $- | $- | $- | $- | **None** | $- | *$-* | ***$-*** |  |
| LAC | Barbados | $- | $- | $- | $- | **None** | $- | *$-* | ***$-*** |  |
| LAC | Belize | $- | $- | $- | $- | **None** | $- | *$3,222,174* |  |  |
| LAC | Cuba | $4,464,467 | $2,036,583 | $- | $1,414,461 | **$2,638,504** | $7,915,511 | *$21,820,228* | ***$7,273,409*** |  |
| LAC | Dominican Republic | $2,476,696 | $1,498,314 | $5,756,912 | $- | **$3,243,974** | $9,731,922 | *$37,001,437* | ***$12,333,812*** |  |
| LAC | Guyana | $872,375 | $631,030 | $358,408 | $877,916 | **$684,932** | $2,054,797 | *$13,554,007* | ***$4,518,002*** |  |
| LAC | Haiti | $2,067,154 | $2,887,523 | $1,187,012 | $1,836,236 | **$1,994,481** | $5,983,444 | *$78,879,167* | ***$26,293,056*** |  |
| LAC | Jamaica | $1,870,134 | $1,590,053 | $4,353,633 | $269,056 | **$2,020,719** | $6,062,156 | *$19,133,368* | ***$6,377,789*** |  |
| LAC | Suriname | $106,125 | $- | $- | $- | **None** | $- | *$2,564,298* |  |  |
| LAC | Trinidad and Tobago | $- | $- | $- | $- | **None** | $- | *$-* | ***$-*** |  |
| EECA | Armenia | $215,889 | $127,160 | $234,889 | $292,088 | **$217,506** | $652,519 | *$9,760,915* | ***$3,253,638*** |  |
| EECA | Azerbaijan | $211,818 | $335,526 | $- | $325,389 | **$290,911** | $872,733 | *$19,817,688* | ***$6,605,896*** |  |
| EECA | Belarus | $2,143,808 | $1,258,814 | $1,577,848 | $1,742,215 | **$1,680,671** | $5,042,013 | *$20,577,582* | ***$6,859,194*** |  |
| EECA | Bulgaria | $698,497 | $- | $- | $561,734 | **$630,115** | $1,890,346 | *$9,240,256* | ***$3,080,085*** |  |
| EECA | Georgia | $667,645 | $854,383 | $1,154,047 | $385,968 | **$765,511** | $2,296,533 | *$33,886,454* | ***$11,295,485*** |  |
| EECA | Kyrgyzstan | $225,891 | $198,923 | $68,989 | $91,139 | **$146,236** | $438,707 | *$29,125,391* | ***$9,708,464*** |  |
| EECA | Moldova | $525,678 | $566,022 | $1,155,995 | $469,197 | **$679,223** | $2,037,669 | *$19,562,222* | ***$6,520,741*** |  |
| EECA | Romania | $- | $- | $- | $- | **None** | $- | *$-* | ***$-*** |  |
| EECA | Russia | $- | $- | $- | $- | **None** | $- | *$15,716,637* |  |  |
| EECA | Serbia | $- | $- | $- | $- | **None** | $- | *$-* | ***$-*** |  |
| EECA | Ukraine | $4,172,546 | $7,468,172 | $10,276,821 | $11,529,949 | **$8,361,872** | $25,085,616 | *$137,283,941* | ***$45,761,314*** |  |
| EECA | Uzbekistan | $1,208,838 | $1,387,534 | $2,375,775 | $736,343 | **$1,427,122** | $4,281,367 | *$27,727,733* | ***$9,242,578*** |  |
| MENA | Algeria | $- | $- | $- | $- | **None** | $- | *$6,533,577* |  |  |
| MENA | Djibouti | $476,145 | $131,631 | $337,585 | $- | **$315,121** | $945,362 | *$5,953,853* | ***$1,984,618*** |  |
| MENA | Egypt | $106,563 | $113,424 | $201,459 | $290,115 | **$177,890** | $533,670 | *$7,040,469* | ***$2,346,823*** |  |
| MENA | Iran | $95,591 | $61,803 | $550,459 | $166,547 | **$218,600** | $655,800 | *$20,238,295* | ***$6,746,098*** |  |
| MENA | Morocco | $641,184 | $532,015 | $665,025 | $317,415 | **$538,910** | $1,616,730 | *$24,605,906* | ***$8,201,969*** |  |
| MENA | Somalia ***** | $381,673 | $243,730 | $235,667 | $313,652 | **$293,680** | $881,041 | *$20,614,311* | ***$6,871,437*** | *$1,765,506* |
| MENA | Sudan | $517,868 | $519,745 | $131,292 | $849,362 | **$504,567** | $1,513,700 | *$38,005,683* | ***$12,668,561*** |  |
| MENA | Tunisia | $45,041 | $89,308 | $173,973 | $13,922 | **$80,561** | $241,683 | *$12,305,077* | ***$4,101,692*** |  |
| MENA | Yemen | $59,270 | $- | $164,914 | $- | **$112,092** | $336,275 | *$11,532,213* |  |  |
| LAC | Bolivia | $614,968 | $283,240 | $436,869 | $260,805 | **$398,970** | $1,196,911 | *$15,556,795* | ***$5,185,598*** |  |
| LAC | Guatemala | $736,850 | $2,056,993 | $1,982,668 | $1,486,571 | **$1,565,770** | $4,697,311 | *$44,836,281* | ***$14,945,427*** |  |
| LAC | Honduras | $181,759 | $61,906 | $145,878 | $86,241 | **$118,946** | $356,838 | *$20,412,040* | ***$6,804,013*** |  |
| LAC | Nicaragua | $507,075 | $430,398 | $557,350 | $3,957 | **$374,695** | $1,124,084 | *$17,390,461* | ***$5,796,820*** |  |
| AWC | Mali | $3,371,512 | $3,874,857 | $4,950,123 | $7,293,963 | **$4,872,614** | $14,617,841 | *$110,607,759* | ***$36,869,253*** |  |
| AP | Mongolia | $5,698 | $12,974 | $549 | $- | **$6,407** | $19,222 | *$5,832,818* | ***$1,944,273*** |  |
| EECA | Tajikistan | $2,732 | $286,136 | $342,312 | $21,237 | **$163,104** | $489,313 | *$24,662,107* | ***$8,220,702*** |  |
| EECA | Kazakhstan | $- | $314,443 | $- | $- | **None** | $- | *$-* | ***$-*** |  |

*Countries with recently updated NFM allocations. Where applicable and known, incentive funding that will be used for ART was incorporated into the analysis (Mozambique, Tanzania, Somalia).

^a^ India values in this table for 2012 and 2013 were based on analysis of procurements conducted by RITES, a Government of India parastatal enterprise, on behalf of the GFATM principal recipient in India (Ministry of Health and Family Welfare). Data are publicly available at <http://new.rites.com/index.php?page=page&id=65&name=Supply%20Tenders%20&mid=73>

**Table B. Global Fund commodity contributions from Country NFM modules (2014 US$)**

| **Country** | **2015** | **2016** | **2017** | **2018** |
| --- | --- | --- | --- | --- |
| Kenya | $64,996,468 | $72,642,724 | $7,464,835 | $24,546,109 |
| Mozambique | $66,178,641 | $52,084,800 |  |  |
| Tanzania | $65,776,756 | $146,908,977 |  |  |
| Gambia | $2,082,873 | $1,814,667.04 | $1,851,414 |  |
| Ghana | $7,671,534 | $19,207,449 | $22,515,254 |  |
| Ukraine | $12,251,363 | $16,684,018 | $7,116,937 |  |

* Sources: see Table A in S5 Text for third party contact information

**Table C. Global Fund 2015 country eligibility criteria, and counterpart financing thresholds (CFT)**

| **Country / Economy** | **Component** | **Global Fund**  **Income Category** | **Disease**  **Burden** | **Eligible for**  **funding** | **CFT  Minimum** |
| --- | --- | --- | --- | --- | --- |
| Afghanistan | HIV | LI | moderate | Yes | 5% |
| Albania | HIV | Upper-LMI | low | Yes | 40% |
| Algeria | HIV | UMI | high | Yesᵠ | 60% |
| Angola | HIV | UMI | severe | Yes | 60% |
| Armenia | HIV | Upper-LMI | high | Yes | 40% |
| Azerbaijan | HIV | UMI | high | Yesᵠ | 60% |
| Bangladesh | HIV | Lower-LMI | low | Yes | 20% |
| Belarus | HIV | UMI | high | Yesᵠ | 60% |
| Belize | HIV | UMI | high | Yesᵠ | 60% |
| Benin | HIV | LI | high | Yes | 5% |
| Bhutan | HIV | Lower-LMI | low | Yes | 20% |
| Bolivia | HIV | Lower-LMI | high | Yes | 20% |
| Botswana | HIV | UMI | extreme | Yes | 60% |
| Bulgaria | HIV | UMI | high | Yesᵠ, NGO rule† | 60% |
| Burkina Faso | HIV | LI | high | Yes | 5% |
| Burundi | HIV | LI | high | Yes | 5% |
| Cambodia | HIV | LI | high | Yes | 5% |
| Cameroon | HIV | Lower-LMI | severe | Yes | 20% |
| Cape Verde | HIV | Upper-LMI | high | Yes | 40% |
| Central African Republic | HIV | LI | severe | Yes | 5% |
| Chad | HIV | LI | severe | Yes | 5% |
| Colombia | HIV | UMI | high | Yesᵠ | 60% |
| Comoros | HIV | LI | severe | Yes | 5% |
| Congo | HIV | Lower-LMI | severe | Yes | 20% |
| Costa Rica | HIV | UMI | high | Yesᵠ | 60% |
| Côte d'Ivoire | HIV | Lower-LMI | severe | Yes | 20% |
| Cuba | HIV | UMI | high | Yesᵠ | 60% |
| Democratic People Republic of Korea | HIV | LI | low | Yes | 5% |
| Democratic Republic of the Congo | HIV | LI | high | Yes | N/A |
| Djibouti | HIV | Lower-LMI | high | Yes | 20% |
| Dominica | HIV | UMI | high | Yesᵠ | 60% |
| Dominican Republic | HIV | UMI | high | Yesᵠ | 60% |
| Ecuador | HIV | UMI | high | Yesᵠ | 60% |
| Egypt | HIV | Upper-LMI | moderate | Yes | 40% |
| El Salvador | HIV | Upper-LMI | high | Yes | 40% |
| Eritrea | HIV | LI | high | Yes | 5% |
| Ethiopia | HIV | LI | high | Yes | 5% |
| Fiji | HIV | UMI | low | Not eligible | 60% |
| Gabon | HIV | UMI | severe | Yes | 60% |
| Gambia | HIV | LI | high | Yes | 5% |
| Georgia | HIV | Upper-LMI | high | Yes | 40% |
| Ghana | HIV | Lower-LMI | high | Yes | 20% |
| Grenada | HIV | UMI | low | Yesᵠ | 60% |
| Guatemala | HIV | Upper-LMI | high | Yes | 40% |
| Guinea | HIV | LI | high | Yes | 5% |
| Guinea-Bissau | HIV | LI | severe | Yes | 5% |
| Guyana | HIV | Upper-LMI | high | Yes | 40% |
| Haiti | HIV | LI | severe | Yes | 5% |
| Honduras | HIV | Lower-LMI | high | Yes | 20% |
| India | HIV | Lower-LMI | high | Yes | 20% |
| Indonesia | HIV | Upper-LMI | high | Yes | 40% |
| Iran (Islamic Republic of) | HIV | UMI | high | Yesᵠ | 60% |
| Iraq | HIV | UMI | low | Not eligible | 60% |
| Jamaica | HIV | UMI | high | Yesᵠ | 60% |
| Kazakhstan | HIV | UMI | moderate | Not eligible | 60% |
| Kenya | HIV | Lower-LMI | severe | Yes | 20% |
| Kiribati | HIV | Lower-LMI | low | Yes | 20% |
| Kosovo | HIV | Upper-LMI | low | Yes | 40% |
| Kyrgyzstan | HIV | LI | high | Yes | 5% |
| Lao People Democratic Republic | HIV | Lower-LMI | low | Yes | 20% |
| Lesotho | HIV | Lower-LMI | extreme | Yes | 20% |
| Liberia | HIV | LI | moderate | Yes | 5% |
| Madagascar | HIV | LI | high | Yes | 5% |
| Malawi | HIV | LI | extreme | Yes | 5% |
| Malaysia | HIV | UMI | high | Yesᵠ | 60% |
| Maldives | HIV | UMI | low | Yesᵠ | 60% |
| Mali | HIV | LI | high | Yes | 5% |
| Mauritania | HIV | Lower-LMI | high | Yes | 20% |
| Mauritius | HIV | UMI | high | Yesᵠ | 60% |
| Micronesia | HIV | Upper-LMI | low | Yes | 40% |
| Mongolia | HIV | UMI | high | Yes | 60% |
| Morocco | HIV | Upper-LMI | high | Yes | 40% |
| Mozambique | HIV | LI | extreme | Yes | 5% |
| Myanmar | HIV | Lower-LMI | high | Yes | 20% |
| Namibia | HIV | UMI | extreme | Yes | 60% |
| Nepal | HIV | LI | high | Yes | 5% |
| Nicaragua | HIV | Lower-LMI | high | Yes | 20% |
| Niger | HIV | LI | high | Yes | 5% |
| Nigeria | HIV | Lower-LMI | severe | Yes | 20% |
| Pakistan | HIV | Lower-LMI | high | Yes | 20% |
| Panama | HIV | UMI | high | Yesᵠ | 60% |
| Papua New Guinea | HIV | Lower-LMI | high | Yes | 20% |
| Paraguay | HIV | Upper-LMI | high | Yes | 40% |
| Peru | HIV | UMI | high | Yesᵠ | 60% |
| Philippines | HIV | Lower-LMI | high | Yes | 20% |
| Republic of Moldova | HIV | Lower-LMI | high | Yes | 20% |
| Romania | HIV | UMI | high | Yesᵠ, NGO rule† | 60% |
| Russian Federation | HIV | HI | high | Yesᵠ, grace-period‡,  NGO rule† | N/A |
| Rwanda | HIV | LI | severe | Yes | 5% |
| Samoa | HIV | Upper-LMI | low | Yes | 40% |
| Sao Tome & Principe | HIV | Lower-LMI | high | Yes | 20% |
| Senegal | HIV | Lower-LMI | high | Yes | 20% |
| Seychelles | HIV | UMI | high | Yesᵠ | 60% |
| Sierra Leone | HIV | LI | high | Yes | 5% |
| Solomon Islands | HIV | Lower-LMI | low | Yes | 20% |
| Somalia | HIV | LI | moderate | Yes | N/A |
| South Africa | HIV | UMI | extreme | Yes | 60% |
| Sri Lanka | HIV | Upper-LMI | low | Yes | 40% |
| St Lucia | HIV | UMI | high | Yesᵠ | 60% |
| St Vincent & Grenadines | HIV | UMI | high | Yesᵠ | 60% |
| Sudan | HIV | Lower-LMI | low | Yes | 20% |
| Sudan South | HIV | LI | severe | Yes | 5% |
| Suriname | HIV | UMI | high | Yesᵠ | 60% |
| Swaziland | HIV | Upper-LMI | extreme | Yes | 40% |
| Syrian Arab Republic | HIV | Upper-LMI | low | Yes | 40% |
| Tajikistan | HIV | Lower- LMI | high | Yes | 20% |
| Tanzania, United Republic (Mainland) | HIV | LI | severe^^ | Yes | 5% |
| Tanzania, United Republic (Zanzibar) | HIV | LI | severe^^ | Yes | 5% |
| Thailand | HIV | UMI | high | Yesᵠ | 60% |
| Timor-Leste | HIV | Upper-LMI | low | Yes | 40% |
| Togo | HIV | LI | severe | Yes | 5% |
| Tonga | HIV | UMI | low | Yesᵠ | 60% |
| Tunisia | HIV | UMI | high | Yesᵠ | 60% |
| Turkmenistan | HIV | UMI | low | Not eligible | 60% |
| Tuvalu | HIV | UMI | low | Yesᵠ | 60% |
| Uganda | HIV | LI | severe | Yes | 5% |
| Ukraine | HIV | Upper-LMI | high | Yes | 40% |
| Uzbekistan | HIV | Lower-LMI | high | Yes | 20% |
| Vanuatu | HIV | Upper-LMI | low | Yes | 40% |
| Viet Nam | HIV | Lower-LMI | high | Yes | 20% |
| West Bank and Gaza | HIV | Upper-LMI | low | Yes | 40% |
| Yemen | HIV | Lower-LMI | high | Yes | 20% |
| Zambia | HIV | Lower-LMI | extreme | Yes | 20% |
| Zimbabwe | HIV | LI | extreme | Yes | 5% |

ᵠ: an applicant is eligible to receive a pre-defined maximum amount of funding for the eligible disease component, as part of the allocation methodology assessed by the Strategy, Investment and Impact Committee.

†: NGO Rule

For HIV/AIDS only, UMIs not listed on the OECD-DAC list of ODA recipients will only be able to access funding if they meet all the conditions below, in particular the confirmation that the services requested in the application are not being provided due to political barriers. In all cases, such applications must be submitted by an NGO and must comply with all of the following conditions:

a. Such country has a reported disease burden of ‘high’, ‘severe’ or ‘extreme’;

b. The application is submitted and the program will be managed by an NGO within the country in which activities would be implemented;

c. The government of a such country shall not directly receive any funding;

d. Requests are submitted as a non-CCM or other valid application;

e. Such funding requests must meet the ‘focus of application’ requirements set forth in Paragraph 17 of the ECFP4 and must demonstrate that they target key services, as supported by evidence and the country’s epidemiology; and

f. Applicants must provide confirmation that the services requested in the application are not being provided due to political barriers.

‡: Grace period :countries with an existing HIV grant under the NGO rule that become ineligible due to changes in income level are provided with a grace-period of one allocation period; such countries must continue to meet the conditions of the NGO rule

<http://www.theglobalfund.org/en/fundingmodel/updates/2015-02-27_2015_Eligibility_list_for_2015_released/>

**Table D. Domestic contributions for ART: Summary of factors applied (X: applied)**

| **Region** | **Country** | **CFT** | **Commodity DC** | **Non-commodity DC** |
| --- | --- | --- | --- | --- |
| AES | Angola |  | X* | X |
| AES | Botswana |  | X | X |
| AES | Comoros | X |  | X |
| AES | Eritrea | X |  | X |
| AES | Ethiopia | X | X* | X |
| AES | Kenya |  | X* | X |
| AES | Lesotho | X |  | X |
| AES | Madagascar | X |  | X |
| AES | Malawi | X |  | X |
| AES | Mauritius | X |  | X |
| AES | Mozambique | X |  | X |
| AES | Namibia |  | X* | X |
| AES | Rwanda | X |  | X |
| AES | South Africa |  | X* | X |
| AES | South Sudan | X |  | X |
| AES | Swaziland |  | X | X* |
| AES | Tanzania | X |  | X |
| AES | Uganda |  | X* | X |
| AES | Zambia | X |  | X |
| AES | Zimbabwe | X |  | X |
| AP | Bangladesh | X |  | X |
| AP | Bhutan | X |  | X |
| AP | Cambodia | X |  | X |
| AP | China |  | X | X |
| AP | India |  | X* | X |
| AP | Indonesia | X |  | X |
| AP | Laos | X |  | X |
| AP | Malaysia |  | X | X |
| AP | Mongolia | X |  | X |
| AP | Myanmar | X |  | X |
| AP | Nepal | X |  | X |
| AP | Pakistan | X |  | X |
| AP | Papua New Guinea |  | X | X |
| AP | Philippines | X |  | X |
| AP | Sri Lanka | X |  | X |
| AP | Thailand |  | X | X |
| AP | Viet Nam |  | X* | X |
| AWC | Benin | X |  | X |
| AWC | Burkina Faso | X |  | X |
| AES | Burundi | X |  | X |
| AWC | Cameroon | X |  | X |
| AWC | Central African Republic | X |  | X |
| AWC | Chad | X |  | X |
| AWC | Congo | X |  | X |
| AWC | Côte d'Ivoire | X |  | X |
| AWC | Democratic Republic of Congo | X |  | X |
| AWC | Equatorial Guinea | X |  | X |
| AWC | Gabon | X |  | X |
| AWC | Gambia | X |  | X |
| AWC | Ghana |  | X* | X |
| AWC | Guinea | X |  | X |
| AWC | Guinea-Bissau | X |  | X |
| AWC | Liberia | X |  | X |
| AWC | Mali | X |  | X |
| AWC | Mauritania | X |  | X |
| AWC | Niger | X |  | X |
| AWC | Nigeria | X |  | X |
| AWC | Senegal | X |  | X |
| AWC | Sierra Leone | X |  | X |
| AWC | Togo | X |  | X |
| EECA | Armenia | X |  | X |
| EECA | Azerbaijan | X |  | X |
| EECA | Belarus | X |  | X |
| EECA | Bulgaria | X |  | X |
| EECA | Georgia | X |  | X |
| EECA | Kazakhstan |  | X | X |
| EECA | Kyrgyzstan | X |  | X |
| EECA | Moldova | X |  | X |
| EECA | Romania |  | X | X |
| EECA | Russia |  | X | X |
| EECA | Serbia |  | X | X |
| EECA | Tajikistan | X |  | X |
| EECA | Ukraine | X |  | X |
| EECA | Uzbekistan | X |  | X |
| LAC | Bahamas |  | X | X |
| LAC | Barbados |  | X | X |
| LAC | Belize |  | X | X |
| LAC | Bolivia | X |  | X |
| LAC | Cuba | X |  | X |
| LAC | Dominican Republic |  | X* | X |
| LAC | Guatemala | X |  | X |
| LAC | Guyana | X |  | X |
| LAC | Haiti | X |  | X |
| LAC | Honduras | X |  | X |
| LAC | Jamaica | X |  | X |
| LAC | Nicaragua | X |  | X |
| LAC | Suriname |  | X | X |
| LAC | Trinidad and Tobago |  | X | X |
| MENA | Algeria |  | X | X |
| MENA | Djibouti | X |  | X |
| MENA | Egypt | X |  | X |
| MENA | Iran | X |  | X |
| MENA | Morocco | X |  | X |
| MENA | Somalia | X |  | X |
| MENA | Sudan | X |  | X |
| MENA | Tunisia | X |  | X |
| MENA | Yemen | X |  | X |

* Indicates that the domestic contribution total is shown in Table C

**Table E. Domestic contribution totals (not %) applied for certain countries (2014 US$)**

| **Country** | **Sources** | **Estimated Domestic Contribution towards ART commodities (2014 US$)** | | | | | | |
| --- | --- | --- | --- | --- | --- | --- | --- | --- |
|  |  | *2014  (not used)* | **2015** | **2016** | **2017** | **2018** | **2019** | **2020** |
| Swaziland* | [[9](#_ENREF_9)] | *N/A* | $19,036,480 | $19,955,911 | Continued | | | |
| Kenya | [[10](#_ENREF_10)] | *N/A* | $19,342,000 | Continued (based on 1.9 billion KES at US$0.01018 per KES) | | | | |
| India | [[11](#_ENREF_11)] | *$11,706,373* | $14,255,048 | Continued (based on RITES procurement orders) | | | | |
| Russia | [[12](#_ENREF_12)] | *$318,000,000* | $211,800,000 | Continued (based on 12 billion roubles, at current exchange rates) | | | | |
| Thailand | [[13](#_ENREF_13)] | *$92,820,000* | Continued | | | | | |
| Botswana | [[14](#_ENREF_14)] | *$143,000,000* | Continued (based on 1.3 billion pula, at US$0.11 per pula average for 2014) | | | | | |
| Namibia | [[15](#_ENREF_15)] | *$6,640,000* | $664,000 | Continued (at Namibian $166 mil., at US$0.08 per Namibian $) | | | | |
| South Africa | [[16](#_ENREF_16)] | *N/A* | $266,666,667 | $266,666,667 | $266,666,667 | Continued (R10 billion, split equally at US$0.08 per rand) | | |
| Uganda | [[17](#_ENREF_17)] | *N/A* | $32,723,788 | Continued (based on 84 billion UGX at US$0.0004 per UGX) | | | | |
| Angola | [[18](#_ENREF_18)] | *N/A* | $15,773,425 | Continued (for ARVs, lab reagents at US$0.0093 per kwanza) | | | | |
| Vietnam | [[19](#_ENREF_19)] | *N/A* | $1,083,264 | $1,155,583 | $1,232,730 | Continued (growth 2016-17 at 6.7% p.a.) | | |
| Dominican  Republic | [[20](#_ENREF_20)] | *$3,176,874* | Continued (value is an average of contributions over 2012, 2013) | | | | | |
| Ghana | NFM concept note | *N/A* | $3,367,046 | $3,954,339 | $4,918,270 | Continued (based on NFM concept note and value of commodities. See SI.15) | | |
| Swaziland*  (non commodity) | [[9](#_ENREF_9)] | *N/A* | $6,732,443 | $7,069,066 | Continued | | | |

* Information for Swaziland's domestic contribution towards non-commodity resource needs was also available.

**Table F. Estimate of annual PEPFAR support to countries, 2015-2020 (2014 US$)**

| **Countries** | **ARVs** | **Lab commodities** | **Conservative: site-level** | **Expansive: site-level** |
| --- | --- | --- | --- | --- |
| Angola | $0 | $0 | $0 | $0 |
| Botswana | $131,858 | $0 | $3,846,646 | $5,634,472 |
| Myanmar | $0 | $0 | $813,574 | $1,176,260 |
| Burundi | $1,208,202 | $0 | $789,757 | $1,186,177 |
| Cambodia | $1,321 | $0 | $1,916,926 | $2,771,479 |
| Cameroon | $147,228 | $0 | $535,630 | $1,321,382 |
| Cote d'Ivoire | $15,153,200 | $162,789 | $12,759,889 | $22,057,005 |
| Dominican Republic | $0 | $0 | $233,302 | $333,668 |
| Democratic Republic of Congo | $1,483,576 | $0 | $4,455,717 | $8,329,545 |
| Ethiopia | $229,147 | $9,019,710 | $16,406,282 | $27,899,311 |
| Ghana | $0 | $0 | $0 | $0 |
| Guyana | $459,493 | $0 | $541,485 | $774,431 |
| Haiti | $3,002,618 | $2,932,450 | $18,840,220 | $26,945,247 |
| India | $0 | $0 | $856,106 | $1,237,752 |
| Indonesia | $0 | $0 | $109,600 | $158,459 |
| Kenya | $58,513,172 | $26,917,148 | $37,612,973 | $52,227,095 |
| Lesotho | $20,560 | $5,000 | $2,387,081 | $3,787,130 |
| Malawi | $15,341 | $642,267 | $6,278,509 | $10,269,162 |
| Mozambique | $20,458,428 | $10,059,795 | $33,986,804 | $49,882,752 |
| Namibia | $873,985 | $35,771 | $6,080,773 | $8,293,379 |
| Nigeria | $47,215,534 | $18,863,489 | $65,464,442 | $101,587,726 |
| Papua New Guinea | $0 | $0 | $343,580 | $496,746 |
| Rwanda | $5,776,659 | $9,591,415 | $4,746,796 | $8,248,460 |
| South Africa | $2,676,973 | $102,545 | $103,349,358 | $130,835,565 |
| South Sudan | $0 | $0 | $1,136,931 | $1,707,616 |
| Swaziland | $0 | $2,361 | $5,233,080 | $7,512,800 |
| Tanzania* | $30,960,722 | $10,086,004 | $44,953,851 | $64,540,477 |
| Uganda | $49,113,785 | $11,010,782 | $51,250,124 | $67,686,398 |
| Ukraine | $0 | $0 | $1,321,010 | $2,047,359 |
| Vietnam (commodities till 2016) | $9,505,668 | $1,403,102 | $6,159,729 | $8,905,694 |
| Zambia | $25,962,160 | $13,690,022 | $26,761,704 | $45,891,396 |
| Zimbabwe | $12,033,840 | $2,656,443 | $5,343,693 | $9,992,525 |
| **Total** | **$284,943,470** | **$117,181,093** | **$464,515,571** | **$673,737,468** |

Source: FY 2013 expenditures (http://data.pepfar.net) and FY 2014 planned treatment budgets [[8](#_ENREF_8)]

*Data from COP 2015

**REFERENCES**

1. The Global Fund to Fight AIDS TaM Price and Quality Reporting. Available at: <http://www.theglobalfund.org/en/procurement/pqr/>.

2. The Global Fund to Fight AIDS TaM (2014) A Quick Guide to the Global Fund’s Price and Quality Reporting System (PQR).

3. The Global Fund (2014) The Global Fund: ARV Acquisition Strategy (presentation at PEPFAR ARV Supplier Conference: June 24-25, 2014). Dubai, UAE: The Global Fund to Fight AIDS, Tuberculosis and Malaria.

4. The Global Fund to Fight AIDS TaM Eligibility List 2014.

5. Global Fund to Fight AIDS T, and Malaria, (2013) The Global Fund Eligibility and Counterpart Financing Policy. .

6. (2014) AIDSinfo Online Database.

7. Birx DL (2015) Rights things in the right places, right now for epidemic control and an AIDS-free generation. PEPFAR Scientific Advisory Board. Washington, DC.

8. OGAC (2014) Congressional Budget Justification Supplement, Fiscal Year 2014 - President's Emergency Plan for AIDS Relief (PEPFAR). Washignton, DC: Office of the United States Global AIDS Coordinator (OGAC).

9. Hunger J, Derrick D, Korah G (2014) Increasing Domestic Investment (PowerPoint Presentation). Geneva, Switzerland: The Global Fund.

10. Government of Kenya (2015) Preliminary FY 2015/16 Budget Allocations. Nairobi, Kenya.

11. RITES (2015) Procurement orders available at <http://new.rites.com/index.php?page=page&id=65&name=Supply%20Tenders%20&mid=73> Accessed May 11, 2015. New Delhi, India: RITES.

12. ITPCru, EVA, Simona+ (2012) To treat or not to treat? Report based on the results of community research: ARV treatment procurement and provision in Russia. St. Petersburg, Russia.

13. Sorakii Bhakeecheep (2013) ART Program Management under Universal Health Coverage, Presentation at 7th IAS Conference on HIV Pathogenesis, Treatment and Prevention (Kuala Lumpur, Malaysia). National Health Security Office, Government of Thailand.

14. Sennamose O (2013) Government spends P1.3 billion on ARVs (<http://www.dailynews.gov.bw/news-details.php?nid=5293>, accessed May 11, 2015). Botswana Daily News. Gaborone, Botswana: Botswana Daily News.

15. Kuzeeko Tjitemisa (2014) HOHSS awards multi-million tenders (https://[www.newera.com.na/2014/08/08/mohss-awards-multi-million-tenders/](http://www.newera.com.na/2014/08/08/mohss-awards-multi-million-tenders/) accessed May 11, 2015). New Era. Windhoek, Namibia.

16. Times Live (2014) Health Department awards R10 billion ARV tender (Dec. 24, 2014) <http://www.timeslive.co.za/politics/2014/12/24/health-department-awards-r10-billion-arv-tenders> accessed May 11, 2015. Johannesburg, South Africa.

17. Government of Uganda (2015) National Budget Framework Paper FY 2015/16, Section 3: Health Sector. Kampala, Uganda.

18. Government of Angola (2015) Plano de Acção 2015 Revisto. Luanda, Angola: Government of Angola.

19. Kashi Barbara Carasso, Grace Chee, Altea Cico, Duong Hoang Quyen, Phan Cam Tu, et al. (2014) Options for Integrating Procurement and Supply Chain Systems for ARVs, Methadone, and anti-Tuberculosis Drugs in Vietnam. Bethesda, Maryland: Health Finance & Governance Project, Abt Associates Inc.

20. SIAPS (2013) Reevaluation of the HIV Financing Gap in the Framework of SUGEMI Planning for 2013. Santo Domingo, Dominican Republic: Systems for Improved Access to Pharmaceuticals and Services (SIAPS).
