# Supplementary material for: The HIV Treatment Gap: Estimates of the Financial Resources Needed versus Available for Scale-Up of Antiretroviral Therapy in 97 Countries from 2015 to 2020
Source: PLoS Med. 2015 Nov 24;12(11):e1001907. doi: 10.1371/journal.pmed.1001907 (PMC4658189; doi:10.1371/journal.pmed.1001907)
Supplement: S5 Text — (DOCX) [file pmed.1001907.s005.docx]

S5 Text. Data sources and availability

Most data in the study are from third party sources including government agencies, watchdog organizations, and external funding organizations. Readers may access data sources as follows:

1. Official Spectrum model projection files (81 of 97 countries) are downloadable by request using the form on the UNAIDS website (<http://apps.unaids.org/spectrum>), and the Spectrum Country Data Package (16 of 97 countries) is downloadable as a combined executable file which links with the Spectrum software from the Avenir Health website (<http://www.avenirhealth.org/Download/Spectrum/CountryDataInstall.EXE>)
2. Additional programmatic data, epidemiological data, and information on coverage of antiretroviral treatment are available from public sources which have been cited in the manuscript. These include reports filed by national ministries of health to UNAIDS, refereed journal papers, and online databases (e.g., on resistance to antiretrovirals). Additional epidemiological parameters used in the analytical models, especially Spectrum, are contained in the Spectrum software itself, which is freely downloadable and has been described in widely available papers. Other parameters used for epidemiological estimates are listed in the study narrative or supplementary information, with original sources cited.
3. Unit costs, past drug prices, and related estimates are from a variety of sources, nearly all publically available or downloadable from journal websites. We have cited these sources, such as the Global Price Reporting Mechanism (freely accessible on the World Health Organization website at http://www.who.int/hiv/amds/gprm/en/), the Global Fund's past transactions database (online and freely accessible at www.theglobalfund.org), and specific country-level cost and transactions data (e.g., India's drug procurement tenders from www.rites.com). A large number of unit costs are from previously published studies, which have been analyzed. Full assumptions for some lab unit cost data used from Global Fund concept note application and analyzed information is presented in this supplementary information for scrutiny and reproducibility.
4. PEPFAR has not made 2014 expenditures by country available. Therefore we have only used publically available information, from http://data.pepfar.net (i.e. 2013 expenditures and planned budgets) and the PEPFAR Congressional Budget Justification (supplement) for Fiscal Year 2014, available online without restriction.
5. Not all funding decisions from the Global Fund are made public and the supporting information, usually documentation and budget files, are not generally released. Major watchdog organizations routinely access data and make it available online (recently, see: http://data.aidspan.org/ for a freely accessible database of funding levels, allocations, etc.). In any case, information related to the budgetary information as used in the study derived from these "New Funding Model Concept Notes" (proposals made by countries to Global Fund) is either in tables located in S4 Text or can be sourced from this document: http://lacfondomundial.org/wp-content/uploads/2015/07/GF-B33-ER03-GAC-Report-to-the-Board-SENT.pdf which is also available online from a watchdog organization. These are the only significant Concept Note values used in the gap analysis in the study. On occasion, we used a few data elements which were in additional supporting documents submitted by countries to the Global Fund. The Global Fund has recently committed to a transparency initiative to release most of the "Concept Note" documents online (see http://www.aidspan.org/gfo_article/global-fund-announces-plans-make-some-concept-note-attachments-public), however, we are not aware of the timeline for this eventual release. Contact information are available in Table A for country/government representatives who can be reached in the case of clarifications on Global Fund funding levels for specific countries.

Almost all data used are those from public and not proprietary sources; and major analytical results should be broadly reproducible from these based on applying the same methodologies and software.

**Table A. Third Party Data Availability Statement**

| **Countries in SI. 8** | **Named Contact Information for Principal Recipient, Global Fund Grants** |
| --- | --- |
| Kenya | **Margaret ONYIMBO**  Global Fund Coordinator  100 Nairobi  Telephone: +254 2-22252299  Email: [onyimbom@yahoo.co.uk](mailto:onyimbom@yahoo.co.uk) |
| Mozambique | **Carla SILVA MATOS**  Address: Ministry of Health of Mozambique -  DPC Avenida Eduardo Mondlane - PO Box 264, Maputo  Telephone: -823139502  Email: [cmatos@misau.gov.mz](mailto:cmatos@misau.gov.mz) |
| Tanzania | **Fatma MRISHO**  Executive Chairman  Address: TACAIDS Dar es Salaam  Email: [fmrisho@tacaids.go.tz](mailto:fmrisho@tacaids.go.tz) |
| Gambia | **Ousman BADJIE**  Director  Address: NAS, FIB Building, Kairaba Avenue, KSMD, The Gambia  West Africa  Telephone: -9976991  Fax: -4395614  Email: [badjieous@hotmail.com](mailto:badjieous@hotmail.com) |
| Ghana | **Albert WUDDAH - MARTEY**  Programme Manager  Accra  Telephone: +233 21 310369  Fax: +233 21 304567  Email: [amartey@ppag-gh.org](mailto:amartey@ppag-gh.org) |
| Ukraine | **Natalya NIZOVA**  Director of Ukrainian AIDS Prevention Center  Address: Moskovskyi av., 19 04655  Kiev  Telephone: +380-44 287 34 16  Fax: +380-44 287 34 17  Email: [natalya.nizova@gmail.com](mailto:natalya.nizova@gmail.com) |
